# Reactivity of α-diazo sulfonium salts: rhodium-catalysed ring expansion of indenes to naphthalenes†

**DOI:** 10.1039/d4sc01138d

**Published:** 2024-03-25

**Authors:** Sven Timmann, Tun-Hui Wu, Christopher Golz, Manuel Alcarazo

**Affiliations:** a Institut für Organische und Biomolekulare Chemie, Georg August Universität Göttingen Tammannstr 2 37077 Göttingen Germany manuel.alcarazo@chemie.uni-goettingen.de

## Abstract

In the presence of catalytic amounts of the paddlewheel dirhodium complex Rh_2_(esp)_2_, α-diazo dibenzothiophenium salts generate highly electrophilic Rh-coordinated carbenes, which evolve differently depending on their substitution pattern. Keto-moieties directly attached to the azomethinic carbon promote carbene insertion into one of the adjacent C–S bonds, giving rise to highly electrophilic dibenzothiopyrilium salts. This intramolecular pathway is not operative when the carbene carbon bears ester or trifluoromethyl substituents; in fact, these species react with olefins delivering easy to handle cyclopropyl-substituted sulfonium salts. When indenes are the olefins of choice, the initially formed cyclopropyl rings smoothly open with concomitant departure of dibenzothiophene, enabling access to a series of 2-functionalized naphthalenes.

## Abstract 2

Cyclopropyl-substituted sulfonium salts are obtained by Rh-catalysed addition of α-diazo dibenzothiophenium salts to olefins. When indenes are used as substrates, initially formed cyclopropyl rings open with concomitant elimination of dibenzothiophene, enabling access to 2-substituted naphthalenes.

## Graphical abstract

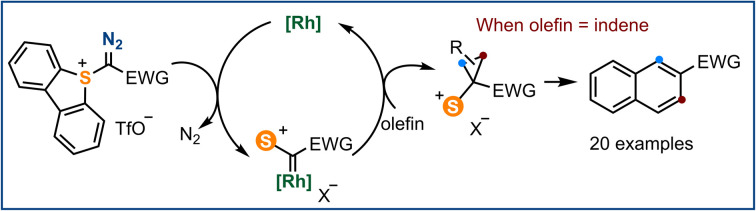

## Introduction

Arguably, the most prominent feature of sulfonium salts when compared with hypervalent I(iii)-reagents of analogous structure is their enhanced thermal stability.^1,2^ This property ultimately makes these species practical reagents for synthesis^3^ because: (i) it facilitates their handling even in large-scale;^4^ (ii) allows expedition of purifications, often through traditional chromatographic techniques,^5^ and importantly, (iii) it enables functional group manipulations to be carried out on these reagents after incorporation of the sulfonium moiety into their structures.^6^ This robustness is particularly manifested when the sulfonium salt bears other sensitive functional groups, and has recently been exploited for the design of sulfur-based reagents with no parallelism in the realm of hypervalent iodine species.^7^

**Scheme 1:**
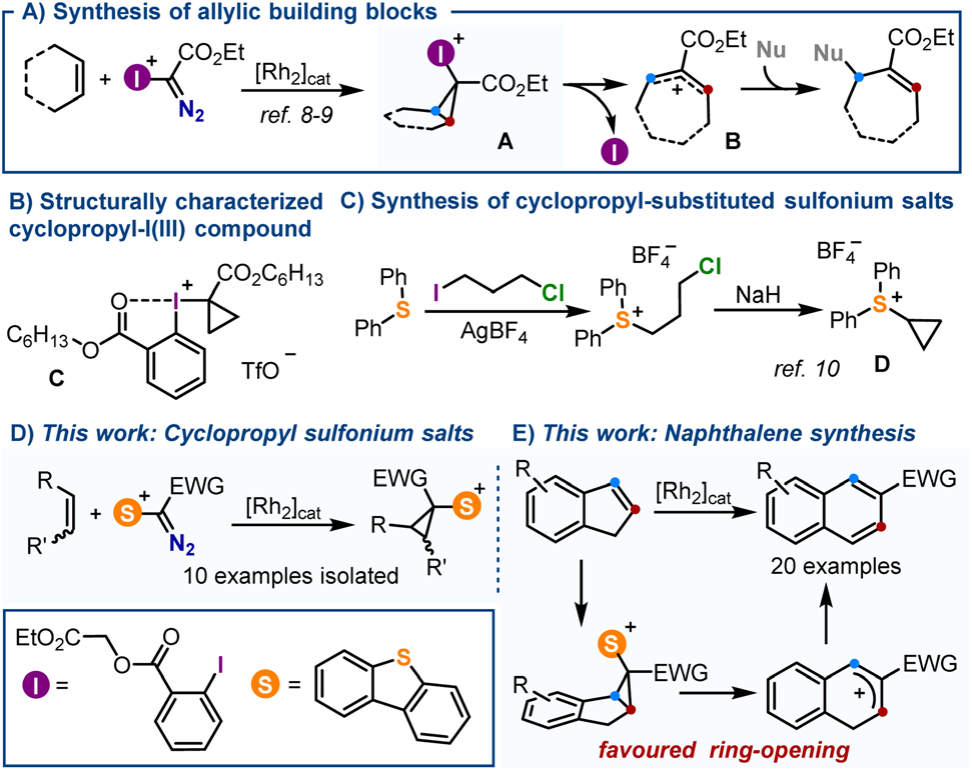
(A) Reactivity of cyclopropyl-I(iii) reagents; (B) only characterized cyclopropyl-substituted λ^3^-iodane; (C) classical synthesis of cyclopropyl-substituted sulfonium salts; (D) and (E) new synthesis of cyclopropyl-substituted sulfonium salts and reactivity studies.

Recently, Suero and co-workers reported the Rh-catalysed formation of cyclopropyl-I(iii) intermediates A by reaction of α-diazoiodonium salts with olefins.^8,9^ Such species smoothly evolve, even at temperatures as low as −50 °C, to synthetically useful allylic cations B*via* scission of their distal C–C bond with concomitant elimination of the iodine moiety (Scheme 1A). Due to this intrinsic reactivity, cyclopropyl-substituted iodonium salts have eluded systematic isolation and, in fact, compound C is the only member of the series that has been characterized (Scheme 1B).^8^ Contrarily, cyclopropyl-substituted sulfonium salts like D are relatively easy to handle, and have been utilized since decades for the synthesis of cyclobutanones (Scheme 1C);^10^ yet, their structural variability remains quite narrow. Since neither D or a structural derivative has been synthesized following an analogous [2 + 1] disconnection, nor their electrocyclic ring-opening has been studied, we decided to tackle both aspects.

Herein, we describe the synthesis of a series of new α-diazosulfonium salts and their transformation into the corresponding cyclopropyl-derivatives through the Rh-catalyzed addition of sulfoniocarbene moieties to olefins.^11^ This new route significantly expands the available structural diversity for cyclopropyl-substituted sulfonium salts, which are all isolated as crystalline materials after column chromatography. Subsequently, making use of the thermodynamically favored electrocyclic ring opening of the salts derived from indene, a protocol is implemented for the transformations of such compounds into 2-substituted naphthalenes (Scheme 1D and E).^12^

## Results and discussion

### Synthesis and structure of α-diazosulfonium salts

**Scheme 2:**
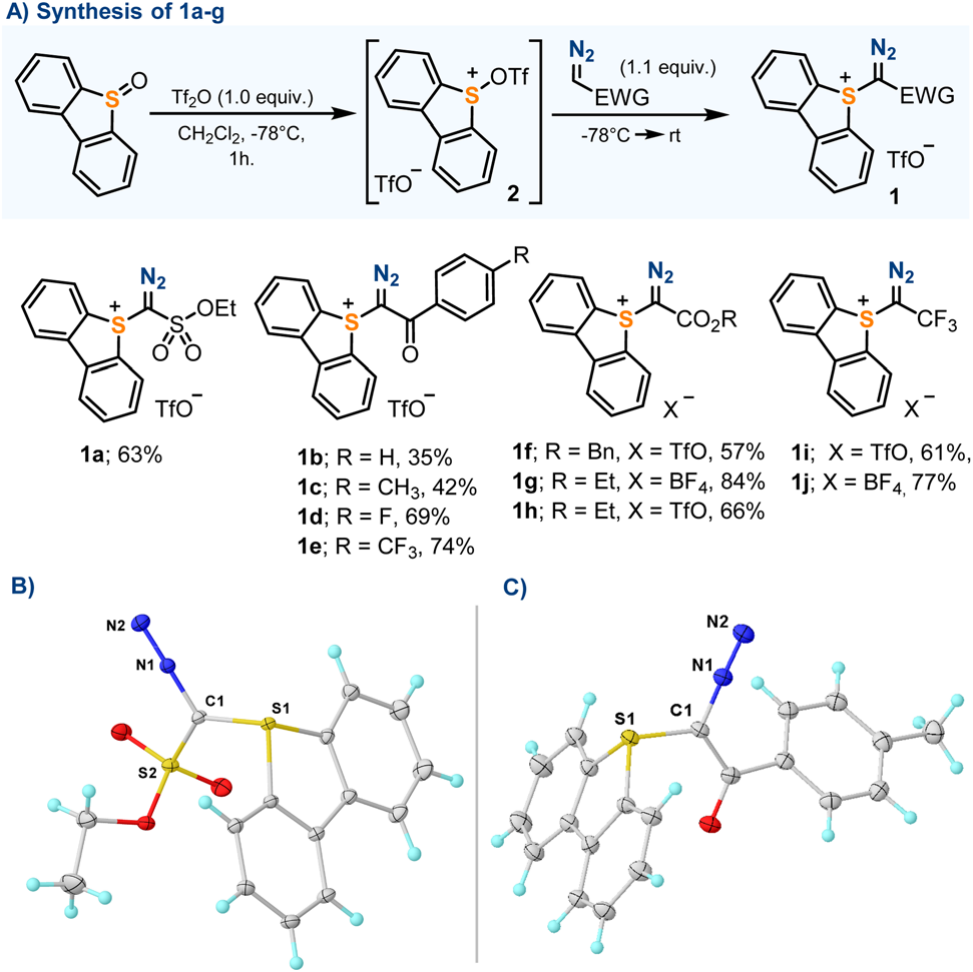
(A) Synthesis of α-diazosulfonium salts; (B) and (C) molecular structures in the solid state of compounds 1a and 1c, respectively. Anisotropic displacements shown at the 50% probability level. Triflate anions and solvent molecules omitted for clarity. Selected bond lengths [Å]: 1a: S1–C1, 1.728(1); C1–N1, 1.333(1); N1–N2, 1.113(1); 1c: S1–C1, 1.742(2); C1–N1, 1.339(2); N1–N2, 1.117(2).

Our initial efforts were focused on the synthesis of parent sulfonium salts 1a–f, all non-reported compounds that share the dibenzothiophene platform and an electron withdrawing group embedding the azomethine carbon. Compounds 1a–h were prepared without exception by reaction of the corresponding diazo compounds with *in situ* generated sulfurane 2.^13^ The reaction took place in moderate to good yields, and compounds 1a–g were isolated as pale-yellow crystalline solids (Scheme 2). Previously reported compounds 1h and 1i were included in this study for completeness.^13*b*^

The molecular structures of 1a–f have been determined by X-ray diffraction, confirming the expected connectivity (Scheme 2B and C and the ESI†). The central sulfur atom (S1) adopts for all compounds a trigonal-pyramidal coordination environment, with the sum of the bond angles around this atom falling within a narrow range (303.8–305.0°). The S1–C1 bond distances are the typical ones for S–C(sp^2^) single bonds (1.728–1.742 Å), and the C1–N1 lengths (1.338–1.331 Å) are identical, within the experimental error, to those found in non-charged diazo compounds.^14^ Salts 1a–f were also studied by simultaneous differential scanning calorimetry-thermogravimetric analysis (DSC-TGA). Sharp exothermic events were detected for all compounds, which start at 80–90 °C and lead to energy releases ranking between 332 J g^−1^ (for 1a) and 485 J g^−1^ (for 1c); however, on the basis of the Yoshida correlation, they are not expected to be explosive or impact sensitive.^15^ The heat release events observed are coupled with acute mass losses that are probably related to the decomposition of the diazo unit.

### Synthesis of cyclopropyl-substituted sulfonium salts

**Scheme 3:**
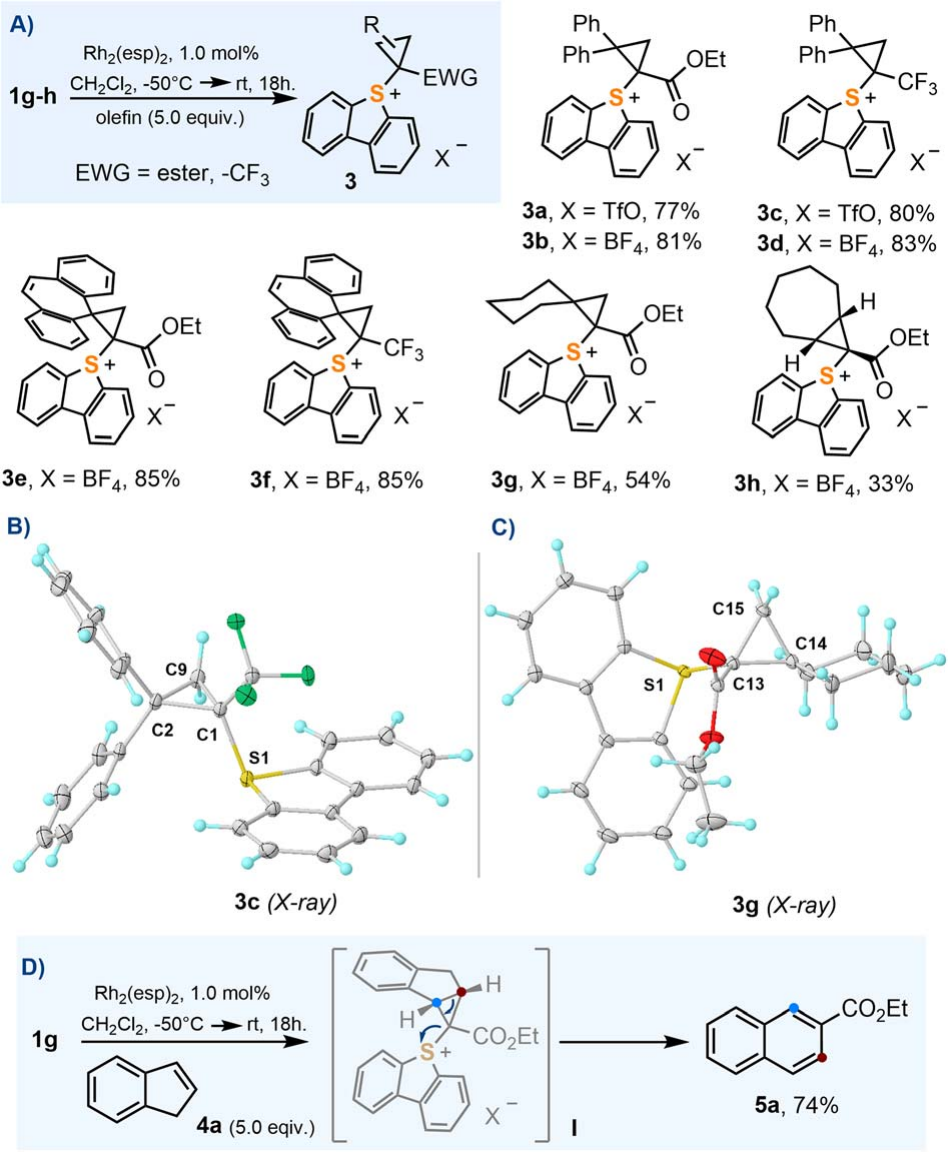
(A) Synthesis of cyclopropyl-substituted sulfonium salts; (B) and (C) molecular structures in the solid state of compounds 3c and 3g, respectively. Anisotropic displacements shown at the 50% probability level. Solvent molecules and anions omitted for clarity. Selected bond lengths [Å]: 3c: S1–C1, 1.793(1); C1–C2, 1.520(1); C1–C9, 1.545(1); C2–C9, 1.500(2); 3g: S1–C13, 1.785(1); C13–C15, 1.522(1); C13–C14, 1.529(1); C14–C15, 1.522(1); (D) indene ring expansion to naphthalene 5a.

In order to evaluate the utility of 1a–j as cyclopropanation reagents, 1g–h were used as model compounds and the reaction conditions optimized by Suero for I(iii)-species were employed (Rh_2_(esp)_2_, 1 mol%; CH_2_Cl_2_, −50 °C → r.t.; olefin, 5.0 equiv.).^8^ The cyclopropanation reaction works particularly well for 1,1-disubstituted olefins 3a–g; cycloheptene also delivers the expected cyclopropane product 3h, albeit in moderate yield (Scheme 3A). In contrast to their I(iii) analogues,^16^ sulfonium salts 3a–h are all bench stable crystalline materials that can be stored without any precaution for months. Scheme 3B and C depict the molecular structures of 3c and 3g obtained by X-ray diffraction; molecular structures for 3f and 3h can be found in the ESI.† The sulfoniocarbene transfer reaction was further examined by employing indene as the olefin substrate; however, no cyclopropane-substituted sulfonium salt was obtained. Instead the product derived from a Ciamician–Dennstedt rearrangement,^17^ naphthalene 5a, was produced in a remarkable 74% yield. The scope and mechanistic details of this transformation are evaluated in the following section.

**Scheme 4:**
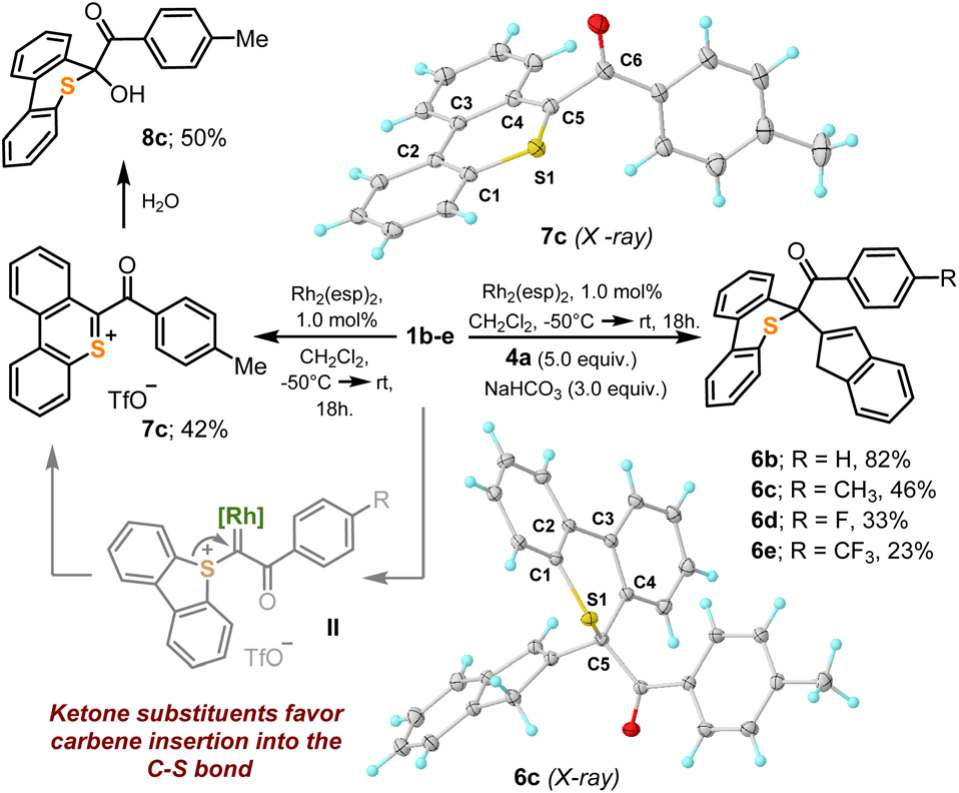
Carbene insertion in a S–C bond of dibenzothiophene. Molecular structures in the solid state of compounds 6c and 7c. Anisotropic displacements shown at the 50% probability level. Anions omitted for clarity. Selected bond lengths [Å]: 6c: S1–C1, 1.756(1); S1–C5, 1.841(1); C1–C2, 1.408(1); C2–C3, 1.481(1); C3–C4, 1.410(1); C4–C5, 1.526(1); 7c: S1–C1, 1.725(1); S1–C5, 1.654(1); C1–C2, 1.413(2); C2–C3, 1.456(2); C3–C4, 1.431(2); C4–C5, 1.417(2), C5–C6, 1.535(2).

Unfortunately, not all the α-diazo sulfonium salts studied get involved in the cyclopropanation of olefins. After initial formation of the Rh–carbene complex II, those featuring strong electron withdrawing ketone-substituents, 1b–e, preferentially evolve towards thiopyrilium cations 7*via* insertion of the carbene into one of the C–S bonds of the dibenzothiophene unit. We have been able to isolate and structurally characterize such salt in the case of 7c (Scheme 4). This competing ring expansion is a fast process for II because even when the reaction is carried out in the presence of 5.0 equivalents of indene, no naphthalene is observed. Instead, the products of nucleophilic attack of indene to the already formed thiopyrilium salts are isolated 6b–e. Addition of water to 7c, delivers, as expected, 8c.

### Naphthalene synthesis: scope and mechanism

The development of efficient methodologies that allow the insertion of new atoms into pre-existing (hetero)cyclic skeletons is particularly intriguing for synthetic chemists because such skeletal modifications often trigger profound changes in the physicochemical properties of a given structure; thus, facilitating the exploration of apparently close chemical space without the need of planning such syntheses *de novo*.^18,19^ The observed ring-expansion from indene 4a to naphthalene 5a belongs to this type of transformation,^12,20^ it also took place under mild conditions and remarkable yield was obtained; hence, we decided to evaluate its scope.

**Scheme 5:**
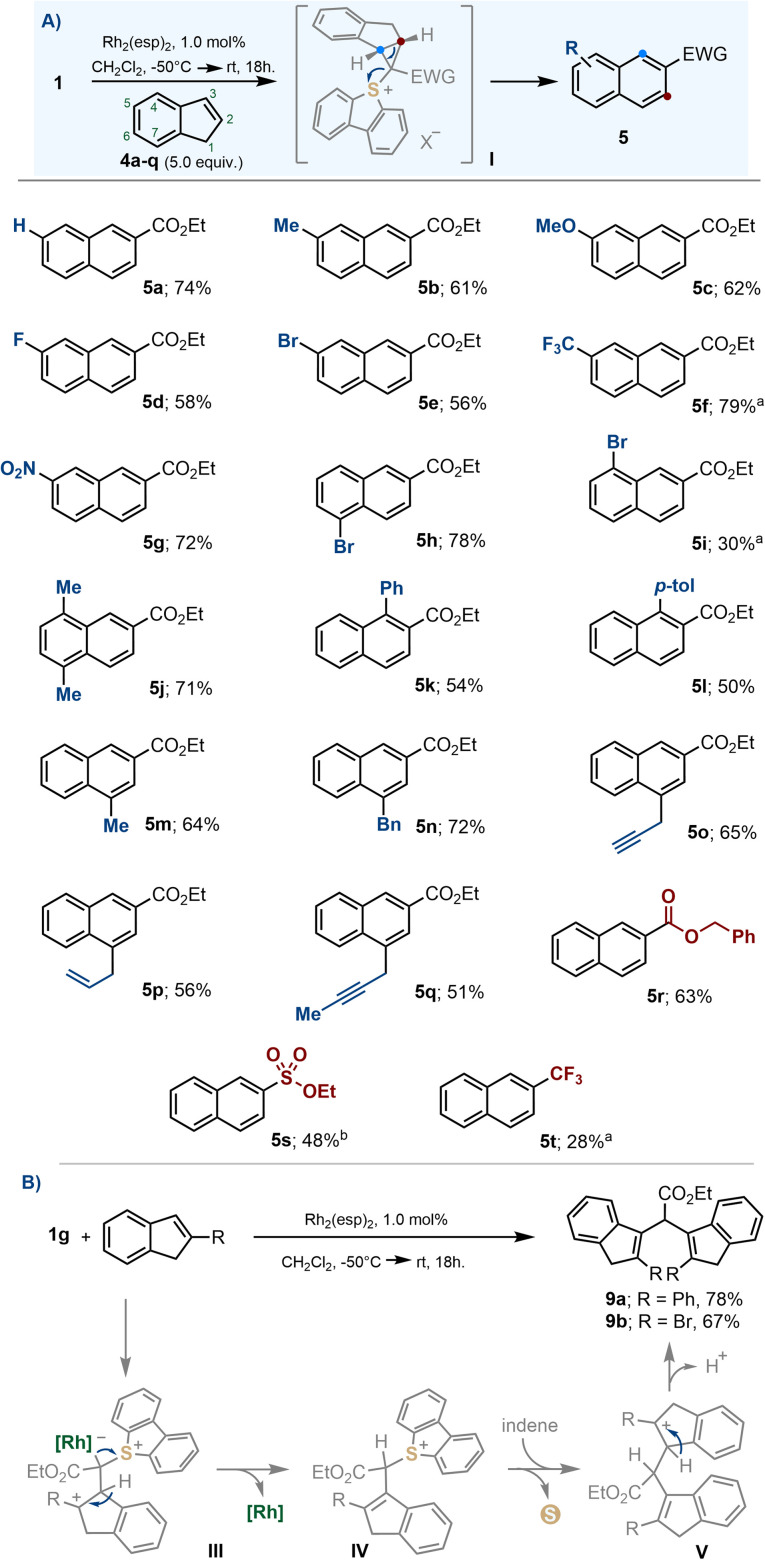
(A) Substrate scope for the ring expansion from indenes to naphthalenes; (B) dimerization of 2-indenes; ^a^ reaction heated at 80 °C for 4 days; ^b^ reaction heated at 40 °C for 12 h.

The insertion reaction is compatible with common electron donating substituents such as alkyl groups (5b, 5j, 5m and 5n) and ethers (5c), as well as electron withdrawing ones, such as halogens (5d, 5e, 5h, 5i), nitro- (5g) or trifluoromethyl moieties (5f). Likewise, exposed allyl- (5p) and propargyl substituents (5o, 5q) were tolerated (Scheme 5A). The reaction also proceeded satisfactorily for α-diazosulfonium salts 1a and 1i, allowing the incorporation of sulfonate esters and trifluoromethyl functionalities on the final naphthalene (5s and 5t, respectively). However, when the indene substrate bears substituents in the 2-position, no naphthalene is observed; instead, the dimeric structures 9 are isolated (Scheme 5B).

The formation of naphthalenes 5a–t surely involves cyclopropanation and electrocyclic ring opening, as previously reported for similar carbon-atom insertion reactions.^19*j*^ However, we believe that no cyclopropane is involved in the formation of 9. Probably, once intermediate III is formed, the regeneration of the original indene olefin is primed by deprotonation. This is followed by a protodemetallation step to deliver sulfonium salt IV, subsequent nucleophilic attack of a second equivalent of indene to form carbocation V, and final deprotonation.

**Scheme 6:**
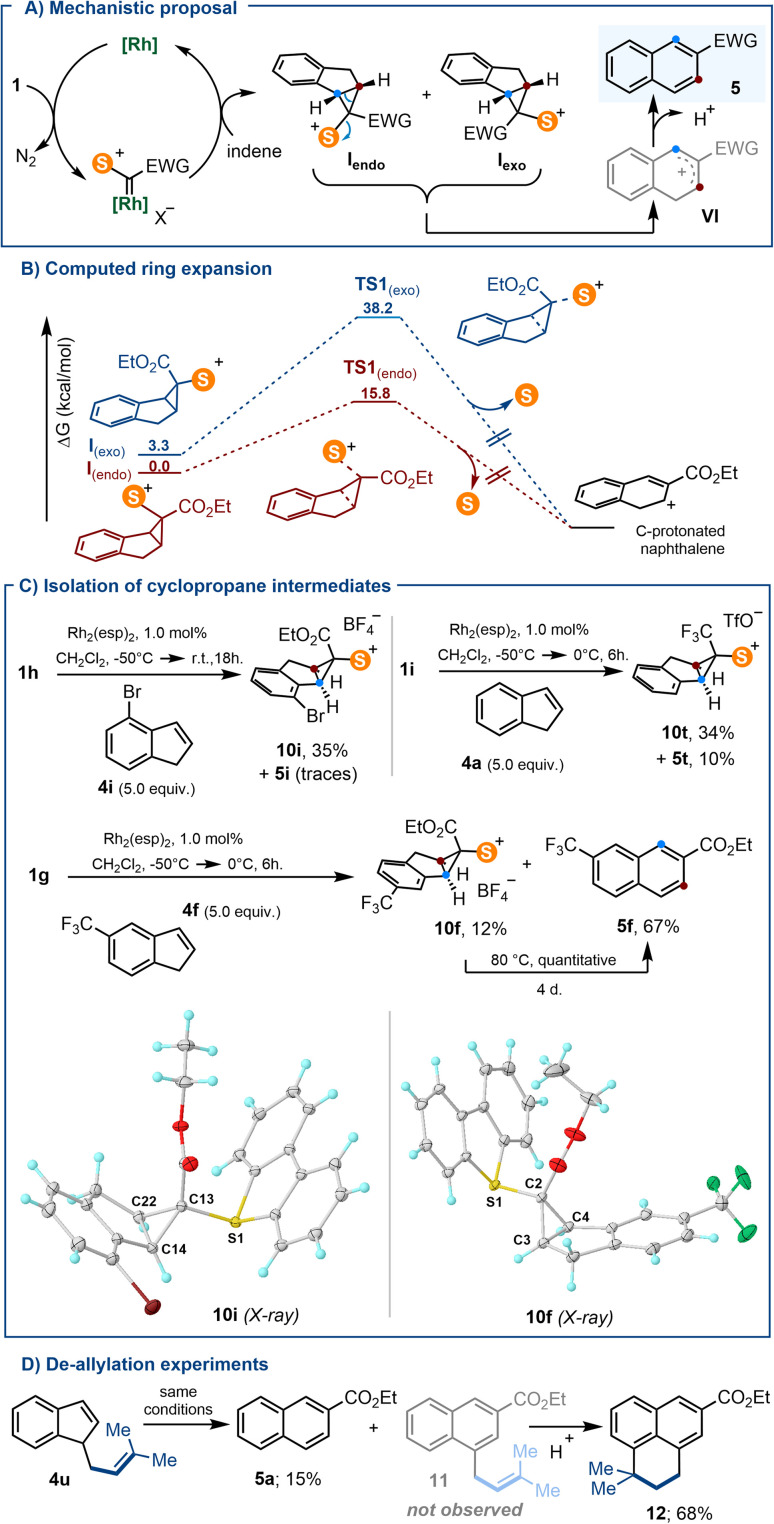
(A) Proposed reaction mechanism; (B) computed Gibbs free energy for the indene ring expansion at the uB3LYP-D3 level; (C) isolation of *exo*-substituted cyclopropyl sulfonium salts, and molecular structures in the solid state of compounds 10i and 10t. Anisotropic displacements shown at the 50% probability level. Solvent molecules and anions omitted for clarity. Selected bond lengths [Å]: 10i: S1–C13, 1.805(1); C13–C14, 1.527(2); C13–C22, 1.513(2); C14–C22, 1.521(2); 10f: S1–C2, 1.795(1); C2–C3, 1.523(2); C2–C4, 1.527(2); C3–C4, 1.517(1); (D) partial de-allylation of 4u.

Because carbenes derived from 1a–g contain two different substituents, the cyclopropanation of indenes with such species is expected to produce a mixture of diastereomeric cyclopropanes that are not likely to open at the same speed (I*_endo_* and I*_exo_*; Scheme 6A). This made us hypothesize that the geometric bias of I*_exo_* against undergoing disrotatory ring opening might facilitate its detection or even its isolation, at least for some of the substrates employed.^21^

DFT calculations at the B3LYP-D3/def2-TZVP level provide a more quantitative perspective to that hypothesis.^22^ Transition states for naphthalene formation were found from both I*_endo_* and I*_exo_*; but, the barrier for the electrocyclic ring opening through TS1*_exo_* is predicted to be significantly higher (34.8 kcal mol^−1^) than that proceeding *via*TS1*_endo_* (15.8 kcal mol^−1^) (Scheme 6B). This is accompanied by a greater degree of C–S bond breaking, and lesser degree of cyclopropane C–C bond cleavage for the unfavoured TS1*_exo_* (S–C, 2.270 Å *vs.* 2.792 Å; for TS1*_endo_* and TS1*_exo_*, respectively; C–C, 1.918 Å *vs.* 1.749 Å; for TS1*_endo_* and TS1*_exo_*, respectively). Jointly, these values justify the reluctance of I*_exo_* to ring open, and suggest that when formed, I*_exo_* should be observable.

Hence, we carefully re-checked the ^1^H NMR spectra for all crude reactions leading to the formation of 5a–t, and gratifyingly found that signals attributable to cyclopropane species were present in three cases (for 5f, 5i and 5t). These assays were subsequently repeated and submitted to careful column chromatography allowing the isolation, albeit in reduced yields, of 10i, 10f and 10t, the respective *exo*-cyclopropane salts (Scheme 6C). The connectivity of such species has been unambiguously confirmed by X-ray diffraction analysis (Scheme 6C and ESI†). It is of note that these sulfonium salts are quantitatively transformed into the corresponding naphthalenes when gently heated in acetonitrile for several days. Finally, the isolation of naphthalene 5a when indene 4u is used as the substrate further suggests the involvement of cationic intermediate VI, which evolves either *via* deprotonation, or alternatively, *via* competitive de-allylation (Scheme 6D). Compound 12 is surely formed by acid promoted cyclisation of non-observed 11.

## Conclusions

A Rh-catalysed ring expansion that enables the transformation of indenes into naphthalenes has been developed. Key for the method is the use of α-diazo sulfonium salts, which act as remarkably stable carbyne equivalents. Mechanistically, the reaction proceeds *via* initial Rh-catalysed transfer of a sulfonio-carbene unit to olefins, delivering the corresponding cyclopropanes. Subsequent electrocyclic opening of the three-membered ring with concomitant elimination of dibenzothiophene delivers the final naphthalene products. All sulfonium reagents involved can be easily handled, the conditions employed are quite mild, and the functional group tolerance is remarkable. This makes us anticipate a broad range of future applications for α-diazo sulfonium salts in the area of skeletal editing.

## Data availability

All data associated with this article are available from ESI.†

## Author contributions

S. T. and M. A. conceived and directed the project and designed the experiments. S. T. and T.-H. W. performed all of the experiments and analysed their results. C. G. carried out the crystallographic studies and calculations. S. T. and M. A. prepared the manuscript.

## Conflicts of interest

There are no conflicts to declare.

## Supplementary Material

SC-015-D4SC01138D-s001

SC-015-D4SC01138D-s002
