# Supplementary material for: Reactivity of α-diazo sulfonium salts: rhodium-catalysed ring expansion of indenes to naphthalenes
Source: Chem Sci. 2024 Mar 25;15(16):5938–43. doi: 10.1039/d4sc01138d (PMC11040645; doi:10.1039/d4sc01138d)
Supplement: SC-015-D4SC01138D-s001 [file SC-015-D4SC01138D-s001.pdf]

Electronic Supplementary Material (ESI) for Chemical Science.
This journal is © The Royal Society of Chemistry 2024

TABLE OF CONTENTS

MATERIALS AND METHODS 2
SYNTHESIS OF SULFONIUM SALTS 4
General procedure A for the synthesis of α-diazo sulfonium salts (**1a – 1j**): 4
General procedure B for the synthesis of cyclopropyl-substituted sulfonium salts (**3a-h**) 9
SYNTHESIS OF INDENE SUBSTRATES 15
General procedure C for the synthesis of 3-substituted indenes (**4k** – **4l**): 16
General procedure D for the synthesis of 1-substituted indenes (**4m** – **4q**): 17
SYNTHESIS OF NAPHTHALENES 20
General procedure E for the synthesis of naphtalenes (**5a-t**): 20
General procedure F for the synthesis of adducts (**6b** – **6e**): 31
Synthesis of **7c**: 33
Formation of bis-indenes (**9a** – **b**): 34
DIFFERENTIAL SCANNING CALORIMETRY (DSC) OF α-DIAZO SULFONIUM SALTS 35
X-RAY CRYSTALLOGRAPHIC ANALYSIS 40
SPECTROSCOPIC DATA 58
COMPUTATIONAL STUDIES 139
REFERENCES 149

MATERIALS AND METHODS

Unless stated otherwise, all reactions were carried out in pre-dried glassware under inert atmosphere (nitrogen or argon) using standard Schlenk techniques, or in a MBraun UNIlab plus glovebox. After quenching, the reaction mixtures were concentrated under reduced pressure in a rotary evaporation device at 25–40 °C. Purified compounds were further dried under high vacuum when necessary. Yields refer to spectroscopically pure compounds.

Solvents: Dry and degassed solvents (THF, dichloromethane, dichloroethane, toluene, diethyl ether, pentane, acetonitrile) were obtained from a MBraun Solvent Purification System (MB-SPS-800) or by distillation over the appropriate drying agent and stored under a protective gas atmosphere.

Chromatography: Thin layer chromatography (TLC) was performed using polygram SIL G/UV254 TLC plates from Macherey Nagel and visualized by UV irradiation and/or phosphomolybdic acid or $KMnO_4$ dip. Flash column chromatography was performed using Macherey Nagel 60 (40-63 µm) silica gel.

Starting materials: Commercially available reagents were purchased from *Acros Organics, ABCR, Alfa Aesar, BLD Pharmatech, Sigma Aldrich* and *TCI*, and used as received.

Starting materials already described were synthesized according to literature procedure: 4-methyl-N'-tosylbenzenesulfonohydrazide **S1**[1]; dibenzo[*b,d*]thiophene 5-oxide **S2**[2]; 2-bromo-1-(4-fluorophenyl)ethan-1-one **S3**[3]; ethyl diazomethanesulfonate **S4**[4]; 2-diazo-1-phenylethan-1-one **S5**[5,6]; 2-diazo-1-(*p*-tolyl)ethan-1-one **S6**[5,6]; 2-diazo-1-(4-fluorophenyl)ethan-1-one **S7**[7]; 2-diazo-1-(4-(trifluoromethyl)phenyl)ethan-1-one **S8**[8]; benzyl 2-diazoacetate **S9**[9]; 2-diazo-1,1,1-trifluoroethane **S10**[10]; 5-methylene-5*H*-dibenzo[*a*,*d*][7]annulene **S11**[11].

NMR: Spectra were recorded on Bruker Avance Neo 600, Avance Neo 400, Avance III HD 400, Avance III 400 or Avance III HD 300 spectrometers. ^{1}H and ^{13}C chemical shifts (δ) are reported in ppm relative to TMS using the solvent signals as reference in $CDCl_3$ (^{1}H: 7.26 ppm, ^{13}C: 77.16 ppm) or C_6D_6 (^{1}H: 7.16 ppm, ^{13}C: 128.1 ppm). Coupling constants (J) are given in Hertz (Hz). Data are reported as follows: s = singlet, d = doublet, t = triplet, q = quartet, m = multiplet, br = broad; coupling constants in Hz; integration.

HRMS: Spectra were recorded using *Bruker Daltonik maXis Q-TOF* (ESI), *Bruker Daltonik micrOTOF* (ESI), *Thermo Scietific LTQ Orbitrap XL* (ESI), *Thermo Scietific Exactive GC-Orbitrap-MS* (EI) or *Jeol AccuTOF* (EI) instruments. Dimensionless mass-to-charge ratios (*m*/*z*) are given.

IR: Infrared spectra were recorded on a Jasco FT/IR-4600 spectrometer and reported in wavenumbers (cm^{-1}).

Melting point: Melting points were measured with a Büchi M-560 apparatus with a heating rate of 5°C/min.

Single crystal X-ray diffraction analysis: Data collection was done on two dual source equipped *Bruker D8 Venture* four-circle-diffractometer from *Bruker AXS GmbH*. The X-ray sources used were: microfocus *IµS 2.0* Cu/Mo and microfocus *IµS 3.0* Ag/Mo from *Incoatec GmbH* with mirror optics *HELIOS* and single-hole collimator from *Bruker AXS GmbH.* The

detectors used were: *Photon III CE14* (Cu/Mo) and *Photon III HE* (Ag/Mo) from *Bruker AXS GmbH*.

APEX4 Suite (v2022.1-1) was employed for data collection together with the therein integrated programs *SAINT* V8.40A (Integration) und *SADABS* 2016/2 (Absorption correction) from *Bruker AXS GmbH*. Structure solution was done with *SHELXT*, refinement with *SHELXL*-2018/3, [12] OLEX^2,[13] and FinalCif were used for data finalization (D. Kratzert, *FinalCif*, *V113*, https://dkratzert.de/finalcif.html).

Special Utilities: *SMZ1270* stereomicroscope from *Nikon Metrology GmbH* was used for sample preparation; crystals were mounted on *MicroMounts* or *MicroLoops* from *MiTeGen* in NVH oil; for sensitive samples the *X-TEMP 2 System* was used for picking of crystals.[14]; crystals were cooled to given temperature with *Cryostream 800* from *Oxford Cryosystems.*

SYNTHESIS OF SULFONIUM SALTS

General procedure A for the synthesis of α-diazo sulfonium salts (**1a – 1j**):

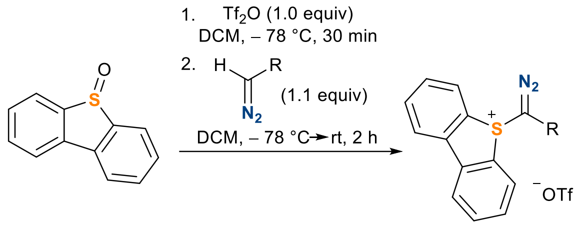

Dibenzo[*b,d*]thiophene 5-oxide **S2** (1.0 equiv) and DCM (until achieving a 0.1 M solution) were added to a Schlenk flask equipped with a magnetic stir bar. The solution was cooled to −78 °C and Tf_2O (1.0 equiv) was added dropwise at that temperature and stirred for 30 min. Afterwards, a solution of the desired diazo compound in DCM was also added dropwise and the resulting reaction mixture was stirred for 1 h at – 78 °C. Then, the cooling bath was removed and the flask was allowed to slowly reach room temperature. Solvent removal under reduced pressure afforded crude **1a-j**, which were further purified as indicated for each compound.

Synthesis of **1a**:

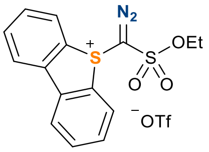

Prepared following general procedure A from dibenzo[*b,d*]thiophene 5-oxide **S2** (0.500 mg, 2.50 mmol, 1.0 equiv.), Tf_2O (0.704 g, 2.50 mmol, 0.420 ml, 1.0 equiv.) and ethyl diazomethanesulfonate **S4** (0.412 g, 2.75 mmol, 1.1 equiv.) as solution in DCM (2 mL, 1.4 M). Crude **1a** was washed with Et_2O (2 × 10 mL) and DCM (2 × 1.5 mL) obtaining a pale white solid (0.763 mg, 1.58 mmol, 63%).

Single crystals suitable for X-ray diffraction were obtained by slow diffusion of Et_2O into a solution of **1a** in MeCN.

^{1}H NMR (400 MHz, CD_3CN): δ = 8.42 (d, J = 8.4 Hz, 2H), 8.24 (d, J = 7.9 Hz, 2H), 7.95 (td, J = 7.6, 1.0 Hz, 2H), 7.80 (td, J = 7.9, 0.9 Hz, 2H), 4.10 (q, J = 7.1 Hz, 2H), 1.12 (t, J = 7.1 Hz, 3H) ppm.

^{13}C{^{1}H} NMR (101 MHz, CD_3CN) δ = 140.2, 136.4, 132.6, 129.6, 128.2, 125.4, 72.1, 14.5 ppm.

^{19}F NMR (282 MHz, CD_3CN) δ = − 79.26 ppm.

IR (ATR, neat) $\tilde{\nu}$ = 3097, 2993, 2159, 1758, 1451, 1428, 1389, 1256, 1196, 1174, 1155, 1028, 986, 926, 791, 761, 703, 622, 564, 539, 515, 481, 444, 419, 406 cm^{-1}.

HRMS-ESI (m/z) calculated for $C_{15}H_{12}N_2O_3S_2$ $[M-OTf]^+$: 333.0362; found, 333.0362.

Melting point: 126.5 °C decomp.

Synthesis of **1b**:

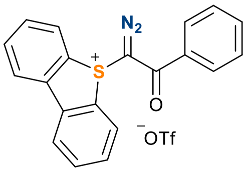

Prepared following general procedure A from dibenzo[*b,d*]thiophene 5-oxide **S2** (0.20 g, 1.0 mmol, 1.0 equiv.), Tf_2O (168 µL, 1.00 mmol, 1.00 equiv.) and 2-diazo-1-phenylethan-1-one **S5** (161 mg, 1.10 mmol, 1.1 equiv.) as solution in DCM (1 mL, 1.1 M). For purification, the reaction was quenched with water (10 mL), extracted with DCM (3 x 50 mL) and dried over anhydrous Na_2SO_4. Evaporation of the organic solvent under reduced pressure afforded a residue, which was further purified by column chromatography on silica gel using DCM/MeOH (20/1 (v/v)) as eluent. **1b** was obtained as a pale-yellow solid (164 mg, 0.35 mmol, 35%).

^{1}H NMR (400 MHz, CD_3CN): δ = 8.36 (d, J = 8.1 Hz, 2H), 8.25 (d, J = 7.8 Hz, 2H), 7.92 (t, J = 7.6 Hz, 2H), 7.77 (t, J = 7.7 Hz, 2H), 7.66 – 7.59 (m, 3H), 7.49 (t, J = 7.7 Hz, 2H) ppm.

^{13}C{^{1}H} NMR (101 MHz, CD_3CN) δ = 183.1, 140.6, 135.7, 135.2, 134.8, 132.4, 130.1, 129.2, 128.6, 125.2, 122.0 (q, J = 320.0 Hz) ppm.

^{19}F NMR (377 MHz, CD_3CN) δ = − 79.2 ppm.

IR (ATR, neat) $\tilde{\nu}$ = 2960, 2361, 2137, 1733, 1651, 1447, 1257, 1154, 1095, 1029, 798, 758, 705, 637, 517 cm^{-1}.

HRMS-ESI (m/z) calculated for $C_{20}H_{13}N_2OS$ [M-OTf]$^+$: 329.0743; found, 329.0744.

Melting point: 102 °C decomp.

Synthesis of **1c**:

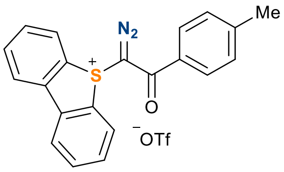

Prepared following general procedure A from dibenzo[*b,d*]thiophene 5-oxide **S2** (0.20 g, 1.00 mmol, 1.00 equiv.), Tf_2O (168 µL, 1.00 mmol, 1.00 equiv.) and 2-diazo-1-(*p*-tolyl)ethan-1-one **S6** (176 mg, 1.10 mmol, 1.1 equiv.) as solution in DCM (1 mL, 1.1 M). For purification, the reaction was quenched with water (10 mL), extracted with DCM (3 x 50 mL) and dried over anhydrous Na_2SO_4. Evaporation of the organic solvent under reduced pressure afforded a residue, which was further purified by column chromatography on silica gel eluting with DCM/MeOH (20/1 (v/v)) to afford **1c** as a pale yellow solid (207 mg, 0.42 mmol, 42%).

^{1}H NMR (400 MHz, CD_3CN): δ = 8.35 (d, J = 8.0 Hz, 2H), 8.24 (d, J = 7.8 Hz, 2H), 7.91 (t, J = 7.6 Hz, 2H), 7.76 (t, J = 7.8 Hz, 2H), 7.51 (d, J = 7.8 Hz, 2H), 7.30 (d, J = 8.0 Hz, 2H), 2.37 (s, 3H) ppm.

^{13}C{^{1}H} NMR (101 MHz, CD_3CN) δ = 182.7, 146.3, 140.5, 135.7, 132.4, 130.7, 129.2, 128.7, 128.6, 125.2, 122.0 (q, J = 320.0 Hz), 21.7. ppm.

^{19}F NMR (377 MHz, CD_3CN) δ = − 79.2 ppm.

IR (ATR, neat) $\tilde{\nu}$ = 2359, 2341, 2136, 1653, 1507, 1254, 1159, 1030, 758, 669, 637, 420 cm^{-1}.

HRMS-ESI (m/z) calculated for $C_{21}H_{15}N_2OS$ [M-OTf]$^+$: 343.0900; found, 343.0890.

Melting point: 103 °C decomp.

Synthesis of **1d**:

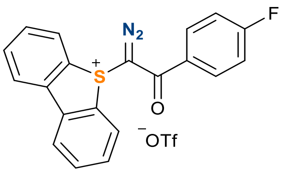

Prepared following general procedure A from dibenzo[*b,d*]thiophene 5-oxide **S2** (0.20 g, 1.00 mmol, 1.00 equiv.), Tf_2O (168 µL, 1.00 mmol, 1.00 equiv.) and 2-diazo-1-(4-fluorophenyl)ethan-1-one **S7** (181 mg, 1.10 mmol, 1.10 equiv.) as solution in DCM (1 mL, 1.1 M). For purification, the solvent was removed *in vacuo*. The mixture was further washed with dry Et_2O (3 x 10 mL) and DCM (3 x 0.5 mL). **1d** was obtained as pale yellow solid (343 mg, 0.69 mmol, 69%).

^{1}H NMR (400 MHz, CD_3CN): δ = 8.38 (d, *J* = 8.0 Hz, 2H), 8.24 (d, *J* = 7.8 Hz, 2H), 7.91 (t, *J* = 7.6 Hz, 2H), 7.76 (t, *J* = 7.8 Hz, 2H), 7.67 (dd, *J* = 8.4, 5.3 Hz, 2H), 7.21 (t, *J* = 8.5 Hz, 2H) ppm.

^{13}C{^{1}H} NMR (101 MHz, CD_3CN) δ = 181.8, 167.8, 165.2, 140.5, 135.7, 132.3, 131.7, 131.7, 131.6, 131.5, 129.2, 128.5, 125.1, 122.0 (q, *J* = 320.0 Hz), 117.3, 117.1 ppm.

^{19}F NMR (377 MHz, CD_3CN) δ = − 79.1, − 105.2 ppm.

IR (ATR, neat) $\tilde{\nu}$ = 3090, 2138, 1708, 1650, 1600, 1508, 1449, 1252, 1224, 1158, 1100, 1030, 850, 798, 758, 745, 637 cm^{-1}.

HRMS-ESI (m/z) calculated for $C_{20}H_{12}N_2OSF$ [M-OTf]$^+$: 347.0649; found, 347.0649.

Melting point: 103 °C decomp.

Synthesis of **1e**:

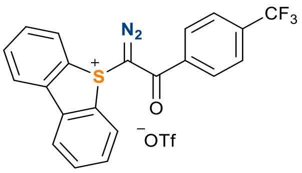

Prepared following general procedure A from dibenzo[*b,d*]thiophene 5-oxide **S2** (0.20 g, 1.00 mmol, 1.00 equiv.), Tf_2O (168 µL, 1.00 mmol, 1.00 equiv.) and 2-diazo-1-(4-(trifluoromethyl)phenyl)ethan-1-one **S8** (236 mg, 1.10 mmol, 1.10 equiv.) as solution in DCM (1 mL, 1.1 M). For purification, the solvent was removed *in vacuo*. The mixture was further washed with dry Et_2O (3 x 10 mL) and DCM (3 x 0.5 mL). **1e** was obtained as pale yellow solid (404 mg, 0.74 mmol, 74%).

^{1}H NMR (400 MHz, CD_3CN): δ = 8.40 (d, *J* = 8.1 Hz, 2H), 8.21 (d, *J* = 7.8 Hz, 2H), 7.91 (t, *J* = 7.6 Hz, 2H), 7.76 (dd, *J* = 15.4, 7.8 Hz, 4H), 7.68 (d, *J* = 8.1 Hz, 2H) ppm.

^{13}C{^{1}H} NMR (101 MHz, CD_3CN) δ = 182.3, 140.5, 138.6, 135.8, 134.7 (q, *J* = 32.7 Hz), 132.4, 129.3, 129.1, 128.4, 127.0 (q, *J* = 4.0 Hz), 125.1, 124.5 (q, *J* = 271.8 Hz), 122.0 (q, *J* = 321.0 Hz) ppm.

^{19}F NMR (377 MHz, CD_3CN) δ = − 63.8, − 79.1 ppm.

IR (ATR, neat) $\tilde{\nu}$ = 3090, 2137, 1706, 1655, 1449, 1410, 1363, 1325, 1251, 1222, 1157, 1130, 1064, 1027, 865, 840, 756, 704, 682, 636, 612, 573, 515, 473, 455, 425 cm^{-1}.

HRMS-ESI (m/z) calculated for $C_{21}H_{12}F_3N_2OS$ $[M\text{-}OTf]^+$: 397.0617; found, 397.0607.

Melting point: 110 °C decomp.

Synthesis of **1f**:

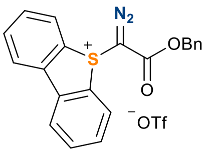

Prepared following general procedure A from dibenzo[*b,d*]thiophene 5-oxide **S2** (1.00 g, 5.0 mmol, 1.0 equiv.), Tf_2O (1.41 g, 5.0 mmol, 0.841 ml, 1.0 equiv.) and benzyl 2-diazoacetate **S9** (1.06 g, 6.00 mmol, 1.1 equiv.) as solution in DCM (3 mL, 2 M). The residue was washed with Et_2O (2 × 10 mL) and DCM (2 × 2 mL). **1f** was obtained as pale white solid (1.44 g, 2.83 mmol, 57%).

^{1}H NMR (400 MHz, CD_3CN): δ = 8.26 (dq, *J* = 8.1, 0.5 Hz, 2 H), 8.02 (d, *J* = 7.7 Hz, 2 H), 7.84 (td, *J* = 7.6, 1.2 Hz, 2 H), 7.72 (td, *J* = 7.8, 1.3 Hz, 2 H), 7.39 – 7.23 (m, 3 H), 6.88 (d, *J* = 7.2 Hz, 2 H), 4.80 (s, 2 H).

^{13}C{^{1}H} NMR (101 MHz, CD_3CN) δ = 159.1, 140.0, 135.5, 134.9, 132.2, 129.7, 129.5, 129.5, 129.4, 128.8, 125.0, 122.2 (q, *J* = 320.0 Hz), 69.8 ppm.

^{19}F NMR (377 MHz, CD_3CN) δ = − 79.17 ppm.

IR (ATR, neat) $\tilde{\nu}$ = 3094, 2997, 2157, 1769, 1710, 1450, 1427, 1375, 1253, 1222, 1152, 1081, 1048, 1028, 941, 906, 753, 735, 700, 634, 572, 531, 514, 472, 434, 418 cm^{-1}.

HRMS-ESI (m/z) calculated for $C_{21}H_{15}N_2O_2S$ $[M\text{-}OTf]^+$: 359.0849; found, 359.0847.

Melting point: 145 °C decomp.

Synthesis of **1g**:

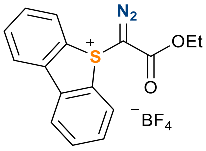

To a solution of α-diazo sulfonium triflate **1h** (5.00 g, 11.2 mmol) in MeCN (60 mL) a sat. aq. $NaBF_4$ solution (30 mL) was added, and the mixture stirred for 10 min. Afterwards, DCM (50 mL) and the aqueous phase additionally extracted with DCM (3 × 50 mL). The combined organic phases were dried over Na_2SO_4 and concentrated. The residue thus obtained was purified by chromatography on silica gel eluting with DCM/acetone (8/2 (v/v)) to afford the product as pale-yellow solid (3.62 g, 9.42 mmol, 84%).

Single crystals suitable for X-ray diffraction were obtained by slow diffusion of Et2O into a solution of **1g** (BF_4^-) in MeCN.

^{1}H NMR (400 MHz, CD_3CN): δ = 8.30 (d, *J* = 8.1 Hz, 2H), 8.23 (d, *J* = 7.7 Hz, 2H), 7.90 (t, *J* = 7.6 Hz, 2H), 7.75 (t, *J* = 7.7 Hz, 2H), 3.89 (q, *J* = 7.1 Hz, 2H), 0.85 (t, *J* = 7.2 Hz, 3H) ppm.

^{13}C{^{1}H} NMR (101 MHz, CD_3CN) δ = 159.0, 140.1, 135.5, 132.2, 129.4, 128.9, 124.9, 118.3, 64.3, 13.8 ppm.

^{19}F NMR (282 MHz, CD_3CN) δ = − 151.46 ppm.

IR (ATR, neat) $\tilde{\nu}$ = 3107, 2155, 1715, 1577, 1484, 1467, 1450, 1428, 1393, 1361, 1275, 1191, 1167, 1007, 955, 883, 867, 755, 728, 704, 612, 545, 521, 471, 445, 421, 411 cm^{-1}.

HRMS-ESI (m/z) calculated for $C_{16}H_{12}N_2O_2S$ $[M-BF_4]^+$: 297.0692; found, 297.0692.

Melting point: 140 °C decomp.

Synthesis of **1h**:

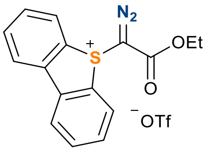

Prepared following general procedure A from dibenzo[*b,d*]thiophene 5-oxide **S2** (4.20 g, 21.0 mmol, 1.0 equiv.), Tf_2O (5.92 g, 21.0 mmol, 3.53 ml, 1.0 equiv.) and ethyl 2-diazoacetate as solution in DCM (wt = 13 %, 2.80 mL, 23.1 mmol, 1.1 equiv.). The desired compound was washed with Et_2O (2 × 20 mL) and DCM (2 × 5 mL). **1h** was obtained as pale white solid (6.62 g, 14.8 mmol, 71%).

Single crystals suitable for X-ray diffraction were obtained by slow diffusion of Et_2O into a solution of **1h** in MeCN.

^{1}H NMR (400 MHz, CD_3CN): δ = 8.32 (d, J = 8.1 Hz, 2H), 8.22 (d, J = 7.8 Hz, 2H), 7.90 (t, J = 7.6 Hz, 2H), 7.74 (t, J = 7.8 Hz, 2H), 3.89 (q, J = 7.1 Hz, 2H), 0.85 (t, J = 7.1 Hz, 3H) ppm.

^{13}C{^{1}H} NMR (101 MHz, CD_3CN) δ = 159.0, 140.1, 135.5, 132.2, 129.5, 128.9, 124.8, 122.0 (q, J = 318.5 Hz), 64.3, 13.8 ppm.

^{19}F NMR (377 MHz, CD_3CN) δ = − 79.17 ppm.

The spectral data were in accordance with those reported in literature.[15]

Synthesis of **1i**:

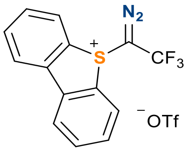

Prepared following general procedure A from dibenzo[*b,d*]thiophene 5-oxide **S2** (2.00 g, 10.0 mmol, 1.0 equiv.), Tf_2O (2.82 g, 10.0 mmol, 1.68 ml, 1.0 equiv) and 2-diazo-1,1,1-trifluoroethane **S10** as solution in DCM (0.366 M, 33.0 mL, 12.0 mmol, 1.1 equiv.). The desired compound was washed with Et_2O (2 × 20 mL) and DCM (2 × 5 mL). **1i** was obtained as pale white solid (2.88 g, 6.13 mmol, 61%).

^{1}H NMR (300 MHz, CD_3CN): δ = 8.42 (d, J = 8.1 Hz, 2H), 8.26 (dd, J = 7.8, 1.2 Hz, 2H), 7.96 (td, J = 7.7, 1.1 Hz, 2H), 7.81 (td, J = 7.8, 1.2 Hz, 2H) ppm.

^{13}C{^{1}H} NMR (101 MHz, CD_3CN) δ = 139.3, 136.3, 132.7, 129.5, 128.7, 125.5, 123.0 (q, J = 272.1 Hz), 122.0 (q, J = 321.5 Hz) ppm.

^{19}F NMR (377 MHz, CD_3CN) δ = −55.3, −79.3 ppm.

The spectral data were in accordance with those reported in literature.[15]

Synthesis of **1j**:

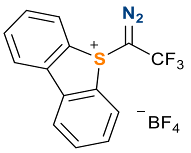

To a solution of α-diazo sulfonium triflate **1i** (0.70 g, 1.58 mmol, 1.0 equiv.) in MeCN (20 mL) a sat. aq. $NaBF_4$ solution (5 mL) was added and the mixture stirred for 10 min. Afterwards mixture was diluted with DCM (30 mL) and the aqueous phase further extracted with DCM (3 × 30 mL). The combined organic phases were dried over Na_2SO_4 and concentrated. The residue was purified by column chromatography on silica gel eluting with DCM/acetone (8/2 (v/v)) to afford **1j** as pale yellow solid (0.46 g, 1.22 mmol, 77%).

^{1}H NMR (400 MHz, CD_3CN): δ = 8.40 (d, J = 8.1 Hz, 2H), 8.26 (dd, J = 7.8, 1.2 Hz, 2H), 7.96 (td, J = 7.6, 1.1 Hz, 2H), 7.82 (td, J = 7.8, 1.2 Hz, 2H) ppm.

^{13}C{^{1}H} NMR (101 MHz, CD_3CN) δ = 139.3, 136.3, 132.7, 129.5, 128.6, 125.5, 123.0 (q, J = 273.8 Hz) ppm.

^{19}F NMR (282 MHz, CD_3CN) δ = −55.3, −151.6 ppm.

IR (ATR, neat) $\tilde{\nu}$ = 3093, 2153, 1841, 1576, 1485, 1465, 1452, 1427, 1300, 1213, 1168, 1144, 958, 782, 740, 706, 695, 611, 589, 579, 548, 521, 509, 475, 465 cm^{-1}.

HRMS-ESI (m/z) calculated for $C_{14}H_8N_2O_2S$ $[M-BF_4]^+$: 293.0355; found, 293.0357.

Melting point: 150 °C decomp.

General procedure B for the synthesis of cyclopropyl-substituted sulfonium salts (**3a-h**)

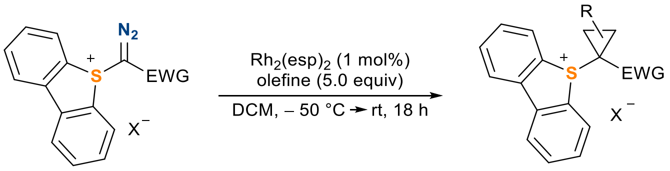

The desired α-diazo sulfonium salt **1** (0.2 mmol, 1.0 equiv.) and $Rh_2(esp)_2$ (1.5 mg, 2.0 µmol, 1 mol%) were added as solids to a Schlenk flask equipped with a magnetic stir bar. The flask was then cooled to – 50 °C and DCM (2.8 mL) was slowly added. Finally, the stirring was started and the desired olefine was added as a solution in DCM (0.2 mL). The resulting mixture was allowed to reach room temperature slowly overnight. Analytically pure materials were obtained following the method indicated for each product.

Synthesis of **3a**:

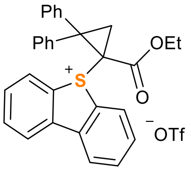

Prepared following general procedure B from α-diazo sulfonium salt **1g** (89.1 mg, 0.20 mmol, 1.0 equiv.) and 1,1-diphenylethylene (181 mg, 177 µL, 1.0 mmol 5.0 equiv.). Once the starting materials were consumed, the reaction mixture was diluted with DCM (5 mL), washed with water (5 mL) and the aq. phase was extracted further with DCM (3 × 20 mL). The combined organic phase was dried over Na_2SO_4, filtered, and the solvent removed under reduced

pressure. The residue thus obtained was purified by chromatography on silica gel eluting with DCM/MeOH (100/0→95/5 (v/v)) to afford **3a** as colorless solid (91.7 mg, 153.2 µmol, 77%).

Single crystals suitable for X-ray diffraction were obtained by slow diffusion of Et_2O into a solution of **3a** in MeCN.

^{1}H NMR (400 MHz, CD_3CN): *δ* = 8.44 (dd, *J* = 8.1, 1.2 Hz, 1H), 8.03 – 7.87 (m, 4H), 7.83 (t, *J* = 7.5 Hz, 2H), 7.75 (td, *J* = 7.7, 1.3 Hz, 1H), 7.37 – 7.25 (m, 6H), 7.22 – 7.15 (m, 1H), 7.10 – 7.00 (m, 5H), 3.85 (dq, *J* = 10.7, 7.1 Hz, 1H), 3.67 (dq, *J* = 10.7, 7.1 Hz, 1H), 3.37 (d, *J* = 8.2 Hz, 1H), 3.03 (d, *J* = 8.0 Hz, 1H), 0.71 (t, *J* = 7.1 Hz, 3H) ppm.

^{13}C{^{1}H} NMR (101 MHz, CD_3CN) δ = 165.5, 141.0, 141.0, 140.2, 135.3, 135.3, 134.3, 132.5, 132.1, 130.0, 130.0, 129.7, 129.5, 129.4, 129.4, 129.2, 128.9, 128.1, 128.0, 127.7, 125.6, 125.3, 125.2, 123.7, 122.7, 122.1 (q, *J* = 321.0 Hz), 64.9, 51.7, 50.5, 25.8, 13.4 ppm.

^{19}F NMR (377 MHz, CD_3CN) δ = −79.3 ppm.

IR (ATR, neat) $\tilde{\nu}$ = 3086, 2994, 2361, 1770, 1758, 1732, 1683, 1576, 1495, 1447, 1368, 1272, 1242, 1148, 1098, 1060, 1027, 968, 893, 851, 796, 754, 703, 633, 571, 544, 516, 498, 482, 457, 446, 418 cm^{-1}.

HRMS-ESI (m/z) calculated for $C_{30}H_{25}O_2S$ $[M\text{-}OTf]^+$: 449.1570; found, 449.1579.

Melting point: 136°C decomp.

Synthesis of **3b**:

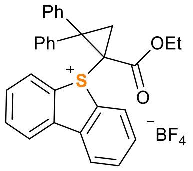

Prepared following general procedure B from α-diazo sulfonium salt **1g** (77.1 mg, 0.20 mmol, 1.0 equiv.) and 1,1-diphenylethylene (181 mg, 177 µL, 1.0 mmol 5.0 equiv.). Once the starting materials were consumed, the reaction mixture was diluted with DCM (5 mL), washed with water (5 mL) and the aq. phase was further extracted with DCM (3 × 20 mL). The combined organic phase was dried over Na_2SO_4, filtered, and the solvent removed under reduced pressure. The residue thus obtained was purified by chromatography on silica gel eluting with DCM/MeOH (100/0→95/5 (v/v)) to afford **3b** as colorless solid (87.4 mg, 162.9 µmol, 81%).

Single crystals suitable for X-ray diffraction were obtained by slow diffusion of Et_2O into a solution of **3b** in MeCN.

^{1}H NMR (400 MHz, CD_3CN): *δ* = 8.43 (dd, *J* = 8.3, 1.0 Hz, 1H), 8.01 – 7.87 (m, 4H), 7.83 (td, *J* = 7.7, 1.3 Hz, 2H), 7.75 (td, *J* = 7.7, 1.3 Hz, 1H), 7.35 – 7.26 (m, 5H), 7.21 – 7.17 (m, 1H), 7.08 – 7.05 (m, 4H), 3.85 (dq, *J* = 10.8, 7.1 Hz, 1H), 3.66 (dq, *J* = 10.8, 7.2 Hz, 1H), 3.37 (d, *J* = 8.0 Hz, 1H), 3.04 (d, *J* = 8.1 Hz, 1H), 0.71 (t, *J* = 7.2 Hz, 3H) ppm.

^{13}C{^{1}H} NMR (101 MHz, CD_3CN) δ = 165.5, 141.0, 141.0, 140.2, 135.3, 135.3, 134.3, 132.5, 132.1, 130.0, 130.0, 129.7, 129.6, 129.4, 129.3, 129.2, 128.8, 128.0, 127.7, 125.3, 125.2, 64.9, 51.7, 50.4, 25.7, 13.4 ppm.

^{19}F NMR (377 MHz, CD_3CN) δ = −151.6 ppm.

IR (ATR, neat) $\tilde{\nu}$ = 3092, 2979, 1732, 1671, 1494, 1448, 1432, 1365, 1300, 1244, 1166, 1048, 1021, 969, 951, 889, 873, 844, 824, 794, 777, 753, 737, 700, 678, 609, 596, 547, 520, 499, 474, 454, 434, 414 cm^{-1}.

HRMS-ESI (m/z) calculated for $C_{30}H_{25}O_2S$ [M-BF_4]$^+$: 449.1570; found, 449.1572.

Melting point: 148 °C decomp.

Synthesis of **3c**:

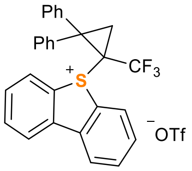

Prepared following general procedure B from α-diazo sulfonium salt **1i** (88.5 mg, 0.20 mmol, 1.0 equiv.) and 1,1-diphenylethylene (181 mg, 177 µL, 1.0 mmol 5.0 equiv.). Once the starting materials were consumed, the reaction mixture was diluted with DCM (5 mL), washed with water (5 mL) and the aq. phase was additionally extracted with DCM (3 × 20 mL). The combined organic phase was dried over Na_2SO_4, filtered, and the solvent removed under reduced pressure. The residue was purified by column chromatography on silica gel eluting with DCM/MeOH (100/0→95/5 (v/v)) to afford **3c** as colorless solid (95.7 mg, 161.0 µmol, 80%).

Single crystals suitable for X-ray diffraction were obtained by slow diffusion of Et_2O into a solution of **3c** in MeCN.

^{1}H NMR (400 MHz, CD_3CN): δ = 8.55 (d, J = 8.1, 1H), 8.36 – 8.23 (m, 3H), 8.03 (td, J = 7.7, 1.1 Hz, 1H), 7.99 – 7.89 (m, 4H), 7.81 (td, J = 7.8, 1.3 Hz, 1H), 7.70 (d, J = 7.8 Hz, 1H), 7.57 (t, J = 7.7 Hz, 2H), 7.49 – 7.30 (m, 4H), 3.63 (dq, J = 8.9, 1.8 Hz, 1H), 3.04 (d, J = 8.9 Hz, 1H) ppm.

^{13}C{^{1}H} NMR (101 MHz, CD_3CN) δ = 141.0, 140.6, 138.5, 137.7, 136.5, 136.0, 132.7, 132.5, 131.1, 130.5, 130.2, 130.1, 130.1, 129.8, 129.7, 129.0, 128.4, 125.9, 125.8, 125.6, 123.5 (q, J = 280.0 Hz), 122.1 (q, J = 321.0 Hz), 49.9 (q, J = 33.9 Hz), 48.3, 22.0 ppm.

^{19}F NMR (377 MHz, CD_3CN) δ = −57.8, −79.2 ppm.

IR (ATR, neat) $\tilde{\nu}$ = 2996, 1770, 1754, 1495, 1449, 1248, 1187, 1150, 1093, 1027, 754, 708, 658, 635, 571, 542, 514, 497, 421 cm^{-1}.

HRMS-ESI (m/z) calculated for $C_{28}H_{20}F_3S$ [M-OTf]$^+$: 445.1232; found, 445.1241.

Melting point: 148 °C decomp.

Synthesis of **3d**:

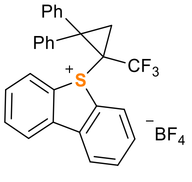

Prepared following general procedure B from α-diazo sulfonium salt **1j** (76.1 mg, 0.20 mmol 1.0 equiv.) and 1,1-diphenylethylene (180 mg, 177 µL, 1.0 mmol, 5.0 equiv.). Once the starting materials were consumed, the reaction mixture was diluted with DCM (5 mL), washed with water (5 mL) and the aq. phase was further extracted with DCM (3 × 20 mL). The combined organic phase was dried over Na_2SO_4, filtered, and the solvent removed under reduced

pressure. The residue thus obtained was purified by chromatography on silica gel eluting with DCM/MeOH (100/0→95/5 (v/v)) to afford **3d** as colorless solid (88.1 mg, 165.5 µmol, 83%).

Single crystals suitable for X-ray diffraction were obtained by slow diffusion of Et_2O into a solution of **3d** in MeCN.

^{1}H NMR (400 MHz, CD_3CN): *δ* = 8.55 (d, *J* = 8.1 Hz, 1H), 8.32 – 8.25 (m, 3H), 8.04 (td, *J* = 7.7, 1.1 Hz, 1H), 8.00 – 7.90 (m, 4H), 7.81 (td, *J* = 7.9, 1.3 Hz, 1H), 7.70 (d, *J* = 7.3 Hz, 2H), 7.57 (t, *J* = 7.8 Hz, 2H), 7.50 – 7.31 (m, 4H), 3.60 (dq, *J* = 8.8, 1.8 Hz, 1H), 3.05 (d, *J* = 8.9 Hz, 1H) ppm.

^{13}C{^{1}H} NMR (101 MHz, CD_3CN) δ = 141.0, 140.6, 138.4, 137.7, 136.5, 136.0, 132.7, 132.5, 131.0, 130.5, 130.2, 130.1, 130.1, 129.8, 129.7, 128.9, 128.4, 125.9, 125.8, 125.6, 123.5 (q, *J* = 280.0 Hz), 49.9 (q, *J* = 34.3 Hz), 48.3, 22.0 ppm.

^{19}F NMR (377 MHz, CD_3CN) δ = −57.8, −151.7 ppm.

IR (ATR, neat) $\tilde{\nu}$ = 3099, 3012, 1496, 1449, 1423, 1336, 1320, 1276, 1245, 1197, 1156, 1132, 997, 955, 923, 899, 874, 853, 788, 739, 727, 698, 658, 604, 543, 518, 498, 488, 470, 437, 417 cm^{-1}.

HRMS-ESI (m/z) calculated for $C_{28}H_{20}F_3S$ $[M-BF_4]^+$: 445.1232; found, 445.1247.

Melting point: 158 °C decomp.

Synthesis of **3e**:

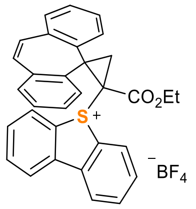

Prepared following general procedure B from α-diazo sulfonium salt **1g** (76.9 mg, 0.20 mmol 1.0 equiv.) and 5-methylene-5*H*-dibenzo[*a*,*d*][7]annulene **S11** (204.5 mg, 1.000 mmol, 5.0 equiv.). Once the starting materials were consumed, the reaction mixture was diluted with DCM (5 mL), washed with water (5 mL), and the aq. phase was further extracted with DCM (3 × 20 mL). The combined organic phase was dried over Na_2SO_4, filtered, and the solvent removed under reduced pressure. The residue was purified by chromatography on silica gel eluting with DCM/MeOH (100/0→95/5 (v/v)) to afford **3e** as colorless solid (95.7 mg, 170.8 µmol, 85%).

Single crystals suitable for X-ray diffraction were obtained by slow diffusion of Et_2O into a solution of **3d** in MeCN.

^{1}H NMR (400 MHz, CD_3CN): *δ* = 8.23 (d, *J* = 8.1 Hz, 1H), 8.00 (dd, *J* = 5.8, 3.3 Hz, 1H), 7.92 (d, *J* = 3.6, 2H), 7.84–7.73 (m, 4H), 7.49 (d, *J* = 7.7 Hz, 1H), 7.39 (dt, *J* = 6.9, 2.6 Hz, 1H), 7.35 – 7.25 (m, 2H), 7.17 (ddd, *J* = 8.2, 6.2, 2.3 Hz, 1H), 7.05 – 6.83 (m, 4H), 6.27 (d, *J* = 11.8 Hz, 1H), 3.78 (dq, *J* = 10.7, 7.1 Hz, 1H), 3.57 (dq, *J* = 10.7, 7.1 Hz, 1H), 3.37 (d, *J* = 8.6 Hz, 1H), 3.29 (d, *J* = 8.6 Hz, 1H), 0.71 (t, *J* = 7.1 Hz, 3H) ppm.

^{13}C{^{1}H} NMR (101 MHz, CD_3CN) δ = 165.9, 141.3, 140.5, 137.2, 136.3, 135.5, 135.1, 133.8, 132.6, 132.2, 131.7, 131.2, 131.0, 130.4, 130.3, 130.2, 129.9, 129.3, 129.1, 129.0, 128.6, 127.9, 127.2, 126.6, 125.3, 124.8, 64.8, 51.6, 49.5, 25.0, 13.4 ppm.

^{19}F NMR (377 MHz, CD_3CN) δ = −151.7 ppm.

IR (ATR, neat) $\tilde{\nu}$ = 2984, 2357, 1768, 1747, 1731, 1698, 1558, 1540, 1488, 1448, 1371, 1319, 1243, 1165, 1049, 883, 853, 809, 795, 754, 702, 669, 637, 612, 565, 518, 502, 469, 438, 417 cm^{-1}.

HRMS-ESI (m/z) calculated for $C_{32}H_{25}O_2S$ [M-BF_4]$^+$: 473.1570; found, 473.1583.

Melting point: 128 °C

Synthesis of **3f**:

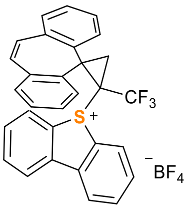

Prepared following general procedure B from α-diazo sulfonium salt **1j** (76.2 mg, 0.20 mmol, 1.0 equiv.) and 5-methylene-5*H*-dibenzo[*a*,*d*][7]annulene **S11** (204.2 mg, 1.00 mmol, 5.0 equiv.). Once the starting materials were consumed, the reaction mixture was diluted with DCM (5 mL), washed with water (5 mL) and the aq. phase was further extracted with DCM (3 × 20 mL). The combined organic phase was then dried over Na_2SO_4, filtered, and the solvent removed under reduced pressure. The residue thus obtained was purified by chromatography on silica gel eluting with DCM/MeOH (100/0→95/5 (v/v)) to deliver **3f** as colorless solid (95.1 mg, 170.9 mmol, 85%).

Single crystals suitable for X-ray diffraction were obtained by slow diffusion of Et_2O into a solution of **3d** in MeCN.

^{1}H NMR (300 MHz, CD_3CN): δ = 8.32 (d, *J* = 8.1 Hz, 1H), 8.25 (d, *J* = 8.2 Hz, 1H), 8.19 (d, *J* = 7.8 Hz, 2H), 7.95 (dt, *J* = 7.7, 3.4 Hz, 2H), 7.89 – 7.76 (m, 3H), 7.69 – 7.38 (m, 9H), 3.63 (dq, *J* = 9.7, 1.9 Hz, 1H), 3.33 (d, *J* = 9.5 Hz, 1H) ppm.

^{13}C{^{1}H} NMR (101 MHz, CD_3CN) δ = 140.2, 139.6, 136.0, 135.5, 135.2, 135.0, 133.2, 132.6, 131.6, 131.3, 131.0, 130.2, 130.2, 129.8, 129.7, 129.6, 129.5, 128.8, 128.2, 128.1, 128.0, 127.4, 124.6, 124.6, 124.2, 121.8 (q, *J* = 280.0 Hz), 48.8 (q, *J* = 34.0 Hz), 45.4, 20.2 ppm.

^{19}F NMR (377 MHz, CD_3CN) δ = −59.0, −151.7 ppm.

IR (ATR, neat) $\tilde{\nu}$ = 2994, 2360, 2340, 1770, 1758, 1558, 1488, 1448, 1435, 1374, 1329, 1243, 1188, 1161, 1051, 893, 812, 755, 736, 703, 652, 612, 559, 519, 499, 466, 447, 418, 403 cm^{-1}.

HRMS-ESI (m/z) calculated for $C_{30}H_{20}F_3S$ [M-BF_4]$^+$: 469.1232; found, 469.1247.

Melting point: 166 °C decomp.

Synthesis of **3g**:

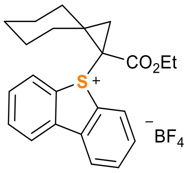

Prepared following general procedure B from α-diazo sulfonium salt **1g** (76.9 mg, 0.20 mmol,1.0 equiv) and methylenecyclohexane (96.3 mg, 120.5 µL, 1.00 mmol, 5.0 equiv). Once the starting materials were consumed, the desired product was crystallized from the crude reaction mixture by layering with Et_2O. Compound **3g** was obtained as colorless crystals (48.7mg, 107.7 mmol, 54%).

^{1}H NMR (400 MHz, $CDCl_3$): δ = 8.26 (d, J = 8.1 Hz, 1H), 8.15 (d, J = 7.8 Hz, 1H), 8.07 (d, J = 7.8 Hz, 1H), 7.96 (d, J = 8.0 Hz, 1H), 7.83 (t, J = 7.6 Hz, 1H), 7.72 (dt, J = 15.5, 7.6 Hz, 2H), 7.59 (t, J = 7.7 Hz, 1H), 3.66 – 3.34 (m, 2H), 2.84 (d, J = 6.9 Hz, 1H), 2.33 – 2.11 (m, 3H), 2.01 – 1.97 (m, 1H), 1.86 – 1.56 (m, 4H), 1.47 – 1.37 (m, 2H), 1.28 – 1.19 (m, 1H), 0.55 (t, J = 7.1 Hz, 3H) ppm.

^{13}C{^{1}H} NMR (101 MHz, $CDCl_3$) δ = 162.2, 139.8, 139.5, 134.5, 134.5, 131.5, 131.2, 129.0, 128.7, 127.7, 127.0, 124.4, 123.9, 63.0, 49.2, 39.2, 34.5, 31.5, 26.3, 25.6, 25.4, 13.3 ppm.

^{19}F NMR (377 MHz, $CDCl_3$) δ = −151.1 ppm.

IR (ATR, neat) $\tilde{\nu}$ = 2994, 1770, 1758, 1731, 1668, 1447, 1369, 1307, 1244, 1211, 1161, 1046, 965, 854, 767, 753, 705, 614, 521, 510, 479, 446, 421, 403 cm^{-1}.

HRMS-ESI (m/z) calculated for $C_{23}H_{25}O_2S$ $[M\text{-}BF_4]^+$: 365.1570; found, 365.1574.

Melting point: 108 °C decomp.

Synthesis of **3h**:

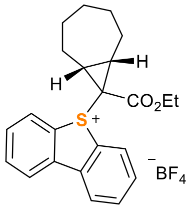

Prepared following general procedure B from α-diazo sulfonium salt **1g** (77.2 mg, 0.20 mmol 1.0 equiv) and cycloheptene (96.4 mg, 117 µL, 1.00 mmol, 5.0 equiv). Once the starting materials were consumed, the reaction mixture was concentrated under reduced pressure and Et_2O (5 mL) was added to induce precipitation. The precipitate was washed with Et_2O (2 × 1 mL) and dried under high vacuum to afford the desired compound as light brown solid (30.1 mg, 66.6 µmol, 33%).

^{1}H-NMR (400 MHz, $CDCl_3$): δ = 8.18 (d, J = 7.8 Hz, 2H), 7.92 (d, J = 8.0 Hz, 2H), 7.83 (t, J = 7.6 Hz, 2H), 7.71 (t, J = 7.7 Hz, 2H), 3.61 (q, J = 7.1 Hz, 2H), 2.88 – 2.62 (m, 4H), 2.32 – 1.98 (m, 5H), 1.64 – 1.54 (m, 3H), 0.61 (t, J = 7.1 Hz, 3H) ppm.

^{13}C{^{1}H}-NMR (101 MHz, $CDCl_3$) δ = 164.2, 140.3, 134.3, 131.5, 127.0, 124.3, 63.3, 48.4, 37.3, 31.2, 28.0, 27.0, 13.2 ppm.

^{19}F NMR (377 MHz, $CDCl_3$) δ = − 152.8 ppm.

IR (ATR, neat) $\tilde{\nu}$ = 2926, 2851, 1734, 1575, 1464, 1447, 1391, 1363, 1273, 1257, 1224, 1140, 1096, 912, 862, 842, 804, 774, 700, 612, 561, 519, 508, 485, 475, 435, 422 cm^{-1}.

HRMS-ESI (m/z) calculated for $C_{23}H_{25}O_2S$ $[M\text{-}BF_4]^+$: 365.1570; found, 365.1579.

Melting point: 134 °C.

SYNTHESIS OF INDENE SUBSTRATES

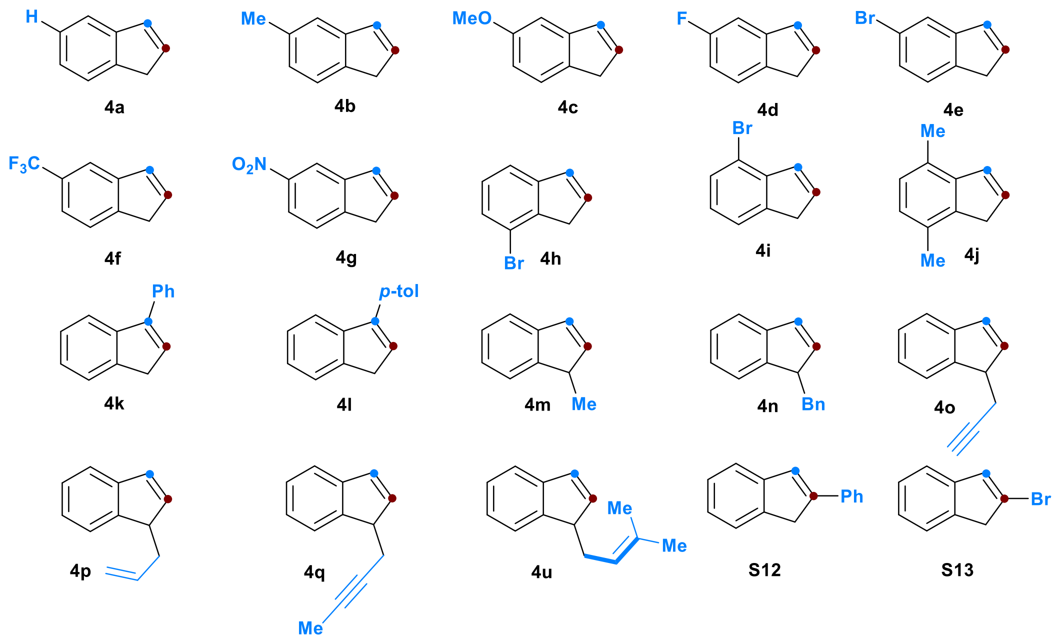

Substrates **4a**, **4h**, **4j** and **S13** were commercially available (BLD Pharm, Acros, ABCR, Alfa Aesar, Sigma Aldrich, Fluorochem, TCI) and were used as received unless stated otherwise.

Substrates **4b**[16], **4c**[16], **4d**[16], **4e**[17], **4g**[18], **4i**[19], **S12**[18] were prepared according to literature procedure.

Synthesis of **4f**:

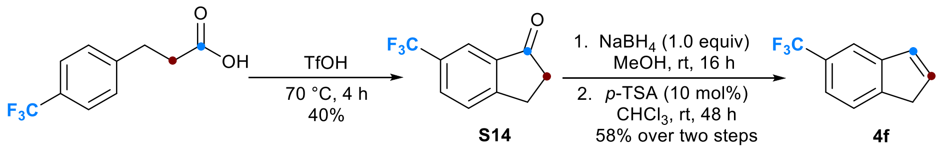

This protocol modifies a literature known procedure.[20] To a Schlenk flask equipped with a magnetic stir bar 3-(4-(trifluoromethyl)phenyl)propanoic acid (2.85 g, 13.0 mmol, 1.0 equiv.) and TfOH (5 mL) were added. The resulting mixture was heated to 70 °C for 4 h, after this allowed to reach room temperature, and finally it was quenched with H_2O at 0 °C. The mixture thus obtained was transferred to a separatory funnel and extracted with DCM (3 × 50 mL). The combined organic layers were washed with brine and finally dried over Na_2SO_4, filtered, and the solvent was removed under reduced pressure. The residue thus obtained was purified by chromatography on silica gel eluting with hexane/EtOAc (100/0→6/1 (v/v)) to afford indanone **S14** as a colorless oil (1.05 g, 5.25 mmol, 40%).

^{1}H-NMR (300 MHz, $CDCl_3$): δ = 8.02 (s, 1H), 7.83 (d, J = 8.0 Hz, 1H), 7.62 (d, J = 8.0 Hz, 1H), 3.22 (t, J = 6.2 Hz, 2H), 2.81 – 2.71 (m, 2H) ppm.

^{13}C{^{1}H}-NMR (75 MHz, $CDCl_3$) δ = 205.6, 158.4, 137.6, 131.2 (q, J = 3.4 Hz), 130.4 (q, J = 33.3 Hz), 127.6, 123.9 (q, J = 272.8 Hz), 121.2 (q, J = 3.7 Hz), 36.5, 26.1 ppm.

^{19}F NMR (282 MHz, $CDCl_3$) δ = − 62.45 ppm.

IR (ATR): $\tilde{\nu}$ = 2930, 1716, 1623, 1586, 1442, 1405, 1326, 1294, 1263, 1236, 1195, 1161, 1114, 1059, 911, 839, 737, 702, 662, 615, 558, 508, 490, 407 cm^{-1}.

HRMS-EI (m/z) calculated for $C_{10}H_7F_3O$ $[M]^{\cdot+}$: 200.0444; found, 200.0443.

In a subsequent step, a round bottom flask equipped with a magnetic stir bar was charged with **S14** (1.00 g, 5.00 mmol, 1.0 equiv.) and methanol (10 mL). Subsequently $NaBH_4$ (189.2 mg, 5.00 mmol, 1.0 equiv.) was added in one portion and the resulting reaction mixture stirred at room temperature for 16 h. The solvent was then removed under reduced pressure and the residue diluted with ice cold water (20 mL) and extracted with Et_2O (3 × 50 mL).The combined organic layers were dried over Na_2SO_4, filtered, and concentrated under reduced pressure. The oil thus obtained was dissolved in $CHCl_3$ (25 mL) and *p*-TSA (86.3 mg, 0.500 mmol, 0.1 equiv.) was added. This reaction mixture was stirred at ambient temperature until consumption of the starting material (monitored by TLC). Then, the solvent was removed under reduced pressure and the remaining residue purified by chromatography on silica gel eluting with hexane. **4f** is obtained as a colorless oil (529.3 mg, 2.870 mmol, 58%).

^{1}H-NMR (400 MHz, $CDCl_3$): *δ* = 7.65 (s, 1H), 7.56 (d, *J* = 7.8 Hz, 1H), 7.46 (d, *J* = 7.9 Hz, 1H), 7.26 (brs, 1H), 6.92 (dd, *J* = 4.4, 1.2 Hz, 1H), 6.69 (dd, *J* = 3.8, 1.9 Hz, 1H), 3.46 (s, 2H) ppm.

^{13}C{^{1}H}-NMR (101 MHz, $CDCl_3$) δ = 147.4, 145.4, 136.1, 131.6, 129.1 (q, *J* = 31.9 Hz), 124.9 (q, *J* = 273.2 Hz), 123.9, 121.7 (q, *J* = 3.9 Hz), 117.8 (q, *J* = 4.4 Hz), 39.28 ppm.

^{19}F NMR (377 MHz, $CDCl_3$) δ = – 61.70 ppm.

IR (ATR): $\tilde{\nu}$ = 1433, 1386, 1357, 1320, 1276, 1229, 1199, 1157, 1108, 1071, 1054, 943, 918, 894, 828, 730, 714, 647, 579, 563, 536, 449, 440 cm^{-1}.

HRMS-EI (m/z) calculated for $C_{10}H_7F_3$ $[M]^{\cdot+}$: 184.0494; found, 184.0492.

General procedure C for the synthesis of 3-substituted indenes (**4k – 4l**):

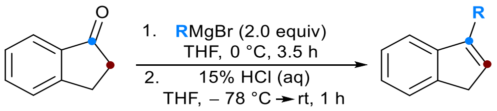

This protocol modifies a literature known procedure.[21,22] To a Schlenk flask equipped with a magnetic stir bar the corresponding RMgBr was added as a solution in Et_2O or THF (2.0 equiv.) and diluted with THF until its concentration was 0.4 M. Then, the solution was cooled to 0 °C in an ice-bath and a THF solution of 1-indanone (2.0 M) was added dropwise (1.0 equiv.). The reaction mixture was then stirred for 3.5 h. and subsequently cooled to –78 °C. Then, few drops of a 15% HCl solution were added, and the mixture allowed to reach ambient temperature. One hour later the reaction mixture was transferred into a separatory funnel and extracted with Et_2O (3 × 50 mL). The combined organic layers were washed with sat. $NaHCO_3$ solution, water and brine, and finally dried over Na_2SO_4. The solvent was removed under reduced pressure and the residue obtained was purified by chromatography on silica gel eluting with the indicated solvent mixtures.

Synthesis of **4k**:

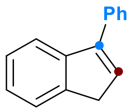

Prepared following general procedure C from 1-indanone (2.00 g, 15.1 mmol, 1.0 equiv.) and phenylmagnesium bromide as solution in Et_2O (10.0 mL, 30.3 mmol, 3.0 M, 2.0 equiv.). Crude **4k** was purified by chromatography on silica gel eluting with pentane. **4k** was obtained as a colorless oil (2.17 g, 11.3 mmol, 75%).

^{1}H NMR (400 MHz, $CDCl_3$): δ = 7.67 – 7.60 (m, 3H), 7.57 (d, *J* = 7.3 Hz, 1H), 7.48 (t, *J* = 7.4 Hz, 2H), 7.45 – 7.32 (m, 2H), 7.32 – 7.26 (m, 1H), 6.61 (t, *J* = 2.2 Hz, 1H), 3.54 (d, *J* = 2.2 Hz, 2H) ppm.

^{13}C{^{1}H} NMR (101 MHz, $CDCl_3$) δ = 145.3, 144.9, 144.1, 136.3, 131.1, 128.7, 127.9, 127.7, 126.3, 125.0, 124.3, 120.5, 38.3 ppm.

HRMS-ESI (m/z) calculated for $C_{15}H_{12}$ $[M+H]^+$: 193.1012; found: 193.1011.

The spectral data were in accordance with those reported in literature.[22]

Synthesis of **4l**:

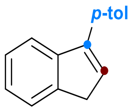

Prepared following general procedure C from 1-indanone (1.00 g, 7.57 mmol, 1.0 equiv.) and *p*-tolylmagnesium bromide as solution in THF (15.1 mL, 15.1 mmol, 1.0 M, 2.0 equiv.). Crude **4l** was purified by chromatography on silica gel eluting with pentane. **4l** is obtained as a colorless oil (1.14 g, 5.53 mmol, 73%).

^{1}H NMR (400 MHz, $CDCl_3$): δ = 7.66 (d, *J* = 7.6 Hz, 1H), 7.60 – 7.56 (m, 3H), 7.38 (t, *J* = 7.5 Hz, 1H), 7.32 – 7.29 (m, 3H), 6.60 (t, *J* = 2.2 Hz, 1H), 3.55 (d, *J* = 2.2 Hz, 2H), 2.47 (s, 3H) ppm.

^{13}C{^{1}H} NMR (101 MHz, $CDCl_3$) δ = 145.2, 144.9, 144.2, 137.4, 133.4, 130.5, 129.4, 127.7, 126.3, 124.9, 124.2, 120.5, 38.3, 21.4 ppm.

HRMS-EI (m/z) calculated for $C_{16}H_{14}$ $[M+H]^+$: 207.1168; found: 207.1166.

The spectral data were in accordance with those reported in literature.[23]

General procedure D for the synthesis of 1-substituted indenes (**4m – 4q**):

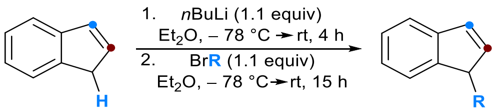

This protocol follows a modified literature procedure.[24] A Schlenk flask containing a solution of 1-*H*-indene (10.0 mmol, 1.0 equiv.) in Et_2O (20 mL) was cooled to – 78 °C and then, *n*-butyllithium (2.5 M in hexanes, 11.0 mmol, 4.4 ml, 1.1 equiv.) was dropwise added. After this, the cooling bath was removed, the reaction was allowed to reach room temperature, and additionally stirred for 4 h. After that the reaction mixture was cooled to again – 78 °C, and the desired alkyl bromide added dropwise. Finally, the reaction mixture was slowly warmed to ambient temperature and stirred for 15 h. Evaporation of the solvents at low pressure afforded a residue that was extrated with pentane (2 × 50 mL). The combined extracts were

concentrated under reduced pressure to yield the desired indene derivatives. These did not need further purification.

Synthesis of **4m**:

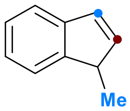

Prepared following general procedure D from 1*H*-indene (1.16 g, 10.0 mmol, 1.0 equiv.) and methyliodid (1.55 g, 11.0 mmol, 0.680 ml, 1.1 equiv.). **4m** was obtained as a yellow liquid (1,12 g, 8.60 mmol, 87%).

^{1}H NMR (400 MHz, $CDCl_3$): δ = 7.48 (d, J = 7.3 Hz, 1H), 7.41 (d, J = 6.6 Hz, 1H), 7.36 – 7.23 (m, 2H), 6.84 (dd, J = 5.6, 1.9 Hz, 1H), 6.54 (dd, J = 5.5, 1.9 Hz, 1H), 3.55 (q, J = 7.6 Hz, 1H), 1.38 (d, J = 7.6 Hz, 3H) ppm.

^{13}C{^{1}H} NMR (101 MHz, $CDCl_3$) δ = 149.3, 144.1, 141.5, 130.3, 126.5, 124.9, 122.7, 121.1, 45.2, 16.1 ppm.

HRMS-EI (m/z) calculated for $C_{10}H_{10}$ $[M]^{\cdot+}$: 130.0777; found, 130.0777.

The spectral data were in accordance with those reported in literature.[24]

Synthesis of **4n**:

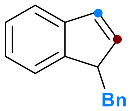

Prepared following general procedure D from 1*H*-indene (1.16 g, 10.0 mmol, 1.0 equiv.) and benzyl bromide (1.87 g, 11.0 mmol, 1.30 ml, 1.1 equiv.). **4n** was obtained as a yellow liquid (1.97 g, 9.53 mmol, 96%).

^{1}H NMR (400 MHz, $CDCl_3$): δ = 7.50 – 7.30 (m, 8H), 7.25 (q, J = 7.2 Hz, 1H), 6.87 (d, J = 5.5 Hz, 1H), 6.52 (d, J = 5.2 Hz, 1H), 3.80 (t, J = 7.1 Hz, 1H), 3.19 (dd, J = 13.5, 6.8 Hz, 1H), 2.79 (dd, J = 13.5, 9.2 Hz, 1H) ppm.

^{13}C{^{1}H} NMR (101 MHz, $CDCl_3$) δ = 147.2, 144.5, 140.5, 139.1, 131.1, 129.2, 128.5, 126.8, 126.4, 124.8, 123.3, 121.3, 51.9, 38.1 ppm.

HRMS-EI (m/z) calculated for $C_{16}H_{14}$ $[M]^{\cdot+}$: 206.1090; found, 206.1087.

The spectral data were in accordance with those reported in literature.[24]

Synthesis of **4o**:

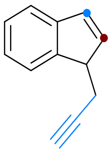

Prepared following general procedure D from 1*H*-indene (1.16 g, 10.0 mmol, 1.0 equiv.) and 3-bromoprop-1-yne (1.63 g, 11.0 mmol, 1.22 mL (80% wt in toluene), 1.1 equiv.). **4o** was obtained as a yellow liquid (1,39 g, 8.99 mmol, 90%).

^{1}H NMR (400 MHz, $CDCl_3$): δ = 7.56 (d, J = 7.4 Hz, 1H), 7.37 (d, J = 7.3 Hz, 1H), 7.29 (tt, J = 7.7, 1.6 Hz, 1H), 7.25 – 7.19 (m, 1H), 6.86 (dt, J = 5.7, 1.7 Hz, 1H), 6.60 (dt, J = 5.6, 1.6 Hz, 1H), 3.63 (t, J = 7.3 Hz, 1H), 2.71 – 2.58 (m, 1H), 2.40 – 2.33 (m, 1H), 2.06 – 2.04 (m, 1H) ppm.

$^{13}C\{^1H\}$ NMR (101 MHz, $CDCl_3$) δ = 146.3, 144.4, 138.3, 131.9, 127.2, 125.2, 123.2, 121.3, 82.8, 69.3, 48.9, 21.2 ppm.

IR (ATR): $\tilde{\nu}$ = 3293, 3066, 2907, 2118, 1608, 1458, 1427, 1362, 1304, 1266, 1217, 1165, 1068, 1019, 997, 936, 891, 868, 816, 773, 745, 723, 712, 631, 580, 556, 540, 522, 486, 454, 426, 409 cm^{-1}.

HRMS-EI (m/z) calculated for $C_{12}H_{10}$ $[M]^{\cdot+}$: 154.0777; found, 154.0777.

Synthesis of **4p**:

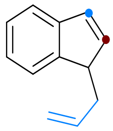

Prepared following general procedure D from 1*H*-indene (1.16 g, 10.0 mmol, 1.0 equiv.) and 3-bromoprop-1-ene (1.32 g, 11.0 mmol, 0.942 ml, 1.1 equiv.). **4p** was obtained as a yellow liquid (1,37 g, 8.76 mmol, 88%).

^1H NMR (400 MHz, $CDCl_3$): δ = 7.46 (d, J = 7.3 Hz, 1H), 7.37 (d, J = 7.4 Hz, 1H), 7.28 (t, J = 7.4 Hz, 1H), 7.21 (t, J = 7.4 Hz, 1H), 6.83 (dd, J = 5.6, 2.0 Hz, 1H), 6.55 (dd, J = 5.6, 2.0 Hz, 1H), 5.86 (ddt, J = 17.1, 10.1, 7.0 Hz, 1H), 5.11 (dq, J = 17.1, 1.7 Hz, 1H), 5.06 (d, J = 10.8 Hz, 1H), 3.54 (dd, J = 8.1, 5.8 Hz, 1H), 2.71 – 2.59 (m, 1H), 2.37 – 2.23 (m, 1H) ppm.

$^{13}C\{^1H\}$ NMR (101 MHz, $CDCl_3$) δ = 147.3, 144.5, 139.1, 136.4, 131.2, 126.7, 124.8, 123.2, 121.2, 116.5, 50.0, 35.8 ppm.

IR (ATR): $\tilde{\nu}$ = 3064, 1640, 1458, 1437, 1362, 1018, 996, 912, 865, 773, 743, 725, 712, 638, 610, 561, 542, 441, 404 cm^{-1}.

HRMS-EI (m/z) calculated for $C_{12}H_{12}$ $[M]^{\cdot+}$: 156.0934; found, 156.0933.

Synthesis of **4q**:

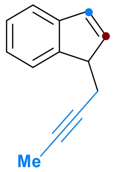

Prepared following general procedure D from 1*H*-indene (1.16 g, 10.0 mmol, 1.0 equiv.) and 1-bromobut-2-yne (1.46 g, 11.0 mmol, 0.962 ml, 1.1 equiv.). **4q** was obtained as a yellow liquid (1,45 g, 8.60 mmol, 86%).

^1H NMR (400 MHz, $CDCl_3$): δ = 7.55 (d, J = 7.4 Hz, 1H), 7.37 (d, J = 7.4 Hz, 1H), 7.29 (t, J = 7.4 Hz, 1H), 7.21 (t, J = 7.4 Hz, 1H), 6.87 – 6.81 (m, 2H), 6.62 (dd, J = 5.4, 1.4 Hz, 2H), 3.59 (t, J = 7.8 Hz, 1H), 2.59 (ddt, J = 15.1, 7.3, 2.8 Hz, 1H), 2.32 – 2.21 (m, 1H), 1.85 (t, J = 2.6 Hz, 3H) ppm.

$^{13}C\{^1H\}$ NMR (101 MHz, $CDCl_3$) δ = 146.6, 144.4, 139.0, 131.4, 127.0, 125.0, 123.2, 121.2, 77.7, 76.6, 49.7, 21.6, 3.6 ppm.

IR (ATR): $\tilde{\nu}$ = 3064, 2957, 2916, 2853, 1770, 1457, 1427, 1362, 1307, 1246, 1067, 1019, 991, 936, 866, 773, 744, 710, 578, 556, 447, 404 cm^{-1}.

HRMS-EI (m/z) calculated for $C_{13}H_{12}$ $[M]^{\cdot+}$: 168.0934; found, 168.0932.

Synthesis of **4u**:

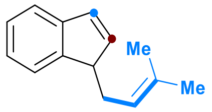

Prepared following general procedure D from 1*H*-indene (1.00 g, 8.60 mmol, 1.0 equiv.) and 1-bromo-3-methylbut-2-ene (1.41 g, 9.47 mmol, 1.09 ml, 1.1 equiv.). **4u** was obtained as a yellow liquid (1,45 g, 7.87 mmol, 91%).

^{1}H NMR (300 MHz, $CDCl_3$): δ = 7.44 (d, *J* = 7.2 Hz, 1H), 7.36 (d, *J* = 7.3 Hz, 1H), 7.31 – 7.13 (m, 2H), 6.80 (dd, *J* = 5.7, 1.8 Hz, 1H), 6.52 (dd, *J* = 5.6, 1.9 Hz, 1H), 5.25 (t, *J* = 7.1 Hz, 1H), 3.46 (t, *J* = 7.5 Hz, 1H), 2.54 (dt, *J* = 13.8, 6.6 Hz, 1H), 2.18 (dt, *J* = 15.1, 8.1 Hz, 1H), 1.73 (s, 3H), 1.60 (s, 3H) ppm.

^{13}C{^{1}H} NMR (75 MHz, $CDCl_3$) δ = 147.7, 144.6, 139.7, 133.2, 130.8, 126.6, 124.8, 123.1, 122.3, 121.1, 50.8, 30.2, 25.9, 18.0 ppm.

IR (ATR): $\tilde{\nu}$ = 3062, 2965, 2912, 2856, 1608, 1456, 1375, 1104, 1018, 984, 934, 852, 806, 771, 740, 711, 618, 562, 453 cm^{-1}.

HRMS-EI (m/z) calculated for $C_{14}H_{16}$ $[M]^{\cdot+}$: 184.1247; found, 184.1245.

SYNTHESIS OF NAPHTHALENES

General procedure E for the synthesis of naphtalenes (**5a-t**):

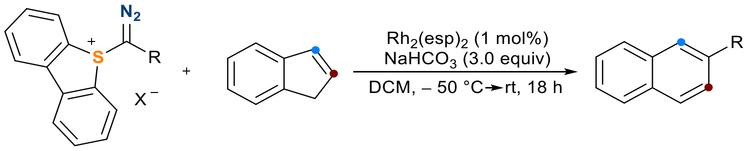

A Schlenk flask was charged with the desired α-diazo sulfonium salt **1** (0.2 mmol, 1.0 equiv.), $Rh_2(esp)_2$ (1.5 mg, 2.0 μmol, 1 mol%) and $NaHCO_3$ (50.5 mg, 0.6 mmol, 3.0 equiv.) and cooled to – 50 °C. Then, DCM (2.8 mL) was added and finally, a solution of the desired indene **4** in DCM was added. The resulting mixture was allowed to reach room temperature overnight. After this, the reaction mixture was transferred to a separation funnel, diluted with DCM (5.0 mL), and washed with water (5 mL). The aq. phase was extracted with DCM (3 × 20 mL), the combined organic phase was dried over Na_2SO_4, and finally filtered. Removal of solvents under reduced pressure afforded a residue, which was purified by chromatography on silica gel using as eluent the solvent mixture indicated.

Synthesis of **5a**:

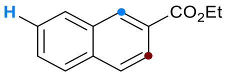

Prepared following the general procedure E from 1*H*-indene **4a** (116.5 mg, 1.0 mmol, 5.0 equiv.) and α-diazo sulfonium salt **1g** (76.9 mg, 0.2 mmol, 1.0 equiv.). **5a** was purified by column chromatography on silica gel using pentane/EtOAc (100/0→100/1 (v/v)) as eluent. **5a** was obtained as a colorless oil (29.8 mg, 149.0 μmol, 74%).

^{1}H NMR (300 MHz, $CDCl_3$): δ = 8.62 (s, 1H), 8.08 (dd, J = 8.6, 1.7 Hz, 1H), 7.96 (d, J = 8.0, 1H), 7.88 (d, J = 8.4 Hz, 2H), 7.57 (m, 2H), 4.46 (q, J = 7.1 Hz, 2H), 1.46 (t, J = 7.1 Hz, 3H) ppm.

^{13}C{^{1}H} NMR (101 MHz, $CDCl_3$) δ = 166.9, 135.6, 132.6, 131.1, 129.5, 128.3, 128.2, 127.9, 126.7, 125.4, 61.2, 14.5 ppm.

HRMS-ESI (m/z) calculated for $C_{13}H_{12}O_2$ $[M+Na]^+$: 223.0730; found, 223.0724.

The spectral data were in accordance with those reported in literature.[25]

Synthesis of **5b**:

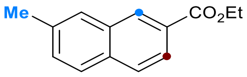

Prepared following the general procedure E from 5-methylindene **4b** (130.8 mg, 1.0 mmol, 5.0 equiv.) and α-diazo sulfonium salt **1g** (0.2 mmol, 1.0 equiv.). **5b** was initially purified by column chromatography on silica gel using pentane/EtOAc (100/0→100/1 (v/v)) as eluent. **F**urther purification by preparative HPLC was needed to obtain **5b** analytically pure. White solid (26.3 mg, 122.8 μmol, 61%).

^{1}H NMR (300 MHz, $CDCl_3$): δ = 8.53 (s, 1H), 8.00 (dd, J = 8.6, 1.7 Hz, 1H), 7.89 – 7.68 (m, 3H), 7.42 (dd, J = 8.5, 1.8 Hz, 1H), 4.44 (q, J = 7.1 Hz, 2H), 1.45 (t, J = 7.1 Hz, 3H) ppm.

^{13}C{^{1}H} NMR (101 MHz, $CDCl_3$) δ = 167.1, 136.5, 133.9, 132.9, 130.6, 130.5, 128.4, 127.9, 127.7, 124.6, 61.2, 21.8, 14.5 ppm.

IR (ATR): $\tilde{\nu}$ = 2981, 2933, 2360, 2340, 1699, 1462, 1442, 1388, 1365, 1334, 1280, 1233, 1194, 1168, 1130, 1094, 1009, 924, 862, 847, 808, 781, 752, 718, 609, 475, 434 cm^{-1}.

HRMS-ESI (m/z) calculated for $C_{14}H_{14}O_2$ $[M+Na]^+$: 237.0886; found, 237.0881.

Melting point: 66.4 °C

Synthesis of **5c**:

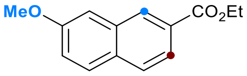

Prepared following the general procedure E from 5-methoxyindene **4c** (145.3 mg, 1.0 mmol, 5.0 equiv.) and α-diazo sulfonium salt **1g** (77.0 mg, 0.2 mmol, 1.0 equiv.). Crude **5c** was purified by column chromatography on silica gel using pentane/EtOAc (100/0→500/1 (v/v)) as eluent. Colorless oil (28.7 mg, 124.6 μmol, 62%).

^{1}H NMR (300 MHz, $CDCl_3$): δ = 8.51 (s, 1H), 7.93 (dd, J = 8.6, 1.7 Hz, 1H), 7.83 – 7.72 (m, 2H), 7.34 – 7.15 (m, 2H), 4.44 (q, J = 7.1 Hz, 2H), 3.94 (s, 3H), 1.45 (t, J = 7.1 Hz, 3H) ppm.

$^{13}C\{^1H\}$ NMR (101 MHz, $CDCl_3$) δ = 167.0, 158.1, 133.8, 131.1, 129.7, 129.2, 128.2, 127.8, 123.2, 121.2, 106.9, 61.1, 55.4, 14.4 ppm.

IR (ATR): $\tilde{\nu}$ = 2979, 2936, 1770, 1710, 1631, 1605, 1512, 1462, 1444, 1391, 1365, 1334, 1276, 1236, 1214, 1190, 1171, 1124, 1095, 1026, 953, 910, 844, 806, 767, 745, 715, 631, 600, 525, 492, 472, 450, 433, 408 cm^{-1}.

HRMS-ESI (m/z) calculated for $C_{14}H_{14}O_3$ $[M+Na]^+$: 253.0835; found, 253.0843.

The spectral data were in accordance with those reported in literature.[26]

Synthesis of **5d**:

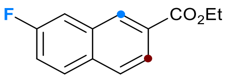

Prepared following the general procedure E from 5-fluoroindene **4d** (135.1 mg, 1.0 mmol, 5.0 equiv.) and α-diazo sulfonium salt **1g** (77.3 mg, 0.2 mmol, 1.0 equiv.). **5d** was purified by column chromatography on silica gel using pentane/EtOAc (100/0→100/1 (v/v)) as eluent. Further purification by preparative HPLC was needed to obtain **5d** analytically pure. Colorless oil (25.3 mg, 115.9 µmol, 58%).

^1H NMR (300 MHz, $CDCl_3$): δ = 8.54 (s, 1H), 8.03 (dd, J = 8.6, 1.7 Hz, 1H), 7.87 (dd, J = 8.6, 4.1 Hz, 2H), 7.56 (dd, J = 9.5, 2.6 Hz, 1H), 7.36 (td, J = 8.7, 2.6 Hz, 1H), 4.45 (q, J = 7.1 Hz, 2H), 1.45 (t, J = 7.1 Hz, 3H) ppm.

$^{13}C\{^1H\}$ NMR (101 MHz, $CDCl_3$) δ = 166.7, 162.3, 159.8, 133.5, 133.4, 132.6, 130.4, 130.3, 130.2, 128.9, 128.2, 124.8, 124.8, 118.8, 118.6, 112.5, 112.3, 61.4, 14.5 ppm.

^{19}F NMR (377 MHz, $CDCl_3$) δ = – 113.48 -113.52 (m)

IR (ATR): $\tilde{\nu}$ = 2981, 2360, 2341, 1714, 1636, 1608, 1581, 1513, 1458, 1365, 1329, 1269, 1228, 1204, 1152, 1120, 1096, 1020, 967, 913, 845, 803, 763, 745, 722, 629, 471, 430 cm^{-1}.

HRMS-ESI (m/z) calculated for $C_{13}H_{11}F_1O_2$ $[M+Na]^+$: 241.0635; found, 241.0650.

Synthesis of **5e**:

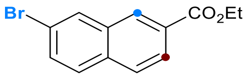

Prepared following the general procedure E from 5-bromoindene **4e** (196.5 mg, 1.0 mmol, 5.0 equiv.) and α-diazo sulfonium salt **1g** (77.1 mg, 0.2 mmol, 1.0 equiv.). Crude **5e** was purified by column chromatography on silica gel using a pentane/EtOAc (100/0→100/1 (v/v)) mixture as eluent. Pale yellow solid (31.6 mg, 113.2 µmol, 56%).

^1H NMR (300 MHz, $CDCl_3$): δ = 8.50 (s, 1H), 8.11 (s, 1H), 8.08 (dd, J = 8.6, 1.7 Hz, 1H), 7.84 (d, J = 8.6 Hz, 1H), 7.74 (d, J = 8.8 Hz, 1H), 7.64 (dd, J = 8.8, 1.9 Hz, 1H), 4.45 (q, J = 7.1 Hz, 2H), 1.45 (t, J = 7.1 Hz, 3H) ppm.

$^{13}C\{^1H\}$ NMR (101 MHz, $CDCl_3$) δ = 166.5, 133.9, 133.7, 131.6, 130.9, 130.0, 129.5, 128.9, 128.2, 125.9, 120.7, 61.4, 14.5 ppm.

IR (ATR): $\tilde{\nu}$ = 3064, 2979, 2955, 2933, 2898, 2866, 2359, 2340, 1713, 1584, 1497, 1473, 1441, 1418, 1388, 1363, 1323, 1259, 1227, 1186, 1159, 1140, 1118, 1103, 1062, 1023, 952.663, 939, 917, 853, 806, 765, 749, 618, 597, 586, 518, 474 cm^{-1}.

HRMS-EI (m/z) calculated for $C_{13}H_{11}BrO_2$ $[M]^{\cdot+}$: 277.9937; found, 277.9937.

Melting point: 91.1 °C

Synthesis of **5f**:

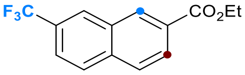

Prepared following the general procedure E from 5-(trifluoromethyl)-1*H*-indene **4f** (184.3 mg, 1.0 mmol, 5.0 equiv.) and α-diazo sulfonium salt **1g** (77.2 mg, 0.2 mmol, 1.0 equiv.). **5f** was purified by column chromatography on silica gel pentane/EtOAc (100/0→200/1 (v/v)) as eluent. Colorless oil (35.8 mg, 133.5 μmol, 67%).

^{1}H NMR (300 MHz, $CDCl_3$): δ = 8.69 (s, 1H), 8.26 (s, 1H), 8.20 (dd, *J* = 8.6, 1.7 Hz, 1H), 7.96 (dd, *J* = 13.8, 8.6 Hz, 2H), 7.74 (dd, *J* = 8.6, 1.8 Hz, 1H), 4.46 (q, *J* = 7.1 Hz, 2H), 1.46 (t, *J* = 7.1 Hz, 3H) ppm.

^{13}C{^{1}H} NMR (75 MHz, $CDCl_3$) δ = 166.3, 136.7, 131.7, 131.5, 129.3, 129.0, 128.7 (q, *J* = 31.7 Hz), 128.2, 127.7, 127.1 (q, *J* = 4.6 Hz), 124.2 (q, *J* = 270.7 Hz), 123.8 (q, *J* = 3.0 Hz), 61.5, 14.5 ppm.

^{19}F NMR (377 MHz, $CDCl_3$) δ = − 62.5 ppm.

IR (ATR): $\tilde{\nu}$ = 3070, 2990, 1770, 1714, 1638, 1434, 1375, 1341, 1305, 1232, 1191, 1164, 1113, 1064, 1019, 946, 854, 817, 777, 748, 667, 640, 605, 589, 574, 523, 503, 479, 450, 416 cm^{-1}.

HRMS-EI (m/z) calculated for $C_{14}H_{11}F_3O_2$ $[M]^{\cdot+}$: 268.0706; found, 268.0704.

Synthesis of **10f**:

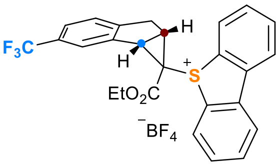

Obtained as a side-product in the synthesis of **5f**. After elution of **5f**, the solvent of the column chromatography was changed to DCM/MeOH (100/0→95/5 (v/v)). Compound **10f** was thus obtained as a white solid (12.6 mg, 23.3 μmol, 12%). Heating of **10f** at 80 °C for 4 days transformed it quantitatively to **5f**.

Single crystals suitable for X-ray diffraction were obtained by slow diffusion of Et_2O into a solution of **10f** in MeCN.

^{1}H NMR (400 MHz, CD_3CN): δ = 8.35 (ddt, *J* = 15.5, 8.1, 0.8 Hz, 2 H), 8.26 (dt, *J* = 7.8, 1.7 Hz, 2 H), 7.97 (tt, *J* = 7.7, 1.3 Hz, 2 H), 7.83 (tdd, *J* = 7.7, 4.0, 1.2 Hz, 2 H), 7.70 (s, 1H), 7.57 – 7.49 (m, 1 H), 7.33 (d, *J* = 8.0 Hz, 1 H), 3.96 (d, *J* = 7.5 Hz, 1 H), 3.50 (ddt, *J* = 18.7, 6.7, 1.3 Hz, 1 H), 3.42 – 3.31 (m, 3H), 3.25 (ddd, *J* = 7.7, 6.7, 0.9 Hz, 1 H), 0.47 (t, *J* = 7.1 Hz, 3 H) ppm.

^{13}C{^{1}H} NMR (75 MHz, $CDCl_3$) δ = 162.0, 148.9, 141.0, 140.9, 139.4, 136.0, 136.0, 132.4, 132.4, 130.2, 130.0, 129.7, 128.1, 128.0, 126.5, 126.4 (q, *J* = 3.8 Hz), 125.4, 125.4, 125.3 (q, *J* = 271.0 Hz), 123.7 (q, *J* = 3.8 Hz), 63.9, 48.0, 38.2, 34.7, 31.9, 13.2 ppm.

^{19}F NMR (377 MHz, CD_3CN) δ = − 62.7, − 151.7 ppm.

IR (ATR): $\tilde{\nu}$ = 3091, 1725, 1446, 1425, 1334, 1292, 1161, 1119, 1058, 1037, 1015, 959, 931, 919, 898, 839, 818, 759, 726, 704, 642, 519, 492, 470, 438, 417 cm^{-1}.

HRMS-ESI (m/z) calculated for $C_{26}H_{20}F_3O_2S$ $[M-BF_4]^+$: 453.1131; found, 453.1128.

Melting point: 194 °C decomp.

Synthesis of **5g**:

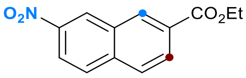

Prepared following the general procedure E from 5-nitroindene **4g** (163.5 mg, 1.0 mmol, 5.0 equiv.) and α-diazo sulfonium salt **1g** (77.0 mg, 0.2 mmol, 1.0 equiv.). **5g** was purified by column chromatography on silica gel using a pentane/EtOAc (100/0→50/1 (v/v)) as eluent. White solid (35.9 mg, 146.4 μmol, 72%).

^{1}H NMR (300 MHz, $CDCl_3$): δ = 8.91 (d, J = 2.3 Hz, 1H), 8.78 (dd, J = 1.5, 0.8 Hz, 1H), 8.31 (ddd, J = 19.1, 8.8, 2.0 Hz, 2H), 8.01 (dd, J = 8.8, 5.2 Hz, 2H), 4.48 (q, J = 7.1 Hz, 2H), 1.47 (t, J = 7.1 Hz, 3H) ppm.

^{13}C{^{1}H} NMR (101 MHz, $CDCl_3$) δ = 165.8, 146.1, 137.8, 132.7, 131.4, 130.0, 129.6, 129.2, 128.4, 125.9, 121.5, 61.7, 14.5 ppm.

IR (ATR): $\tilde{\nu}$ = 3082, 2979, 2924, 2858, 2359, 1709, 1631, 1605, 1521, 1445, 1337, 1267, 1227, 1190, 1150, 1137, 1118, 1099, 1084, 1016, 946, 857, 843, 816, 801, 769, 747, 739, 610, 594, 546, 512, 468, cm^{-1}.

HRMS-ESI (m/z) calculated for $C_{13}H_{11}NO_4$ $[M+Na]^+$: 268.0580; found, 268.0582.

Melting point: 130.4 °C

Synthesis of **5h**:

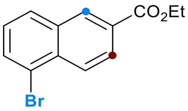

Prepared following the general procedure E from 7-bromo-1*H*-indene **4h** (196.2 mg, 1.0 mmol, 5.0 equiv.) and α-diazo sulfonium salt **1g** (77.2 mg, 0.2 mmol, 1.0 equiv.). **5h** was was purified by column chromatography on silica gel using a pentane/EtOAc (500/1→200/1 (v/v)) mixture as eluent. Colorless oil (43.5 mg, 155.8 μmol, 78%).

^{1}H NMR (300 MHz, $CDCl_3$): δ = 8.57 (d, J = 1.6 Hz, 1H), 8.26 (d, J = 8.9 Hz, 1H), 8.15 (dd, J = 8.9, 1.7 Hz, 1H), 7.88 (dd, J = 12.2, 7.3 Hz, 2H), 7.36 (t, J = 7.8 Hz, 1H), 4.46 (q, J = 7.1 Hz, 2H), 1.46 (t, J = 7.1 Hz, 3H) ppm.

^{13}C{^{1}H} NMR (101 MHz, $CDCl_3$) δ = 166.4, 134.1, 133.9, 132.2, 131.3, 129.4, 128.7, 127.6, 127.1, 126.8, 122.8, 61.4, 14.5 ppm.

IR (ATR): $\tilde{\nu}$ = 2941, 2900, 1718, 1624, 1594, 1561, 1479, 1461, 1392, 1365, 1336, 1271, 1238, 1194, 1140, 1100, 1024, 960, 912, 888, 867, 829, 781, 766, 666, 611, 588, 538, 520, 408 cm^{-1}.

HRMS-ESI (m/z) calculated for $C_{13}H_{11}Br_1O_2$ $[M+Na]^+$: 300.9835; found, 300.9838.

Synthesis of **5i**:

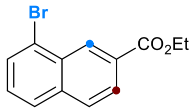

Prepared following the general procedure E from 4-bromo-1*H*-indene **4i** (194.7 mg, 1.0 mmol, 5.0 equiv.) and α-diazo sulfonium salt **1g** (77.2 mg, 0.2 mmol, 1.0 equiv.). The reaction mixture was heated at 80 °C for 4 days. The residue was purified by chromatography on silica gel eluting with pentane/EtOAc (100/0→50/1 (v/v)). **5i** was obtained as a colorless oil (14.2 mg, 50.9 µmol, 30%).

^{1}H NMR (400 MHz, $CDCl_3$): *δ* = 8.99 (s, 1 H), 8.12 (d, *J* = 8.5 Hz, 1 H), 7.86 (dd, *J* = 16.0, 8.2 Hz, 3 H), 7.42 (t, *J* = 7.7 Hz, 1 H), 4.47 (q, *J* = 7.1 Hz, 2 H), 1.47 (t, *J* = 7.0 Hz, 4 H) ppm.

^{13}C{^{1}H} NMR (101 MHz, $CDCl_3$) δ = 166.6, 136.8, 131.6, 130.9, 130.2, 129.4, 128.8, 128.6, 127.9, 126.4, 124.4, 61.5, 14.6 ppm.

IR (ATR): $\tilde{\nu}$ = 2981, 2928, 1716, 1623, 1554, 1442, 1356, 1271, 1241, 1188, 1140, 1099, 1020, 971, 910, 837, 790, 753, 659 cm^{-1}.

HRMS-ESI (m/z) calculated for $C_{13}H_{11}Br_1O_2$ $[M+H]^+$: 279.0015; found, 279.0017.

Synthesis of **10i**:

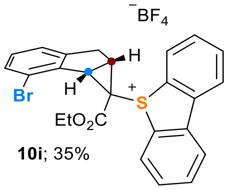

If the former reaction is not heated at 80°C, but just concentrated and submitted to column chromatography on silica gel eluting with DCM/MeOH (97/3 (v/v)), **10i** (38.9 mg, 70.6 µmol, 35%) is obtained as a white solid.

Single crystals suitable for X-ray diffraction were obtained by slow diffusion of Et_2O into a solution of **10i** in MeCN.

^{1}H NMR (400 MHz, CD_3CN): *δ* = 8.42 (d, *J* = 8.1 Hz, 1 H), 8.33 (d, *J* = 8.1 Hz, 1 H), 8.25 (d, *J* = 7.8 Hz, 2 H), 7.96 (q, *J* = 7.8 Hz, 2 H), 7.82 (dt, *J* = 14.9, 7.8 Hz, 2 H), 7.32 (d, *J* = 7.3 Hz, 1 H), 7.17 – 7.07 (m, 2 H), 3.84 (d, *J* = 7.4 Hz, 1 H), 3.66 – 3.43 (m, 2 H), 3.39 (t, *J* = 7.0 Hz, 1 H), 3.29 (m, 2 H), 0.47 (t, *J* = 7.1 Hz, 3 H) ppm.

^{13}C{^{1}H} NMR (101 MHz, CD_3CN) δ = 161.5, 145.8, 141.1, 140.6, 138.9, 136.0, 135.9, 132.5, 132.2, 131.4, 131.0, 130.6, 129.8, 128.8, 127.5, 125.3, 125.3, 125.0, 121.0, 63.8, 48.2, 38.6, 35.6, 31.6, 13.2 ppm.

^{19}F NMR (377 MHz, CD_3CN) δ = – 151.56 ppm.

IR (ATR): $\tilde{\nu}$ = 1728, 1567, 1449, 1428, 1359, 1304, 1186, 1032, 861, 822, 781, 760, 723, 704, 519, 487, 417 cm^{-1}.

HRMS-ESI (m/z) calculated for $C_{25}H_{20}Br_1O_2S$ $[M-BF_4]^+$: 463.0362; found, 463.0355.

Melting point: 204 °C decomp.

Synthesis of **5j**:

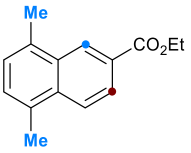

Prepared following the general procedure E from 4,7-dimethyl-1*H*-indene **4j** (144.7 mg, 1.0 mmol, 5.0 equiv.) and α-diazo sulfonium salt **1g** (77.1 mg, 0.2 mmol, 1.0 equiv.). **5j** was purified by column chromatography on silica gel using pentane/EtOAc (100/0→200/1 (v/v)) as eluent. Colorless oil (32.3 mg, 141.5 μmol, 71%).

^{1}H NMR (300 MHz, $CDCl_3$): δ = 8.78 (d, J = 1.7 Hz, 1H), 8.11 (dd, J = 8.8, 1.7 Hz, 1H), 8.03 (d, J = 8.8 Hz, 1H), 7.28 (q, J = 7.6, 7.2 Hz, 2H), 4.47 (q, J = 7.1 Hz, 2H), 2.74 (s, 3H), 2.67 (s, 3H), 1.47 (t, J = 7.1 Hz, 3H) ppm.

^{13}C{^{1}H} NMR (101 MHz, $CDCl_3$) δ = 167.2, 135.1, 134.1, 132.4, 132.1, 128.7, 127.8, 127.1, 125.0, 124.9, 61.2, 19.5, 19.5, 14.6 ppm.

IR (ATR): $\tilde{\nu}$ = 2970, 2931, 2863, 1712, 1619, 1597, 1466, 1439, 1380, 1365, 1277, 1231, 1212, 1169, 1145, 1105, 1021, 908, 835, 822, 778, 752, 705, 416 cm^{-1}.

HRMS-ESI (m/z) calculated for $C_{15}H_{16}O_2$ $[M+H]^+$: 229.1223; found, 229.1220.

Synthesis of **5k**:

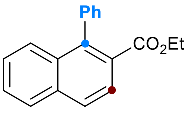

Prepared following the general procedure E from 3-phenylindene **4k** (192.7 mg, 1.0 mmol, 5.0 equiv.) and α-diazo sulfonium salt **1g** (77.3 mg, 0.2 mmol, 1.0 equiv.). **5k** was purified by column chromatography on silica gel using pentane/EtOAc (100/0→400/1 (v/v)) as eluent. Preparative HPLC was necessary to obtain **5k** in analytically pure form. Colorless oil (29.7 mg, 107.5 μmol, 54%).

^{1}H NMR (300 MHz, $CDCl_3$): δ = 8.07 – 7.79 (m, 3H), 7.66 – 7.51 (m, 2H), 7.51 – 7.38 (m, 4H), 7.37 – 7.29 (m, 2H), 4.06 (q, J = 7.1 Hz, 2H), 0.96 (t, J = 7.1 Hz, 3H).

^{13}C{^{1}H} NMR (101 MHz, $CDCl_3$) δ = 168.8, 141.3, 139.3, 134.8, 132.7, 129.9, 128.7, 128.0, 128.0, 127.9, 127.8, 127.5, 127.4, 126.7, 125.6, 61.0, 13.8 ppm.

IR (ATR): $\tilde{\nu}$ = 3056, 2979, 2928, 1706, 1460, 1442, 1386, 1371, 1330, 1277, 1239, 1130, 1108, 1018, 969, 868, 826, 798, 762, 699, 608, 572, 431 cm^{-1}.

HRMS-ESI (m/z) calculated for $C_{19}H_{16}O_2$ $[M+H]^+$: 277.1223; found, 277.1213.

The spectral data were in accordance with those reported in literature.[27]

Synthesis of **5l**:

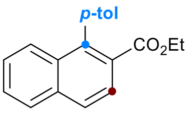

Prepared following the general procedure E from 3-tolylindene **4l** (206.7 mg, 1.0 mmol, 5.0 equiv.) and α-diazo sulfonium salt **1g** (77.2 mg, 0.2 mmol, 1.0 equiv.). **5l** was purified by column chromatography on silica gel using pentane/EtOAc (100/0→400/1 (v/v)) as eluent. Analytically pure **5l** was only obtained after purification by preparative HPLC. Yellow oil (28.9 mg, 99.5 μmol, 50%).

^{1}H NMR (400 MHz, $CDCl_3$): δ = 7.94 (s, 3H), 7.68 (d, J = 8.6 Hz, 1H), 7.58 (t, J = 7.5 Hz, 1H), 7.45 (t, J = 7.5 Hz, 1H), 7.32 (d, J = 7.9 Hz, 2H), 7.25 (d, J = 7.8 Hz, 2H), 4.13 (q, J = 7.3 Hz, 2H), 2.51 (s, 3H), 1.05 (t, J = 7.2 Hz, 3H) ppm.

^{13}C{^{1}H} NMR (101 MHz, $CDCl_3$) δ = 168.8, 141.4, 137.0, 136.2, 134.8, 132.9, 129.7, 128.8, 128.7, 128.0, 127.9, 127.7, 127.4, 126.6, 125.6, 61.0, 21.5, 13.9 ppm.

IR (ATR): $\tilde{\nu}$ = 2978, 2917, 1708, 1514, 1462, 1383, 1371, 1330, 1277, 1238, 1130, 1106, 1020, 974, 868, 814, 765, 724, 674, 615, 571, 519, 431 cm^{-1}

HRMS-ESI (m/z) calculated for $C_{20}H_{18}O_2$ $[M+H]^+$: 291.1380; found, 291.1380.

The spectral data were in accordance with those reported in literature.[28]

Synthesis of **5m**:

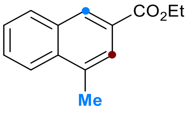

Prepared following the general procedure E from 1-methyl-1*H*-indene **4m** (131.4 mg, 1.0 mmol, 5.0 equiv.) and α-diazo sulfonium salt **1g** (77.1 mg, 0.2 mmol, 1.0 equiv.). **5m** was purified by column chromatography on silica gel using pentane/EtOAc (100/0→500/1 (v/v)) as eluent. Further purification by preparative HPLC was necessary to obtain **5m** analytically pure. Yellow oil (27.3 mg, 127.4 µmol, 64%).

^{1}H NMR (300 MHz, $CDCl_3$): δ = 8.47 (s, 1H), 8.02 (d, J = 8.4 Hz, 1H), 7.96 (d, J = 8.2 Hz, 1H), 7.92 (s, 1H), 7.63 (t, J = 7.6 Hz, 1H), 7.55 (t, J = 7.5 Hz, 1H), 4.44 (q, J = 7.2 Hz, 2H), 1.45 (t, J = 7.1 Hz, 3H) ppm.

^{13}C{^{1}H} NMR (101 MHz, $CDCl_3$) δ = 167.1, 134.9, 132.8, 130.2, 129.6, 128.8, 128.2, 127.4, 126.4, 125.8, 124.3, 61.2, 19.5, 14.6 ppm.

IR (ATR): $\tilde{\nu}$ =2976, 2904, 1711, 1447, 1397, 1377, 1365, 1289, 1238, 1201, 1162, 1145, 1103, 1019, 950, 894, 776, 744, 701, 597, 531, 490, 420 cm^{-1}.

HRMS-ESI (m/z) calculated for $C_{14}H_{14}O_2$ $[M+H]^+$: 215.1067; found, 215.1061.

Synthesis of **5n**:

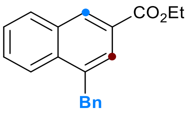

Prepared following the general procedure E from 1-benzyl-1*H*-indene **4n** (207.7 mg, 1.0 mmol, 5.0 equiv.) and α-diazo sulfonium salt **1g** (77.3 mg, 0.2 mmol, 1.0 equiv.). **5n** was purified by column chromatography on silica gel using pentane/EtOAc (100/0→200/1 (v/v)) as eluent. Further purification by preparative HPLC was necessary to obtain **5n** analytically pure. Yellow oil (41.9 mg, 144.3 µmol, 72%).

^{1}H NMR (300 MHz, $CDCl_3$): δ = 8.54 (s, 1H), 8.04 – 7.94 (m, 3H), 7.59 – 7.48 (m, 2H), 7.31 – 7.23 (m, 2H), 7.20 (m, 3H), 4.52 – 4.40 (m, 4H), 1.45 (t, J = 7.1 Hz, 3H) ppm.

^{13}C{^{1}H} NMR (101 MHz, $CDCl_3$) δ = 167.0, 140.4, 137.2, 134.5, 133.3, 130.4, 130.3, 128.7, 128.6, 128.4, 127.5, 126.8, 126.4, 126.3, 124.6, 61.2, 39.3, 14.6 ppm.

IR (ATR): $\tilde{\nu}$ = 3024, 2979, 1712, 1599, 1493, 1455, 1394, 1366, 1285, 1239, 1203, 1156, 1104, 1073, 1029, 947, 907, 774, 753, 715, 697, 667, 596, 514, 458, 422 cm^{-1}.

HRMS-ESI (m/z) calculated for $C_{20}H_{18}O_2$ $[M+H]^+$: 291.1380; found, 291.1379.

The spectral data were in accordance with those reported in literature.[29]

Synthesis of **5o**:

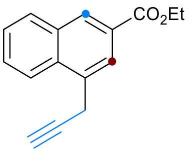

Prepared following the general procedure E from 1-(prop-2-yn-1-yl)-1*H*-indene **4o** (153.7 mg, 1.0 mmol, 5.0 equiv.) and α-diazo sulfonium salt **1g** (77.3 mg, 0.2 mmol, 1.0 equiv.). **5o** was was purified by column chromatography on silica gel using a pentane/EtOAc (100/0→200/1 (v/v)) mixture as eluent. Yellow oil (31.2 mg, 130.9 µmol, 65%).

^{1}H NMR (300 MHz, $CDCl_3$): δ = 8.54 (s, 1H), 8.20 (s, 1H), 8.07 (d, *J* = 8.5 Hz, 1H), 7.99 (d, *J* = 8.2 Hz, 1H), 7.66 (ddd, *J* = 8.4, 6.8, 1.4 Hz, 1H), 7.57 (ddd, *J* = 8.1, 6.8, 1.2 Hz, 1H), 4.45 (q, *J* = 7.1 Hz, 2H), 4.02 (d, *J* = 2.7 Hz, 2H), 2.28 (s, 1H), 1.45 (t, *J* = 7.1 Hz, 3H) ppm.

^{13}C{^{1}H} NMR (101 MHz, $CDCl_3$) δ = 166.8, 133.6, 133.1, 132.7, 130.9, 130.4, 128.7, 127.6, 126.7, 125.2, 123.6, 81.3, 71.8, 61.3, 23.0, 14.6 ppm.

IR (ATR): $\tilde{\nu}$ = 3247, 2984, 1698, 1623, 1471, 1391, 1366, 1309, 1265, 1242, 1217, 1203, 1168, 1109, 1019, 944, 906, 887, 773, 742, 702, 595, 512, 490, 416 cm^{-1}.

HRMS-ESI (m/z) calculated for $C_{16}H_{14}O_2$ $[M+H]^+$: 239.1067; found, 239.1061.

Synthesis of **5p**:

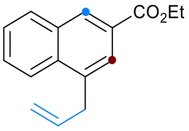

Prepared following the general procedure E from 1-allyl-1*H*-indene **4p** (157.1 mg, 1.0 mmol, 5.0 equiv.) and α-diazo sulfonium salt **1g** (77.0 mg, 0.2 mmol, 1.0 equiv.). **5p** was purified by column chromatography on silica gel using pentane/EtOAc (100/0→200/1 (v/v)) as eluent. Further purification by preparative HPLC was necessary to obtain **5p** analytically pure. Yellow oil (27.2 mg, 113.2 µmol, 56%).

^{1}H NMR (300 MHz, $CDCl_3$): δ = 8.50 (s, 1H), 8.05 (d, *J* = 8.4 Hz, 1H), 8.01 – 7.91 (m, 2H), 7.68 – 7.47 (m, 2H), 6.12 (ddt, *J* = 16.6, 10.3, 6.3 Hz, 1H), 5.19 – 5.03 (m, 2H), 4.45 (q, *J* = 7.1 Hz, 2H), 3.87 (d, *J* = 5.8 Hz, 2H), 1.45 (t, *J* = 7.1 Hz, 3H).

^{13}C{^{1}H} NMR (101 MHz, $CDCl_3$) δ = 167.0, 136.8, 136.6, 134.3, 133.2, 130.3, 130.1, 128.3, 127.5, 126.4, 125.7, 124.3, 116.7, 61.2, 37.4, 14.6 ppm.

IR (ATR): $\tilde{\nu}$ = 3056, 2978, 1712, 1637, 1578, 1508, 1455, 1395, 1366, 1281, 1239, 1202, 1162, 1145, 1104, 1028, 954, 908, 860, 772, 744, 668, 596, 532, 488, 433, 419 cm^{-1}.

HRMS-ESI (m/z) calculated for $C_{16}H_{16}O_2$ $[M+H]^+$: 241.1223 ; found, 241.1216.

Synthesis of **5q**:

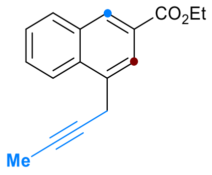

Prepared following the general procedure E from 1-(but-2-yn-1-yl)-1*H*-indene **4q** (169.2 mg, 1.0 mmol, 5.0 equiv.) and α-diazo sulfonium salt **1g** (77.2 mg, 0.2 mmol, 1.0 equiv.). **5q** was purified by column chromatography on silica gel using a pentane/EtOAc (100/0→200/1 (v/v)) mixture as eluent. Colorless oil (25.8 mg, 102.3 µmol, 51%).

^{1}H NMR (400 MHz, $CDCl_3$): δ = 8.52 (s, 1H), 8.18 (s, 1H), 8.09 (d, J = 8.5 Hz, 1H), 7.98 (d, J = 8.2 Hz, 1H), 7.65 (t, J = 7.7 Hz, 1H), 7.56 (t, J = 7.5 Hz, 1H), 4.45 (q, J = 7.2 Hz, 2H), 3.96 (s, 2H), 1.86 (s, 3H), 1.45 (t, J = 7.0 Hz, 3H) ppm.

^{13}C{^{1}H} NMR (101 MHz, $CDCl_3$) δ = 166.9, 134.2, 133.8, 133.1, 130.6, 130.3, 128.5, 127.6, 126.6, 125.1, 123.8, 79.2, 76.2, 61.2, 23.3, 14.6, 3.8 ppm.

IR (ATR): $\tilde{\nu}$ = 2979, 2917, 1712, 1627, 1601, 1578, 1508, 1455, 1399, 1366, 1288, 1271, 1240, 1200, 1164, 1143, 1103, 1017, 947, 907, 889, 774, 666, 640, 593, 523, 488, 429 cm^{-1}.

HRMS-ESI (m/z) calculated for $C_{17}H_{16}O_2$ $[M+H]^+$: 253.1223; found, 253.1220.

Synthesis of **5r**:

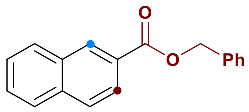

Prepared following the general procedure E from 1*H*-indene (116.6 mg, 1.0 mmol, 5.0 equiv.) and α-diazo sulfonium salt **1f** (102.2 mg, 0.2 mmol, 1.0 equiv.). **5r** was purified by column chromatography using a pentane/EtOAc (100/0→200/1 (v/v)) mixture as eluent. Colorless oil (33.4 mg, 127.3 µmol, 63%).

^{1}H NMR (300 MHz, $CDCl_3$): δ = 8.66 (s, 1H), 8.11 (dd, J = 8.6, 1.7 Hz, 1H), 7.96 (d, J = 7.9 Hz, 1H), 7.89 (d, J = 8.7 Hz, 2H), 7.64 – 7.49 (m, 4H), 7.47 – 7.34 (m, 3H), 5.45 (s, 2H) ppm.

^{13}C{^{1}H} NMR (101 MHz, $CDCl_3$) δ = 166.7, 136.2, 135.7, 132.6, 131.3, 129.5, 128.8, 128.4, 128.4, 128.3, 127.9, 127.5, 126.8, 125.4, 67.0 ppm.

IR (ATR): $\tilde{\nu}$ = 3035, 2968, 2898, 1702, 1496, 1463, 1368, 1348, 1280, 1224, 1192, 1126, 1088, 1076, 954, 909, 875, 832, 780, 754, 699, 598, 581, 528, 475 cm^{-1}.

HRMS-ESI (m/z) calculated for $C_{18}H_{14}O_2$ $[M+Na]^+$: 285.0886; found, 285.0888.

The spectral data were in accordance with those reported in literature.[30]

Synthesis of **5s**:

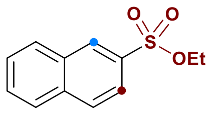

Prepared following the general procedure E from 1*H*-indene (116.5 mg, 1.0 mmol, 5.0 equiv.) and α-diazo sulfonium salt **1a** (96.5 mg, 0.2 mmol, 1.0 equiv.). The reaction mixture was further heated to 40 °C for 12 h. **5s** was purified by column chromatography on silica gel using a pentane/EtOAc (100/0→20/1 (v/v)) mixture as eluent. Yellow oil (22.7 mg, 96.1 µmol, 48%).

^{1}H NMR (300 MHz, $CDCl_3$): *δ* = 8.50 (s, 1H), 8.06 – 7.82 (m, 4H), 7.75 – 7.57 (m, 2H), 4.17 (q, *J* = 7.1 Hz, 2H), 1.31 (t, *J* = 7.1 Hz, 3H) ppm.

^{13}C{^{1}H} NMR (101 MHz, $CDCl_3$) δ = 135.4, 133.3, 132.1, 129.8, 129.7, 129.5, 129.4, 128.1, 127.9, 122.7, 67.3, 14.9 ppm.

IR (ATR): $\tilde{\nu}$ = 3059, 2984, 1771, 1757, 1623, 1591, 1506, 1455, 1348, 1270, 1241, 1174, 1077, 1000, 949, 911, 861, 816, 750, 656, 616, 552, 477, 410 cm^{-1}.

HRMS-ESI (m/z) calculated for $C_{12}H_{12}O_3S$ $[M+Na]^+$: 259.0399; found, 259.0398.

Synthesis of **5t**:

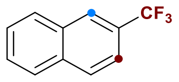

Prepared following the general procedure E from 1*H*-indene (116.2 mg, 1.0 mmol, 5.0 equiv.) and α-diazo sulfonium salt **1i** (88.6 mg, 0.20 mmol, 1.0 equiv.). The reaction mixture was heated at 80 °C for 4 days. **5t** was obtained as a white solid after purification by column chromatography on silica gel using pentane as eluent, 28% yield.

The spectral data were in accordance with those reported in literature.[31]

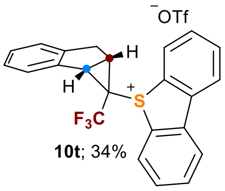

If the former reaction mixture is not heated, the cyclopropyl-substituted dibenzothiophenium triflate **10t** is obtained as a side-product. In this case, once **5t** has been eluted from the column chromatography, the eluent was changed to DCM/MeOH (100/0→95/5 (v/v)). **10t** was obtained as a pale-yellow solid (31.4 mg, 67.1 μmol, 34%).

^{1}H NMR (400 MHz, CD_3CN): *δ* = 8.40 (d, *J* = 8.0 Hz, 2H), 8.33 (d, *J* = 7.8 Hz, 2H), 8.08 – 7.98 (m, 2H), 7.92 – 7.81 (m, 2H), 7.53 (dd, *J* = 6.4, 2.2 Hz, 1H), 7.35 – 7.20 (m, 3H), 3.91 (d, *J* = 8.0 Hz, 1H), 3.51 (dd, *J* = 18.8, 7.0 Hz, 1H), 3.36 (d, *J* = 18.8 Hz, 1H), 3.11 (t, *J* = 7.4 Hz, 1H) ppm.

^{13}C{^{1}H} NMR (101 MHz, CD_3CN) δ = 145.8 (q, *J* = 1.8 Hz), 141.1, 141.1, 136.5, 136.4, 135.4, 132.7, 132.6, 130.4, 130.2, 130.0, 128.3, 127.2, 126.6, 126.3, 125.8, 125.8, 125.3, 124.7 (q, *J* = 281.4 Hz), 122.1 (q, *J* = 320.0 Hz), 46.3 (q, *J* = 32.8 Hz), 39.8, 34.4 (q, *J* = 1.9 Hz), 33.5 ppm.

^{19}F NMR (377 MHz, CD_3CN) δ = − 53.8, − 79.3 ppm.

IR (ATR): $\tilde{\nu}$ = 2994, 2358, 1770, 1758, 1450, 1372, 1339, 1247, 1192, 1140, 1062, 1027, 961, 916, 878, 800, 759, 736, 721, 705, 635, 571, 515, 484, 455, 439, 419, 404 cm^{-1}.

HRMS-ESI (m/z) calculated for $C_{23}H_{16}F_3S$ $[M{-}OTf]^+$: 381.0919; found, 381.0917.

Melting point: 181.5 °C decomp.

Synthesis of **11**:

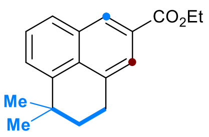

Prepared following the general procedure E from 1-(3-methylbut-2-en-1-yl)-1*H*-indene **4u** (185.3 mg, 1.0 mmol, 5.0 eq) and α-diazo sulfonium salt **1g** (77.2 mg, 0.2 mmol, 1.0 eq). An initial column chromatography on silica gel was performed using a pentane/EtOAc (100/0→200/1 (v/v)) mixture as eluent, but analytically pure **11** was only obtained after further purification by preparative HPLC. Colorless oil (36.5 mg, 136.0 µmol, 68%). As a side-product **5a** was also isolated (5.90 mg, 29.5 µmol, 15%).

^{1}H NMR (300 MHz, $CDCl_3$): δ = 8.43 (s, 1H), 7.85 (s, 1H), 7.79 (d, J = 8.1 Hz, 1H), 7.58 (dd, J = 7.2, 1.3 Hz, 1H), 7.49 (t, J = 7.7 Hz, 1H), 4.44 (q, J = 7.1 Hz, 2H), 3.20 (t, J = 6.4 Hz, 2H), 1.92 (t, J = 6.4 Hz, 2H), 1.45 (t, J = 7.1 Hz, 3H), 1.40 (s, 6H) ppm.

^{13}C{^{1}H} NMR (75 MHz, $CDCl_3$) δ = 167.2, 145.1, 136.2, 133.2, 131.2, 129.1, 127.6, 127.0, 126.5, 123.5, 123.4, 61.1, 37.4, 34.7, 30.3, 27.2, 14.6 ppm.

IR (ATR): $\tilde{\nu}$ = 2958, 2925, 1712, 1620, 1599, 1584, 1458, 1421, 1370, 1323, 1273, 1202, 1106, 1054, 1020, 942, 914, 894, 862, 812, 775, 622, 556, 489, 468, 435 cm^{-1}.

HRMS-ESI (m/z) calculated for $C_{18}H_{20}O_2$ $[M+H]^+$: 269.1536; found, 269.1534.

General procedure F for the synthesis of adducts (**6b – 6e**):

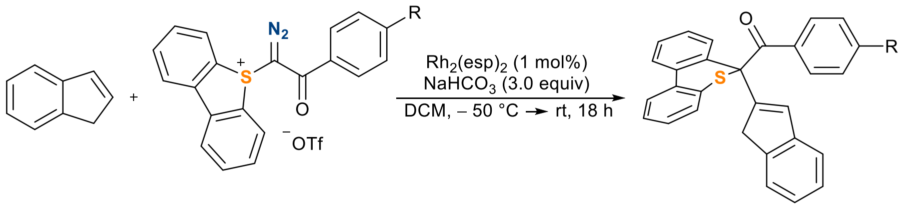

Compounds **1b – 1e** (0.20 mmol, 1.0 equiv.), $NaHCO_3$ (50.4 mg, 0.60 mmol, 3.00 equiv) and $Rh_2(esp)_2$ (1.52 mg, 0.002 mmol, 1 mol%) were dded as solids to a Schlenk tube equipped with a stirring bar. The flask was cooled to −50 °C and DCM (3 mL) and indene (1.00 mmol, 5.0 equiv.) were added slowly. The reaction mixture was then allowed to reach room temperature overnight and after this, it was diluted with DCM (5 mL) and washed with water (5 mL). The aqueous phase was extracted with DCM (3 x 25 mL) and the combined organic layers were dried over anhydrous $MgSO_4$. After evaporation of the solvents at low pressure, a residue was obtained Purified by column chromatography on silica eluting with the indicated solvent mixture afforded **6b-e** analytically pure.

Synthesis of **6b**:

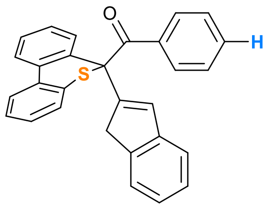

Prepared following the general procedure F from 1*H*-indene (116.4 mg, 1.0 mmol, 5.0 equiv.) and α-diazo sulfonium salt **1b** (95.7 mg, 0.20 mmol, 1.0 equiv.). **6b** was purified by column chromatography using a *n*-hexane/EtOAc (100/0→100/1 (v/v)) mixture as eluent. White solid (68.0 mg, 0.16 mmol, 82%).

^{1}**H NMR** (400 MHz, CD_3CN): *δ* = 7.89 (t, *J* = 8.2 Hz, 2H), 7.83 (d, *J* = 7.9 Hz, 2H), 7.52 – 7.48 (m, 2H), 7.43 (d, *J* = 7.2 Hz, 1H), 7.39 – 7.12 (m, 9H), 7.08 (d, *J* = 7.8 Hz, 1H), 6.58 (s, 1H), 3.75 – 3.56 (m, 2H) ppm.

^{13}**C{^{1}H} NMR** (101 MHz, $CDCl_3$) δ = 198.3, 145.4, 143.8, 143.7, 138.1, 135.8, 135.2, 134.6, 134.5, 132.4, 130.4, 130.4, 128.5, 128.4, 128.1, 127.6, 127.4, 126.8, 126.7, 126.5, 125.9, 125.4, 123.9, 121.6, 63.6, 40.2, 1.2 ppm.

IR (ATR): $\tilde{\nu}$ = 3057, 2958, 2924, 1733, 1676, 1594, 1577, 1487, 1471, 1459, 1443, 1426, 1387, 1372, 1296, 1229, 1181, 1159,1126, 1097, 1075, 1043, 1009, 938, 917, 860, 837, 817, 779, 745, 717, 691, 658, 623, 607, 597, 564, 552, 494, 470, 446, 435, 416 cm^{-1}.

HRMS-ESI (m/z) calculated for $C_{29}H_{20}OS$ $[M+H]^+$: 417.1308; found, 417.1306.

Melting point: 182 °C

Synthesis of **6c**:

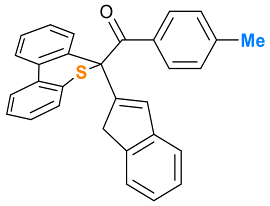

Prepared following the general procedure F from 1*H*-indene (116.6 mg, 1.0 mmol, 5.0 equiv.) and α-diazo sulfonium salt **1c** (100.3 mg, 0.20 mmol, 1.0 equiv.). **6c** was purified by column chromatography using a mixture of *n*-hexane/EtOAc (100/0→100/1 (v/v)) as eluent. White solid (40.1 mg, 0.09 mmol, 46%).

^{1}**H NMR** (400 MHz, CD_3CN): *δ* = 7.87 (t, *J* = 8.7 Hz, 2H), 7.80 (d, *J* = 7.9 Hz, 2H), 7.48 (t, *J* = 7.6 Hz, 1H), 7.38 (d, *J* = 7.2 Hz, 1H), 7.29 (dt, *J* = 15.0, 7.6 Hz, 2H), 7.23 – 7.04 (m, 8H), 6.51 (s, 1H), 3.74 – 3.49 (m, 2H), 2.30 (s, 3H).ppm.

^{13}**C{^{1}H} NMR** (101 MHz, CD_3CN) δ = 197.6, 146.6, 144.9, 144.4, 138.8, 136.0, 135.4, 135.3, 133.5, 131.5, 131.3, 129.4, 129.3, 129.2, 129.1, 129.1, 128.3, 127.9, 127.5, 127.1, 126.7, 126.3, 124.8, 122.2, 64.6, 40.5, 21.6 ppm.

IR (ATR): $\tilde{\nu}$ = 2993, 1769, 1673, 1603, 1372, 1241, 1181, 1046, 746, 717, 608 cm^{-1}.

HRMS-ESI (m/z) calculated for $C_{30}H_{22}OS$ $[M+H]^+$: 431.1464; found, 431.1460.

Melting point: 162 °C

Synthesis of **6d**:

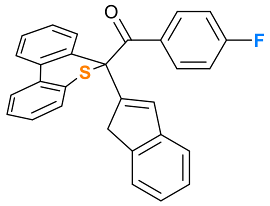

Prepared following the general procedure F from 1*H*-indene (116.5 mg, 1.0 mmol, 5.0 equiv.) and α-diazo sulfonium salt **1d** (99.3 mg, 0.20 mmol, 1.0 equiv.). **6d** was purified by column chromatography using a *n*-hexane/EtOAc (100/0→100/1 (v/v)) mixture as eluent. White solid (33.2 mg, 0.08 mmol, 38%).

^{1}**H NMR** (400 MHz, CD_3CN): *δ* = 7.95 – 7.85 (m, 4H), 7.50 (t, *J* = 7.6 Hz, 1H), 7.44 (d, *J* = 7.2 Hz, 1H), 7.37 – 7.12 (m, 8H), 7.03 (t, *J* = 8.9 Hz, 3H), 6.59 (s, 1H), 3.66 (q, *J* = 23.1 Hz, 2H) ppm.

$^{13}C\{^1H\}$ **NMR** (101 MHz, CD_3CN) δ = 197.1, 167.3, 164.8, 145.9, 144.5, 144.4, 138.9, 136.1, 135.6, 135.4, 134.3, 134.2, 132.7, 132.6, 130.9, 129.4, 129.4, 129.2, 128.6, 127.8, 127.6, 127.1, 126.8, 126.5, 124.9, 122.4, 115.8, 115.5, 64.4, 40.6 ppm.

^{19}F **NMR** (377 MHz, CD_3CN) δ = − 107.3 ppm.

IR (ATR): $\tilde{\nu}$ = 3059, 2360, 1735, 1677, 1594, 1504, 1232, 1158, 1045, 1010, 839, 717, 606 cm^{-1}.

HRMS-ESI (m/z) calculated for $C_{29}H_{19}FOS$ $[M+H]^+$: 435.1213; found, 435.1218.

Melting point: 105 °C

Synthesis of **6e**:

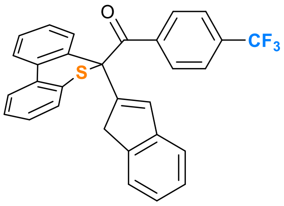

Prepared following the general procedure F from 1*H*-indene (116.8 mg, 1.0 mmol, 5.0 equiv.) and α-diazo sulfonium salt **1e** (109.4 mg, 0.20 mmol, 1.0 equiv.). **6e** was purified by column chromatography using a *n*-hexane/EtOAc (100/0→100/1 (v/v)) mixture as eluent. White solid (22.0 mg, 0.05 mmol, 23%).

^1H **NMR** (400 MHz, CD_3CN): δ = 7.87 (d, *J* = 8.0 Hz, 4H), 7.58 (d, *J* = 8.1 Hz, 2H), 7.53 – 7.44 (m, 2H), 7.40 – 7.19 (m, 5H), 7.18 – 7.00 (m, 3H), 6.72 (s, 1H), 3.80 (d, *J* = 23.1 Hz, 1H), 3.63 (d, *J* = 23.1 Hz, 1H) ppm.

$^{13}C\{^1H\}$ **NMR** (101 MHz, CD_3CN) δ = 198.4, 144.9, 144.4, 144.3, 139.9, 138.8, 136.1, 135.9, 135.3, 133.7 (q, *J* = 32.7 Hz), 131.4, 130.5, 129.5, 129.4, 129.3, 129.3, 128.7, 127.8, 127.7, 127.1, 126.9, 126.7, 125.6 (q, *J* = 3.8 Hz), 124.9, 124.8 (d, *J* = 272.2 Hz), 122.5, 64.3, 40.8 ppm.

^{19}F **NMR** (377 MHz, CD_3CN) δ = − 63.7 ppm.

IR (ATR): $\tilde{\nu}$ = 3059, 1735, 1683, 1443, 1427, 1406, 1323, 1241, 1169, 1128, 1068, 1010, 852, 748, 718, 627, 504, 412 cm^{-1}.

HRMS-ESI (m/z) calculated for $C_{30}H_{19}F_3OS$ $[M+Na]^+$: 507.1001; found, 507.1007.

Melting point: 122 °C

Synthesis of **7c**:

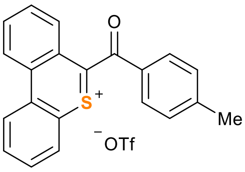

1c (100.1 mg, 0.20 mmol, 1.0 equiv.) and $Rh_2(esp)_2$ (1.60 mg, 2.00 µmol, 1 mol%) were added to a Schlenk tube. The solids were cooled to – 50 °C and DCM (3 mL) was added slowly. The reaction mixture was allowed to reach room temperature overnight. Removal of the solvent at reduced pressure afforded a residue that was washed with Et_2O (2 × 2 mL). Crystallization from DCM/Et_2O yielded **7c** as a dark yellow solid (12.8 mg, 85.9 µmol, 42%).

^{1}H NMR (400 MHz, CD_3CN): δ = 9.34 (dd, J = 8.5, 4.2 Hz, 2H), 8.66 (dd, J = 8.4, 1.3 Hz, 1H), 8.53 (ddd, J = 8.5, 7.0, 1.4 Hz, 1H), 8.33 (ddd, J = 8.6, 7.2, 1.3 Hz, 1H), 8.23 – 8.13 (m, 3H), 8.05 – 7.96 (m, 4H), 7.42 (d, J = 8.1 Hz, 2H), 2.47 (s, 4H) ppm.

^{13}C{^{1}H} NMR (101 MHz, CD_3CN) δ = 187.4, 182.9, 149.7, 142.5, 139.5, 135.2, 132.7, 132.2, 132.1, 131.9, 131.3, 131.0, 130.9, 130.7, 129.9, 129.5, 127.5, 127.1, 22.4 ppm.

^{19}F NMR (377 MHz, CD_3CN) δ = − 78.4 ppm.

IR (ATR): $\tilde{\nu}$ = 3067,1663, 1600, 1533, 1475, 1443, 1375, 1250, 1223, 1150, 1067, 1026, 861, 829, 746, 707, 683, 659, 634, 602, 570, 514, 476, 450, 416, 402 cm^{-1}.

HRMS-ESI (m/z) calculated for $C_{21}H_{15}OS$ [M−OTf]$^{+}$: 315.0838; found, 315.0848.

Melting point: > 153 °C decomp.

Synthesis of **8c**:

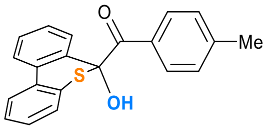

Prepared from α-diazo sulfonium salt **1c** (147.0 mg, 0.30 mmol). Following the same procedure as for **7c**, but submitting the crude reaction mixture to column chromatography (eluted with *n*-hexane/EtOAc (30/1 (v/v))). **8c** was obtained as a white solid (50 mg, 0.15 mmol, 50%).

^{1}H NMR (400 MHz, CD_3CN): δ = 8.07 – 7.97 (m, 4H), 7.53 (t, J = 7.7 Hz, 1H), 7.46 – 7.27 (m, 4H), 7.21 (d, J = 8.1 Hz, 2H), 7.12 (d, J = 7.9 Hz, 1H), 5.40 (s, 1H), 2.36 (s, 3H) ppm.

^{13}C{^{1}H} NMR (101 MHz, CD_3CN) δ = 196.3, 146.2, 136.4, 134.0, 133.6, 132.7, 130.9, 130.4, 130.2, 129.6, 129.3, 129.3, 129.0, 128.0, 127.3, 127.0, 126.8, 86.4, 21.7 ppm.

IR (ATR): $\tilde{\nu}$ = 3402, 3060, 2359, 1733, 1665, 1604, 1473, 1443, 1427, 1372, 1240, 1182, 1080, 1044, 973, 896, 743, 719, 677, 634, 429 cm^{-1}.

HRMS-ESI (m/z) calculated for $C_{21}H_{16}O_2S$ [M+Na]$^{+}$: 355.0763; found, 355.0762.

Melting point: 70 °C

Formation of bis-indenes (**9a – b**):

Synthesis of **9a**:

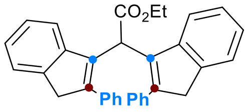

Prepared following the general procedure E from 2-phenyl-1*H*-indene **S9a** (193.5 mg, 1.0 mmol, 5.0 equiv.) and α-diazo sulfonium salt **1g** (77.2 mg, 0.2 mmol, 1.0 equiv.). **9a** was purified by column chromatography on silica gel using pentane/EtOAc (100/0→100/1 (v/v)) as eluent. Yellow oil (73.5 mg, 156.7 µmol, 78%).

^{1}H NMR (300 MHz, $CDCl_3$): δ = 7.66 – 7.59 (m, 4 H), 7.47 (s, 2 H), 7.37 (td, J = 8.0, 1.4 Hz, 5 H), 7.31 – 7.23 (m, 4 H), 7.21 (s, 1 H), 5.11 (s, 1 H), 4.25 (q, J = 7.1 Hz, 2 H), 3.78 (s, 2 H), 1.29 (t, J = 7.2 Hz, 3 H) ppm.

^{13}C{^{1}H} NMR (101 MHz, $CDCl_3$) δ = 173.2, 146.9, 144.6, 143.8, 136.1, 135.9, 128.8, 127.7, 127.3, 126.3, 125.8, 124.1, 121.0, 61.3, 57.3, 39.1, 14.4 ppm.

IR (ATR): $\tilde{\nu}$ = 2921, 2852, 1725, 1594, 1575, 1556, 1489, 1472, 1445, 1388, 1365, 1319, 1282, 1253, 1213, 1150, 1095, 1072, 1026, 956, 908, 881, 839, 805, 754, 727, 688, 667, 647, 594, 579, 559, 528, 492, 416 cm^{-1}.

HRMS-ESI (m/z) calculated for $C_{34}H_{28}O_2$ $[M+H]^+$: 469.2162; found, 469.2162.

Synthesis of **9b**:

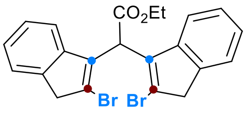

Prepared following the general procedure E from 2-bromo-1*H*-indene (196.1 mg, 1.0 mmol, 5.0 equiv.) and α-diazo sulfonium salt **1g** (77.1 mg, 0.2 mmol, 1.0 equiv.). **9b** was purified by column chromatography using pentane/EtOAc (100/0→100/1 (v/v)) as eluent. Yellow oil (63.4 mg, 133.7 μmol, 67%).

^{1}H NMR (300 MHz, $CDCl_3$): δ = 7.33 (s, 2 H), 7.28 – 7.15 (m, 4 H), 6.90 (s, 2 H), 5.02 (s, 1 H), 4.21 (q, J = 7.1 Hz, 2 H), 3.57 (s, 4 H), 1.26 (t, J = 7.1 Hz, 3 H) ppm.

^{13}C{^{1}H} NMR (101 MHz, $CDCl_3$) δ = 172.9, 143.3, 143.2, 135.8, 132.8, 127.3, 125.3, 123.6, 120.2, 61.4, 57.1, 45.6, 14.3 ppm.

IR (ATR): $\tilde{\nu}$ = 2979, 2931, 1768, 1754, 1730, 1587, 1549, 1470, 1428, 1388, 1367, 1245, 1151, 1057, 1026, 878, 849, 801, 754, 699, 597, 576, 449, 413 cm^{-1}.

HRMS-ESI (m/z) calculated for $C_{22}H_{18}Br_2O_2$ $[M+H]^+$: 472.9746; found, 472.9729.

DIFFERENTIAL SCANNING CALORIMETRY (DSC) OF α-DIAZO SULFONIUM SALTS

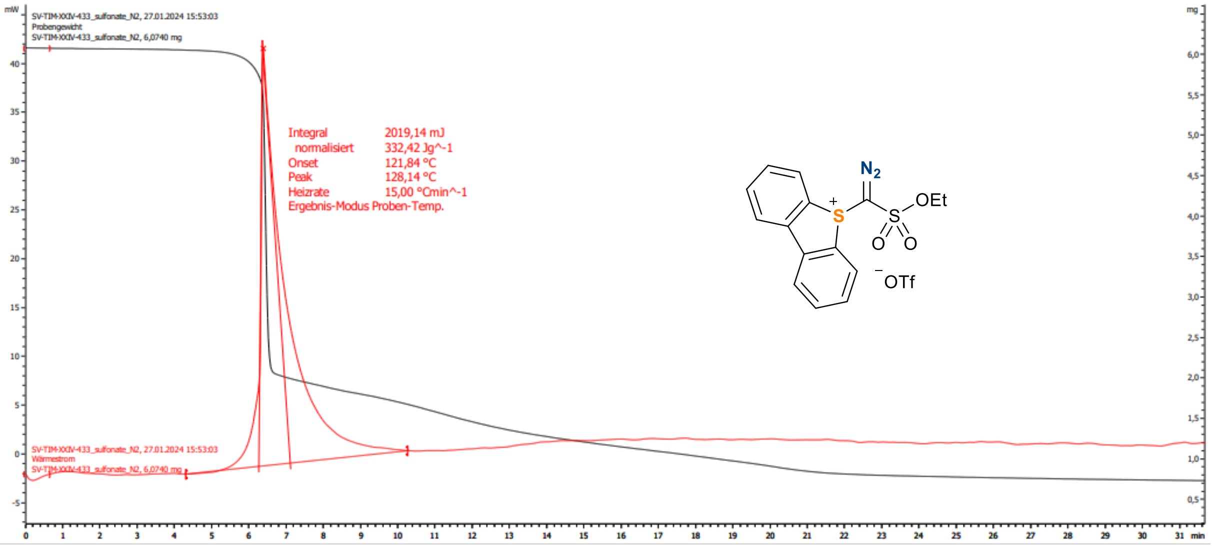

Figure S1: DSC measurement of compound **1a** under N_2.

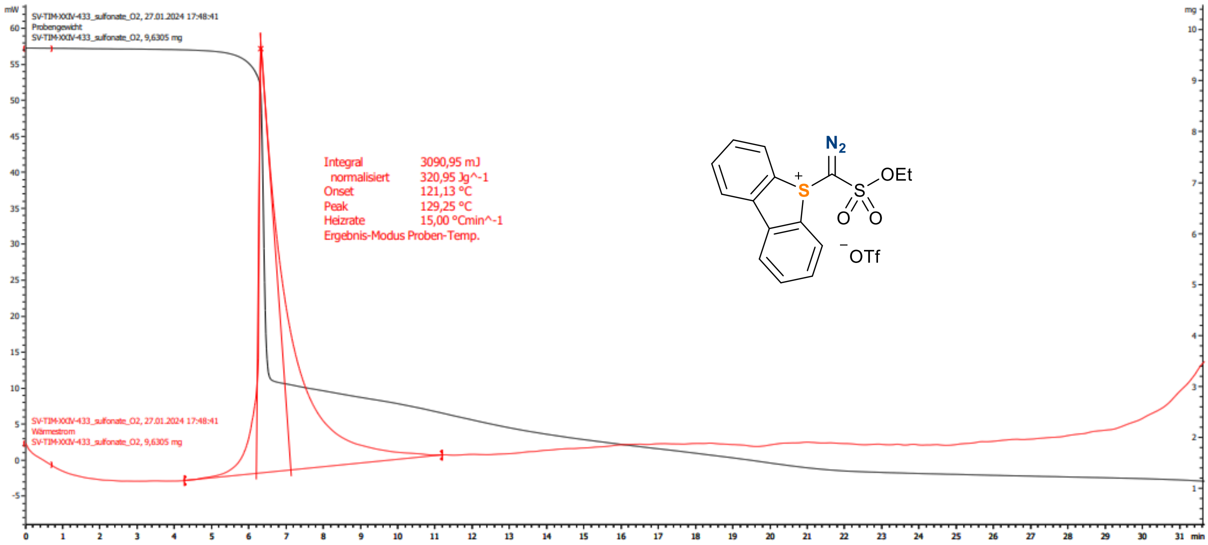

Figure S2: DSC measurement of compound **1a** under air.

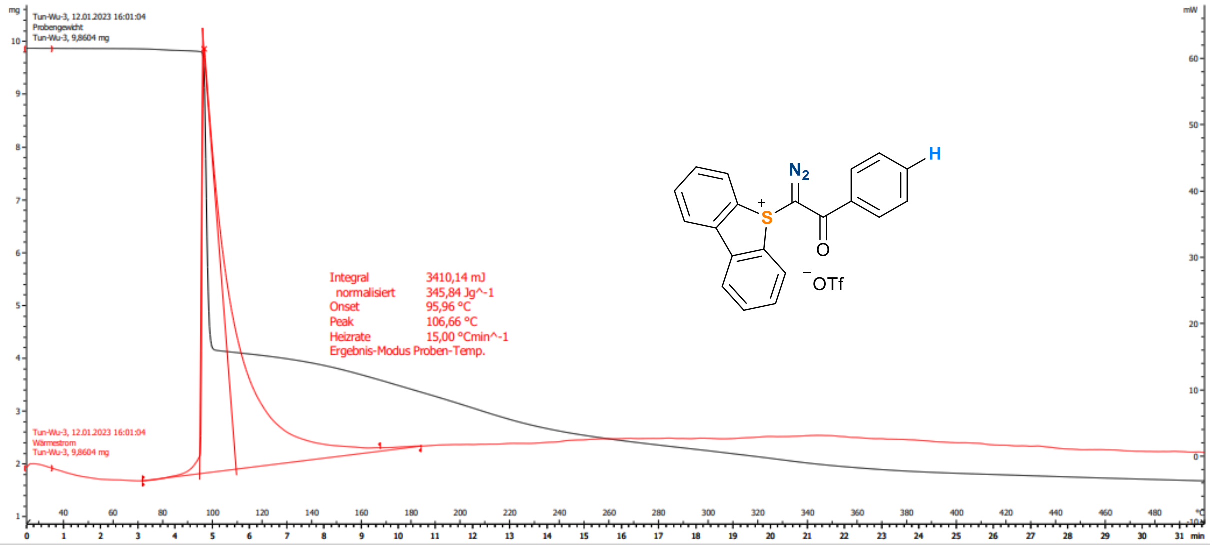

Figure S3: DSC measurement of compound **1b** under N_2.

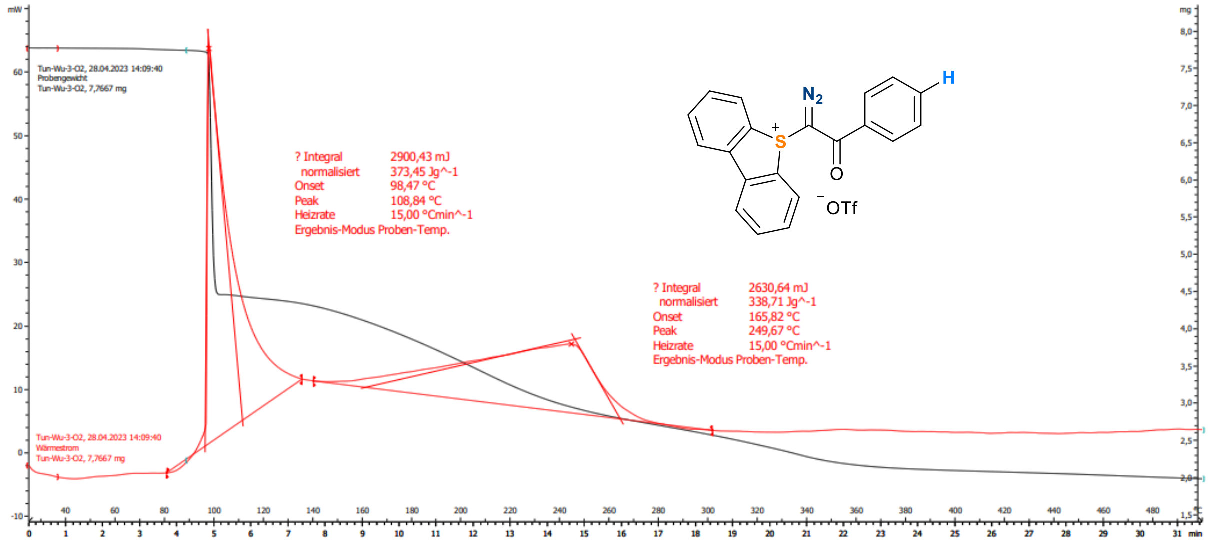

Figure S4: DSC measurement of compound **1b** under air.

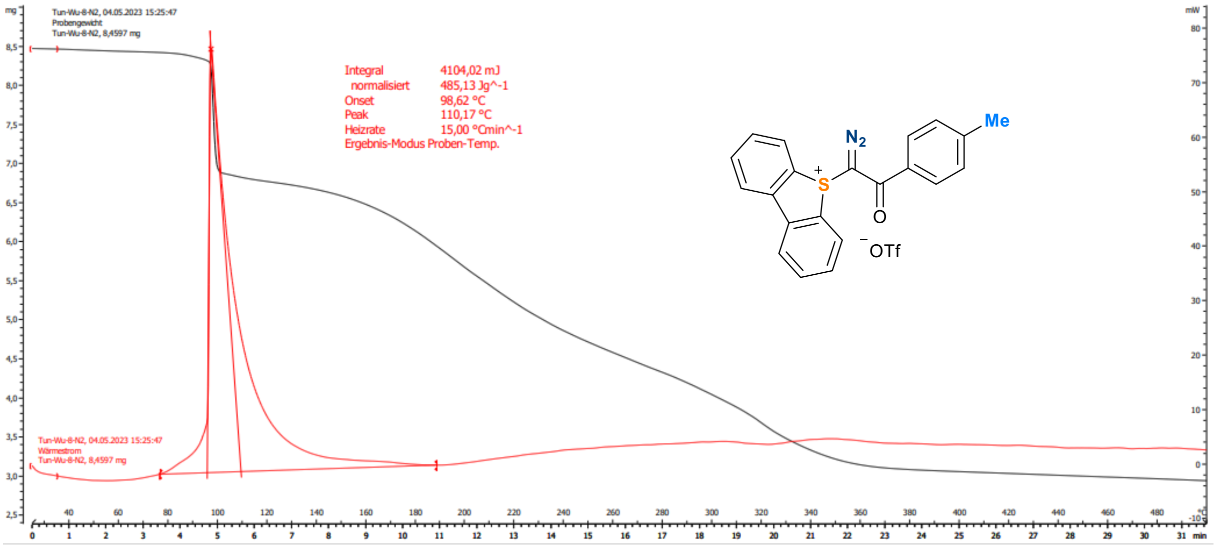

Figure S5: DSC measurement of compound **1c** under N_2.

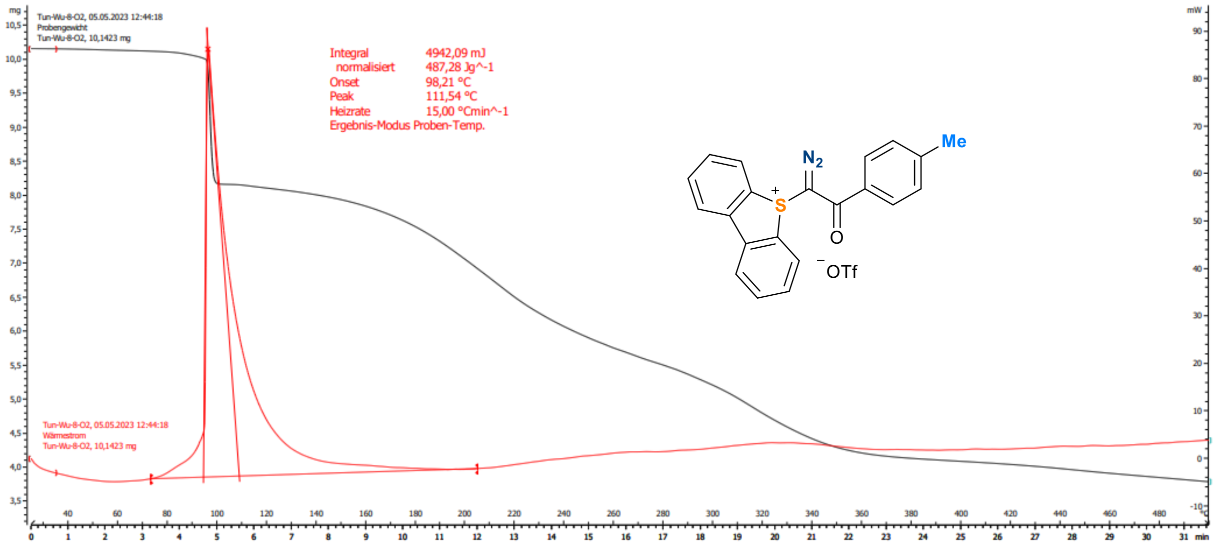

Figure S6: DSC measurement of compound **1c** under air.

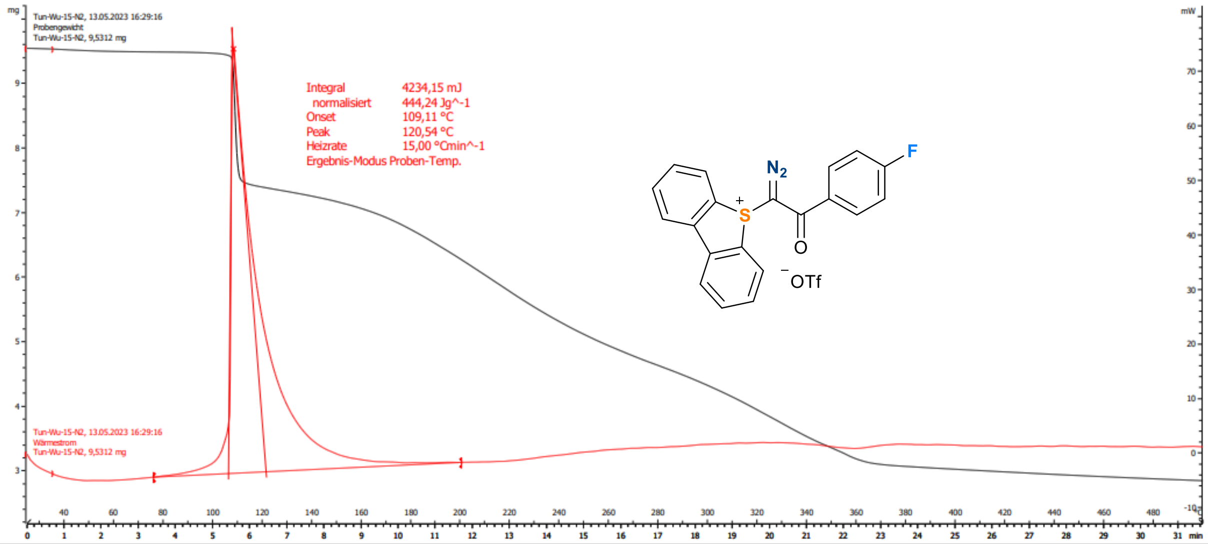

Figure S7: DSC measurement of compound **1d** under N_2.

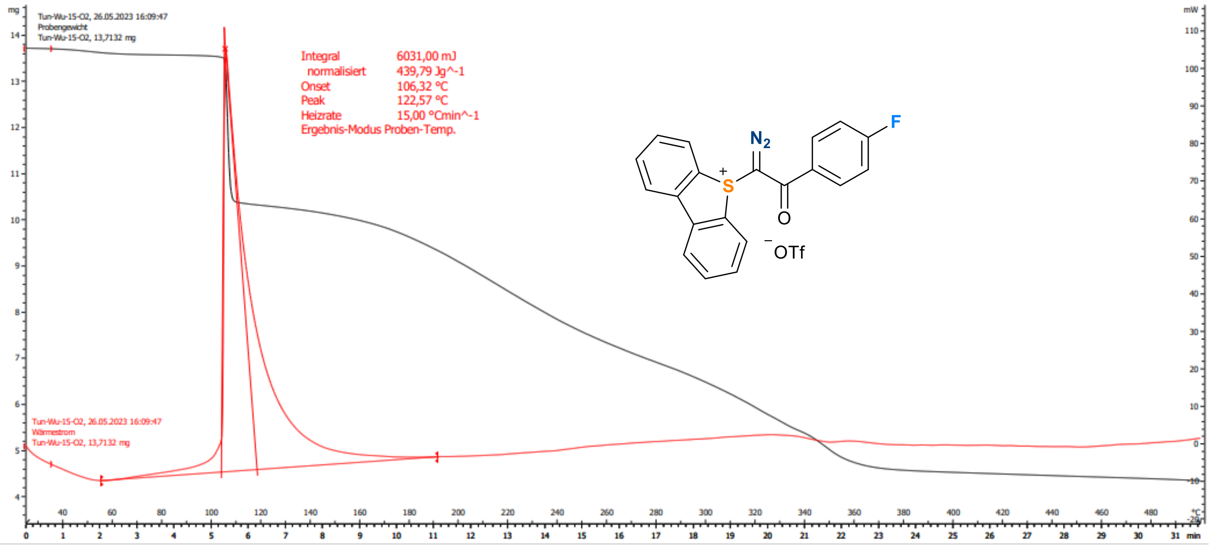

Figure S8: DSC measurement of compound **1d** under air.

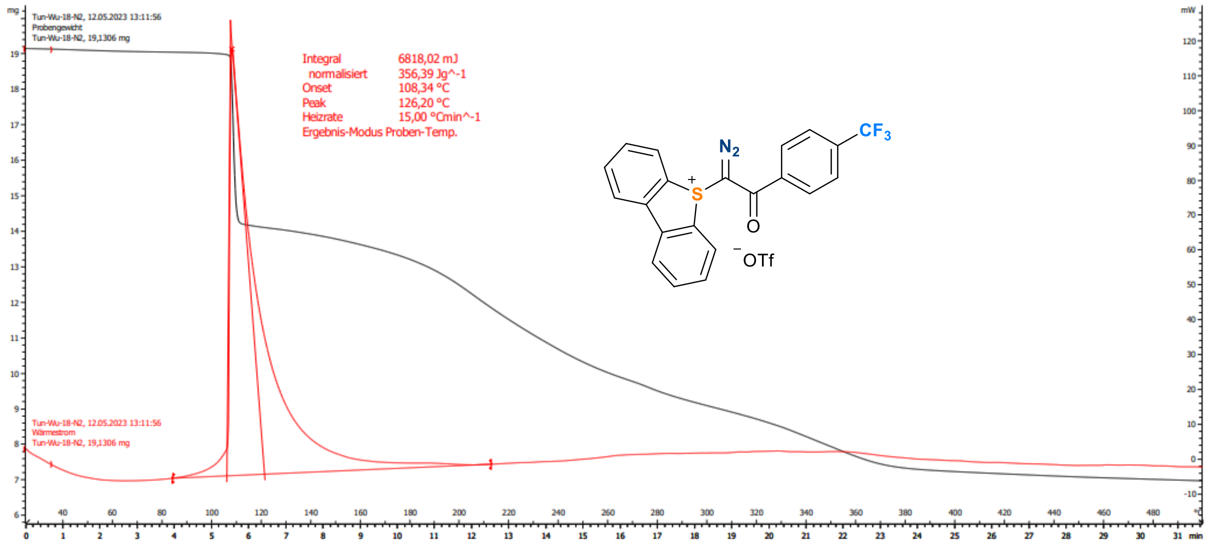

Figure S9: DSC measurement of compound **1e** under N_2.

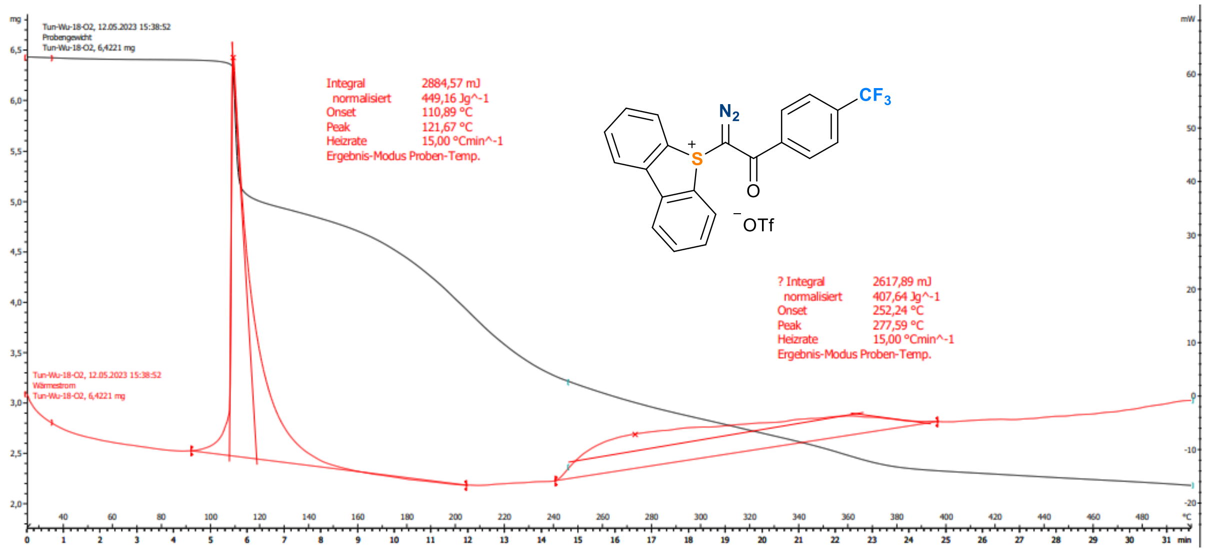

Figure S10: DSC measurement of compound **1e** under air.

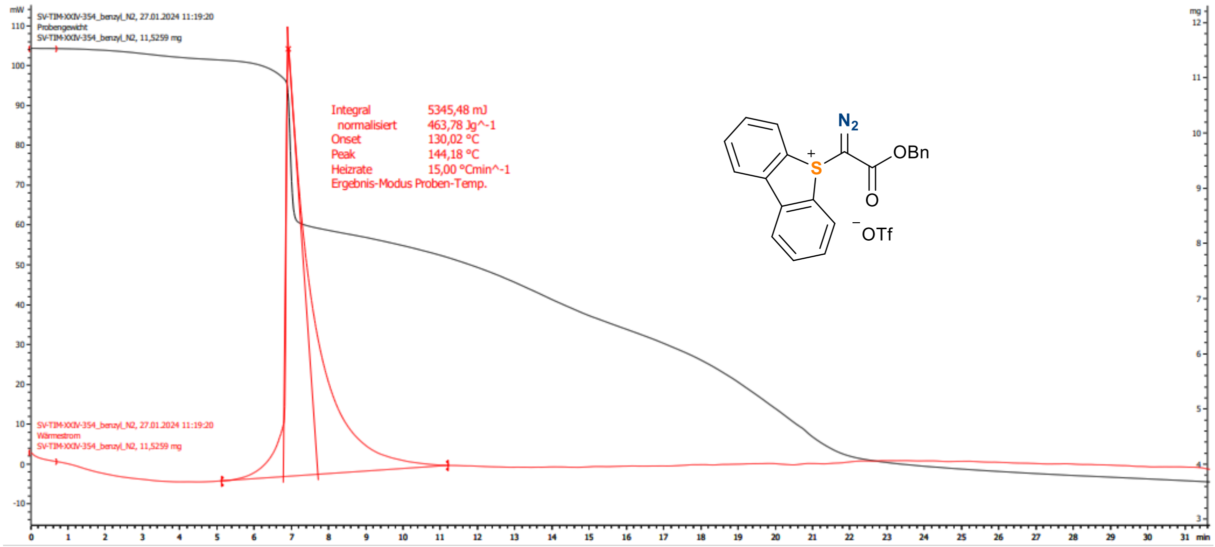

Figure S11: DSC measurement of compound **1f** under N_2.

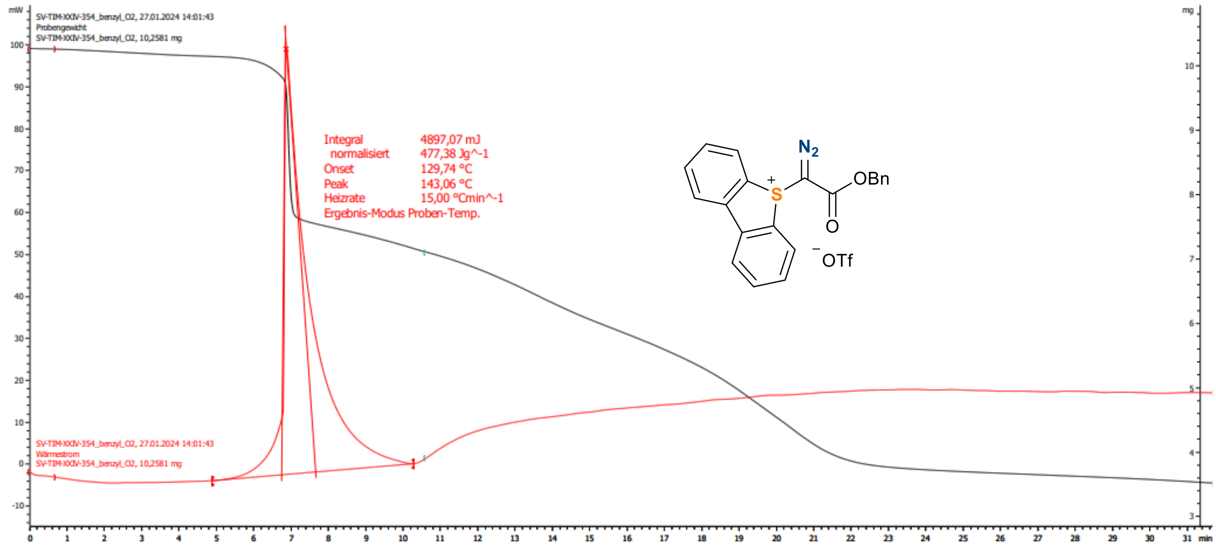

Figure S12: DSC measurement of compound **1f** under air.

X-RAY CRYSTALLOGRAPHIC ANALYSIS

General

The crystallization conditions are individually stated for each compound. The solvent vapor diffusion method refers to the methodology describes in the literature.[32]

This supplement contains in the following the refinement detail tables, a figure of the complete asymmetric unit and a picture of the crystal used for data collection. Further details can be obtained from the crystallographic information files (CIFs) uploaded to the *Cambridge Crystallographic Data Centre* (CCDC), where they can be obtained free of charge.

Identifier	CCDC number	Identifier	CCDC number
**1a**	2332867	**3h**	2332875
**1b**	2332868	**6b**	2332876
**1c**	2332869	**6c**	2332877
**1d**	2332870	**7c**	2332878
**3a**	2332871	**10f**	2332879
**3c**	2332872	**10i**	2332880
**3d**	2332873	**10t**	2332881
**3g**	2332874		

Refinement details

Compound 1a

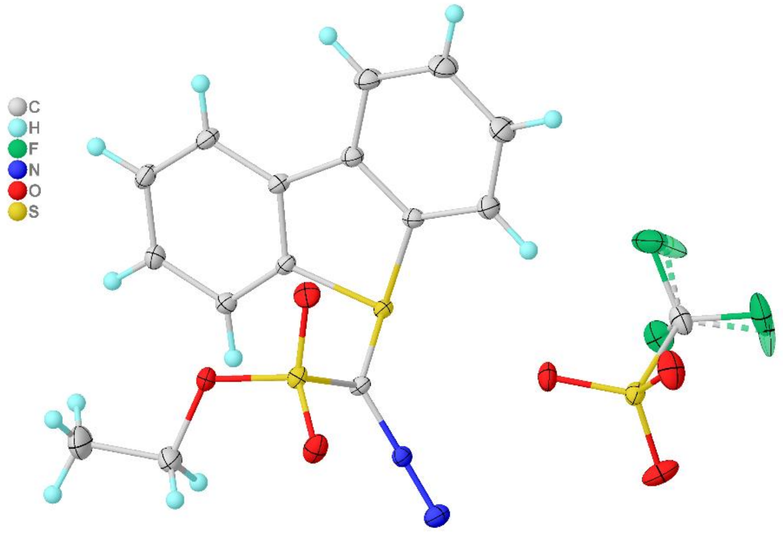

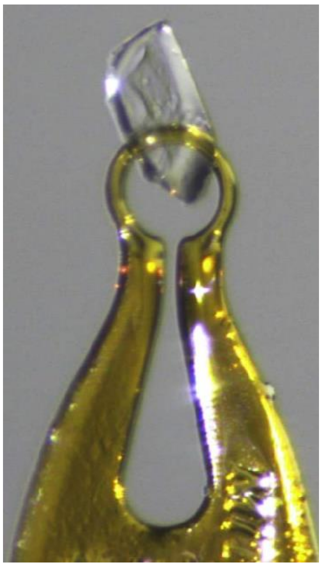

Figure S13: Full asymmetric unit of **1a**. Displacement ellipsoids are drawn at 50% probability level; minor positional disorder was found for the triflate anion, disordered model parts are drawn translucent with stippled bonds. Single crystals were obtained from a mixture of acetonitrile and diethylether by solvent vapor diffusion method.

CCDC number	2332867
Empirical formula	$C_{16}H_{13}F_3N_2O_6S_3$
Formula weight	482.46
Temperature [K]	100.00
Crystal system	Triclinic
Space group (number)	$P\bar{1}$ (2)
a [Å]	8.8939(5)
b [Å]	10.6953(7)
c [Å]	11.3020(8)
α [°]	109.349(2)
β [°]	98.738(2)
γ [°]	98.497(2)
Volume [Å^3]	979.70(11)
Z	2
ρ_{calc} [gcm^{-3}]	1.635
μ [mm^{-1}]	0.444
F(000)	492
Crystal size [mm^3]	0.37×0.179×0.088
Crystal color	Colorless
Crystal shape	Plate
Radiation	MoK_α (λ=0.71073 Å)

2θ range [°]	3.91 to 63.16 (0.68 Å)
Index ranges	−12 ≤ h ≤ 13<br>−15 ≤ k ≤ 15<br>−16 ≤ l ≤ 16
Reflections collected	50889
Independent reflections	6496<br>R_{int} = 0.0362<br>R_{sigma} = 0.0191
Completeness to θ = 25.242°	99.9 %
Data / Restraints / Parameters	6496/3/291
Goodness-of-fit on F^2	1.036
Final *R* indexes [*I*≥2σ(*I*)]	R_1 = 0.0279<br>wR_2 = 0.0789
Final *R* indexes [all data]	R_1 = 0.0298<br>wR_2 = 0.0806
Largest peak/hole [eÅ^{-3}]	0.54/-0.34

Compound 1b◦Et_2O,(MeCN)

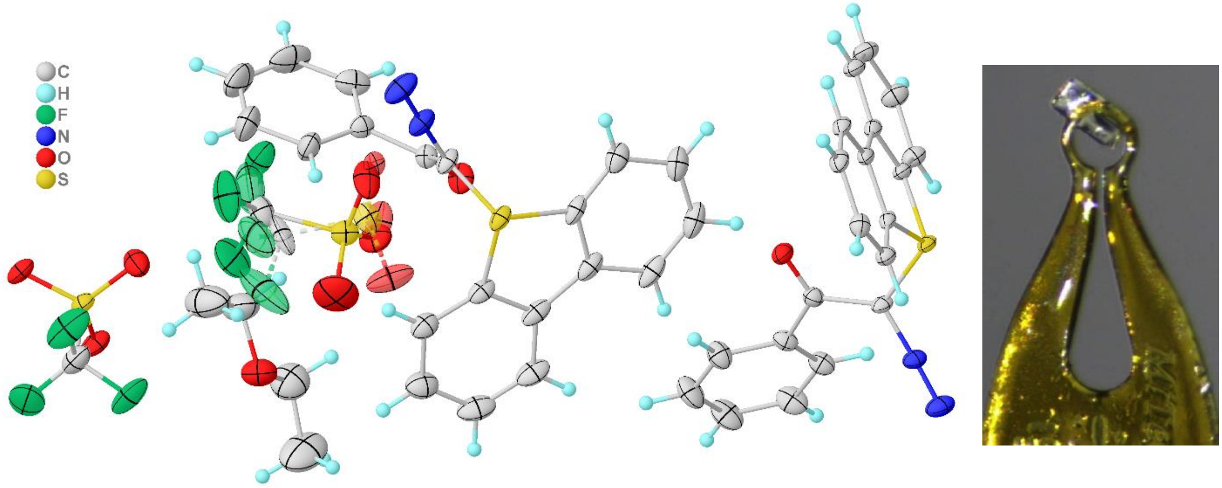

Figure S14: Full asymmetric unit of **1b**◦Et_2O,(MeCN), consisting of two crystallographically independent molecules of **1b** and one molecule of diethylether; a severely disordered molecule of acetonitrile was removed using the solvent mask plugin in OLEX[2]. Displacement ellipsoids are drawn at 50% probability level; minor positional disorder was found for the triflate anion, disordered model parts are drawn translucent with stippled bonds. Single crystals were obtained from a mixture of acetonitrile and diethylether by solvent vapor diffusion method.

CCDC number	2332868
Empirical formula	$C_{48}H_{39}F_6N_5O_9S_4$
Formula weight	1072.08
Temperature [K]	100.00
Crystal system	Triclinic
Space group (number)	$P\bar{1}$ (2)
a [Å]	10.5731(18)
b [Å]	13.509(2)
c [Å]	18.976(3)
α [°]	103.841(5)
β [°]	96.946(4)
γ [°]	107.996(5)
Volume [Å^3]	2446.6(7)
Z	2
ρ_{calc} [gcm^{-3}]	1.455
μ [mm^{-1}]	0.278
F(000)	1104
Crystal size [mm^3]	0.233×0.106×0.035
Crystal color	Colorless
Crystal shape	needle
Radiation	MoK_α (λ=0.71073 Å)

2θ range [°]	4.14 to 55.91 (0.76 Å)
Index ranges	−13 ≤ h ≤ 13<br>−17 ≤ k ≤ 17<br>−24 ≤ l ≤ 24
Reflections collected	92894
Independent reflections	11682<br>R_{int} = 0.0479<br>R_{sigma} = 0.0294
Completeness to θ = 25.242°	100.0 %
Data / Restraints / Parameters	11682/125/697
Goodness-of-fit on F^2	1.146
Final R indexes [I≥2σ(I)]	R_1 = 0.0449<br>wR_2 = 0.1169
Final R indexes [all data]	R_1 = 0.0578<br>wR_2 = 0.1230
Largest peak/hole [eÅ^{-3}]	0.47/-0.36

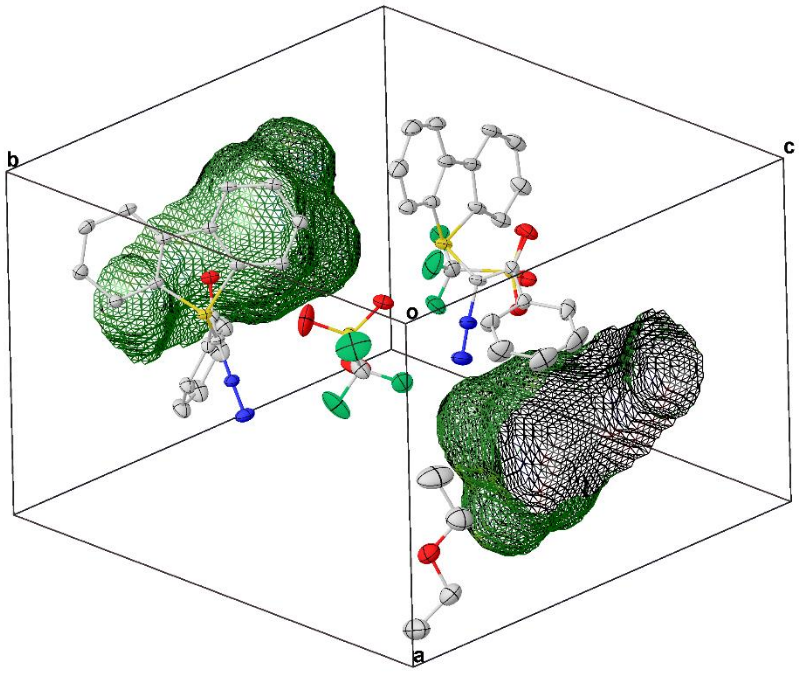

Figure S15: Depiction of the masked solvent cavity, containing a volume of 169.6 Å³ and 36 electrons, which is consistent with one formula unit of acetonitrile.

Compound 1c◦2 DCM

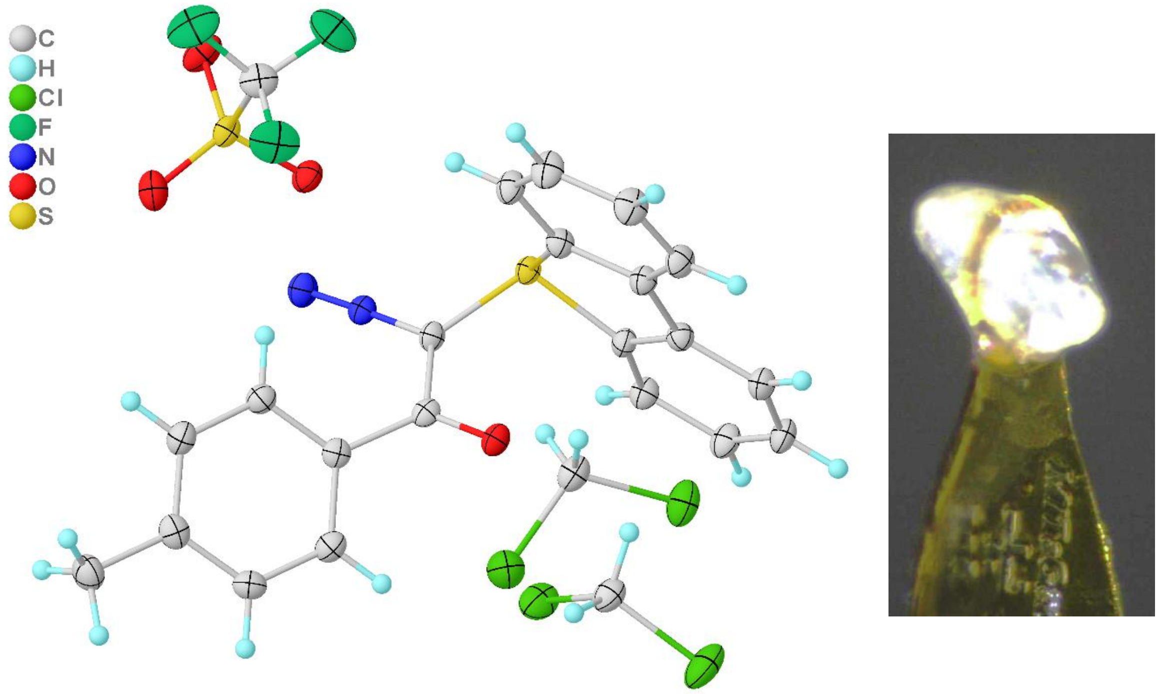

Figure S16: Full asymmetric unit of **1c**◦2 DCM. Displacement ellipsoids are drawn at 50% probability level. Single crystals were obtained by evaporation of a solution in dichloromethane.

CCDC number	2332869
Empirical formula	$C_{24}H_{19}Cl_4F_3N_2O_4S_2$
Formula weight	662.33
Temperature [K]	100.00
Crystal system	Triclinic
Space group (number)	$P\bar{1}$ (2)
a [Å]	11.2625(15)
b [Å]	11.5958(16)
c [Å]	12.8133(18)
α [°]	108.624(4)
β [°]	94.050(4)
γ [°]	114.412(4)
Volume [$Å^3$]	1403.6(3)
Z	2
ρ_{calc} [gcm^{-3}]	1.567
μ [mm^{-1}]	0.625
F(000)	672
Crystal size [mm^3]	0.614×0.381×0.268
Crystal color	Colorless
Crystal shape	Block
Radiation	MoK_α (λ=0.71073 Å)

2θ range [°]	4.09 to 55.91 (0.76 Å)
Index ranges	−14 ≤ h ≤ 14<br>−15 ≤ k ≤ 15<br>−16 ≤ l ≤ 16
Reflections collected	56271
Independent reflections	6707<br>R_{int} = 0.0464<br>R_{sigma} = 0.0209
Completeness to θ = 25.242°	99.6 %
Data / Restraints / Parameters	6707/0/353
Goodness-of-fit on F^2	1.043
Final *R* indexes [$I \geq 2\sigma(I)$]	R_1 = 0.0387<br>wR_2 = 0.1094
Final *R* indexes [all data]	R_1 = 0.0421<br>wR_2 = 0.1132
Largest peak/hole [$eÅ^{-3}$]	0.62/-0.50

Compound 1d◦Et₂O

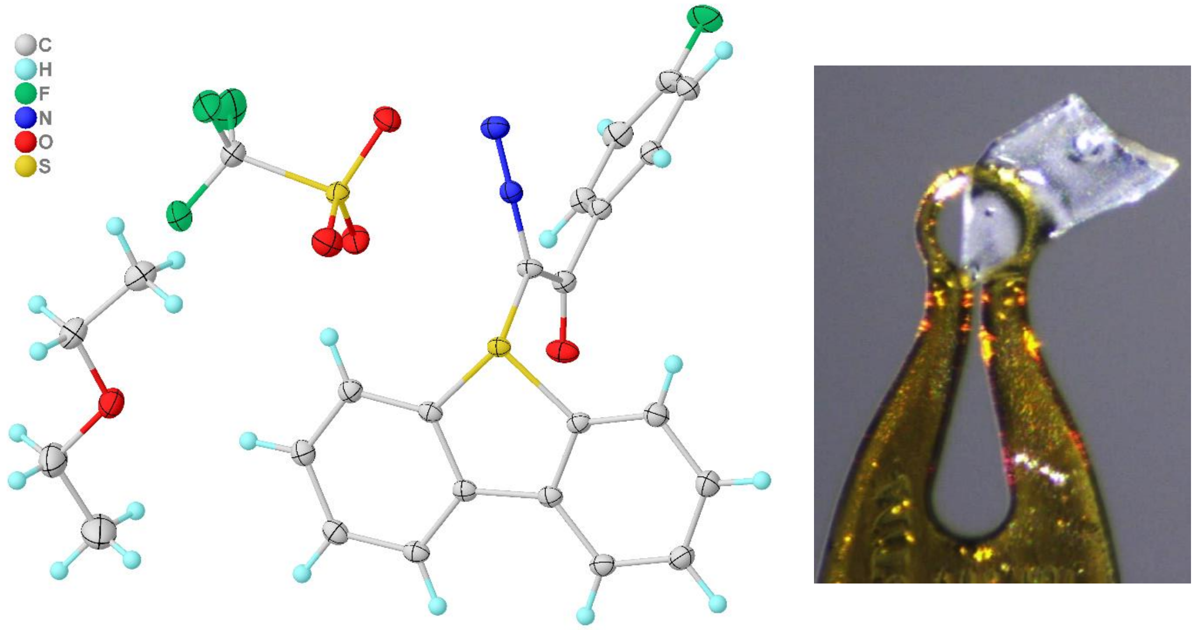

Figure S17: Full asymmetric unit of **1d**◦Et_2O. Displacement ellipsoids are drawn at 50% probability level. Single crystals were obtained from a mixture of acetonitrile and diethylether by solvent vapor diffusion method.

CCDC number	2332870
Empirical formula	$C_{25}H_{22}F_4N_2O_5S_2$
Formula weight	570.56
Temperature [K]	100.00
Crystal system	Triclinic
Space group (number)	$P\bar{1}$ (2)
a [Å]	10.6503(16)
b [Å]	11.1577(17)
c [Å]	12.0098(18)
α [°]	109.986(4)
β [°]	98.765(4)
γ [°]	97.218(5)
Volume [$Å^3$]	1301.1(3)
Z	2
ρ_{calc} [gcm^{-3}]	1.456
μ [mm^{-1}]	0.273
F(000)	588
Crystal size [mm^3]	0.576×0.249×0.064
Crystal color	Colorless
Crystal shape	Block
Radiation	MoK_α (λ=0.71073 Å)

2θ range [°]	3.94 to 61.33 (0.70 Å)
Index ranges	−15 ≤ h ≤ 15<br>−15 ≤ k ≤ 15<br>−17 ≤ l ≤ 17
Reflections collected	66027
Independent reflections	8010<br>R_{int} = 0.0426<br>R_{sigma} = 0.0212
Completeness to θ = 25.242°	100.0 %
Data / Restraints / Parameters	8010/0/345
Goodness-of-fit on F^2	1.036
Final *R* indexes [$I \geq 2\sigma(I)$]	R_1 = 0.0332<br>wR_2 = 0.0901
Final *R* indexes [all data]	R_1 = 0.0368<br>wR_2 = 0.0934
Largest peak/hole [$eÅ^{-3}$]	0.51/-0.33

Compound 3a

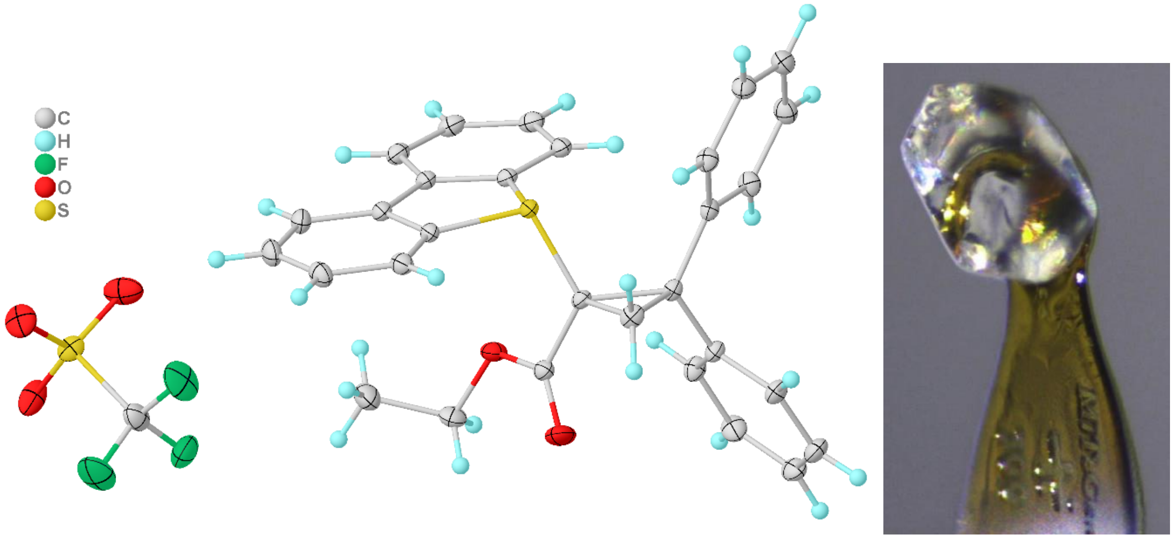

Figure S18: Full asymmetric unit of **3a**. Displacement ellipsoids are drawn at 50% probability level. Single crystals were obtained from a mixture of acetonitrile and diethylether by solvent vapor diffusion method.

CCDC number	2332871
Empirical formula	$C_{31}H_{25}F_3O_5S_2$
Formula weight	598.63
Temperature [K]	100.00
Crystal system	Monoclinic
Space group (number)	$P2_1/c$ (14)
a [Å]	12.1265(5)
b [Å]	11.7452(5)
c [Å]	19.7744(9)
α [°]	90
β [°]	106.223(2)
γ [°]	90
Volume [Å^3]	2704.3(2)
Z	4
ρ_{calc} [gcm^{-3}]	1.470
μ [mm^{-1}]	0.259
F(000)	1240
Crystal size [mm^3]	0.568×0.484×0.264
Crystal color	Colorless
Crystal shape	Block
Radiation	MoK_α (λ=0.71073 Å)
2θ range [°]	4.29 to 65.27 (0.66 Å)

Index ranges	−18 ≤ h ≤ 18<br>−17 ≤ k ≤ 17<br>−29 ≤ l ≤ 29
Reflections collected	172291
Independent reflections	9833<br>R_{int} = 0.0390<br>R_{sigma} = 0.0134
Completeness to θ = 25.242°	100.0 %
Data / Restraints / Parameters	9833/2/377
Absorption correction T_{min}/T_{max} (method)	0.7888/1.0000 (numerical)
Goodness-of-fit on F^2	1.057
Final *R* indexes [I≥2σ(I)]	R_1 = 0.0341<br>wR_2 = 0.0950
Final *R* indexes [all data]	R_1 = 0.0360<br>wR_2 = 0.0966
Largest peak/hole [eÅ^{-3}]	0.53/−0.50

Compound 3c◦Et_2O

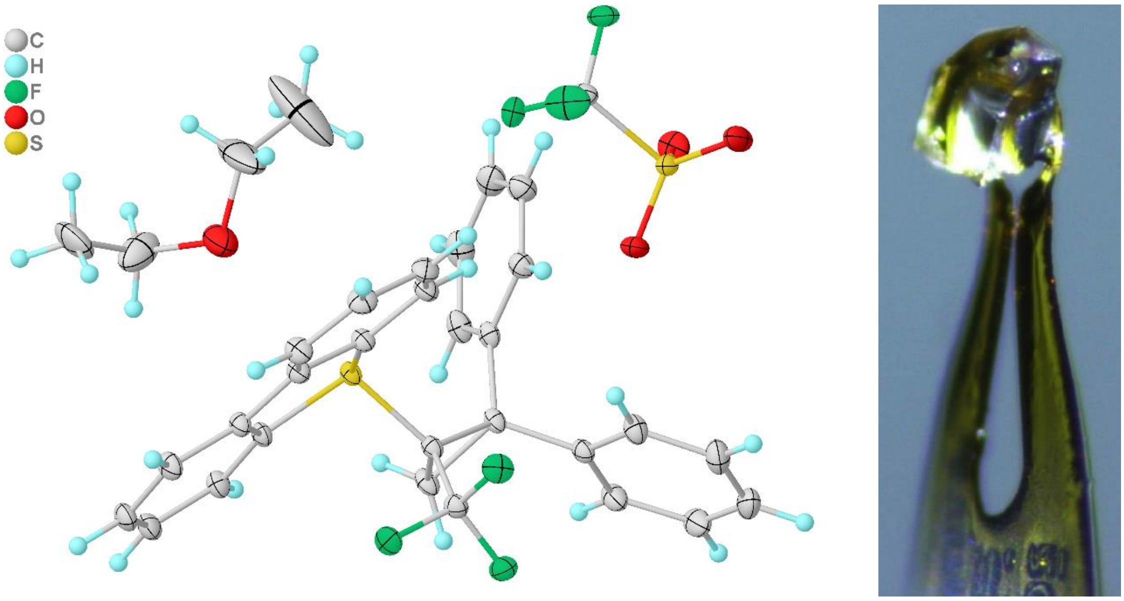

Figure S19: Full asymmetric unit of **3c**◦Et_2O. Displacement ellipsoids are drawn at 50% probability level. Single crystals were obtained from a mixture of acetonitrile and diethylether by solvent vapor diffusion method.

CCDC number	2332872
Empirical formula	$C_{31}H_{25}F_6O_{3.50}S_2$
Formula weight	631.63
Temperature [K]	100.00
Crystal system	Monoclinic
Space group (number)	$P2_1/n$ (14)
a [Å]	11.2326(6)
b [Å]	18.0655(9)
c [Å]	14.1136(4)
α [°]	90
β [°]	101.8440(10)
γ [°]	90
Volume [Å^3]	2803.0(2)
Z	4
ρ_{calc} [gcm^{-3}]	1.497
μ [mm^{-1}]	0.265
F(000)	1300
Crystal size [mm^3]	0.297×0.235×0.122
Crystal color	Colorless
Crystal shape	Block
Radiation	MoK_α (λ=0.71073 Å)
2θ range [°]	4.24 to 61.03 (0.70 Å)

Index ranges	−16 ≤ h ≤ 16<br>−24 ≤ k ≤ 25<br>−20 ≤ l ≤ 14
Reflections collected	51609
Independent reflections	8513<br>R_{int} = 0.0239<br>R_{sigma} = 0.0134
Completeness to θ = 25.242°	100.0 %
Data / Restraints / Parameters	8513/27/408
Absorption correction T_{min}/T_{max} (method)	0.9335/0.9786 (multi-scan)
Goodness-of-fit on F^2	1.038
Final *R* indexes [I≥2σ(I)]	R_1 = 0.0343<br>wR_2 = 0.0893
Final *R* indexes [all data]	R_1 = 0.0375<br>wR_2 = 0.0919
Largest peak/hole [eÅ^{-3}]	0.75/−0.66

Compound 3d◦MeCN

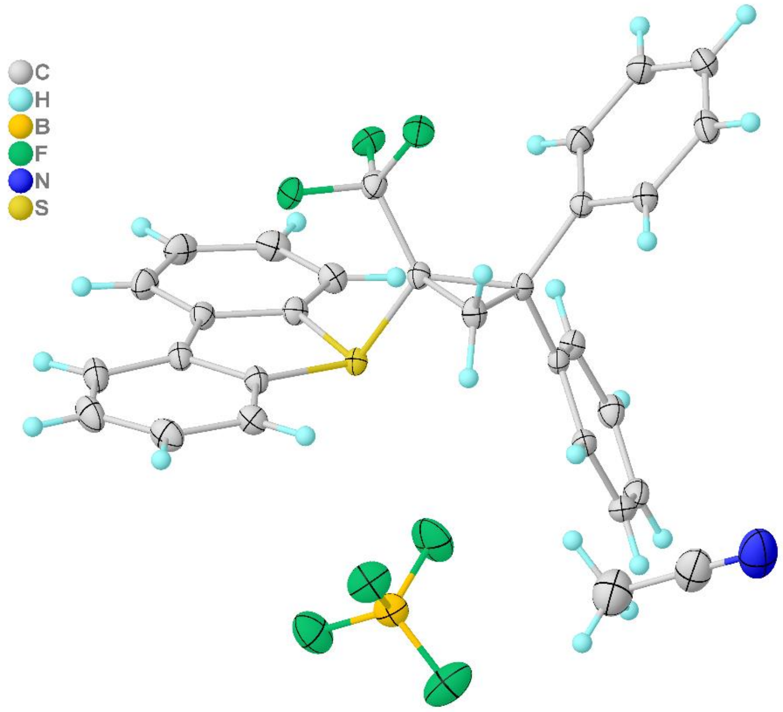

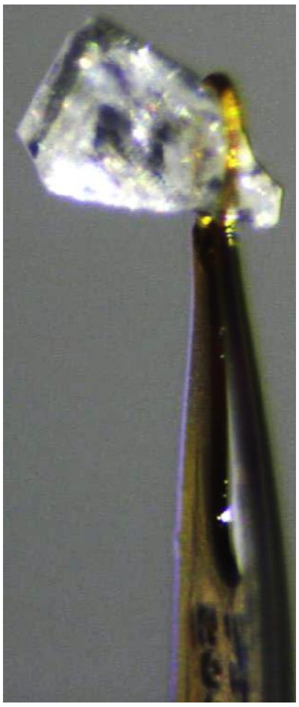

Figure S20: Full asymmetric unit of **3d**◦MeCN. Displacement ellipsoids are drawn at 50% probability level. Single crystals were obtained from a mixture of acetonitrile and diethylether by solvent vapor diffusion method.

CCDC number	2332873
Empirical formula	$C_{30}H_{23}BF_7NS$
Formula weight	573.36
Temperature [K]	100.00
Crystal system	Triclinic
Space group (number)	$P\bar{1}$ (2)
a [Å]	9.7055(6)
b [Å]	11.9141(9)
c [Å]	12.9664(10)
α [°]	79.460(2)
β [°]	68.373(2)
γ [°]	70.766(2)
Volume [Å^3]	1312.75(17)
Z	2
ρ_{calc} [gcm^{-3}]	1.451
μ [mm^{-1}]	0.195
F(000)	588
Crystal size [mm^3]	0.476×0.281×0.154
Crystal color	Colorless
Crystal shape	Block
Radiation	MoK_α (λ=0.71073 Å)

2θ range [°]	4.71 to 61.18 (0.70 Å)
Index ranges	−12 ≤ h ≤ 13<br>−17 ≤ k ≤ 17<br>−18 ≤ l ≤ 18
Reflections collected	76784
Independent reflections	8052<br>R_{int} = 0.0363<br>R_{sigma} = 0.0167
Completeness to θ = 25.242°	100.0 %
Data / Restraints / Parameters	8052/0/362
Goodness-of-fit on F^2	1.046
Final *R* indexes [I≥2σ(I)]	R_1 = 0.0399<br>wR_2 = 0.1085
Final *R* indexes [all data]	R_1 = 0.0434<br>wR_2 = 0.1116
Largest peak/hole [eÅ^{-3}]	0.85/-0.37

Compound 3g

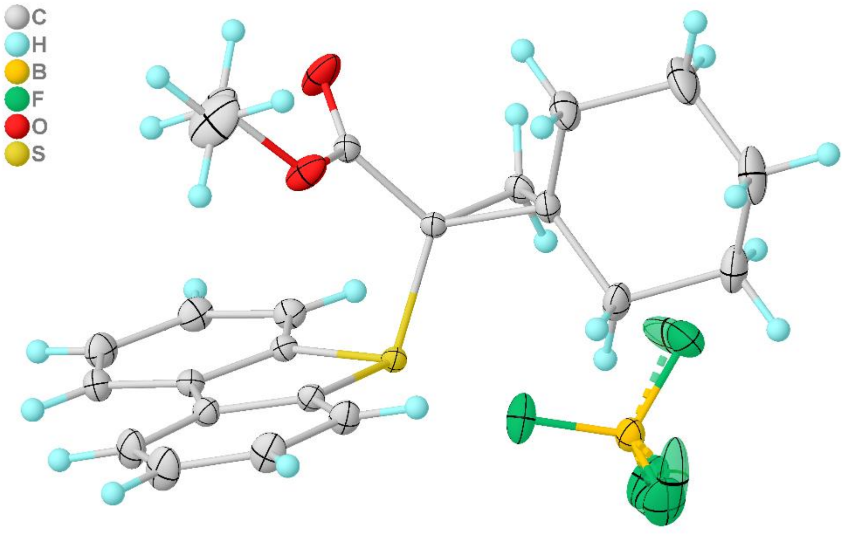

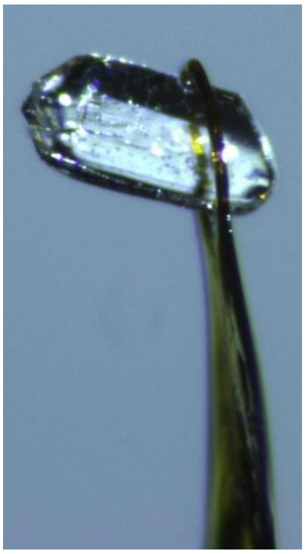

Figure S21: Full asymmetric unit of **3g**. Displacement ellipsoids are drawn at 50% probability level; minor positional disorder was found for the tetrafluoroborate anion, disordered model parts are drawn translucent with stippled bonds. Single crystals were obtained from a mixture of dichloromethane and diethylether by solvent vapor diffusion method.

CCDC number	2332874
Empirical formula	$C_{23}H_{25}BF_4O_2S$
Formula weight	452.30
Temperature [K]	100.00
Crystal system	Monoclinic
Space group (number)	$P2_1/c$ (14)
a [Å]	10.7059(7)
b [Å]	16.8690(7)
c [Å]	12.6192(8)
α [°]	90
β [°]	106.941(2)
γ [°]	90
Volume [Å^3]	2180.1(2)
Z	4
ρ_{calc} [gcm^{-3}]	1.378
μ [mm^{-1}]	0.200
F(000)	944
Crystal size [mm^3]	0.597×0.27×0.222
Crystal color	colorless
Crystal shape	Block
Radiation	MoK_α (λ=0.71073 Å)
2θ range [°]	4.15 to 65.18 (0.66 Å)

Index ranges	−16 ≤ h ≤ 16<br>−17 ≤ k ≤ 25<br>−19 ≤ l ≤ 19
Reflections collected	71540
Independent reflections	7837<br>R_{int} = 0.0210<br>R_{sigma} = 0.0098
Completeness to θ = 25.242°	99.8 %
Data / Restraints / Parameters	7837/3/309
Absorption correction T_{min}/T_{max} (method)	0.9185/0.9811 (numerical)
Goodness-of-fit on F^2	1.031
Final *R* indexes [$I \geq 2\sigma(I)$]	R_1 = 0.0296<br>wR_2 = 0.0823
Final *R* indexes [all data]	R_1 = 0.0311<br>wR_2 = 0.0837
Largest peak/hole [eÅ^{-3}]	0.49/−0.28

Compound 3h

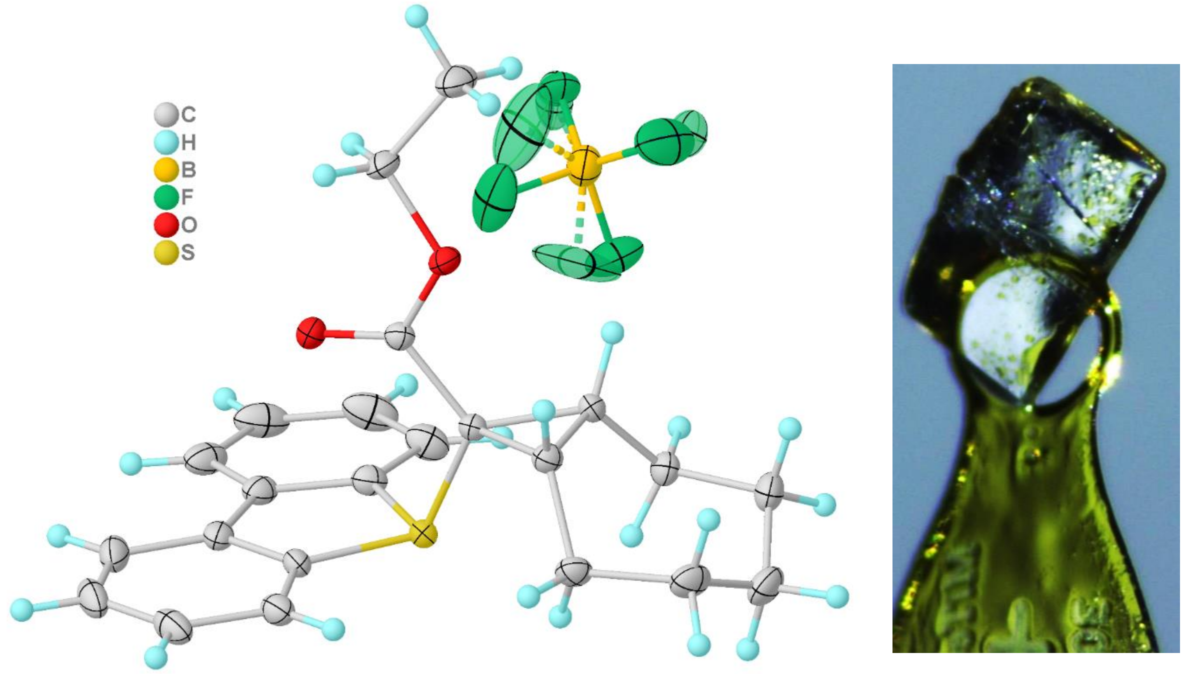

Figure S22: Full asymmetric unit of **3h**. Displacement ellipsoids are drawn at 50% probability level; minor positional disorder was found for the tetrafluoroborate anion, disordered model parts are drawn translucent with stippled bonds. Crystals suitable for diffraction were obtained from a mixture of dichloromethane and diethylether by solvent vapor diffusion method. The material was severely intergrown and two minor domains could be indexed in the crystal used for data collection. The two minor domains are related to the major by (1.002 0.014 -0.024 / -0.006 0.997 0.009 / 0.100 -0.028 0.999) and (0.994 -0.007 0.028 / -0.023 0.968 0.066 / -0.106 -0.184 1.024) respectively, corresponding to rotation by -3.0 ° and -6.8 °, respectively. For the refinement, all domains were used and the final batch scale factors were 0.122(2) and 0.0183(14).

CCDC number	2332875
Empirical formula	$C_{23}H_{25}BF_4O_2S$
Formula weight	452.30
Temperature [K]	100.00
Crystal system	Triclinic
Space group (number)	$P\bar{1}$ (2)
a [Å]	7.4771(6)
b [Å]	9.2628(8)
c [Å]	15.6995(14)
α [°]	75.560(3)
β [°]	84.910(4)
γ [°]	85.280(5)
Volume [Å^3]	1046.79(16)
Z	2
ρ_{calc} [gcm^{-3}]	1.435
μ [mm^{-1}]	0.208
F(000)	472
Crystal size [mm^3]	0.634×0.367×0.098
Crystal color	Colorless
Crystal shape	Plate

Radiation	MoK_{α} (λ=0.71073 Å)
2θ range [°]	4.55 to 59.21 (0.72 Å)
Index ranges	−9 ≤ h ≤ 10<br>−12 ≤ k ≤ 12<br>−21 ≤ l ≤ 21
Reflections collected	10696
Independent reflections	10696<br>R_{int} = 0.0557<br>R_{sigma} = 0.0353
Completeness to θ = 25.242°	99.6 %
Data / Restraints / Parameters	10696/64/320
Absorption correction T_{min}/T_{max} (method)	0.603714/0.735311 (multi-scan)
Goodness-of-fit on F^2	1.058

Final *R* indexes [$I \geq 2\sigma(I)$]	R_1 = 0.0394<br>wR_2 = 0.1007
Final *R* indexes [all data]	R_1 = 0.0447<br>wR_2 = 0.1053
Largest peak/hole [eÅ^{-3}]	0.34/−0.42

Compound 6b

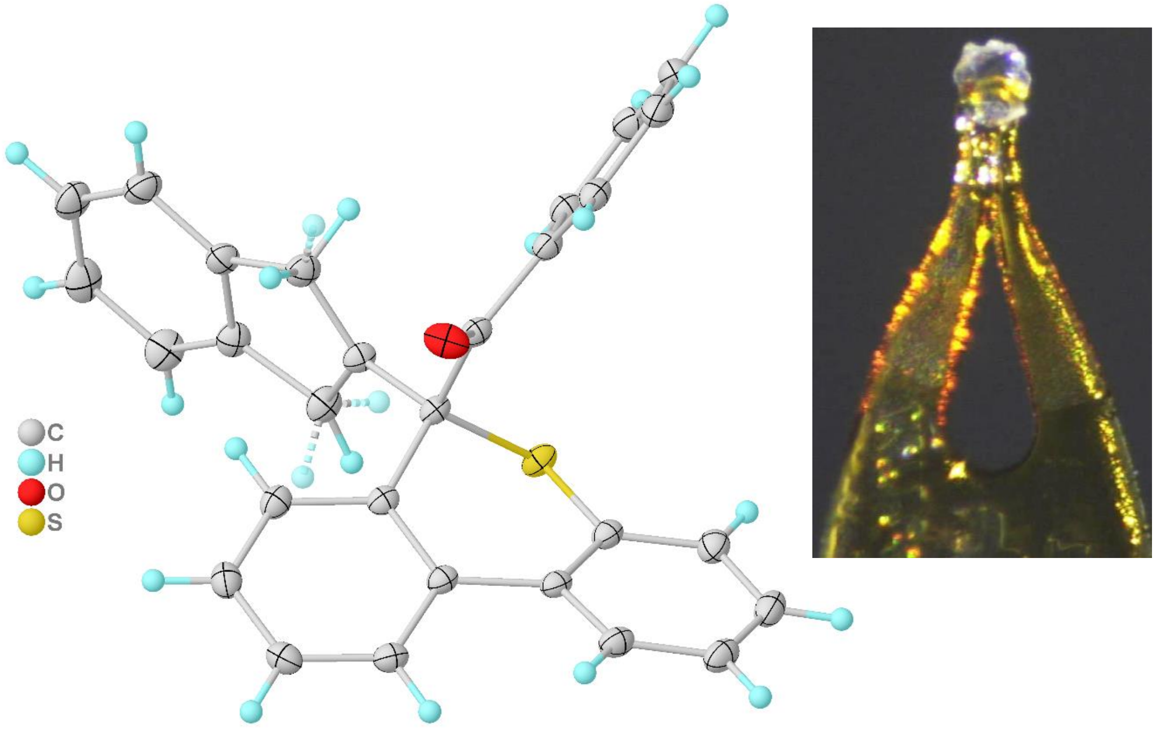

Figure S23: Full asymmetric unit of **6b**. Displacement ellipsoids are drawn at 50% probability level; the indene fragment is disordered, with the CH and CH_2 positions flipping; minor disorder model parts are drawn translucent with stippled bonds. Single crystals were obtained by evaporation from a solution in diethylether. Non-merohedral twinning was found during unit cell indexing, with the two domains related by the twin law (+0.971 -0.044 -0.031 / +0.029 +1.008 -0.008 / +0.091 +0.063 +1.017) corresponding to a rotation of 3.6 °. Twin integration afforded satisfying data quality and refinement was carried out against hklf5 with a final batch scale factor of 0.200(4).

CCDC number	2332876
Empirical formula	$C_{29}H_{20}OS$
Formula weight	416.51
Temperature [K]	100.00
Crystal system	Triclinic
Space group (number)	$P\bar{1}$ (2)
a [Å]	8.945(2)
b [Å]	9.215(3)
c [Å]	14.225(4)
α [°]	97.773(8)
β [°]	102.840(8)
γ [°]	111.165(8)
Volume [Å^3]	1035.8(5)
Z	2
ρ_{calc} [gcm^{-3}]	1.335
μ [mm^{-1}]	0.176
F(000)	436
Crystal size [mm^3]	0.164×0.122×0.088
Crystal color	Colorless
Crystal shape	Block

Radiation	MoK_α (λ=0.71073 Å)
2θ range [°]	4.88 to 61.10 (0.70 Å)
Index ranges	$-8 \le h \le 12$ $-13 \le k \le 13$ $-20 \le l \le 20$
Reflections collected	10570
Independent reflections	10570 R_{int} = 0.0493 R_{sigma} = 0.0362
Completeness to θ = 25.242°	99.9 %
Data / Restraints / Parameters	10570/0/282
Goodness-of-fit on F^2	1.089
Final R indexes [$I \geq 2\sigma(I)$]	R_1 = 0.0426 wR_2 = 0.1143
Final R indexes [all data]	R_1 = 0.0498 wR_2 = 0.1197
Largest peak/hole [eÅ^{-3}]	0.46/-0.26

Compound 6c

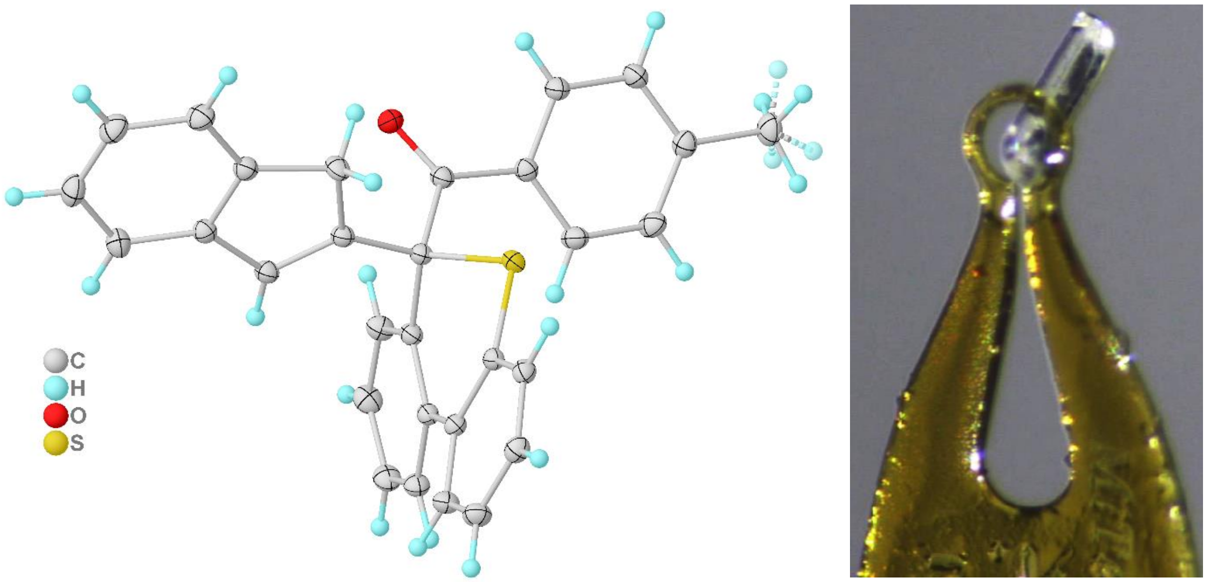

Figure S24: Full asymmetric unit of **6c**. Displacement ellipsoids are drawn at 50% probability level; minor positional disorder was found for the methyl groups, the minor disordered model part is drawn translucent with stippled bonds. Single crystals were obtained by evaporation from a solution in diethylether.

CCDC number	2332877
Empirical formula	$C_{30}H_{22}OS$
Formula weight	430.53
Temperature [K]	100.00
Crystal system	Triclinic
Space group (number)	$P\bar{1}$ (2)
a [Å]	9.4954(8)
b [Å]	11.2014(12)
c [Å]	11.5541(11)
α [°]	72.592(3)
β [°]	87.789(3)
γ [°]	68.141(3)
Volume [Å^3]	1084.64(18)
Z	2
ρ_{calc} [gcm^{-3}]	1.318
μ [mm^{-1}]	0.170
F(000)	452
Crystal size [mm^3]	0.403×0.104×0.104
Crystal color	Colorless
Crystal shape	Plank
Radiation	MoK_α (λ=0.71073 Å)

2θ range [°]	3.71 to 63.20 (0.68 Å)
Index ranges	$-13 \leq h \leq 13$<br>$-16 \leq k \leq 16$<br>$-17 \leq l \leq 16$
Reflections collected	75111
Independent reflections	7250<br>R_{int} = 0.0451<br>R_{sigma} = 0.0205
Completeness to θ = 25.242°	100.0 %
Data / Restraints / Parameters	7250/0/291
Goodness-of-fit on F^2	1.037
Final *R* indexes [$I \geq 2\sigma(I)$]	R_1 = 0.0378<br>wR_2 = 0.1017
Final *R* indexes [all data]	R_1 = 0.0427<br>wR_2 = 0.1061
Largest peak/hole [eÅ^{-3}]	0.53/-0.24

Compound 7c

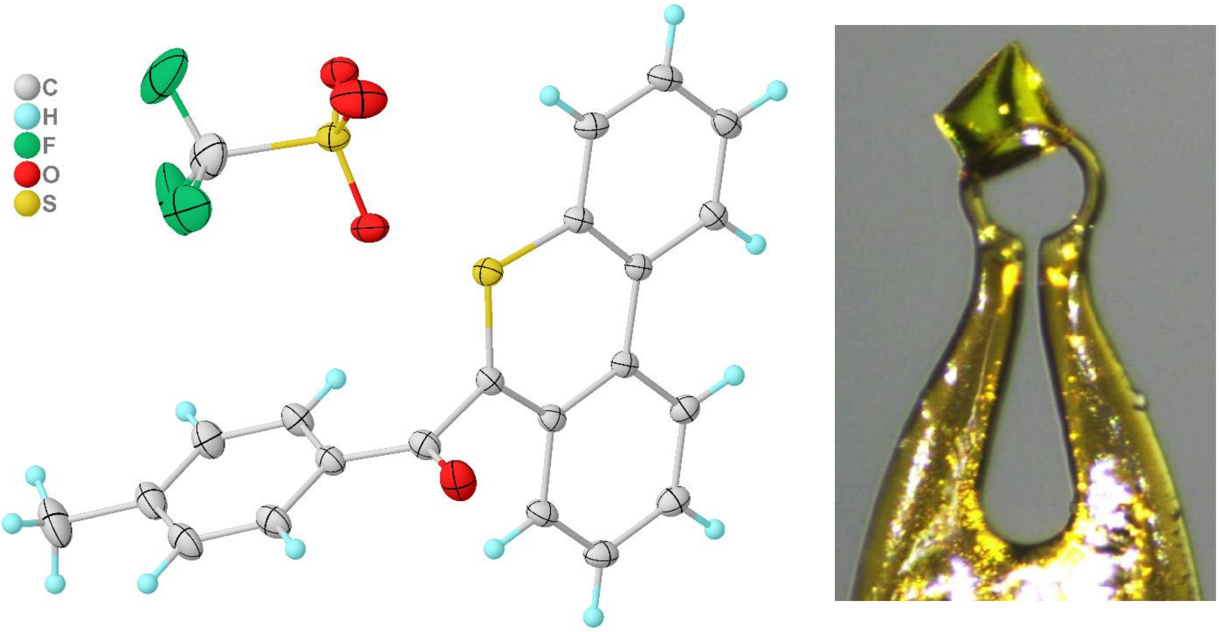

Figure S25: Full asymmetric unit of **7c**. Displacement ellipsoids are drawn at 50% probability level. Single crystals were obtained by evaporation from a solution in diethylether.

CCDC number	2332878
Empirical formula	$C_{22}H_{15}F_3O_4S_2$
Formula weight	464.46
Temperature [K]	100.00
Crystal system	Triclinic
Space group (number)	$P\bar{1}$ (2)
a [Å]	6.8498(16)
b [Å]	9.930(2)
c [Å]	15.949(4)
α [°]	78.205(8)
β [°]	88.999(10)
γ [°]	81.864(11)
Volume [Å^3]	1051.2(4)
Z	2
ρ_{calc} [gcm^{-3}]	1.467
μ [mm^{-1}]	0.306
F(000)	476
Crystal size [mm^3]	0.222×0.215×0.06
Crystal color	Yellow
Crystal shape	Plate
Radiation	MoK_α (λ=0.71073 Å)

2θ range [°]	4.23 to 61.03 (0.70 Å)
Index ranges	−9 ≤ h ≤ 9<br>−14 ≤ k ≤ 14<br>−22 ≤ l ≤ 22
Reflections collected	84131
Independent reflections	6400<br>R_{int} = 0.0469<br>R_{sigma} = 0.0202
Completeness to θ = 25.242°	100.0 %
Data / Restraints / Parameters	6400/0/281
Goodness-of-fit on F^2	1.026
Final *R* indexes [$I \geq 2\sigma(I)$]	R_1 = 0.0399<br>wR_2 = 0.0982
Final *R* indexes [all data]	R_1 = 0.0479<br>wR_2 = 0.1042
Largest peak/hole [eÅ^{-3}]	0.43/-0.41

Compound 10f

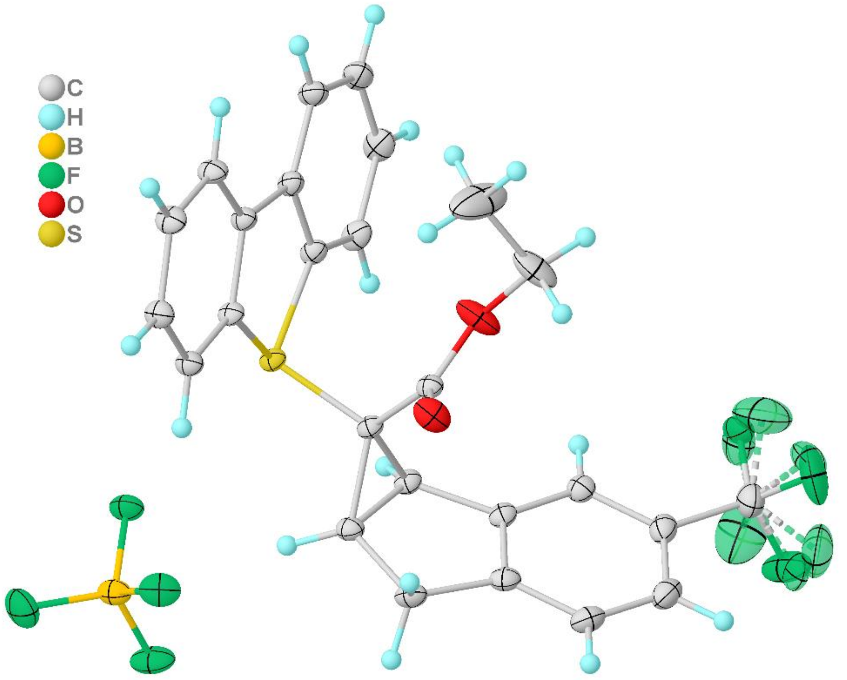

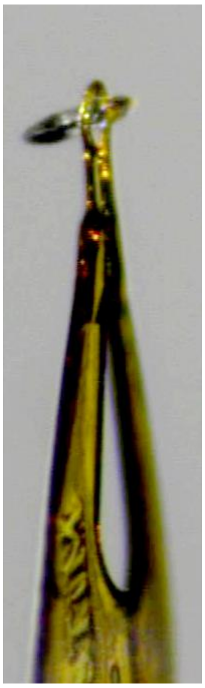

Figure S26: Full asymmetric unit of **10f**. Displacement ellipsoids are drawn at 50% probability level. The CF_3 group exhibits rotational disorder and is split in three positions; the minor disordered model part is drawn translucent with stippled bonds. Single crystals were obtained from a mixture of acetonitrile and diethylether by solvent vapor diffusion method.

CCDC number	2332879
Empirical formula	$C_{26}H_{20}BF_7O_2S$
Formula weight	540.29
Temperature [K]	100.00
Crystal system	Triclinic
Space group (number)	$P\bar{1}$ (2)
a [Å]	8.946(2)
b [Å]	10.892(3)
c [Å]	12.669(4)
α [°]	95.044(7)
β [°]	109.263(11)
γ [°]	93.731(9)
Volume [Å^3]	1155.1(5)
Z	2
ρ_{calc} [gcm^{-3}]	1.553
μ [mm^{-1}]	0.221
F(000)	552
Crystal size [mm^3]	0.223×0.13×0.039
Crystal color	Colorless
Crystal shape	Block
Radiation	MoK_α (λ=0.71073 Å)
2θ range [°]	3.77 to 61.09 (0.70 Å)

Index ranges	−12 ≤ h ≤ 12<br>−15 ≤ k ≤ 15<br>−18 ≤ l ≤ 18
Reflections collected	85969
Independent reflections	7081<br>R_{int} = 0.0353<br>R_{sigma} = 0.0167
Completeness to θ = 25.242°	100.0 %
Data / Restraints / Parameters	7081/129/398
Absorption correction T_{min}/T_{max} (method)	0.7025/0.7251 (multi-scan)
Goodness-of-fit on F^2	1.028
Final *R* indexes [I≥2σ(I)]	R_1 = 0.0375<br>wR_2 = 0.0947
Final *R* indexes [all data]	R_1 = 0.0447<br>wR_2 = 0.1006
Largest peak/hole [eÅ^{-3}]	0.45/−0.46

Compound 10i

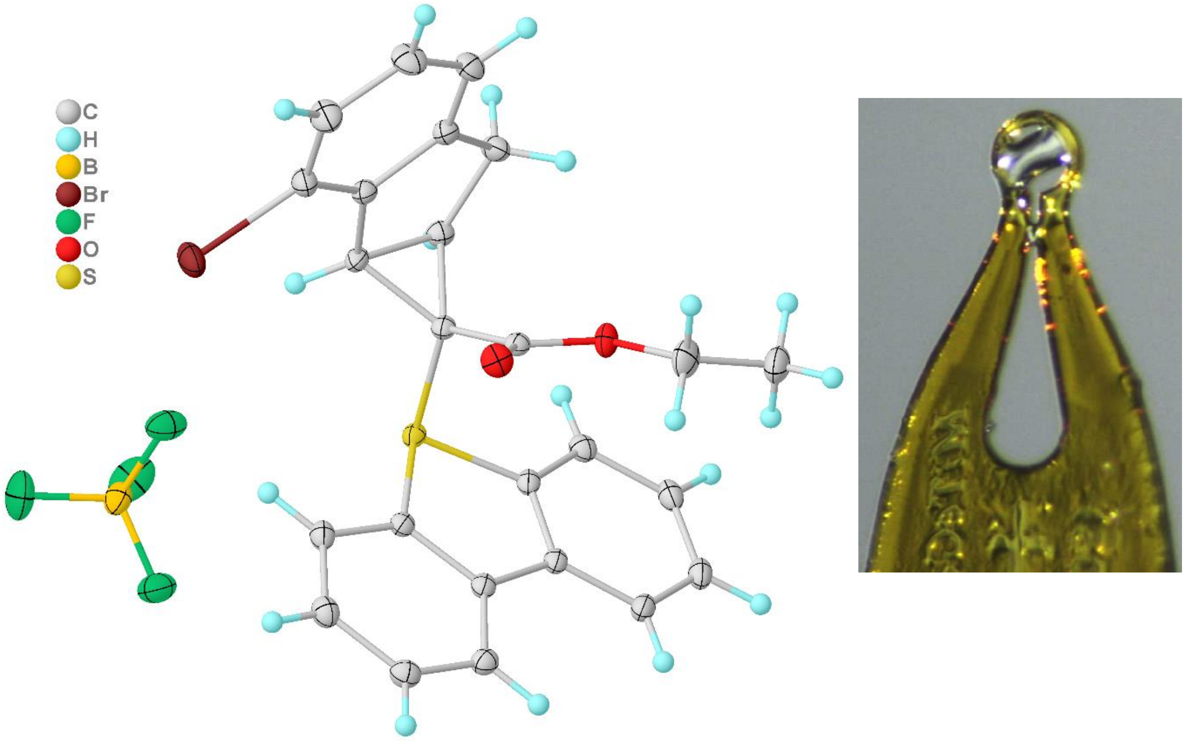

Figure S27: Full asymmetric unit of **10i**. Displacement ellipsoids are drawn at 50% probability level. Single crystals were obtained by evaporation from a solution in diethylether.

CCDC number	2332880
Empirical formula	$C_{25}H_{20}BBrF_4O_2S$
Formula weight	551.19
Temperature [K]	100.00
Crystal system	Triclinic
Space group (number)	$P\bar{1}$ (2)
a [Å]	8.9043(10)
b [Å]	11.4746(13)
c [Å]	12.2940(12)
α [°]	92.737(3)
β [°]	99.024(3)
γ [°]	111.462(3)
Volume [Å^3]	1147.0(2)
Z	2
ρ_{calc} [gcm^{-3}]	1.596
μ [mm^{-1}]	1.937
F(000)	556
Crystal size [mm^3]	0.19×0.112×0.049
Crystal color	Colorless
Crystal shape	Block
Radiation	MoK_α (λ=0.71073 Å)

2θ range [°]	4.81 to 61.16 (0.70 Å)
Index ranges	−12 ≤ h ≤ 12<br>−16 ≤ k ≤ 16<br>−16 ≤ l ≤ 17
Reflections collected	63014
Independent reflections	7033<br>R_{int} = 0.0452<br>R_{sigma} = 0.0222
Completeness to θ = 25.242°	100.0 %
Data / Restraints / Parameters	7033/4/320
Goodness-of-fit on F^2	1.022
Final *R* indexes [$I \geq 2\sigma(I)$]	R_1 = 0.0285<br>wR_2 = 0.0700
Final *R* indexes [all data]	R_1 = 0.0337<br>wR_2 = 0.0729
Largest peak/hole [eÅ^{-3}]	0.91/-0.69

Compound 10t

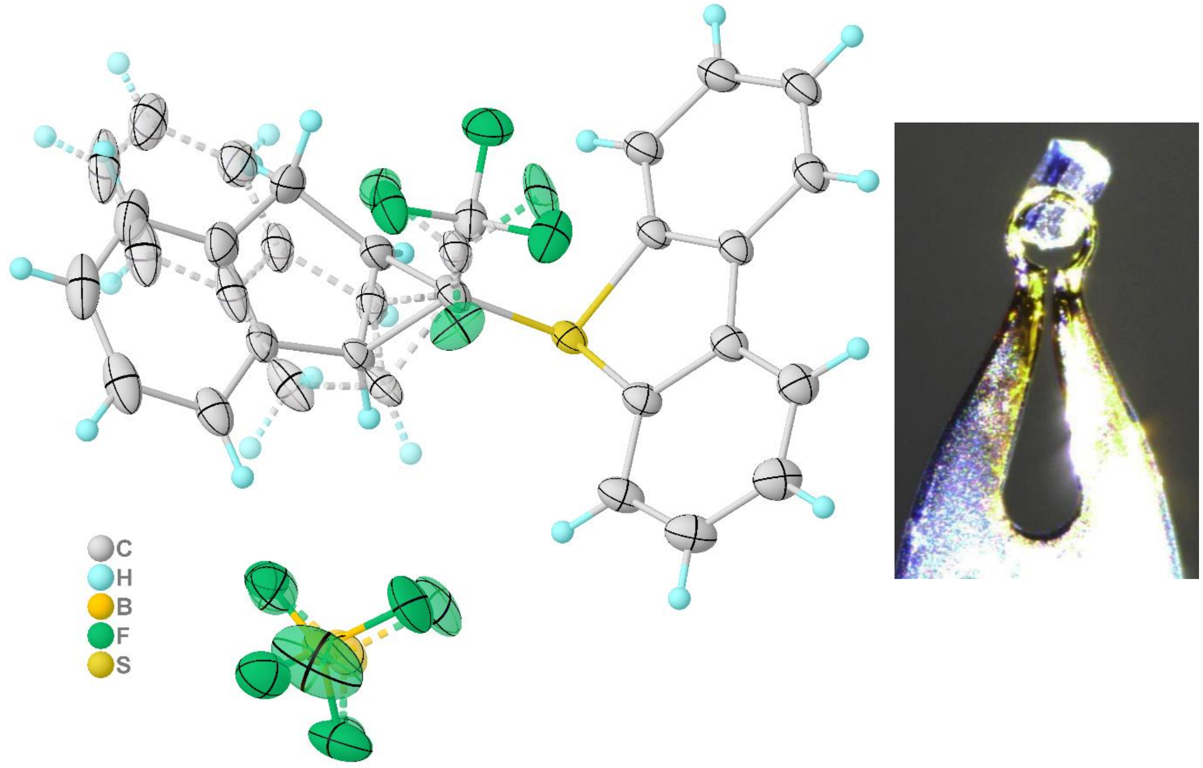

Figure S28: Full asymmetric unit of **10t**. Displacement ellipsoids are drawn at 50% probability level; major positional disorder was found for the indene group, the trifluoromethyl group and the tetrafluoroborate anion. Each disordered fragment was refined with an individual occupancy parameter giving for each a value close to 50%. The minor disordered model part is drawn translucent with stippled bonds. Single crystals were obtained by evaporation from a mixture of methanol, dichloromethane and diethylether.

CCDC number	2332881
Empirical formula	$C_{23}H_{16}BF_7S$
Formula weight	468.23
Temperature [K]	100.00
Crystal system	Triclinic
Space group (number)	$P\bar{1}$ (2)
a [Å]	9.3811(6)
b [Å]	10.8726(11)
c [Å]	12.1464(10)
α [°]	63.826(2)
β [°]	68.524(4)
γ [°]	80.590(2)
Volume [Å^3]	1034.66(15)
Z	2
ρ_{calc} [gcm^{-3}]	1.503
μ [mm^{-1}]	0.227
F(000)	476
Crystal size [mm^3]	0.277×0.125×0.11
Crystal color	Colorless
Crystal shape	Block
Radiation	MoK_α (λ=0.71073 Å)

2θ range [°]	3.96 to 65.16 (0.66 Å)
Index ranges	−14 ≤ h ≤ 14<br>−16 ≤ k ≤ 16<br>−18 ≤ l ≤ 18
Reflections collected	98974
Independent reflections	7529<br>R_{int} = 0.0220<br>R_{sigma} = 0.0096
Completeness to θ = 25.242°	100.0 %
Data / Restraints / Parameters	7529/76/453
Goodness-of-fit on F^2	1.085
Final *R* indexes [$I \geq 2\sigma(I)$]	R_1 = 0.0403<br>wR_2 = 0.1110
Final *R* indexes [all data]	R_1 = 0.0433<br>wR_2 = 0.1133
Largest peak/hole [eÅ^{-3}]	0.59/-0.34

SPECTROSCOPIC DATA

Compound **1a**:

^{1}H NMR (400 MHz, CD_3CN)

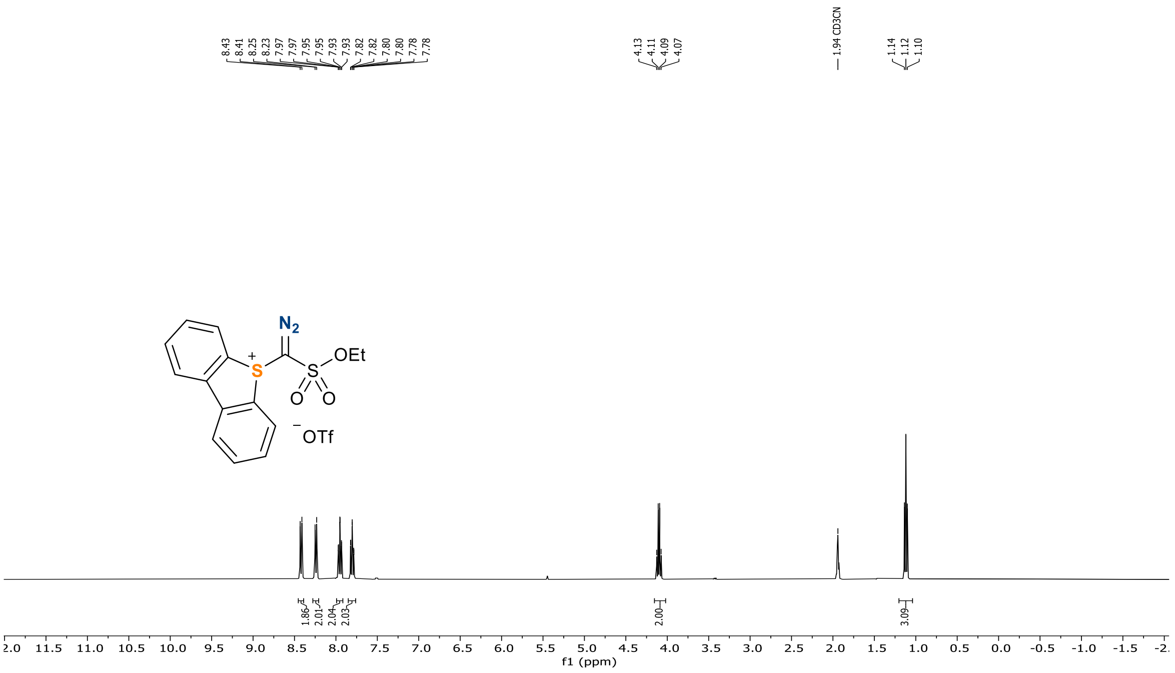

^{13}C{1H} NMR (101 MHz, CD_3CN)

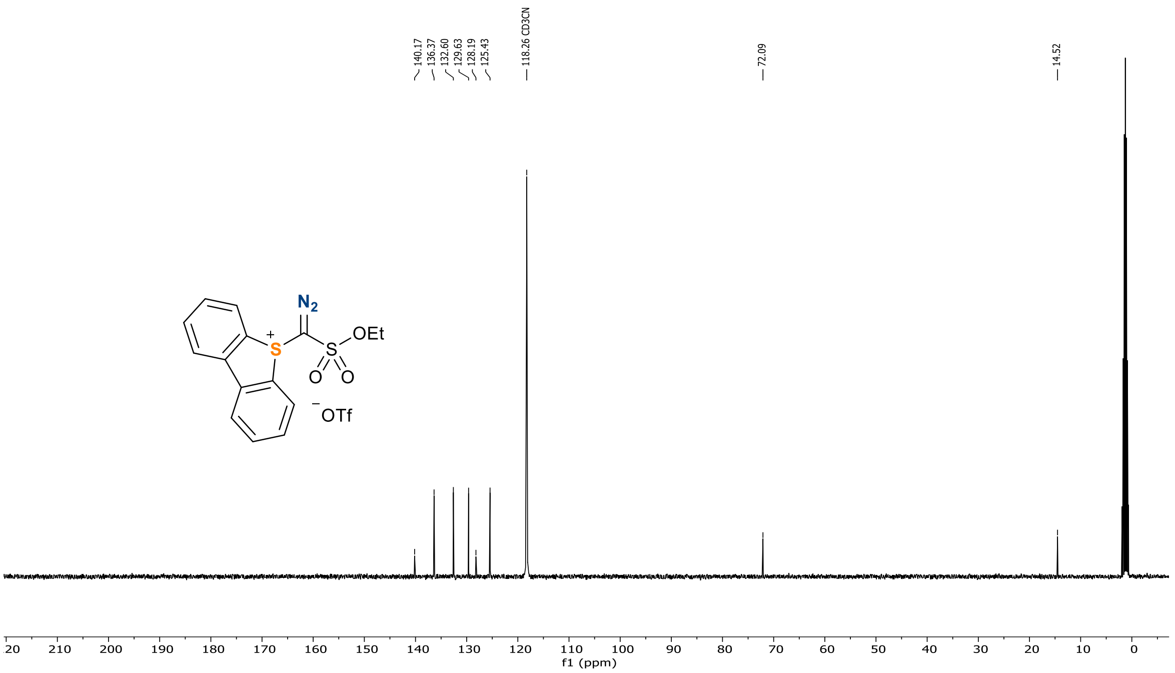

^{19}F NMR (377 MHz, CD_3CN)

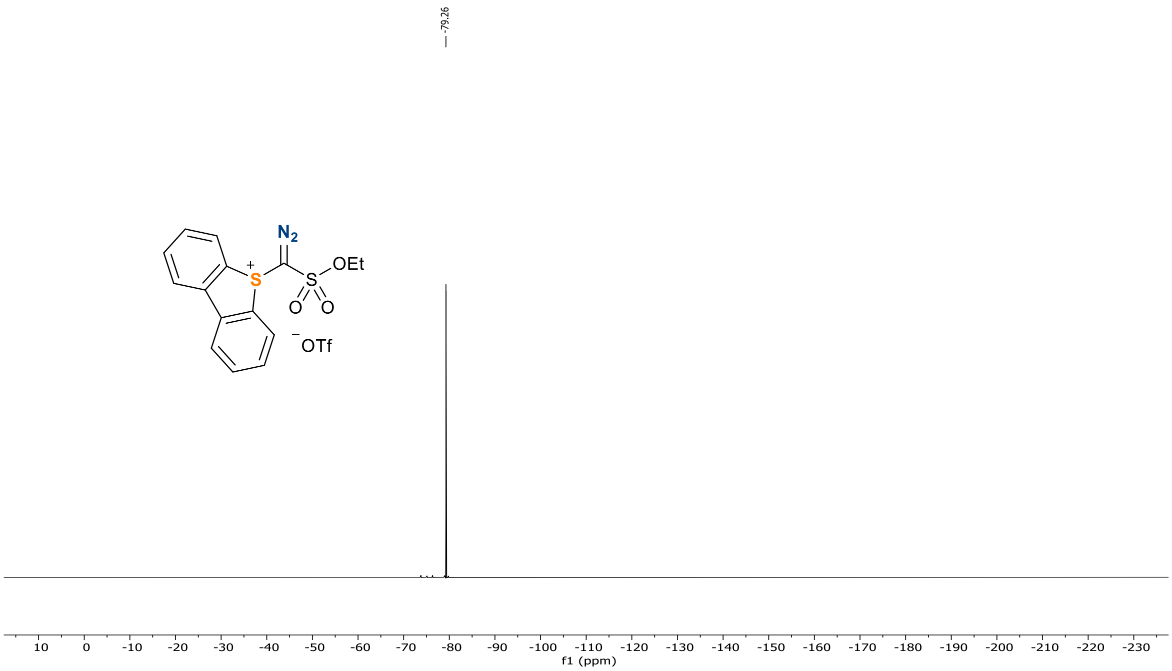

Compound **1b**:

^{1}H NMR (400 MHz, CD_3CN)

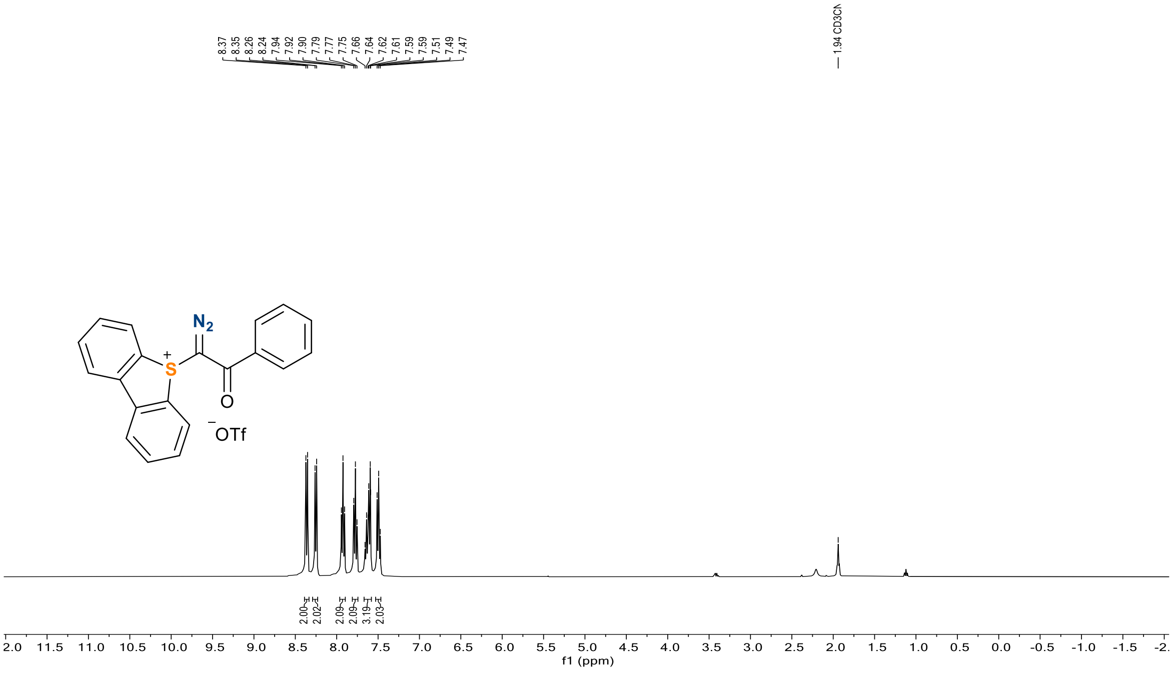

$^{13}C\{^1H\}$ NMR (101 MHz, CD_3CN)

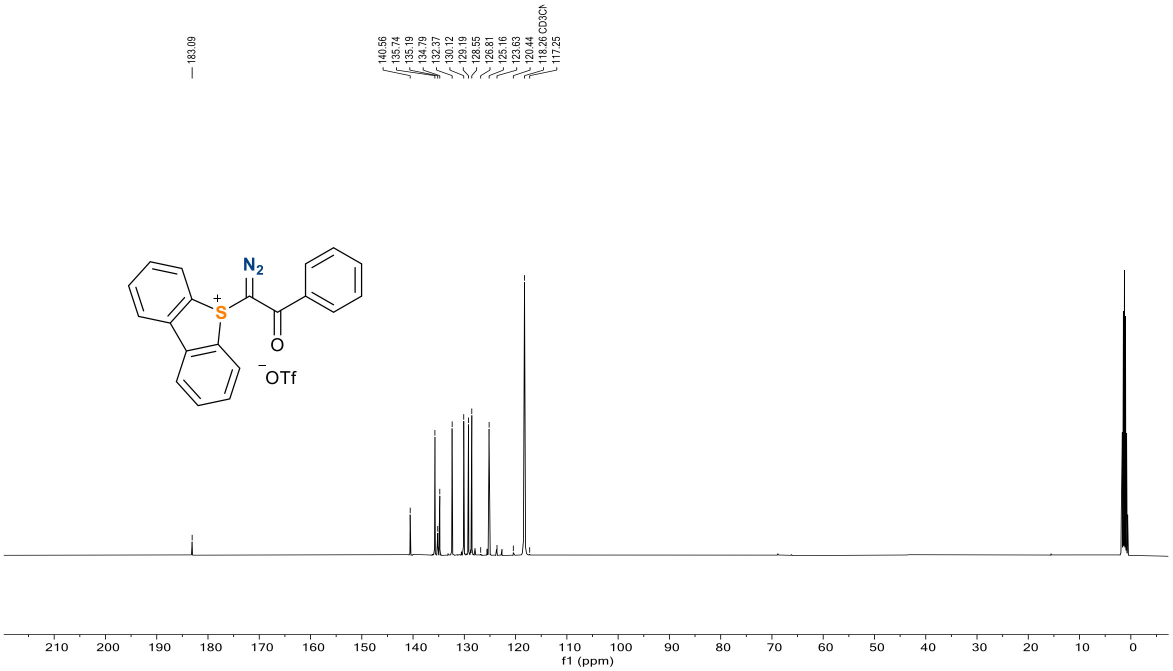

^{19}F NMR (377 MHz, CD_3CN)

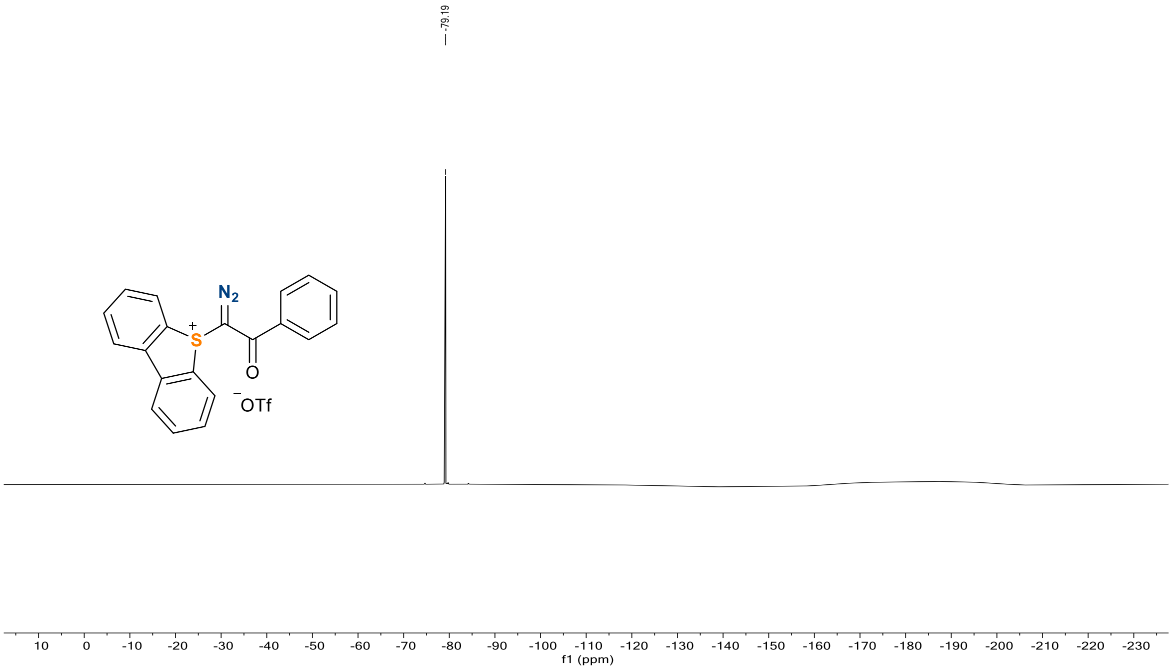

Compound **1c**

^{1}H NMR (400 MHz, CD_3CN)

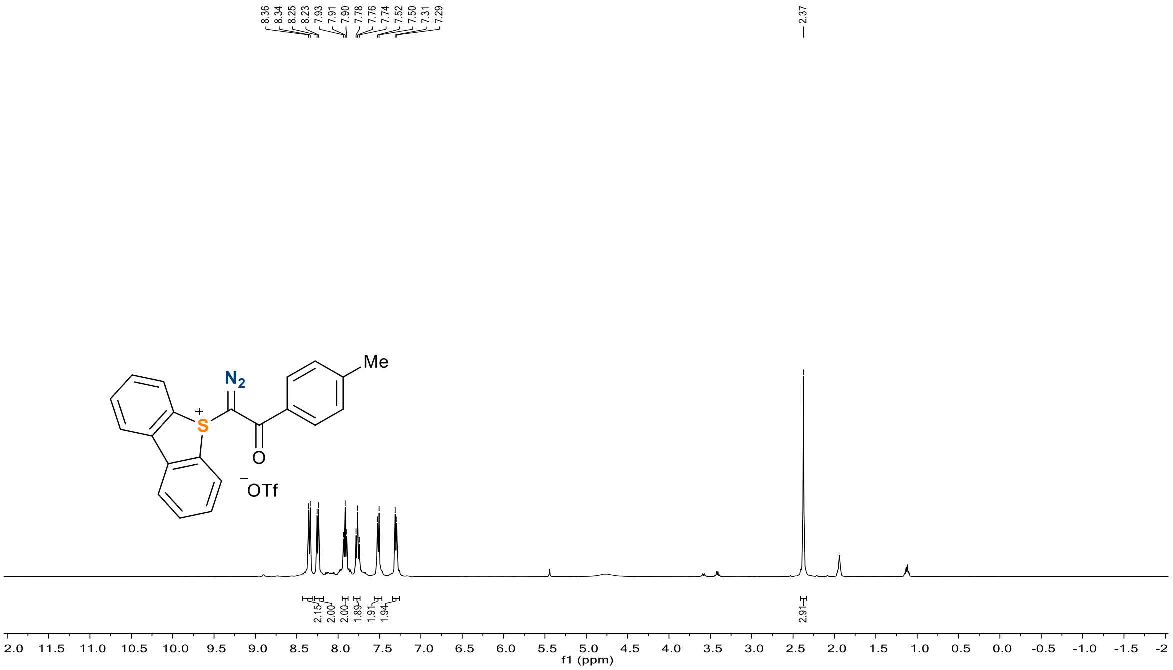

$^{13}C\{1H\}$ NMR (101 MHz, CD_3CN)

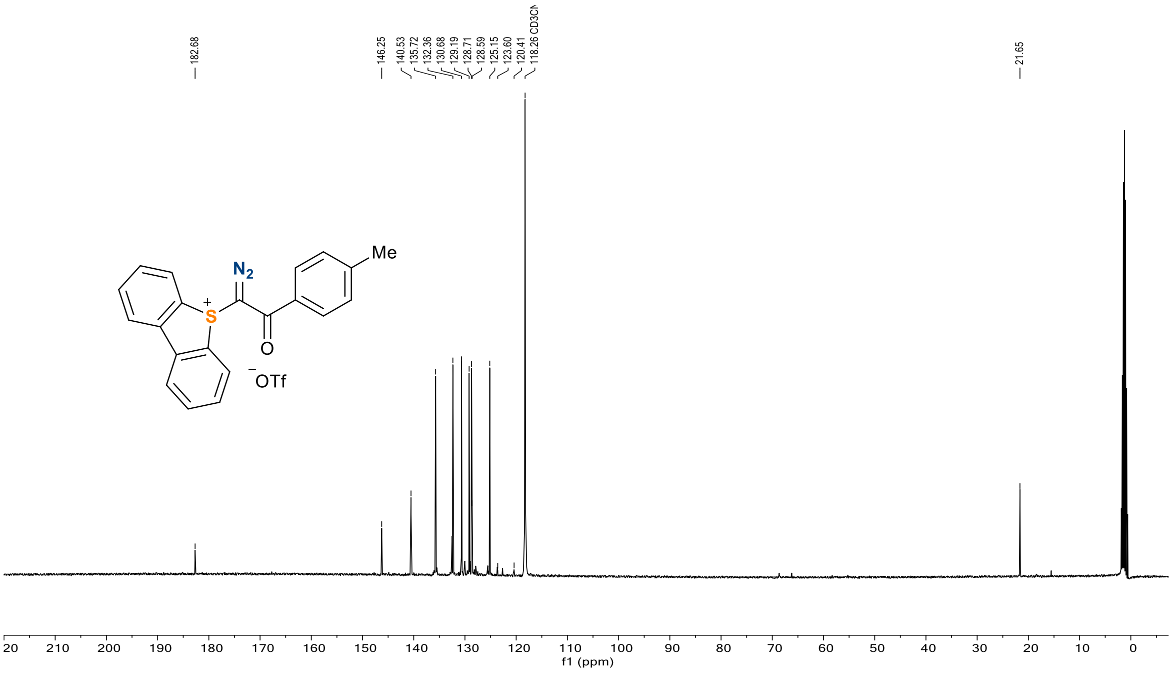

^{19}F NMR (377 MHz, CD_3CN)

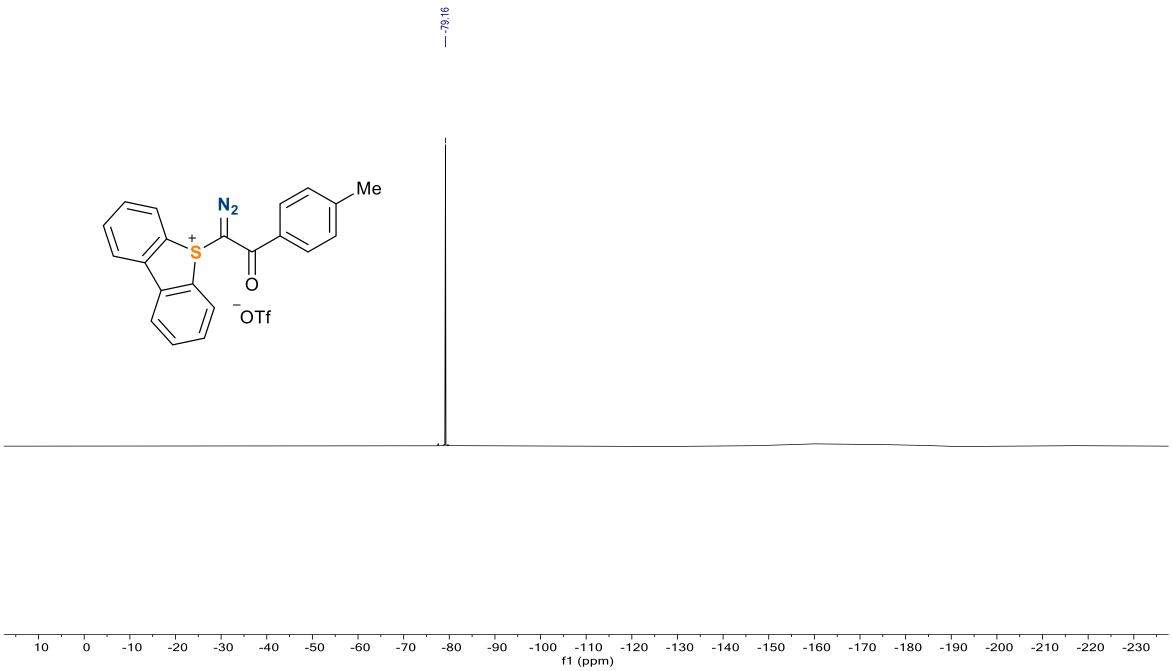

Compound **1d**

^1H NMR (400 MHz, CD_3CN)

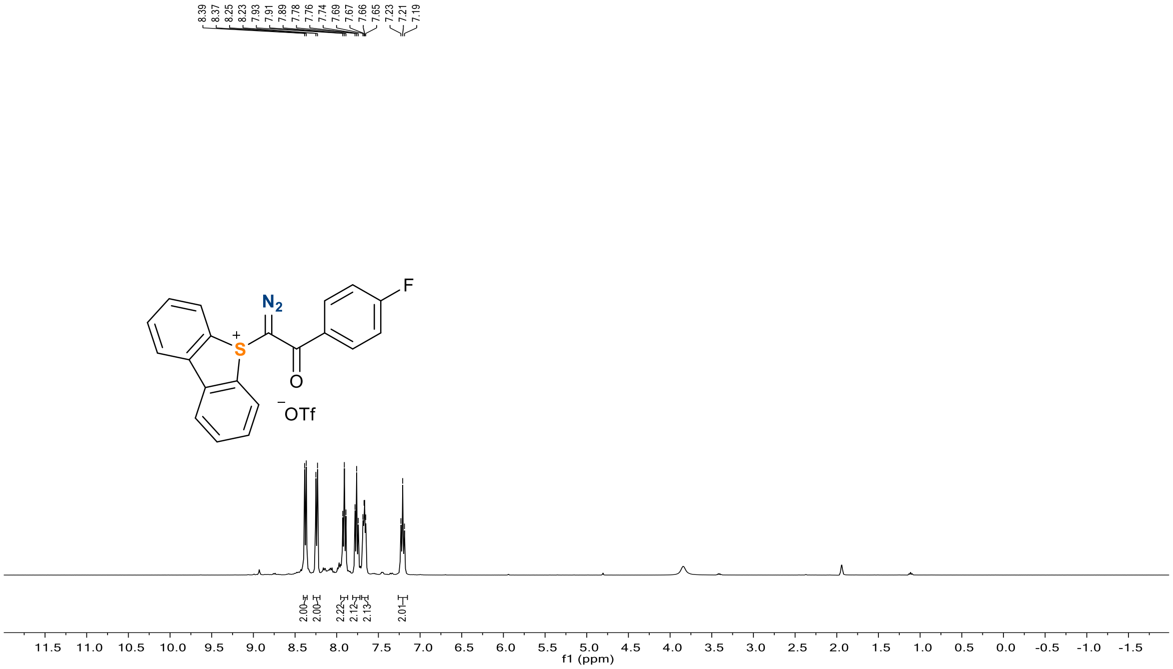

$^{13}C\{1H\}$ NMR (101 MHz, CD_3CN)

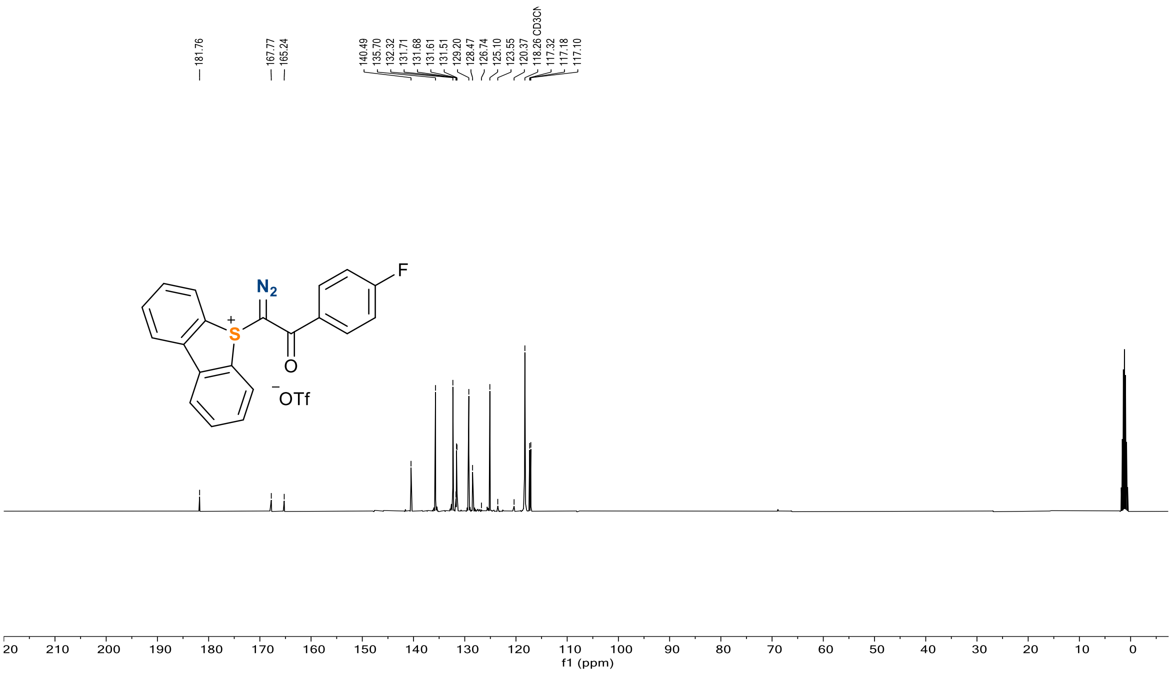

^{19}F NMR (377 MHz, CD_3CN)

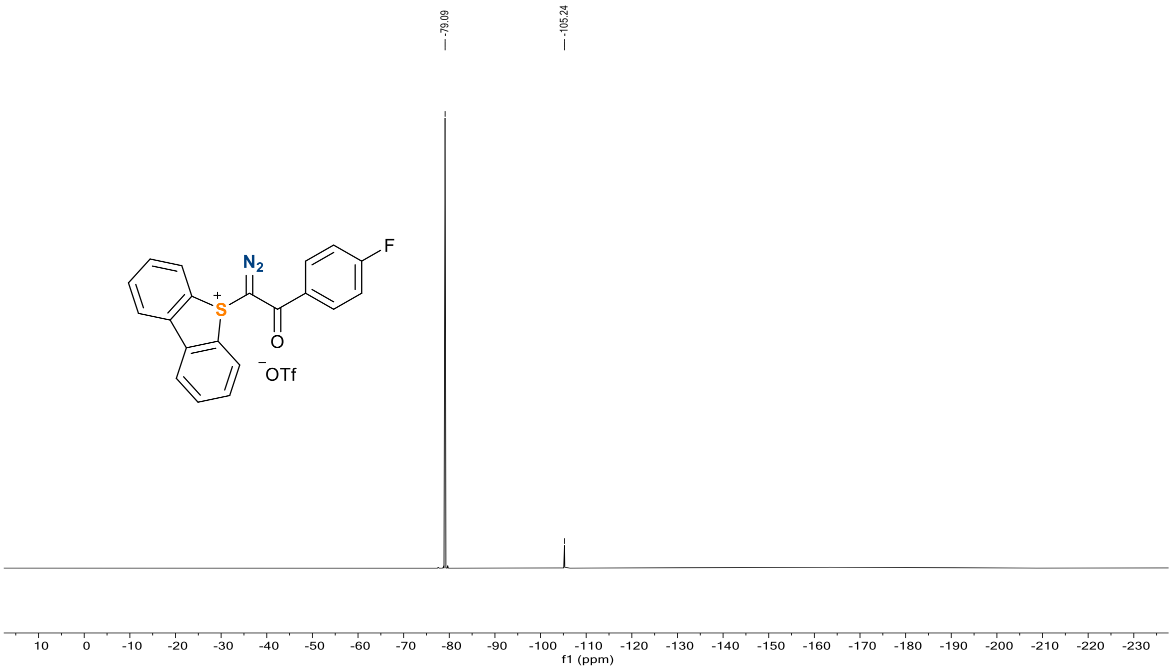

Compound **1e**:

^{1}H NMR (400 MHz, CD_3CN)

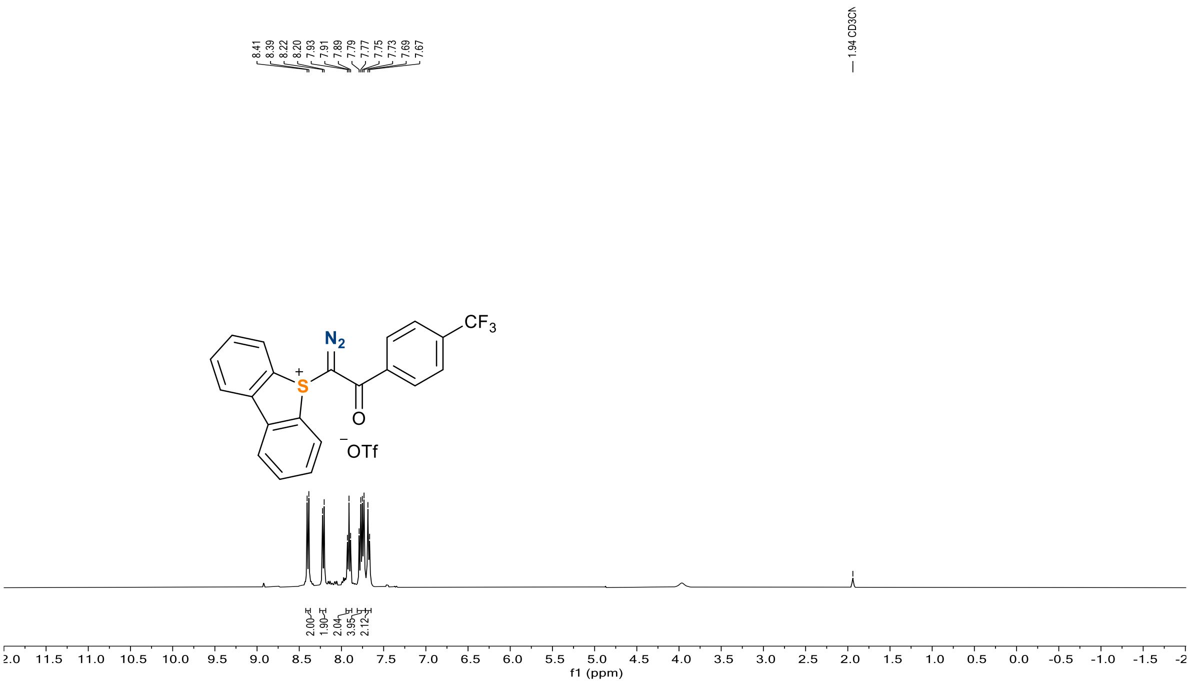

^{13}C{1H} NMR (101 MHz, CD_3CN)

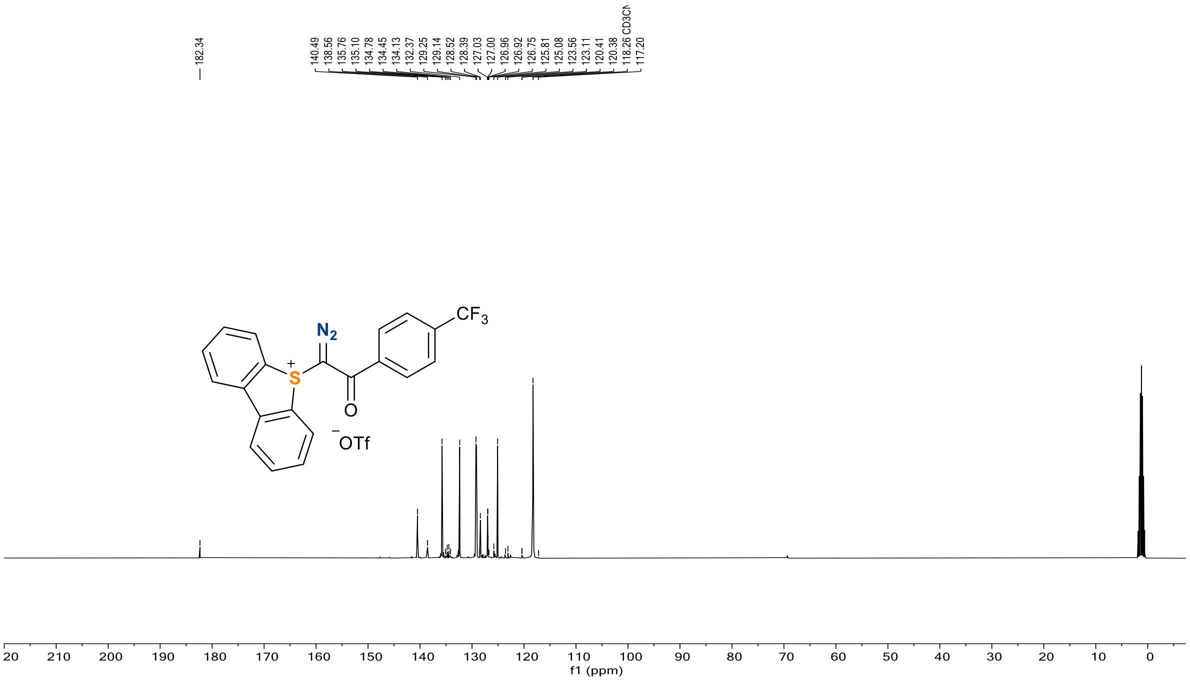

^{19}F NMR (377 MHz, CD_3CN)

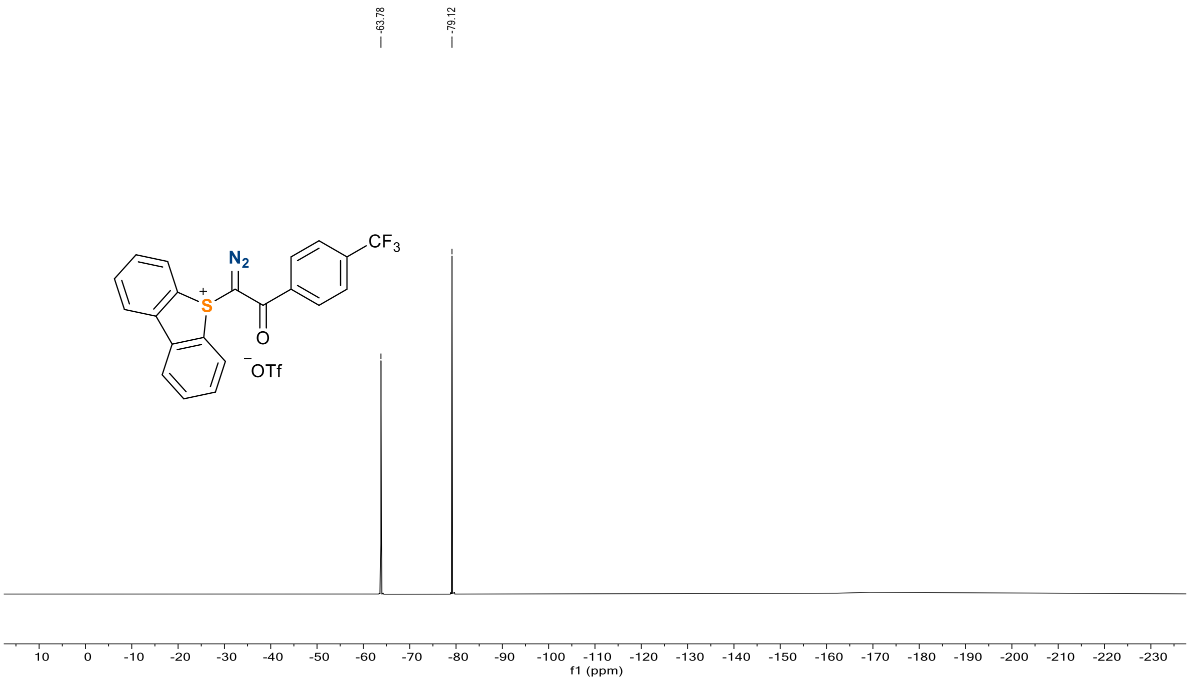

Compound **1f**:

^{1}H NMR (300 MHz, CD_3CN)

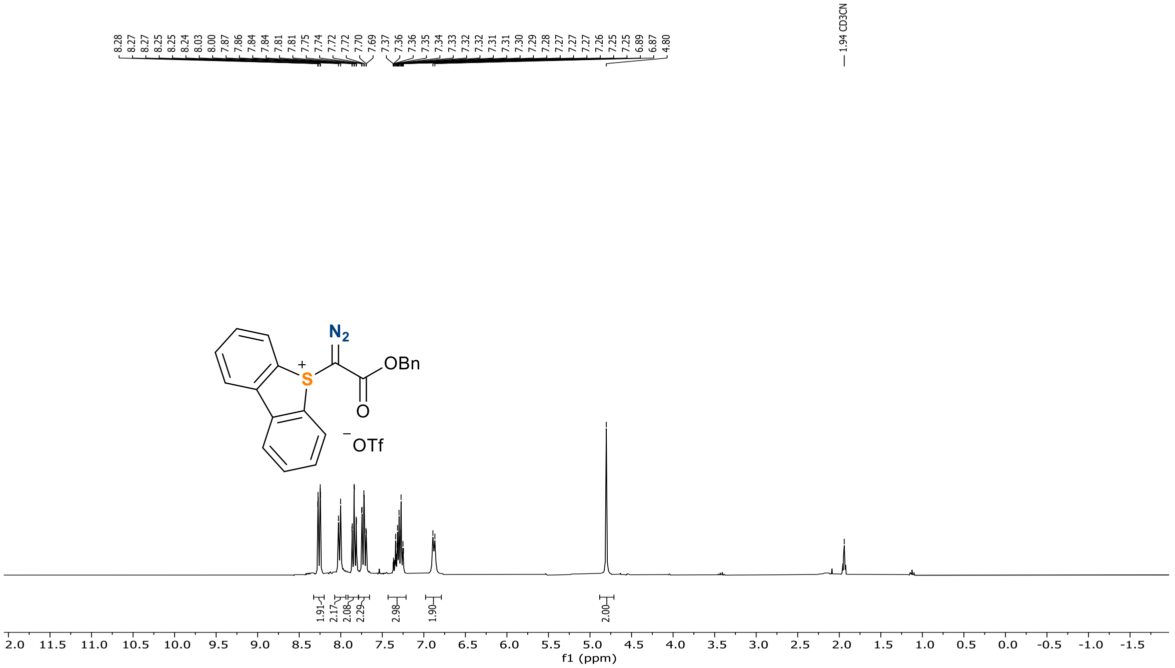

$^{13}C\{1H\}$ NMR (101 MHz, CD_3CN)

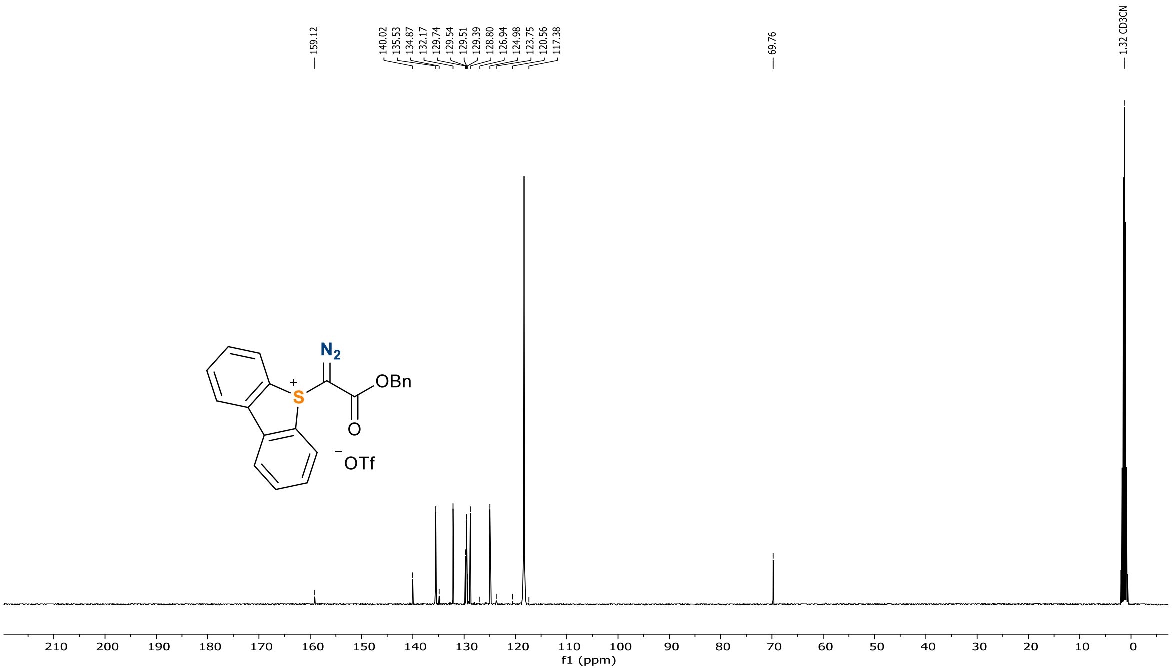

^{19}F NMR (377 MHz, CD_3CN)

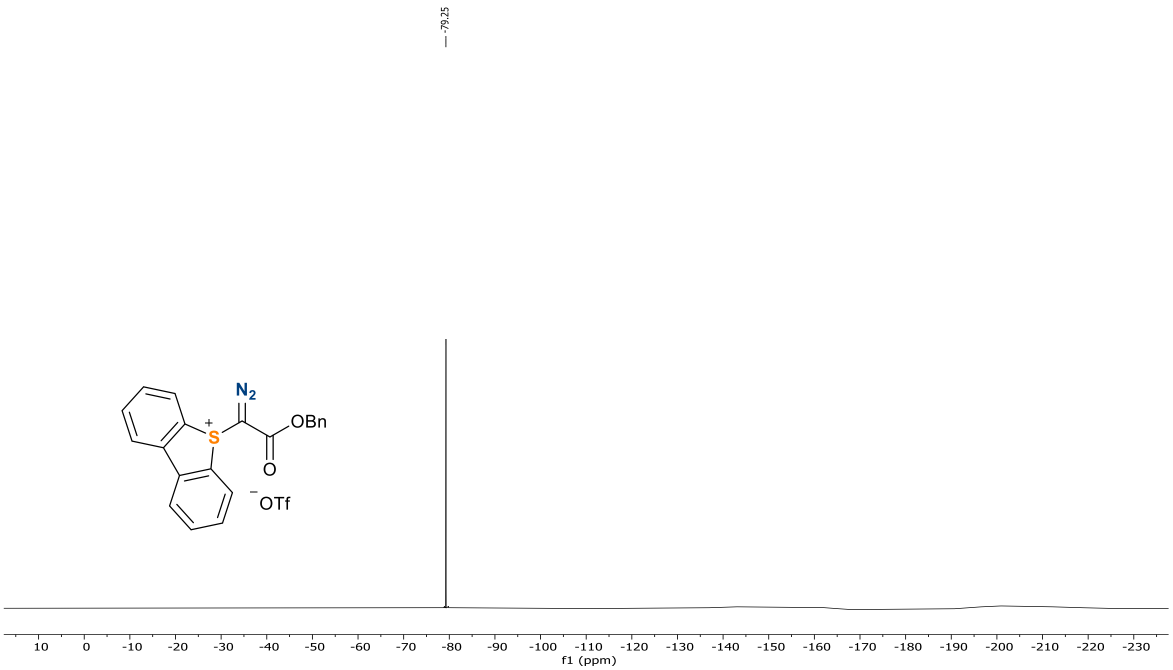

Compound **1g**:

^{1}H NMR (400 MHz, CD_3CN)

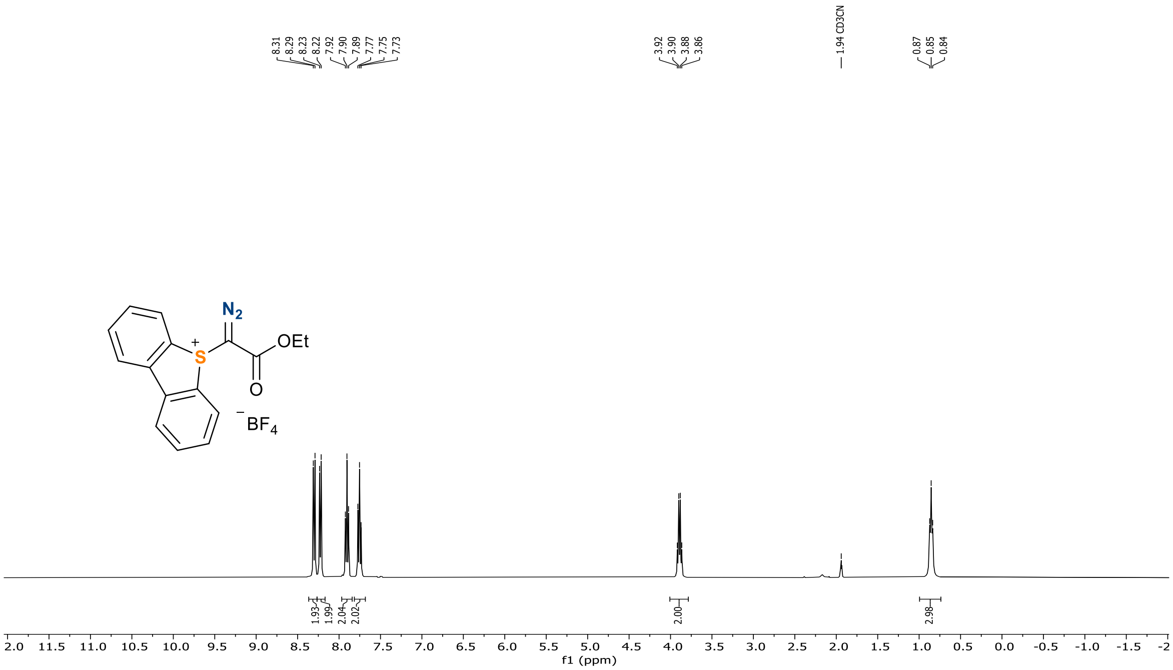

^{13}C{1H} NMR (101 MHz, CD_3CN)

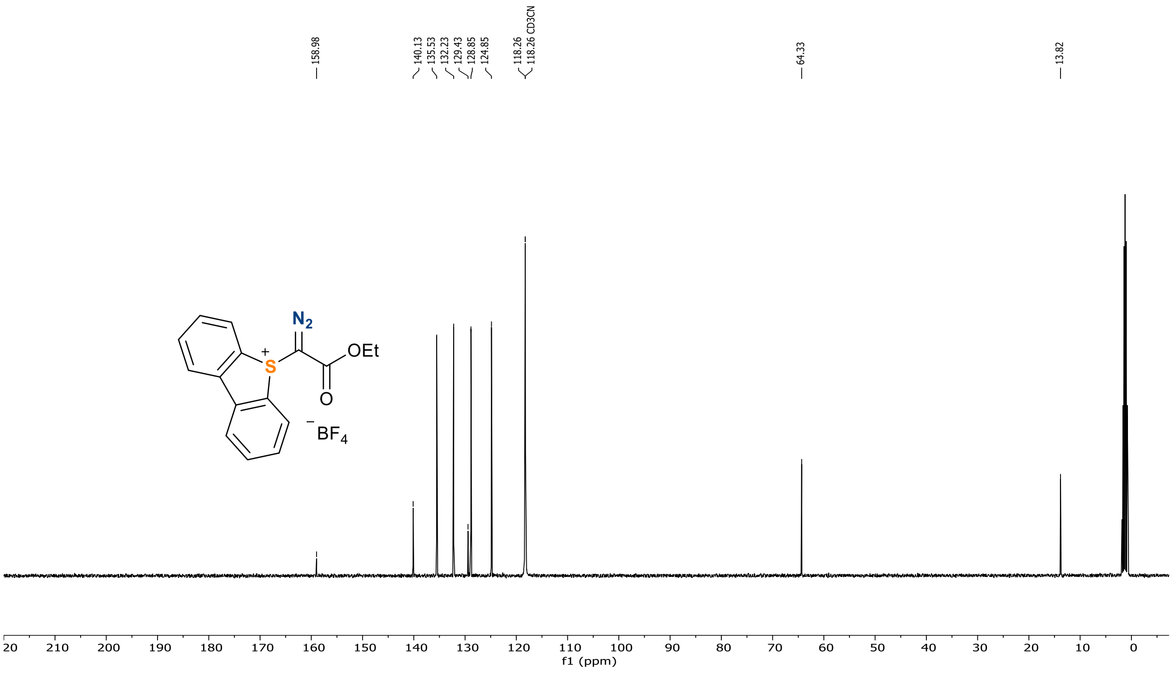

^{19}F NMR (377 MHz, CD_3CN)

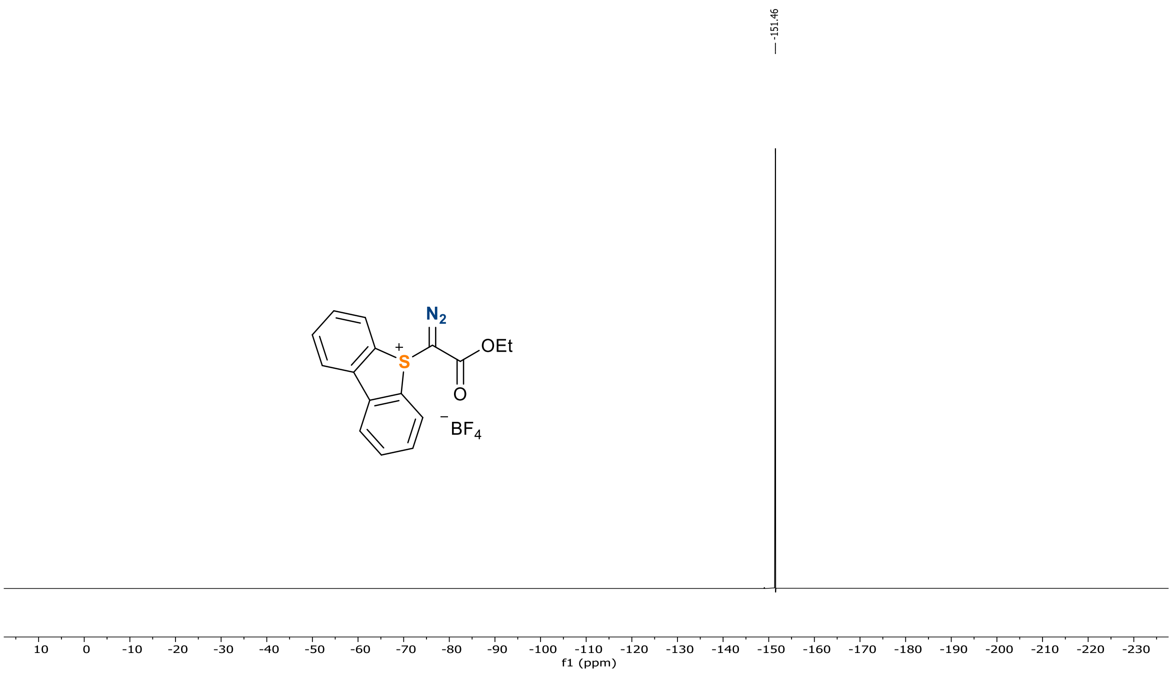

Compound **1h**:

^{1}H NMR (400 MHz, CD_3CN)

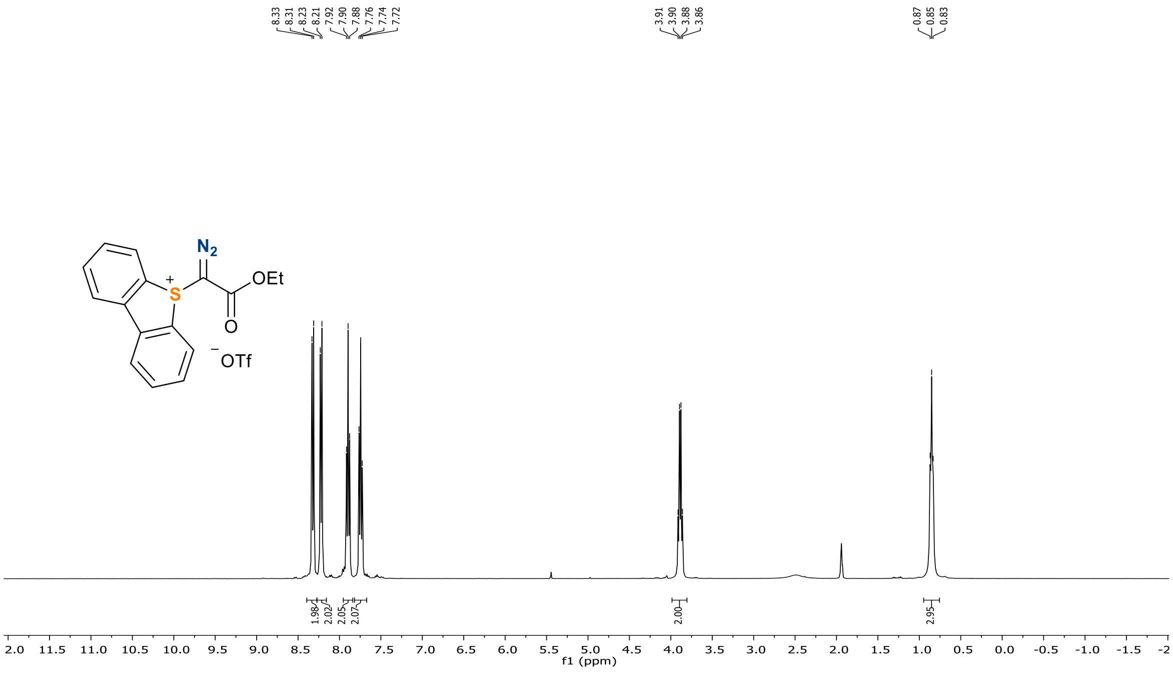

$^{13}C\{1H\}$ NMR (101 MHz, CD_3CN)

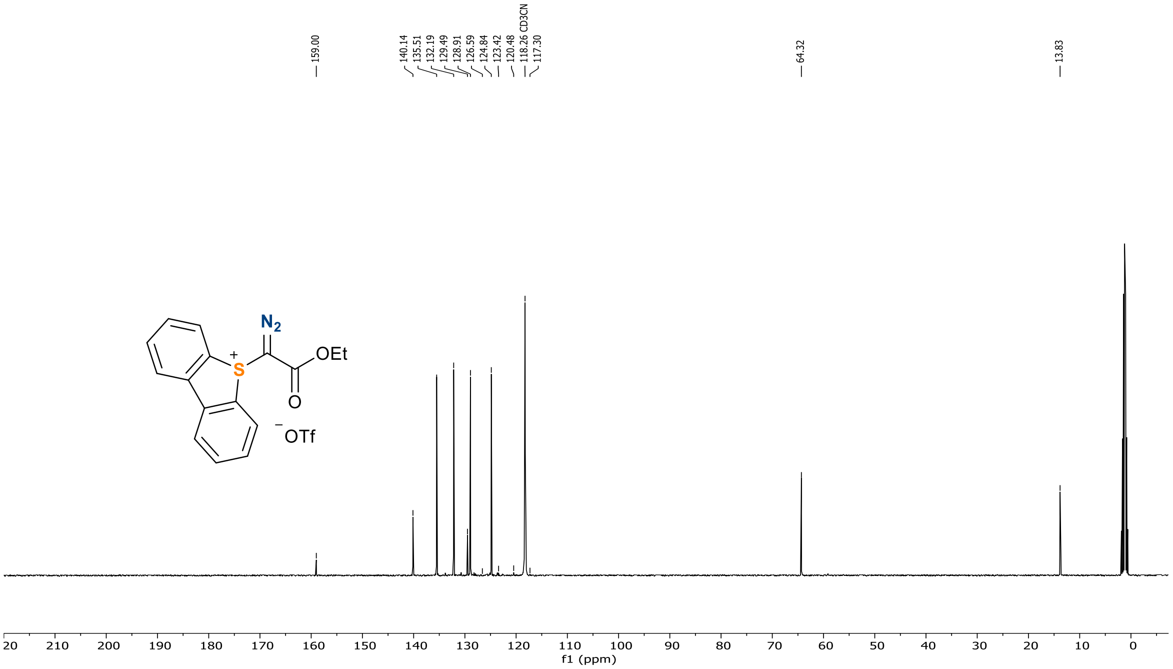

^{19}F NMR (377 MHz, CD_3CN)

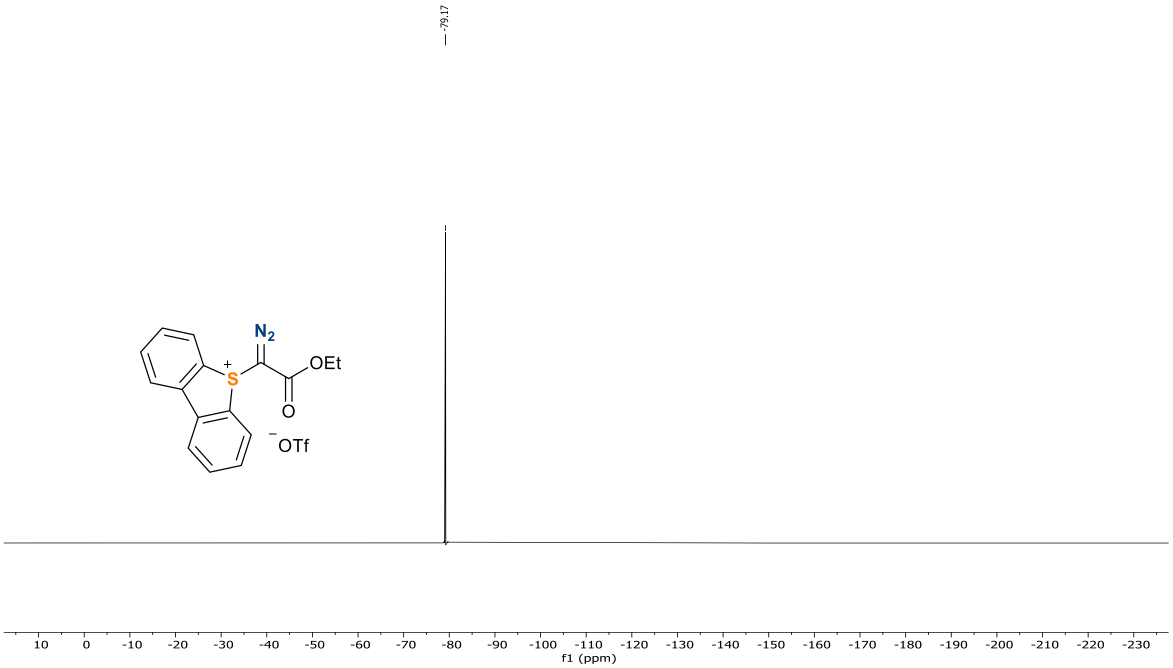

Compound **1i**:

^1H NMR (300 MHz, CD_3CN)

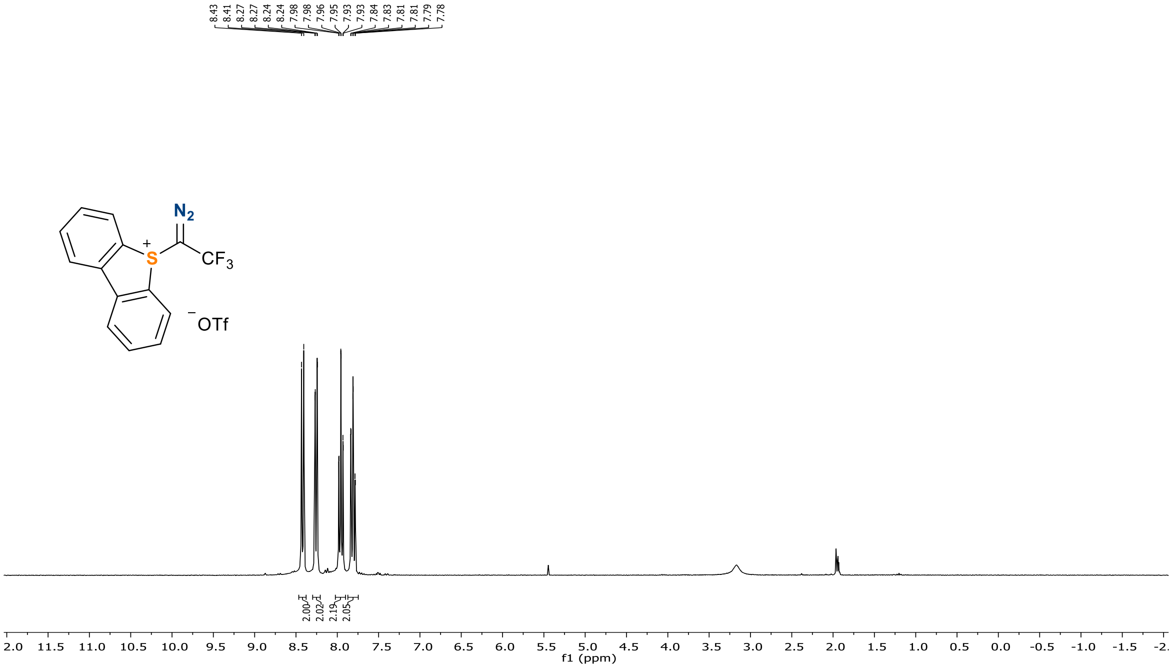

$^{13}C\{1H\}$ NMR (101 MHz, CD_3CN)

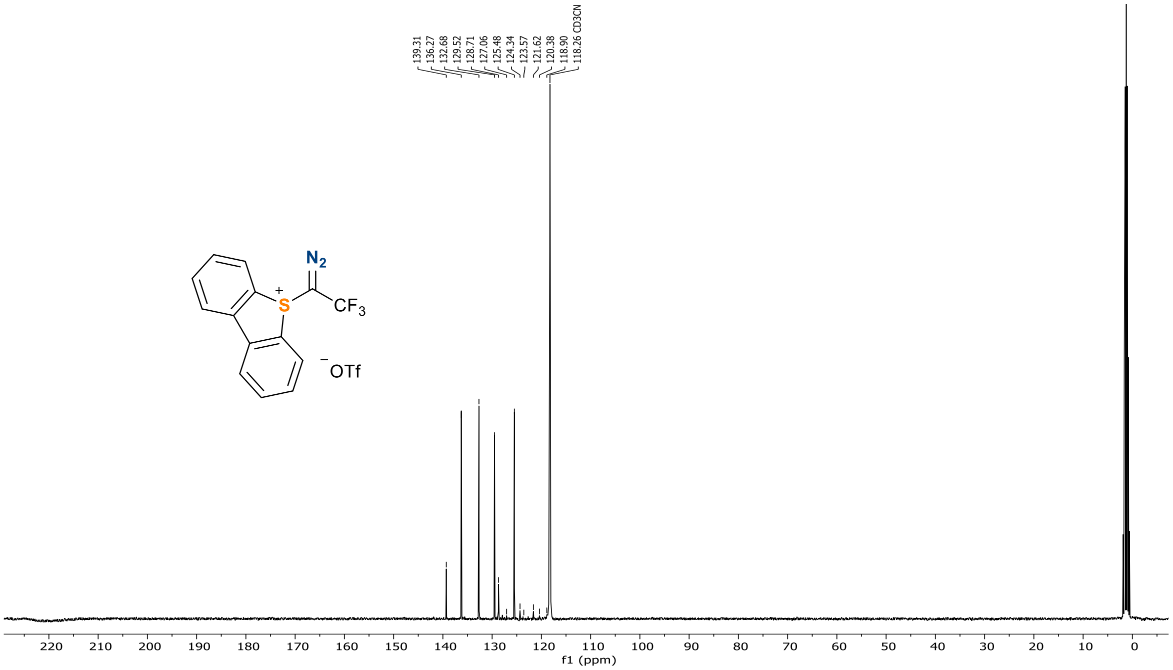

^{19}F NMR (282 MHz, CD_3CN)

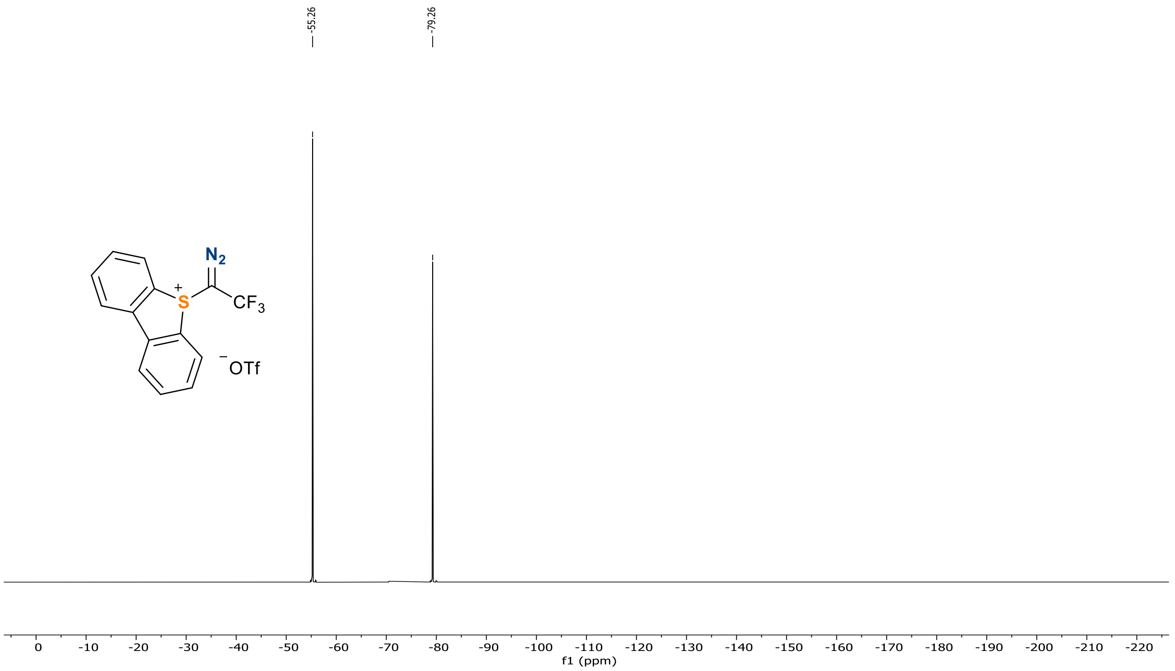

Compound **1j**:

^{1}H NMR (300 MHz, CD_3CN)

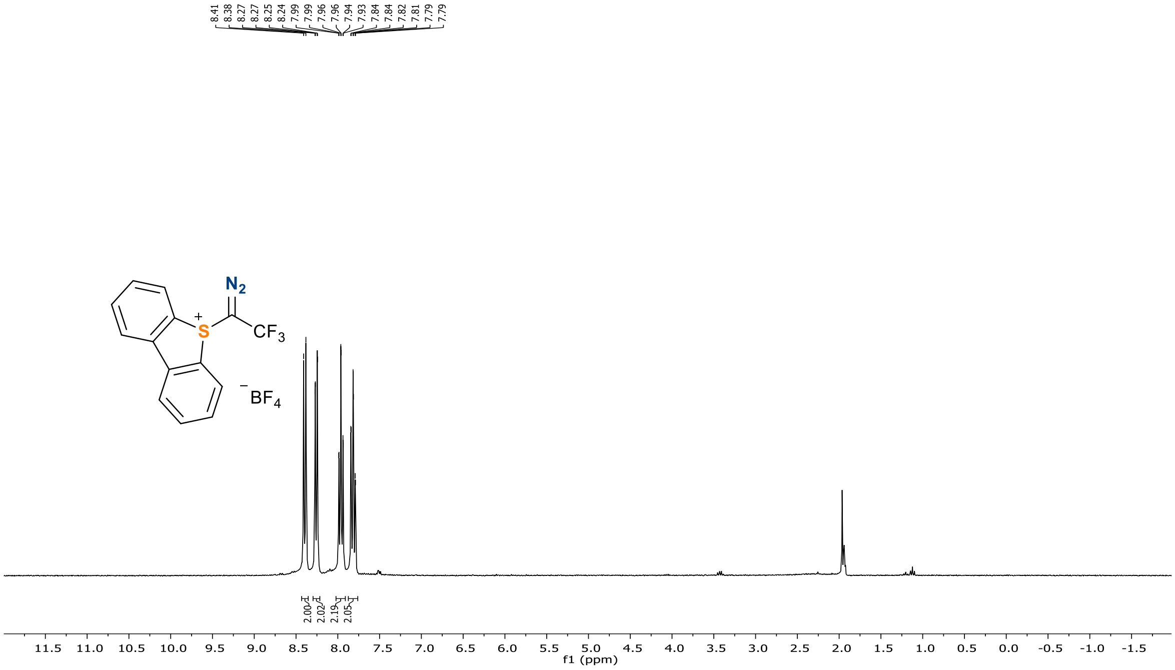

$^{13}C\{1H\}$ NMR (101 MHz, CD_3CN)

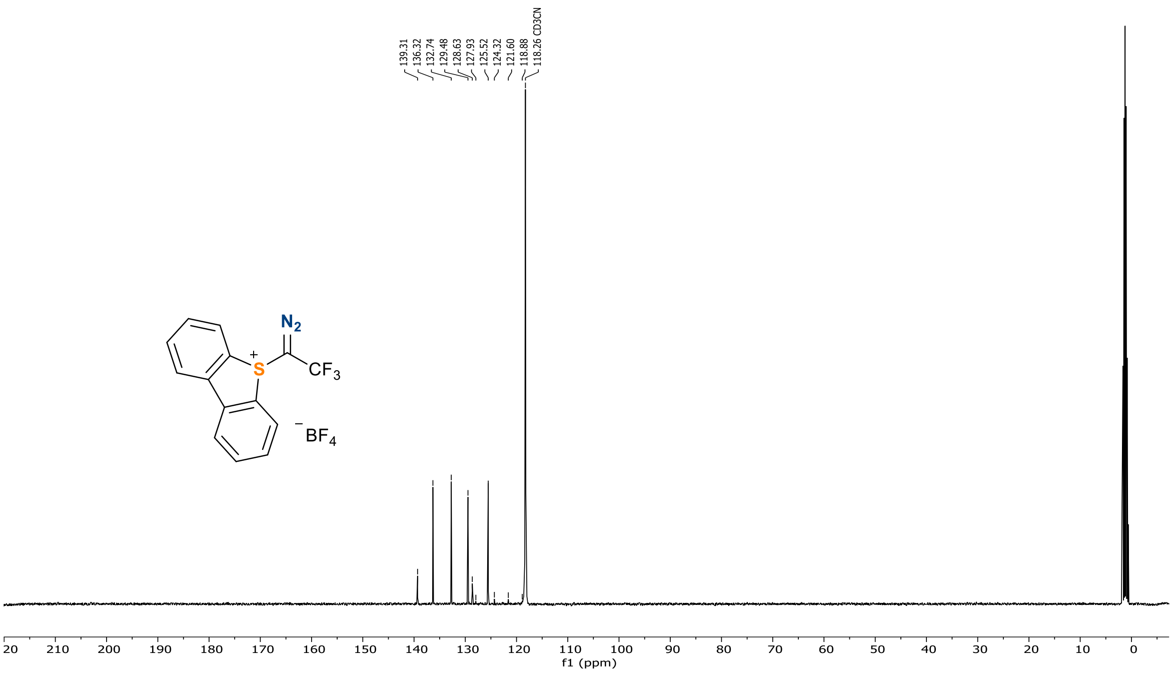

^{19}F NMR (282 MHz, CD_3CN)

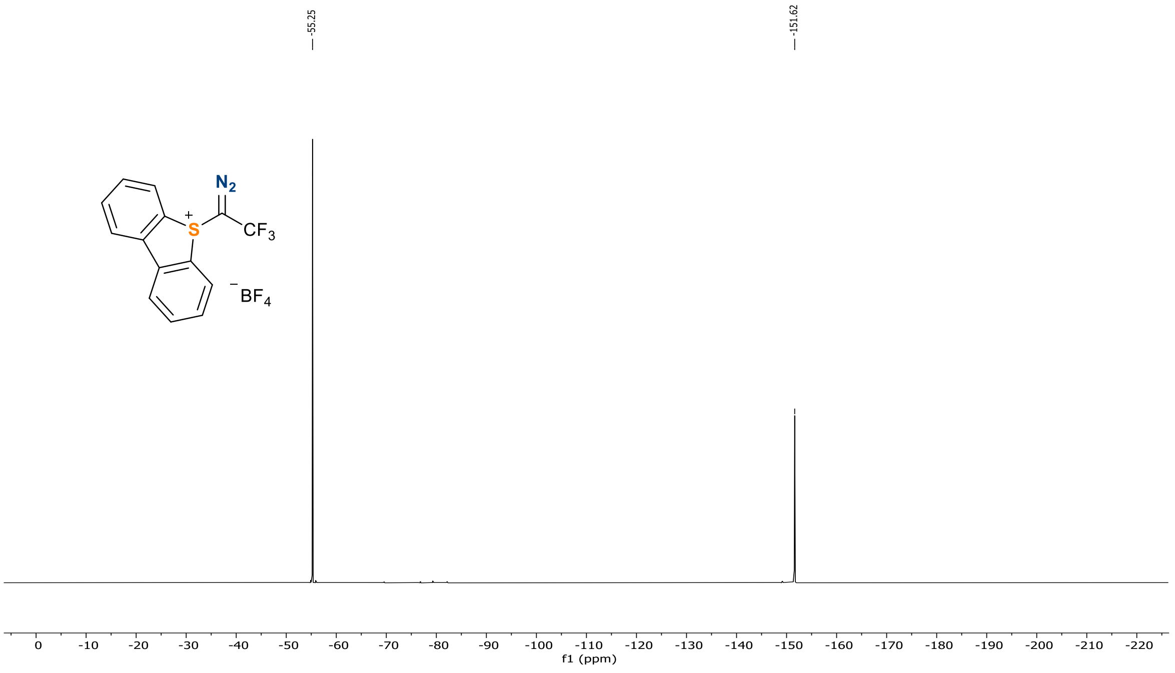

Compound **3a**:

^{1}H NMR (400 MHz, CD_3CN)

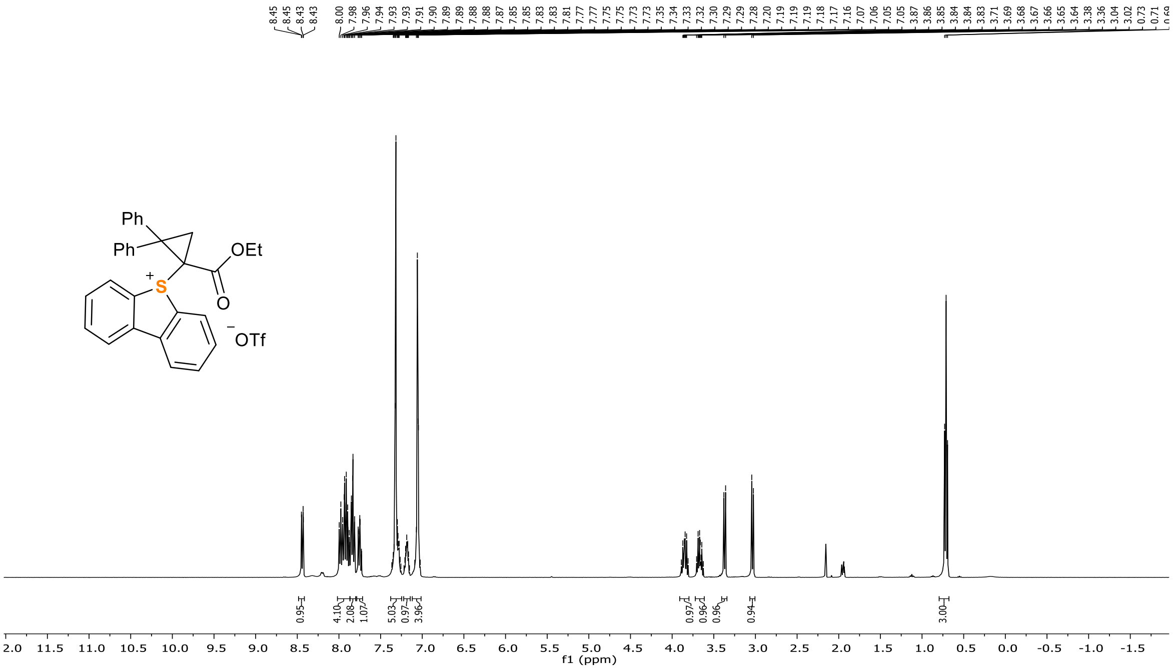

^{13}C{1H} NMR (101 MHz, CD_3CN)

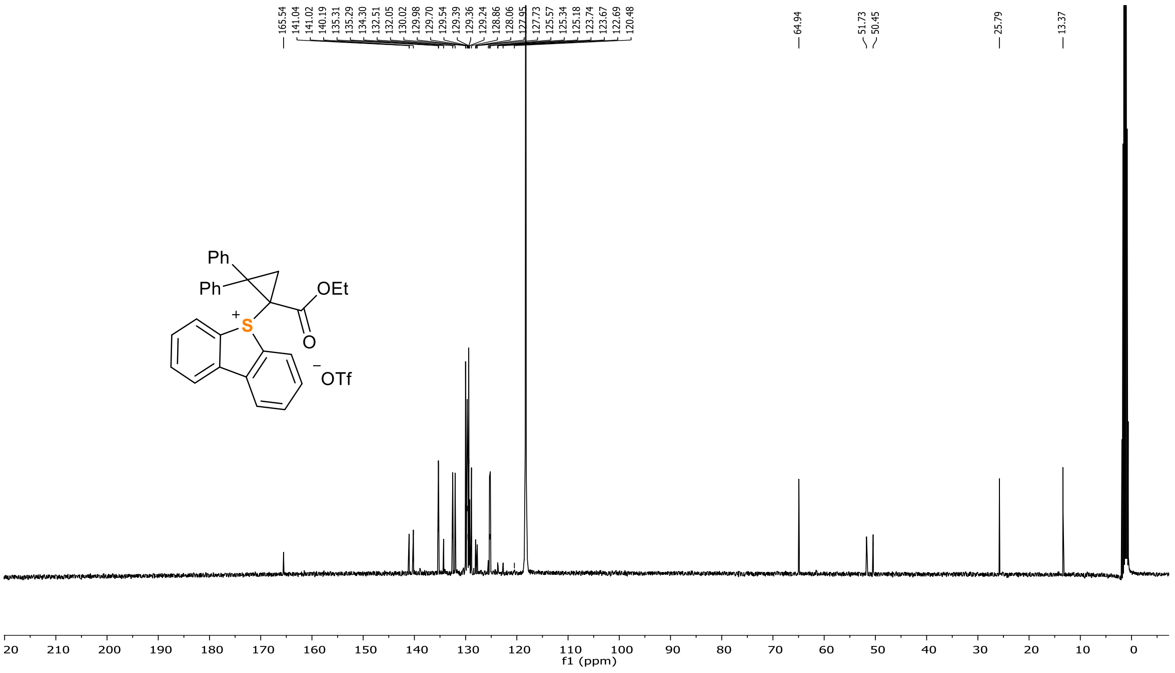

^{19}F NMR (377 MHz, CD_3CN)

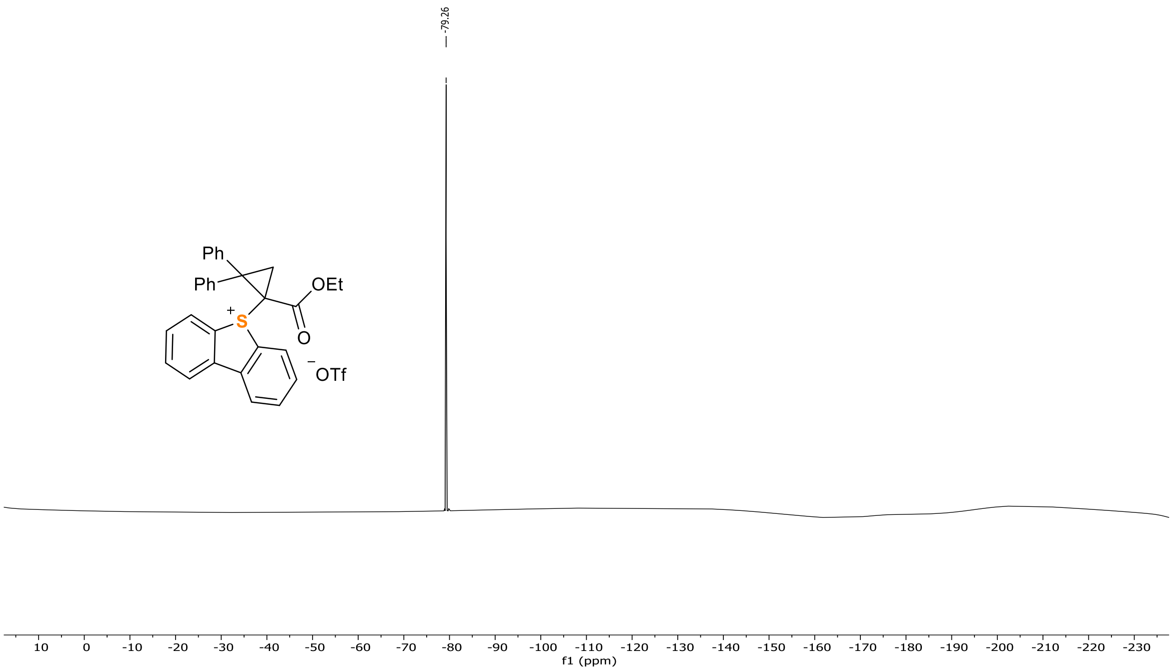

Compound **3b**:

^{1}H NMR (400 MHz, CD_3CN)

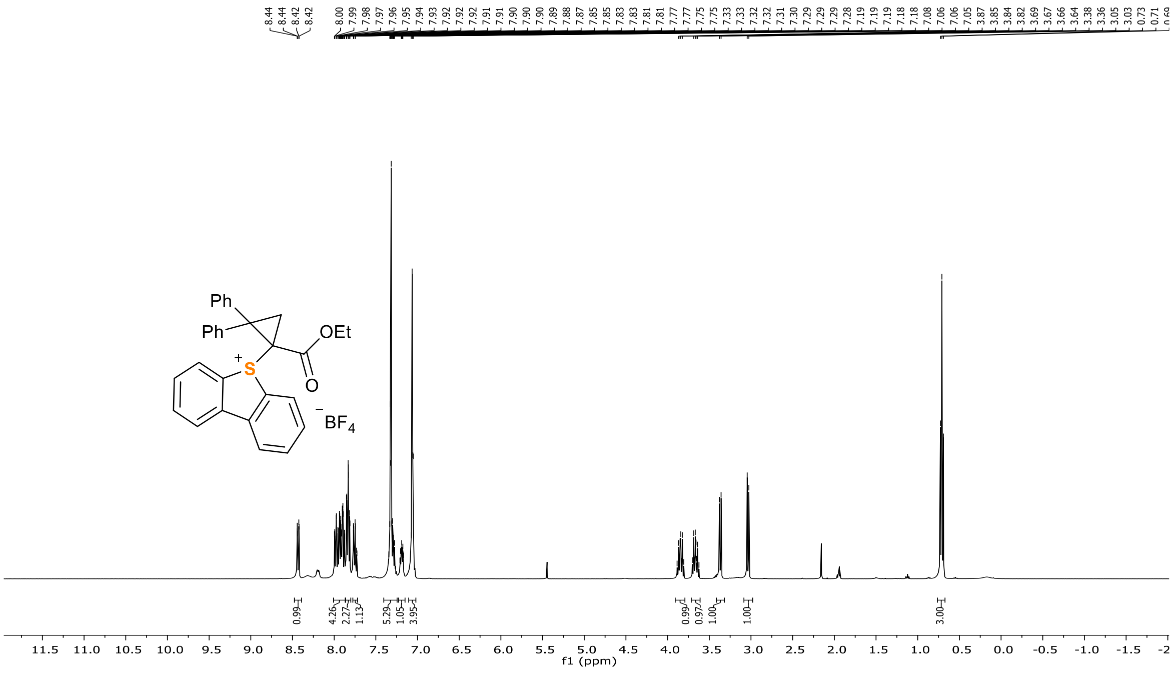

$^{13}C\{1H\}$ NMR (101 MHz, CD_3CN)

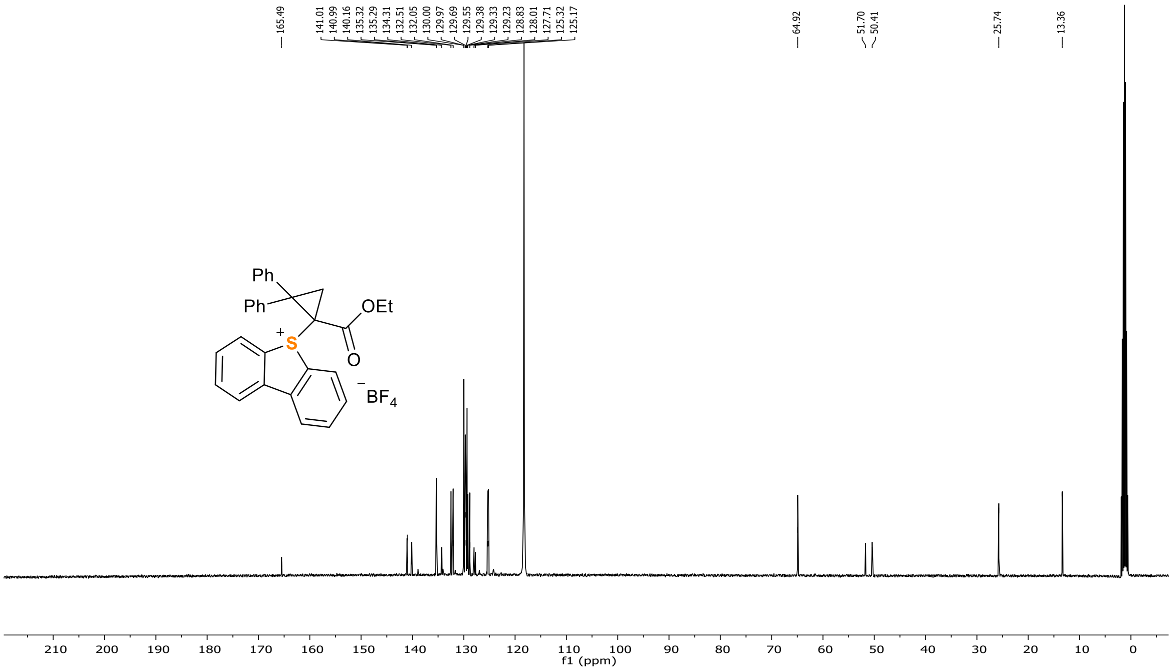

^{19}F NMR (377 MHz, CD_3CN)

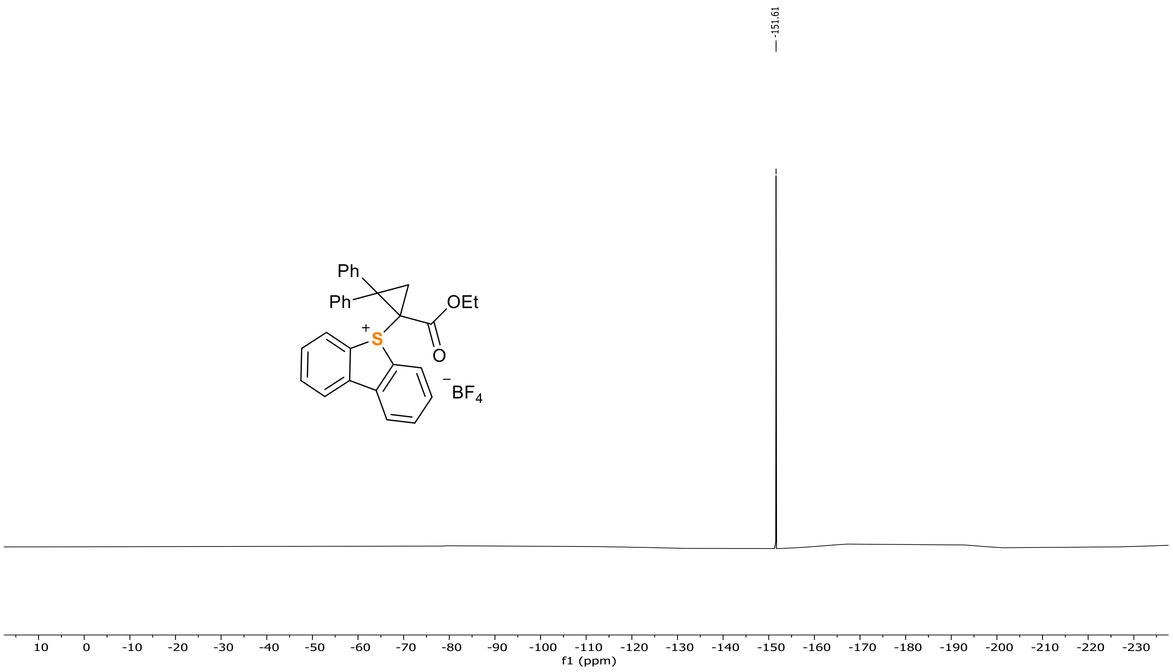

Compound **3c**:

^{1}H NMR (400 MHz, CD_3CN)

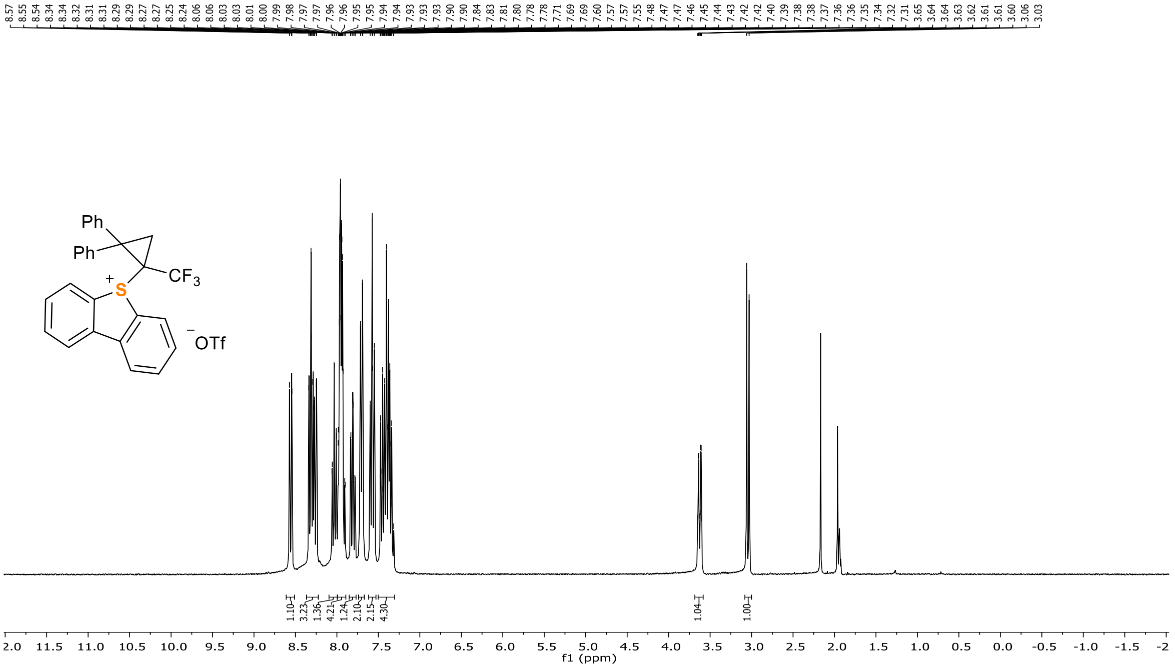

$^{13}C\{1H\}$ NMR (101 MHz, CD_3CN)

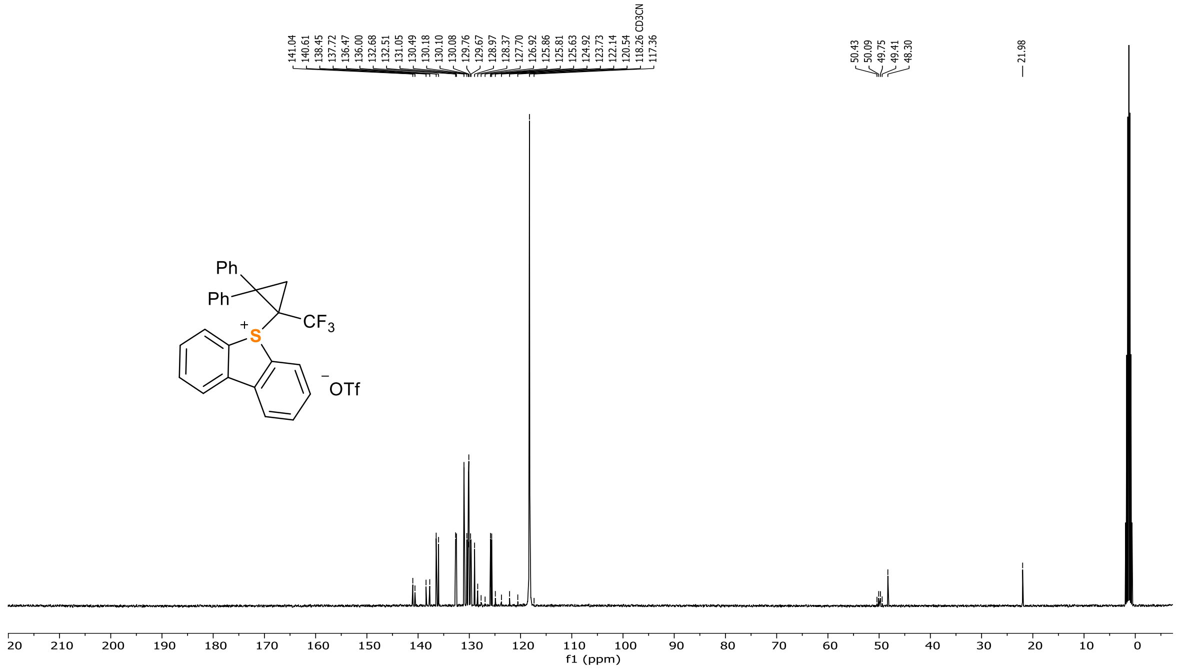

^{19}F NMR (377 MHz, CD_3CN)

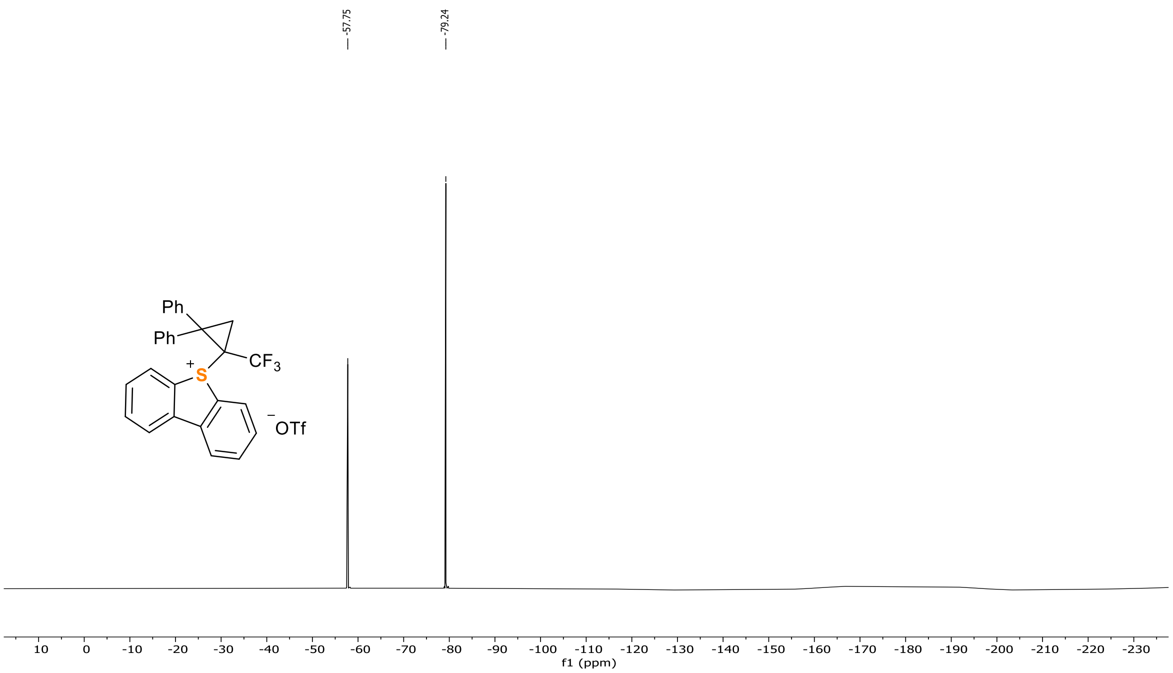

Compound **3d**:

^{1}H NMR (400 MHz, CD_3CN)

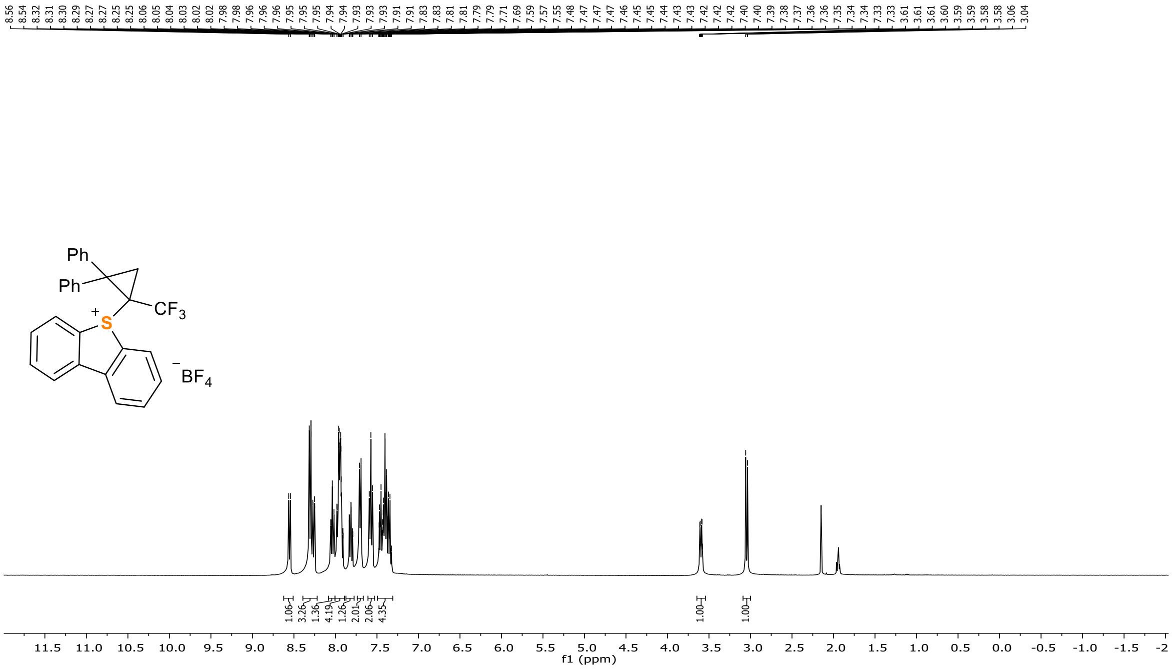

$^{13}C\{1H\}$ NMR (101 MHz, CD_3CN)

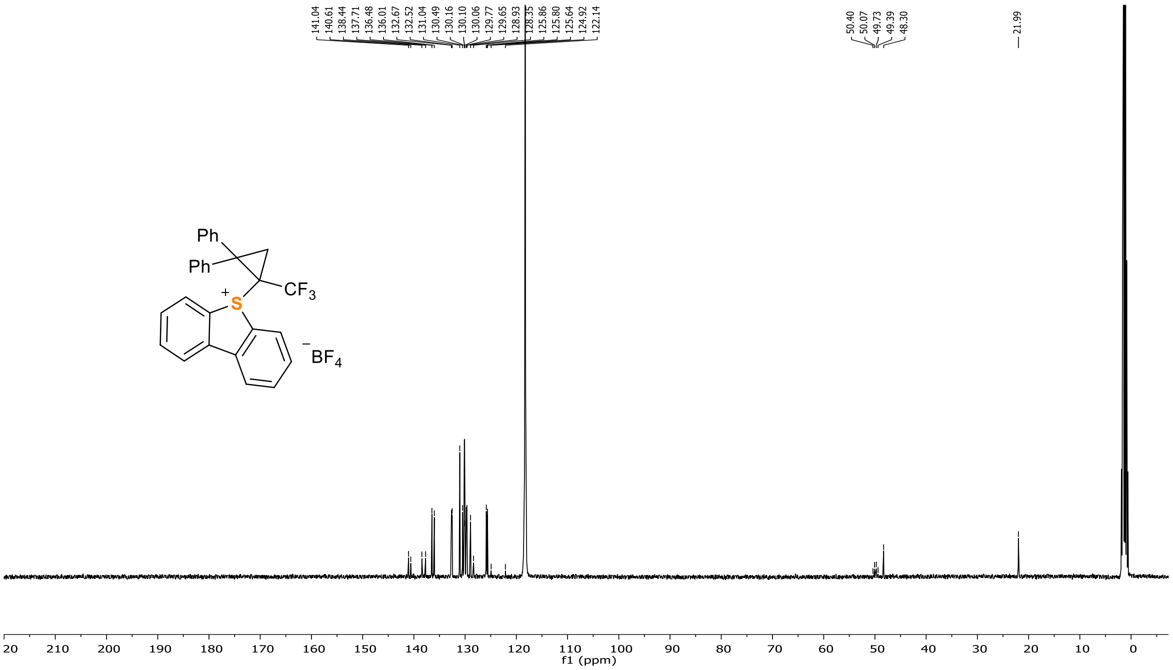

^{19}F NMR (377 MHz, CD_3CN)

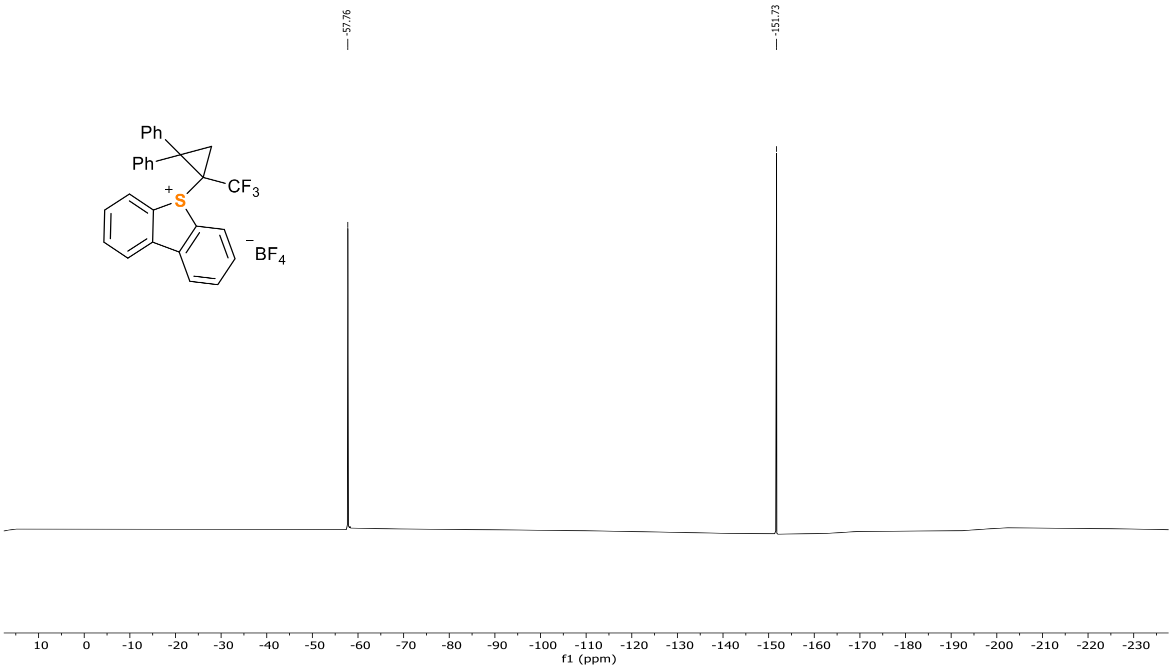

Compound **3e**:

^{1}H NMR (400 MHz, CD_3CN)

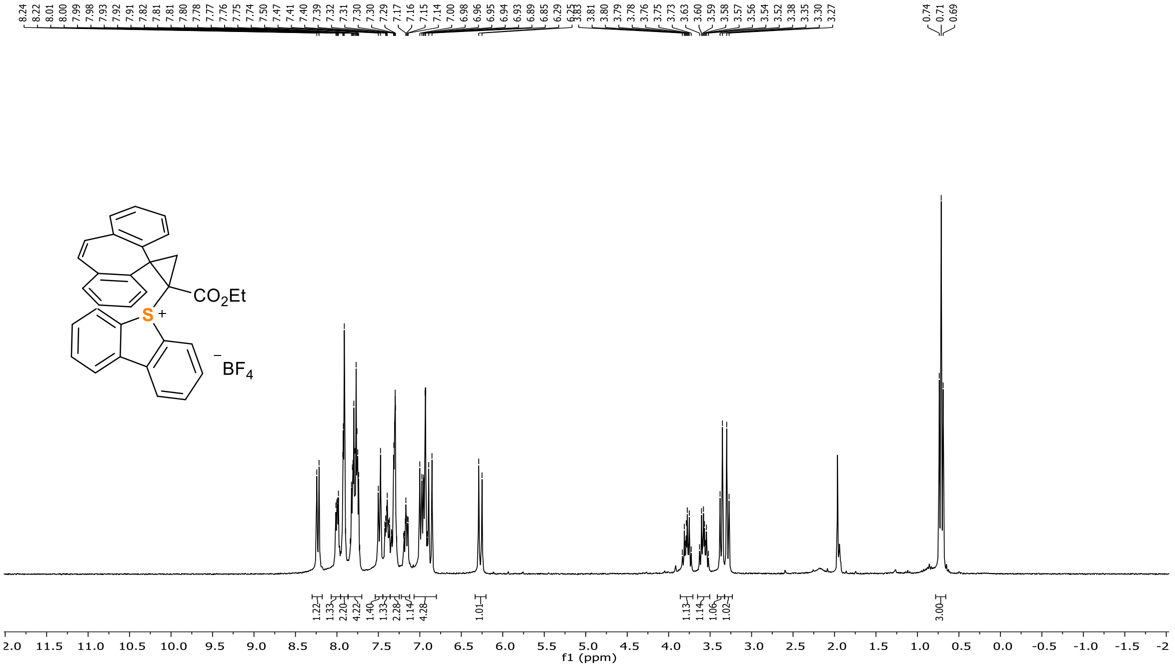

^{13}C{1H} NMR (101 MHz, CD_3CN)

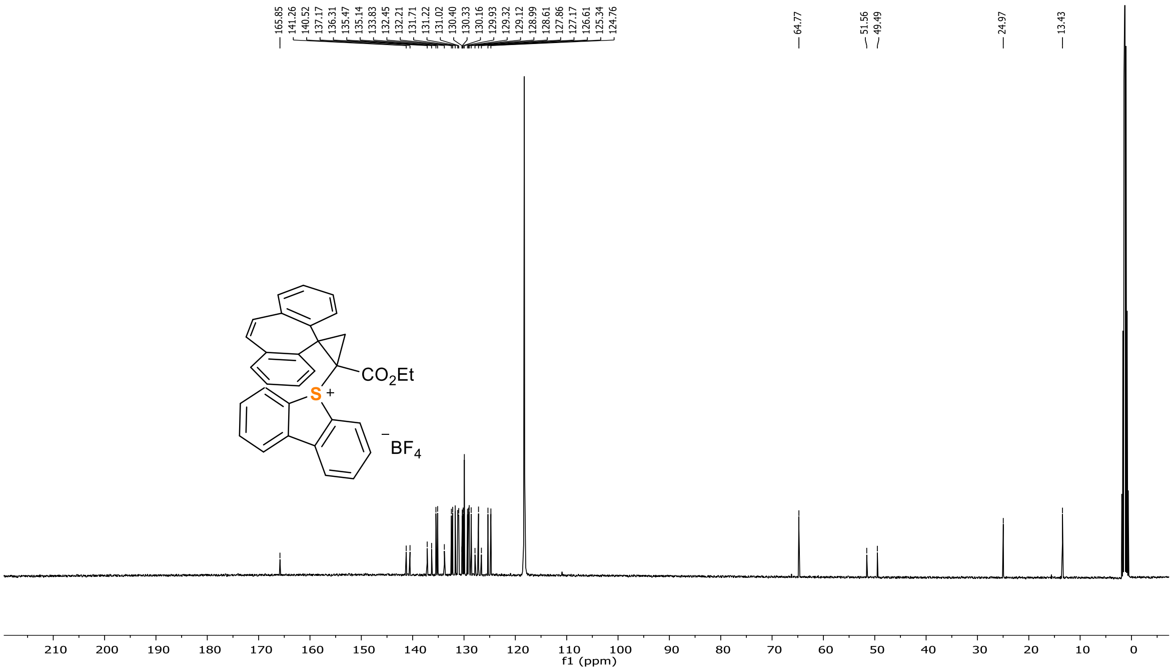

^{19}F NMR (377 MHz, CD_3CN)

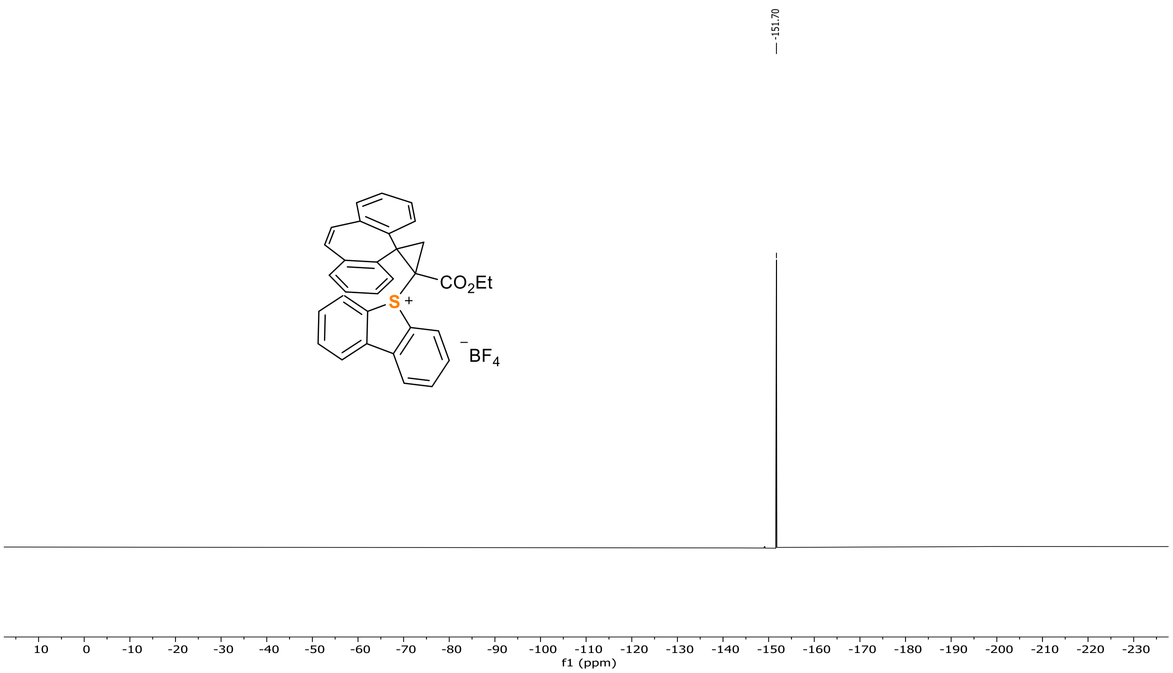

Compound **3f**:

^{1}H NMR (400 MHz, CD_3CN)

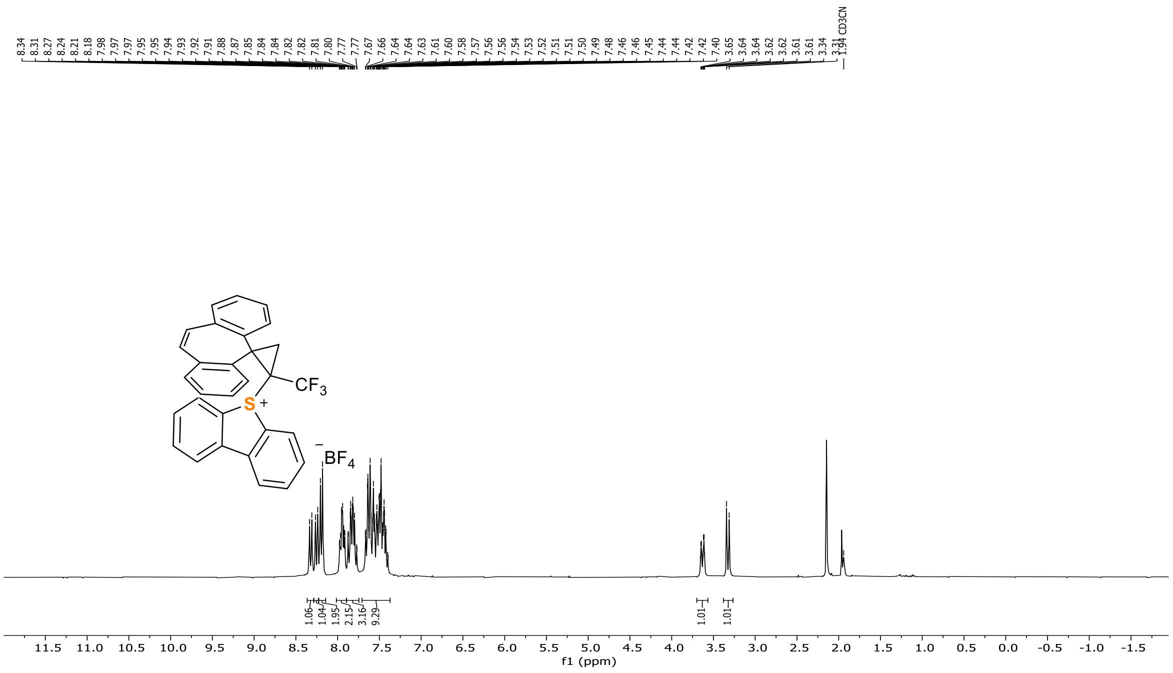

$^{13}C\{^1H\}$ NMR (101 MHz, CD_3CN)

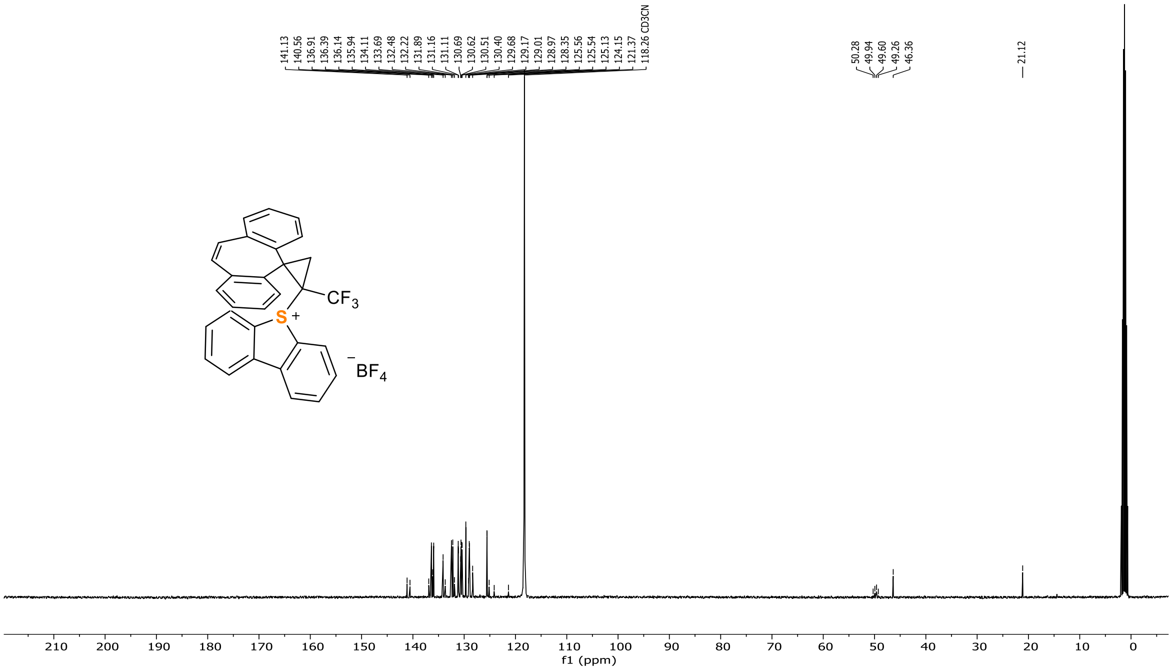

^{19}F NMR (377 MHz, CD_3CN)

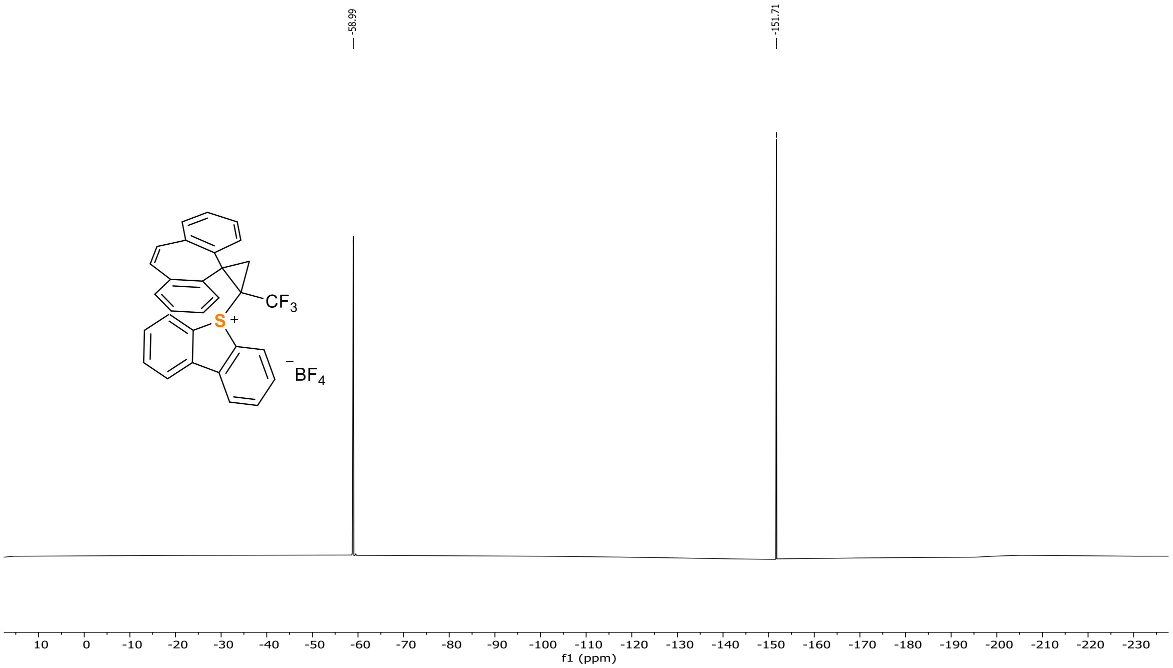

Compound **3g**:

^{1}H NMR (400 MHz, $CDCl_3$)

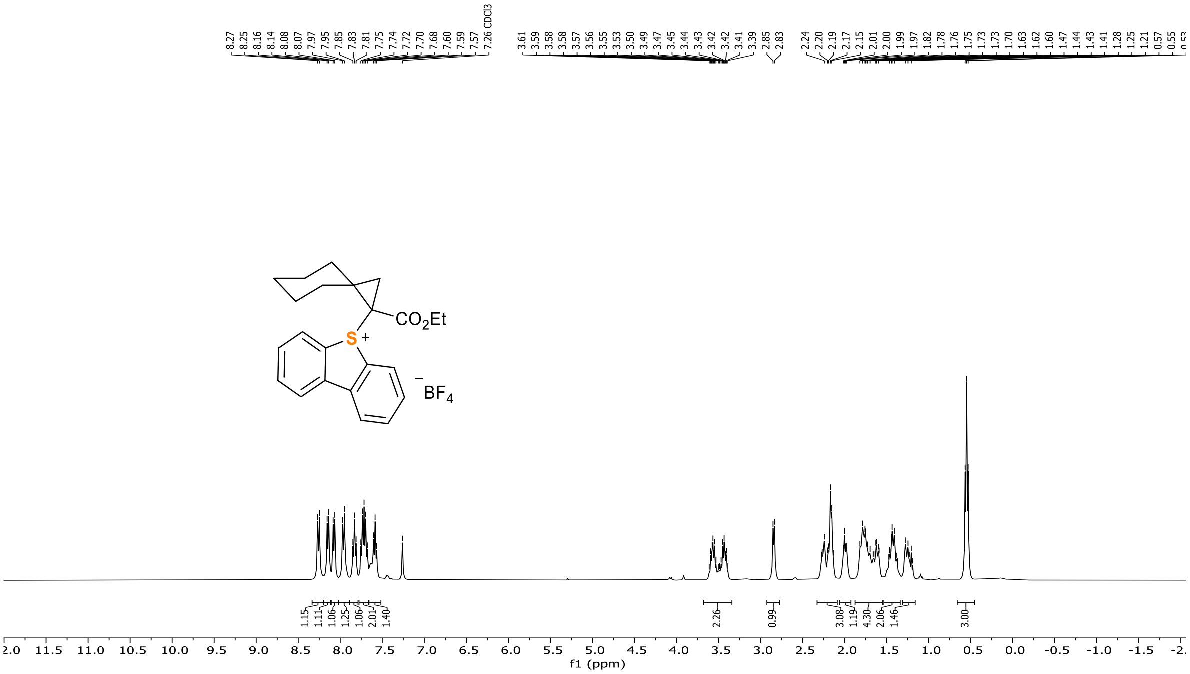

$^{13}C\{1H\}$ NMR (101 MHz, $CDCl_3$)

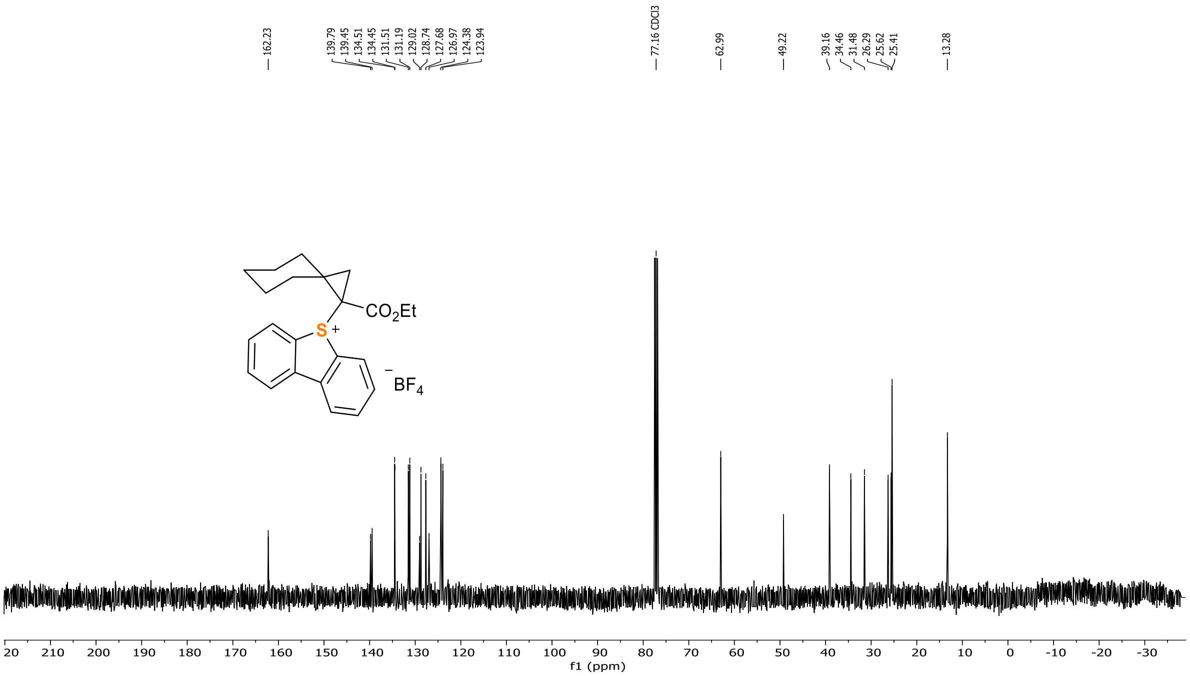

^{19}F NMR (377 MHz, $CDCl_3$)

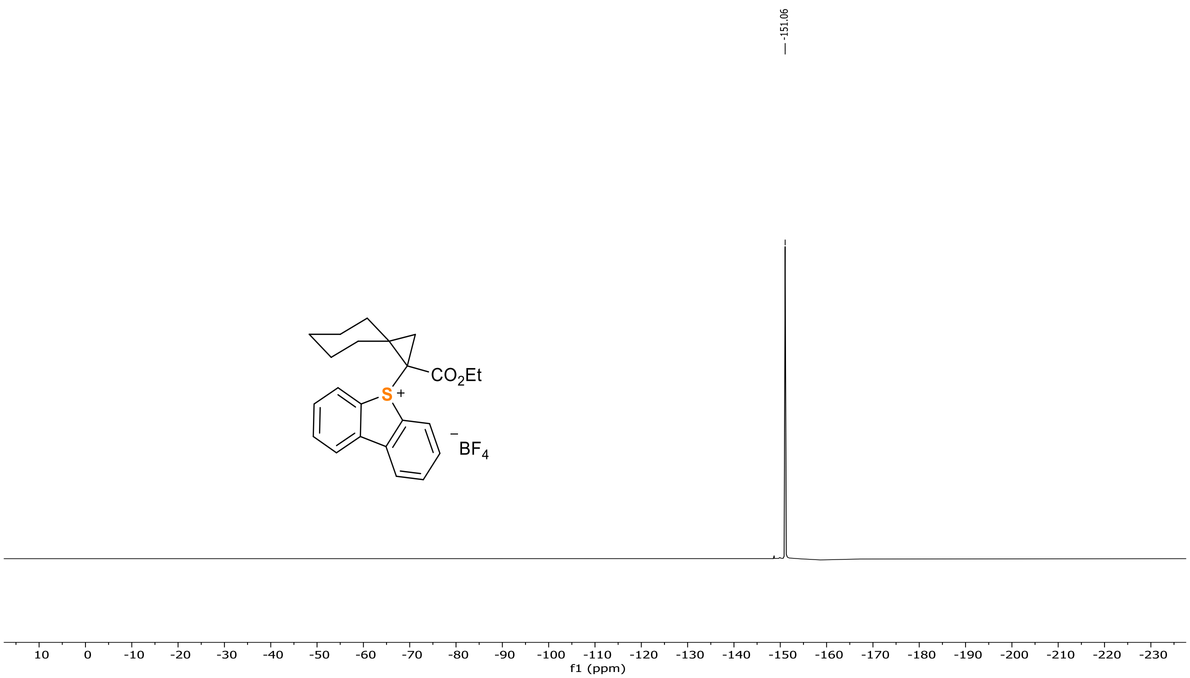

Compound **3h**:

^{1}H NMR (400 MHz, $CDCl_3$)

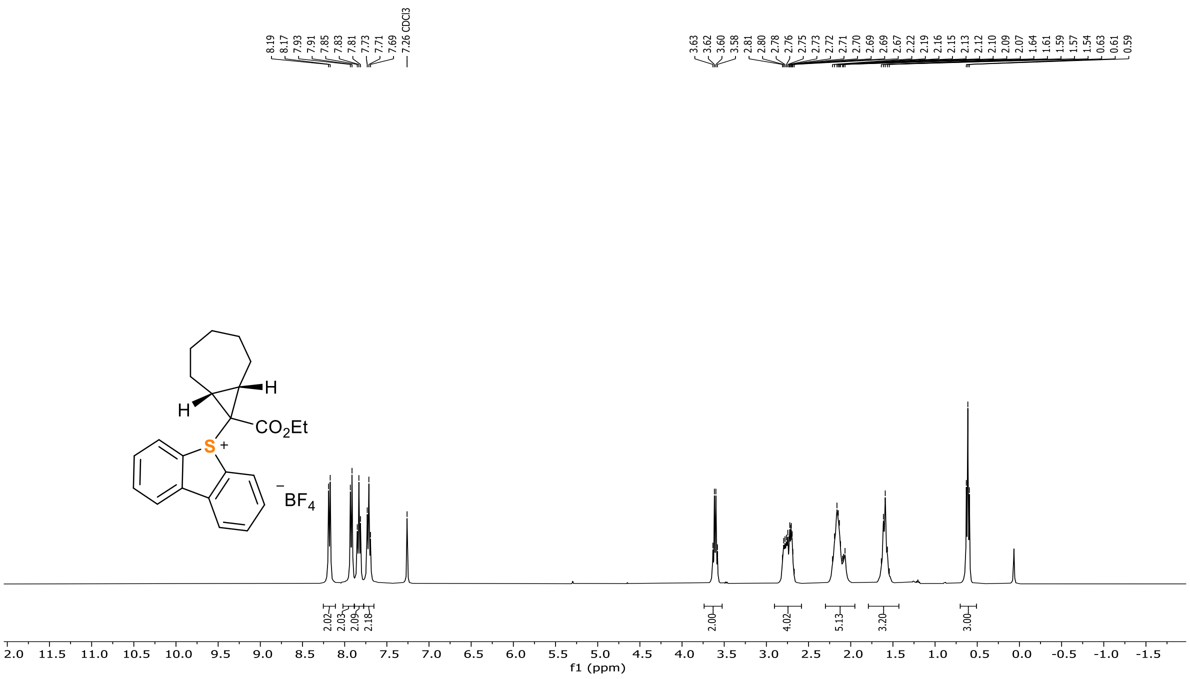

$^{13}C\{1H\}$ NMR (101 MHz, $CDCl_3$)

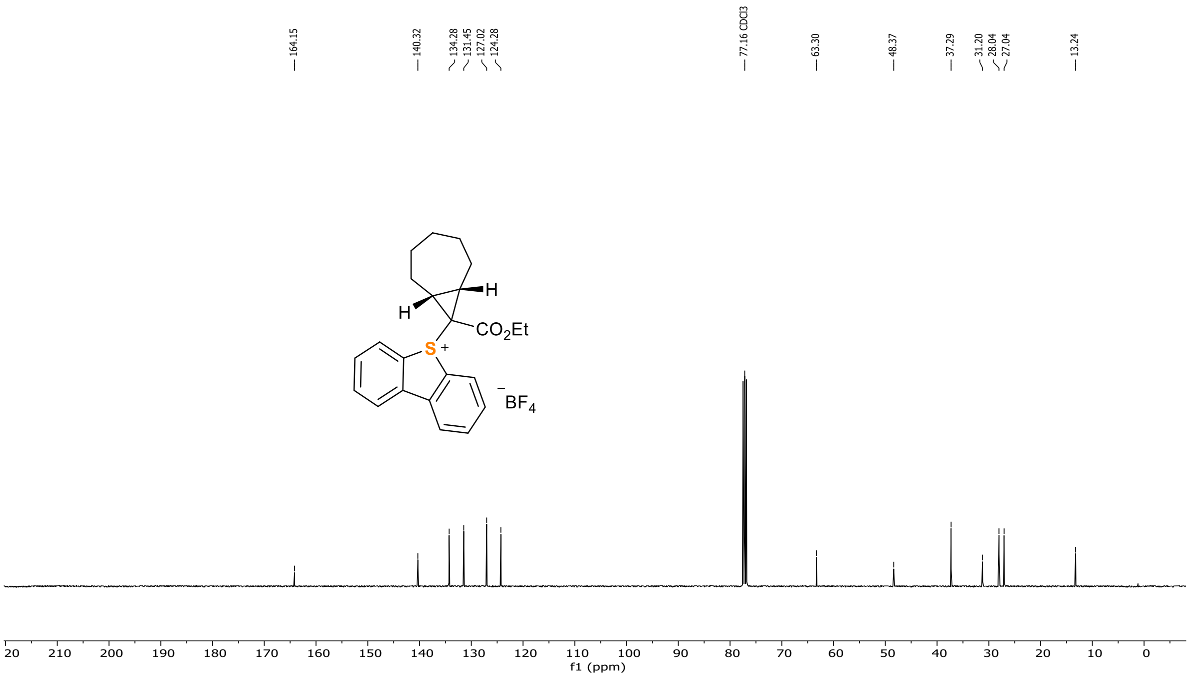

^{19}F NMR (377 MHz, $CDCl_3$)

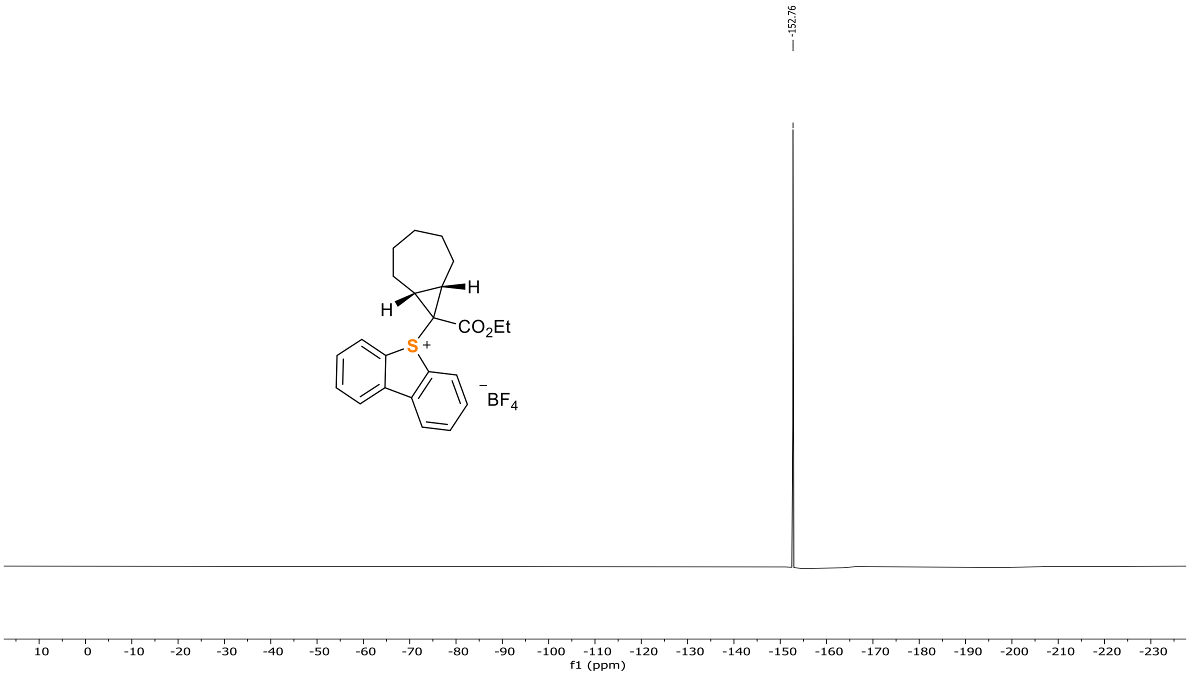

Compound **4b**:

^{1}H NMR (300 MHz, $CDCl_3$)

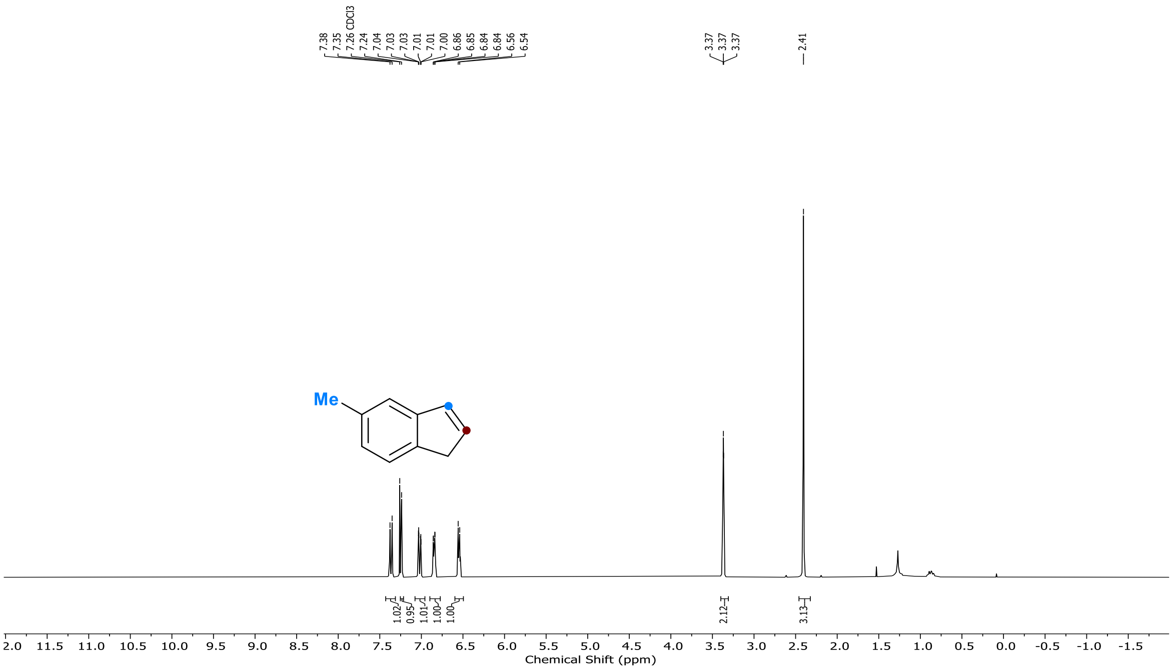

^{13}C{1H} NMR (75 MHz, $CDCl_3$)

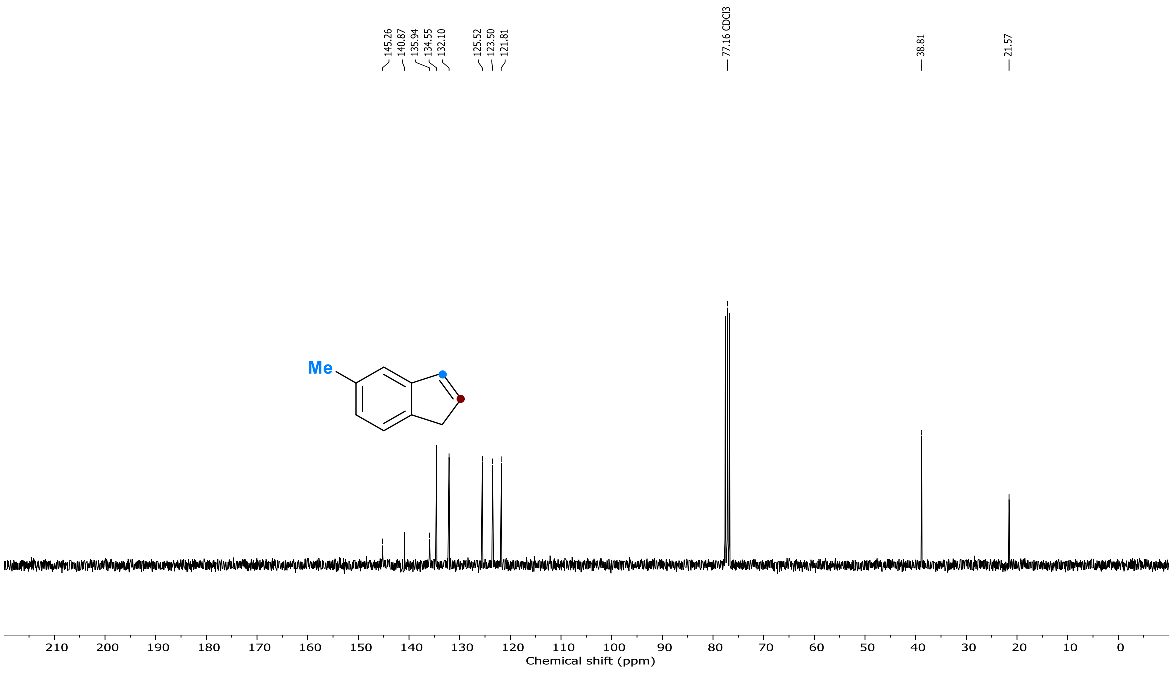

Compound **4c**:

^{1}H NMR (300 MHz, $CDCl_3$)

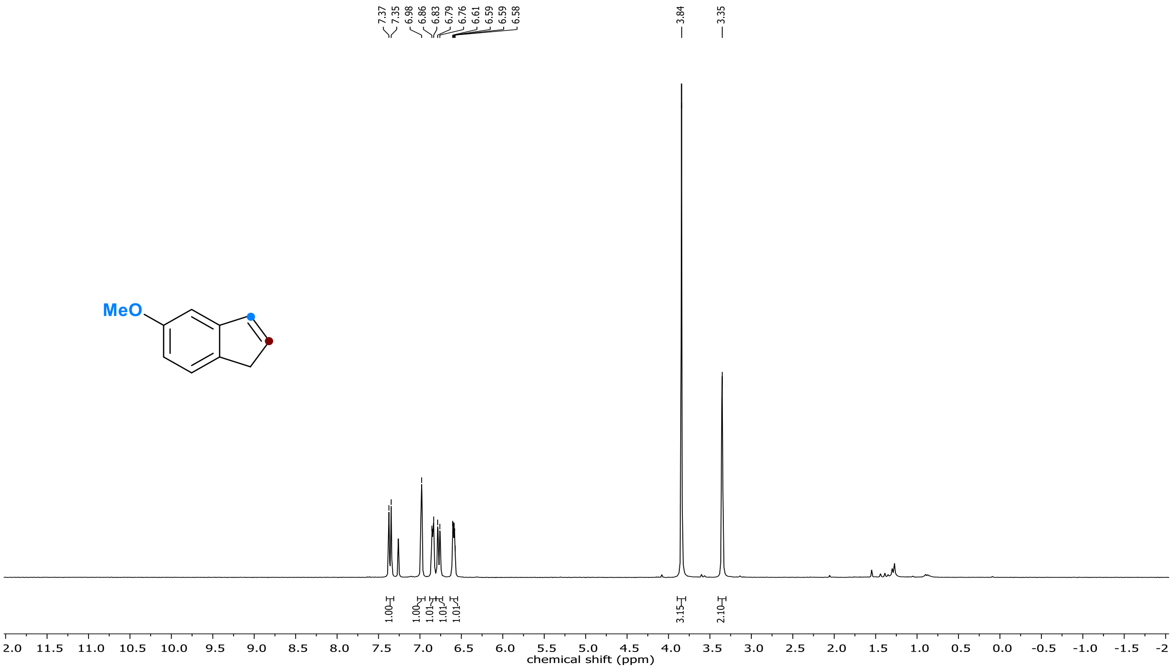

^{13}C{1H} NMR (75 MHz, $CDCl_3$)

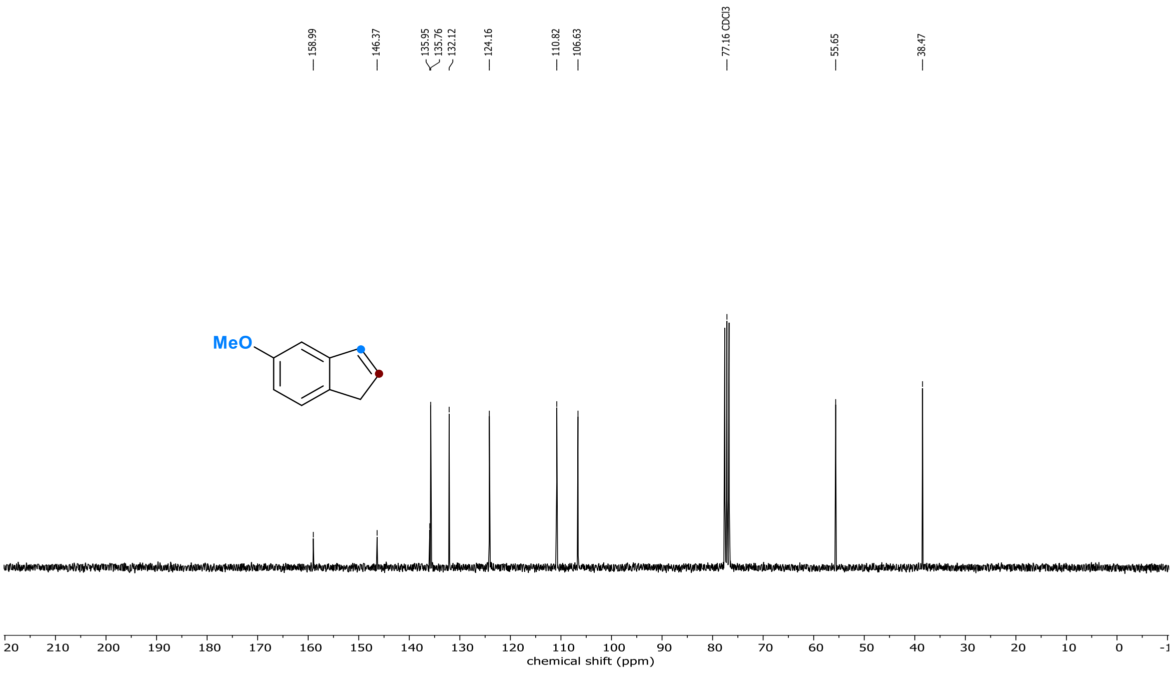

Compound **4d**:

^{1}H NMR (300 MHz, $CDCl_3$)

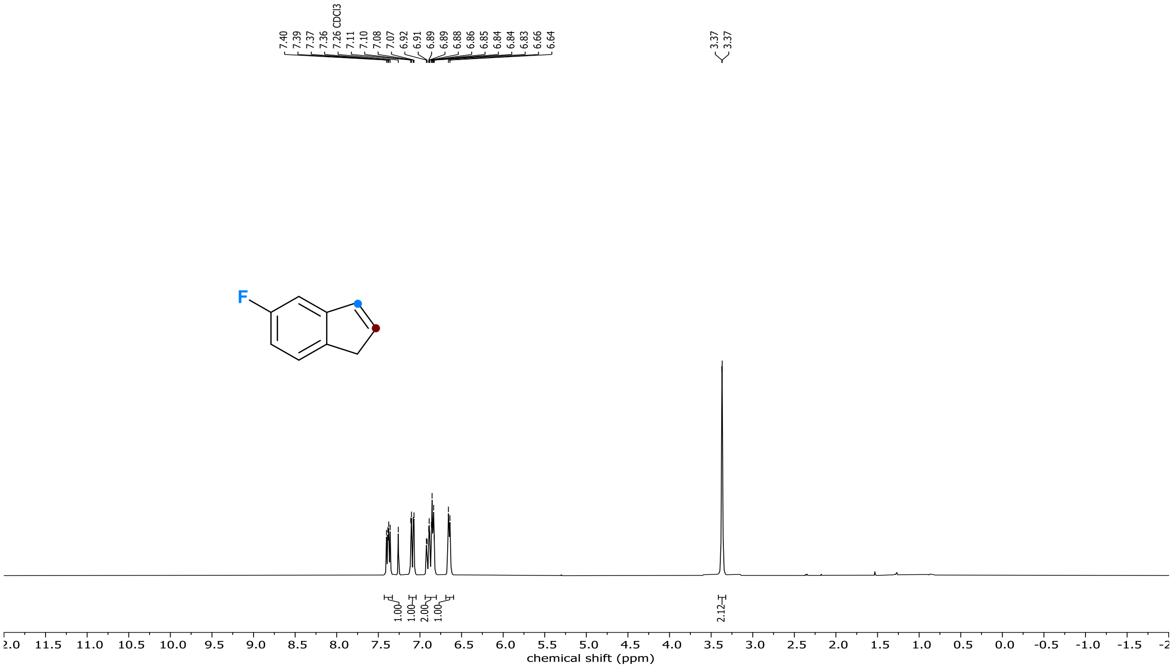

$^{13}C\{1H\}$ NMR (75 MHz, $CDCl_3$)

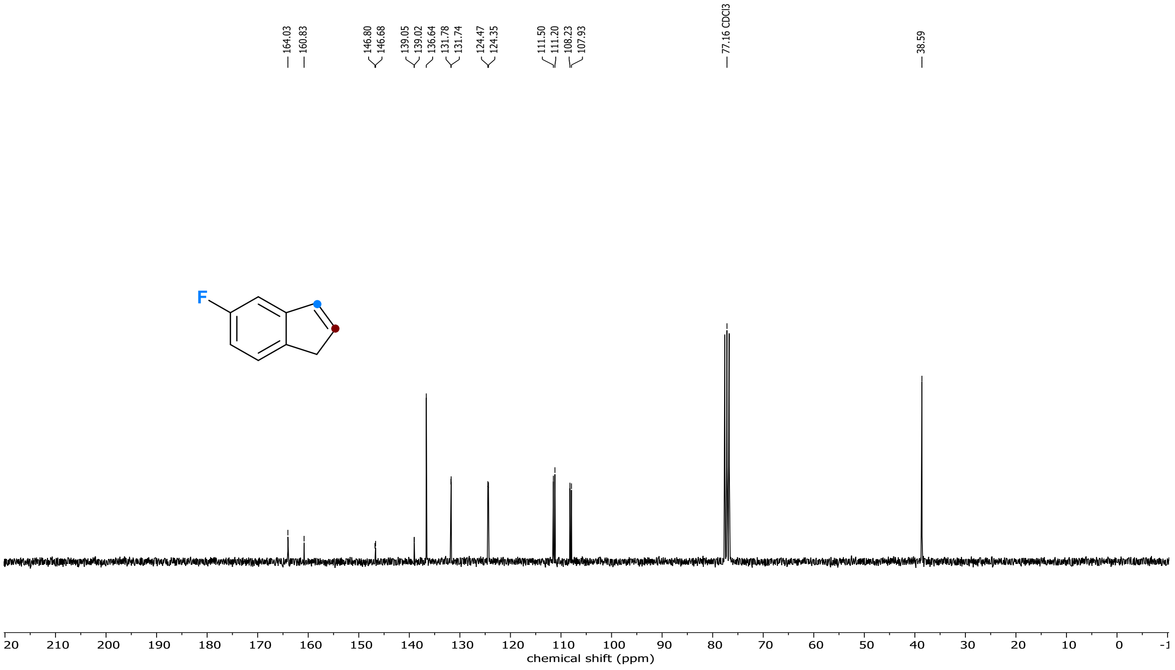

^{19}F NMR (282 MHz, $CDCl_3$)

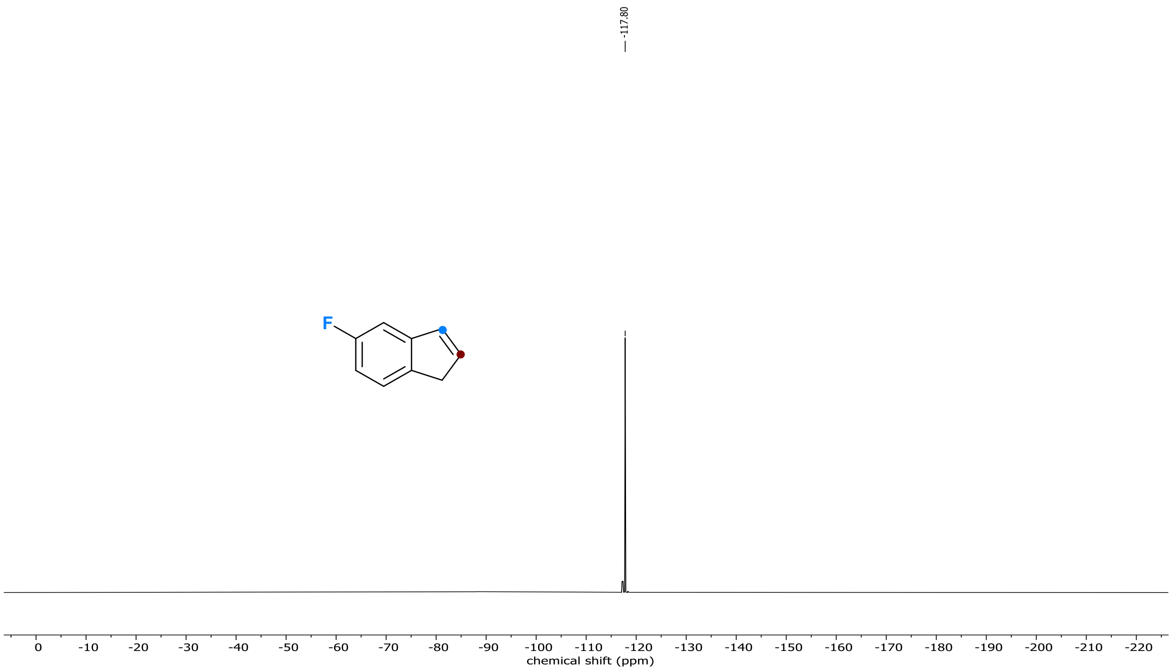

Compound **4e**:

^{1}H NMR (300 MHz, $CDCl_3$)

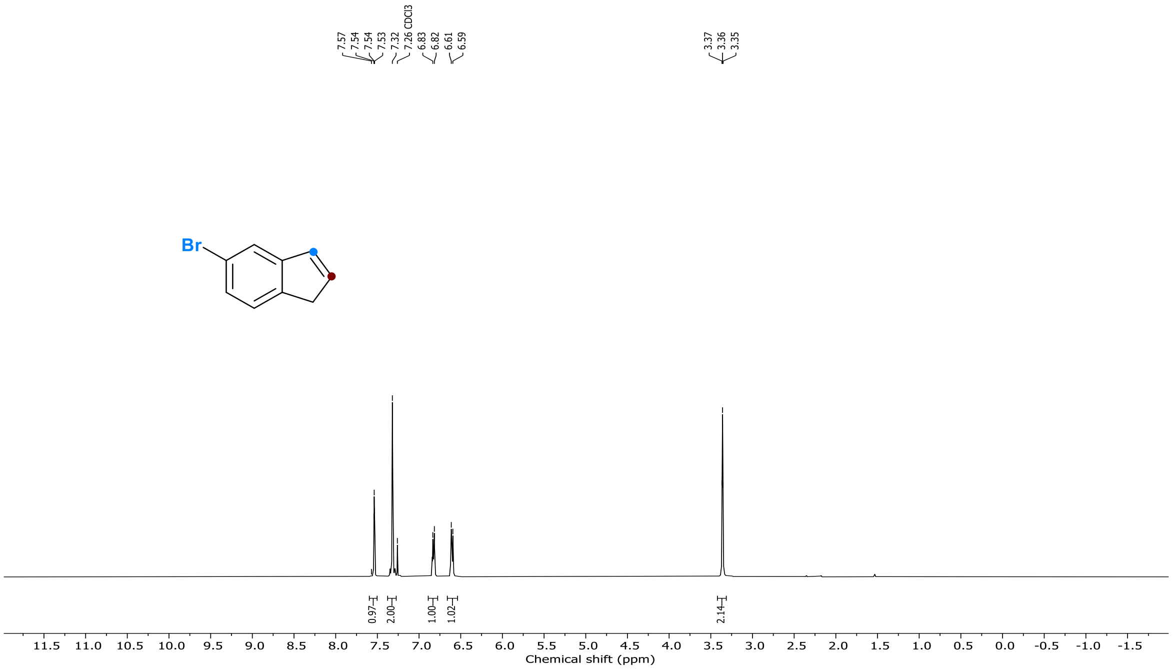

$^{13}C\{1H\}$ NMR (75 MHz, $CDCl_3$)

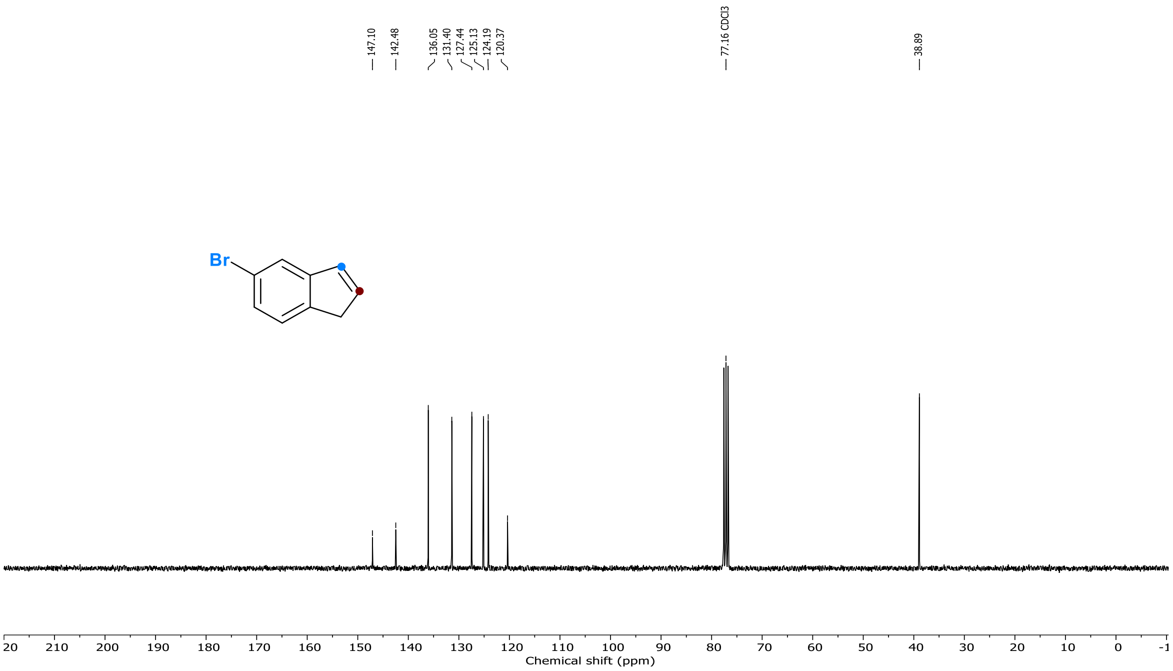

Compound **S14**:

^{1}H NMR (300 MHz, $CDCl_3$)

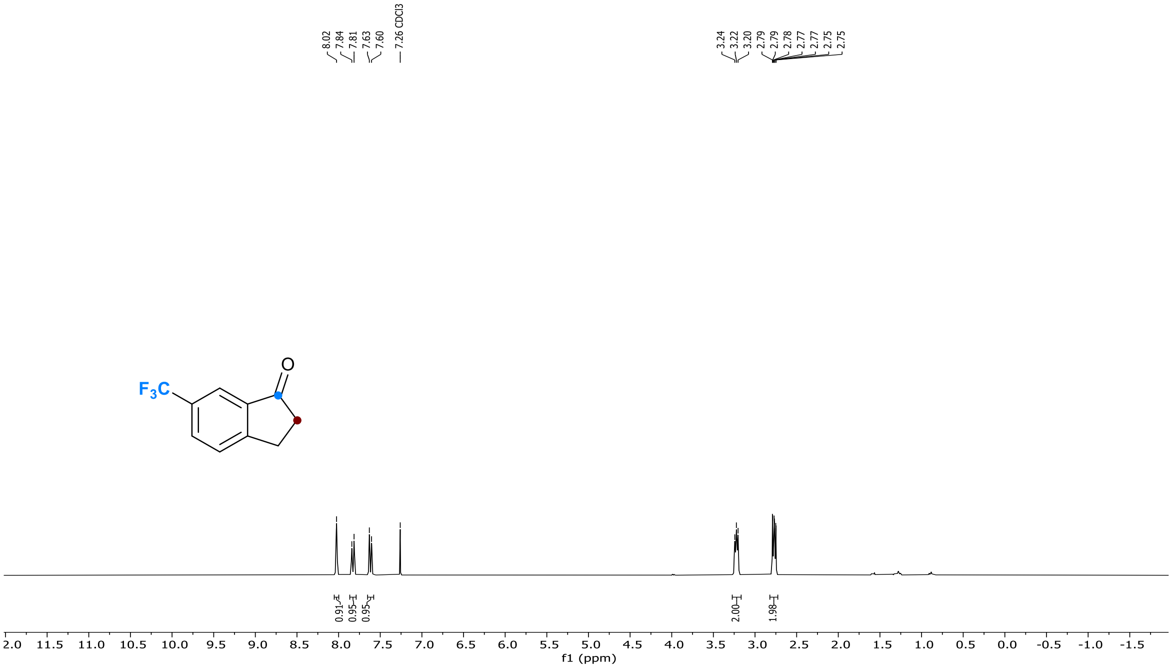

$^{13}C\{1H\}$ NMR (75 MHz, $CDCl_3$)

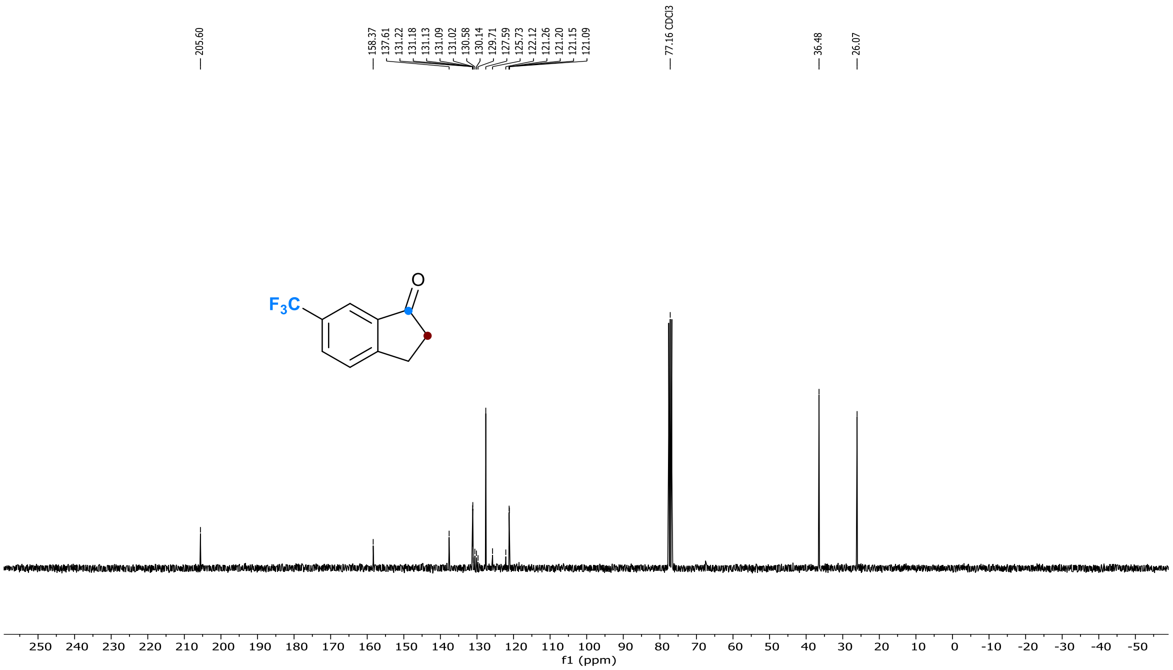

^{19}F NMR (282 MHz, $CDCl_3$)

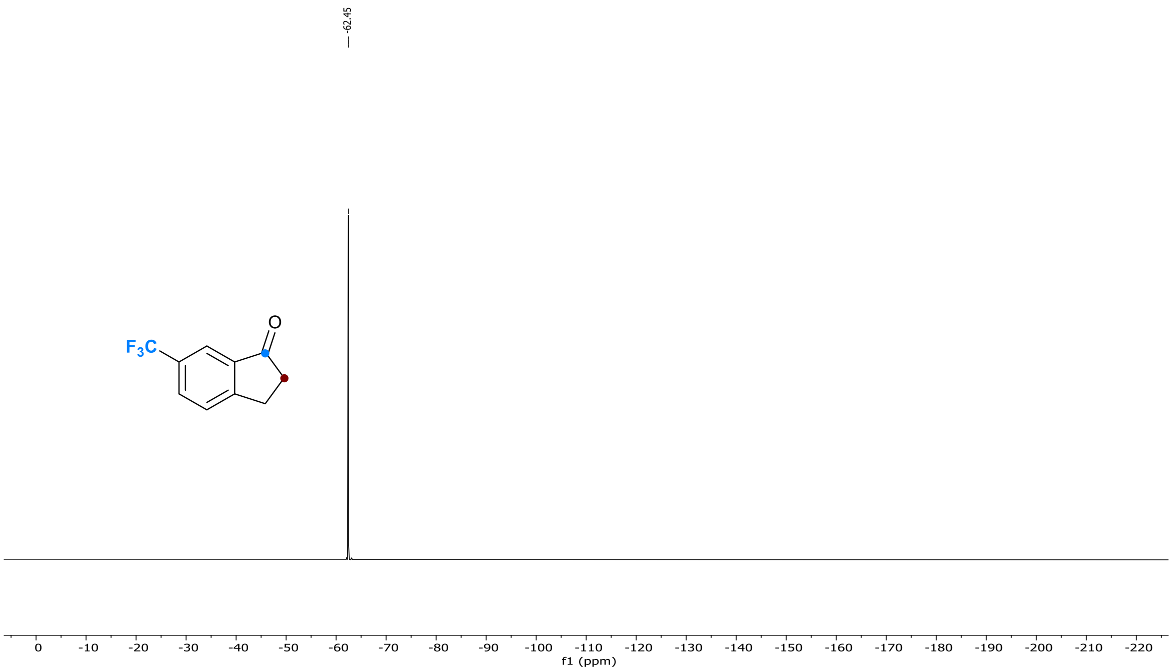

Compound **4f**:

^{1}H NMR (400 MHz, $CDCl_3$)

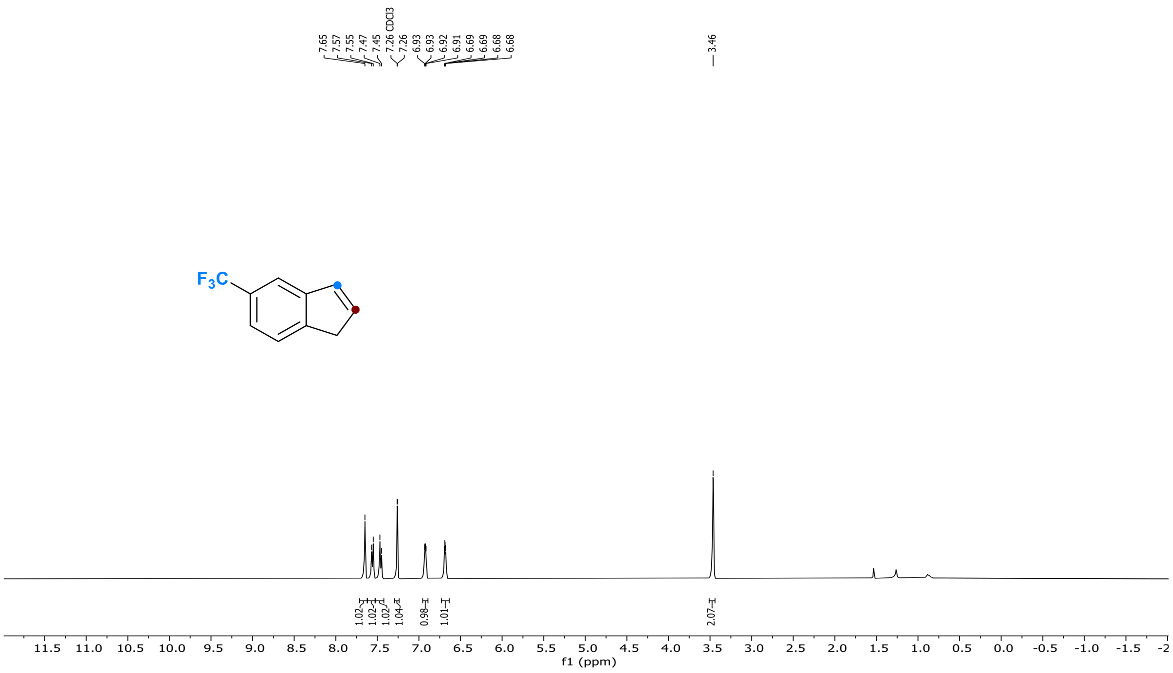

^{13}C{1H} NMR (101 MHz, $CDCl_3$)

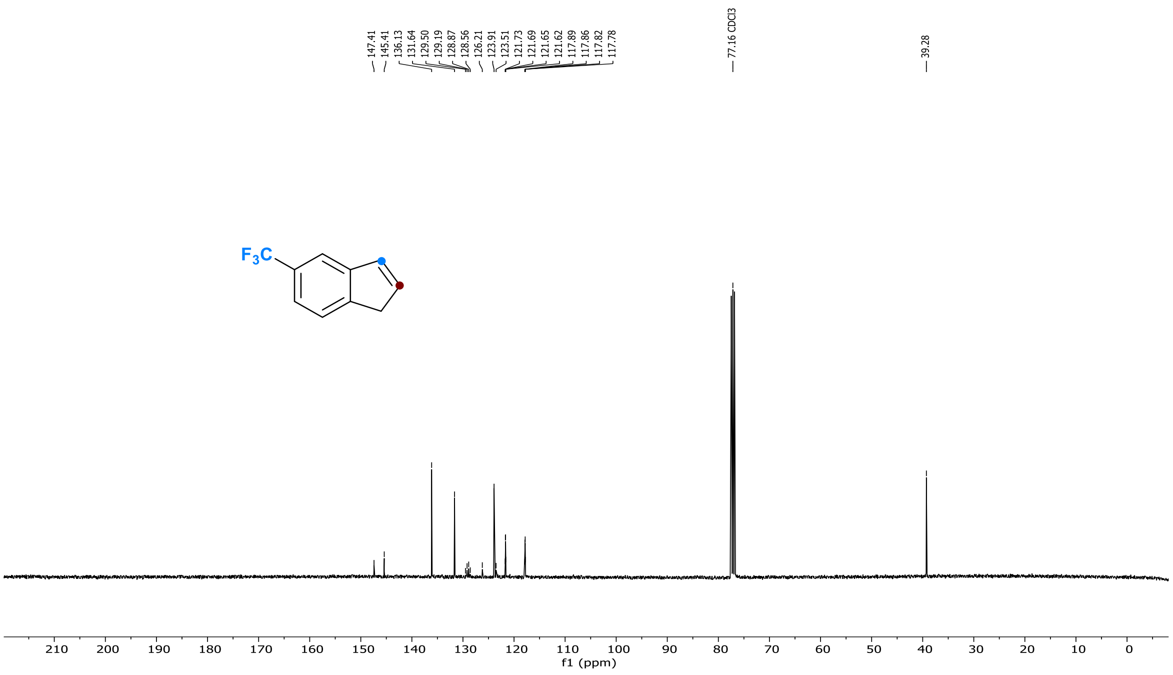

^{19}F NMR (377 MHz, $CDCl_3$)

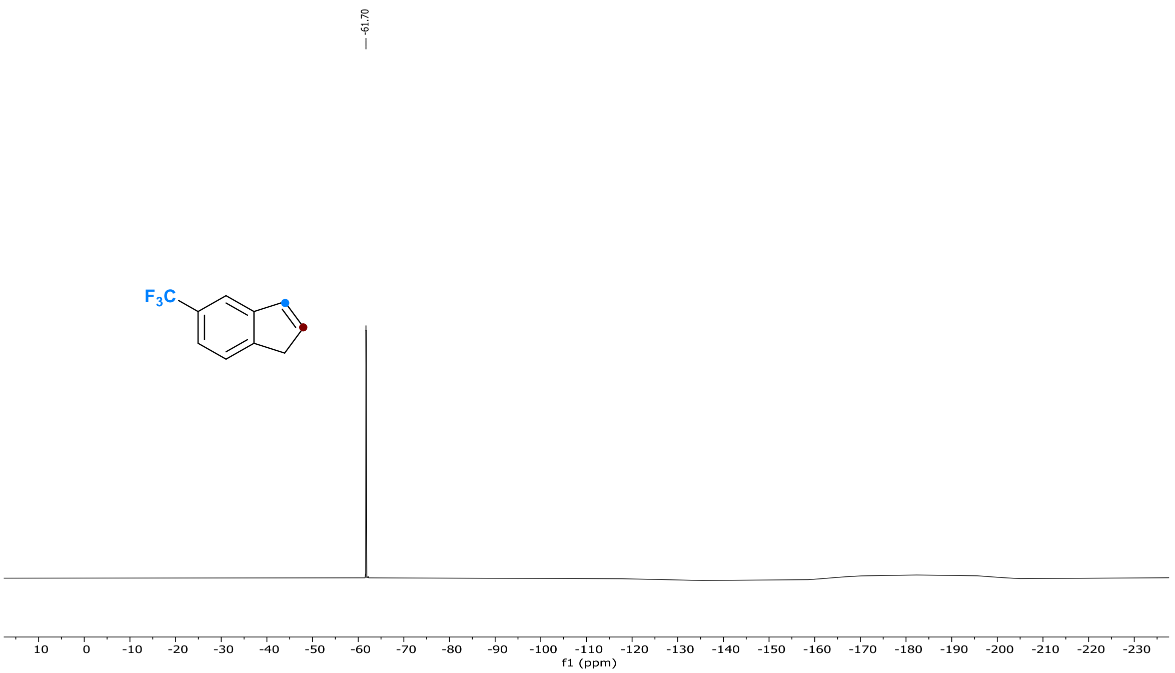

Compoound **4g**:

^{1}H NMR (300 MHz, $CDCl_3$)

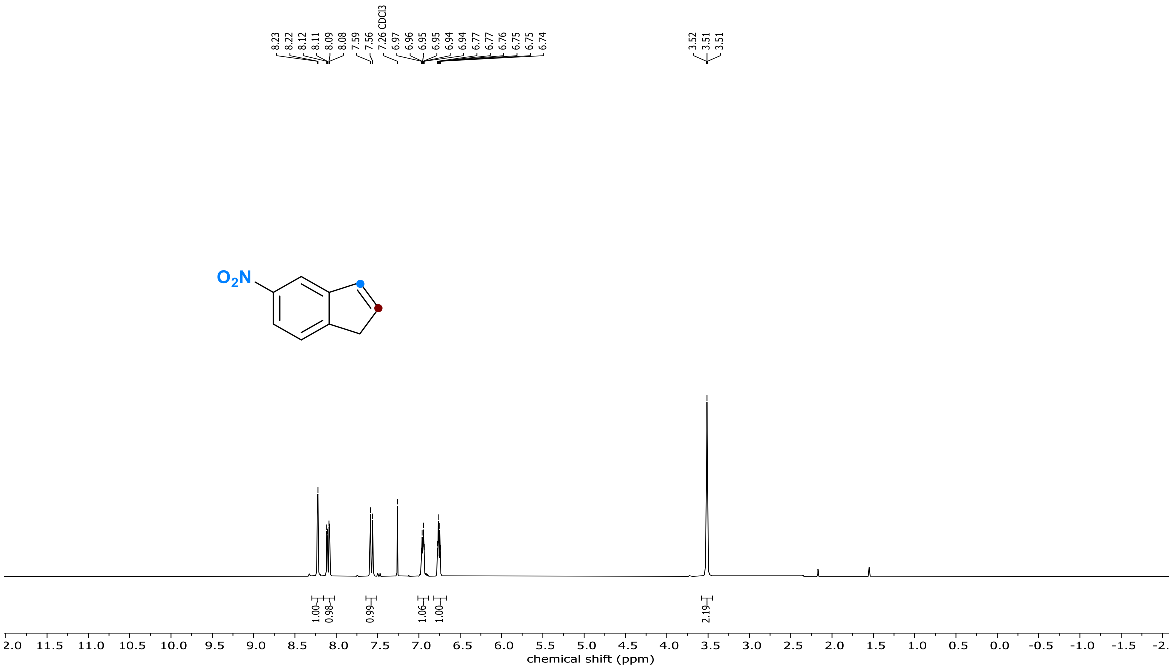

$^{13}C\{1H\}$ NMR (75 MHz, $CDCl_3$)

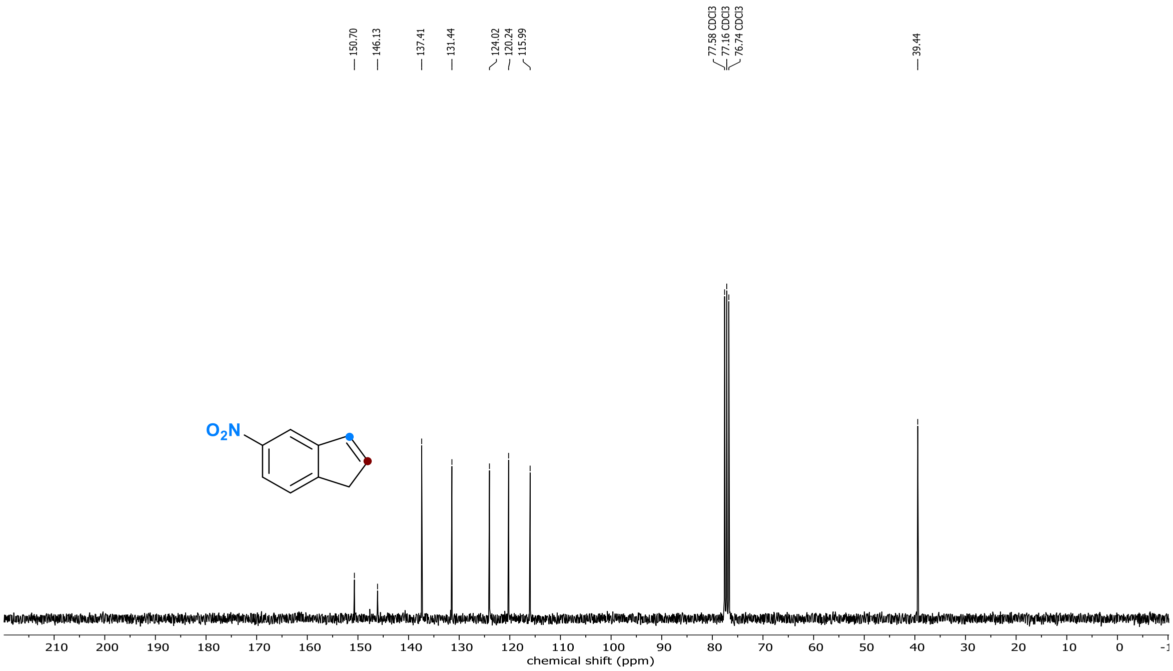

Compound **4i**:

^1H NMR (300 MHz, $CDCl_3$)

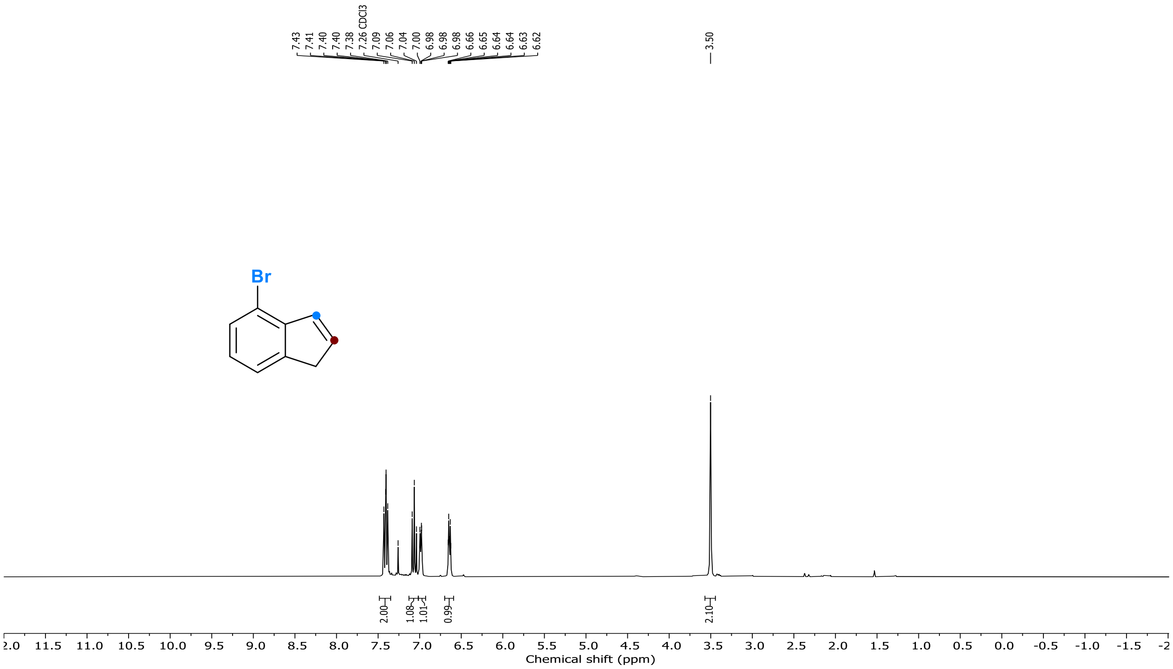

$^{13}C\{1H\}$ NMR (75 MHz, $CDCl_3$)

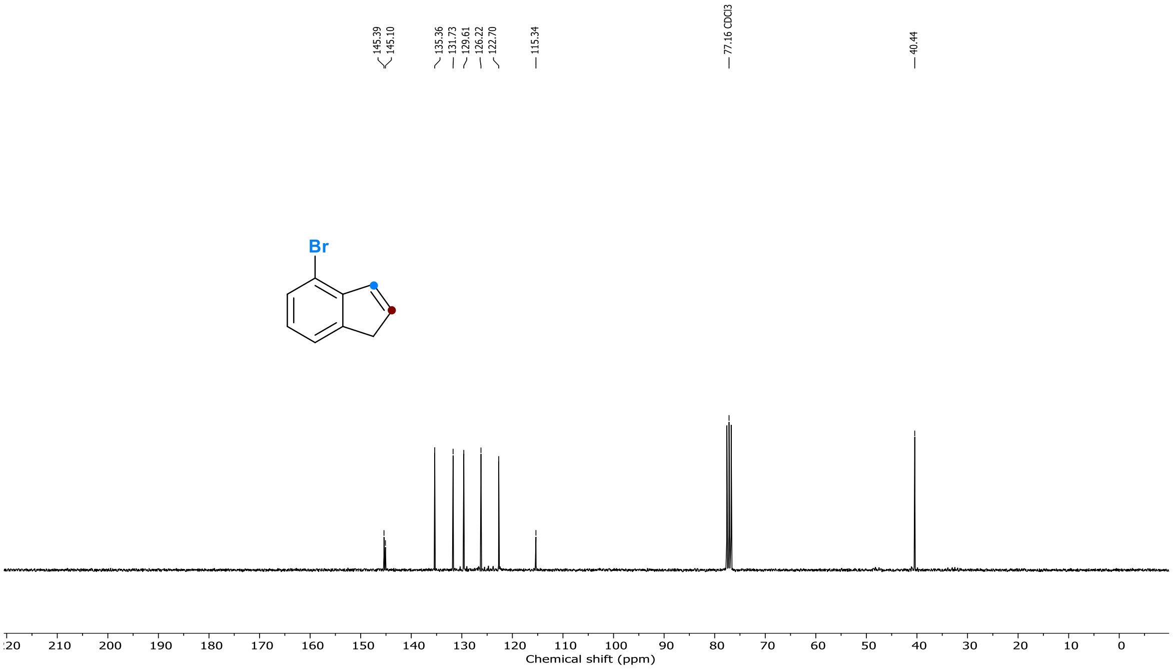

Compound **4k**:

^1H NMR (400 MHz, $CDCl_3$)

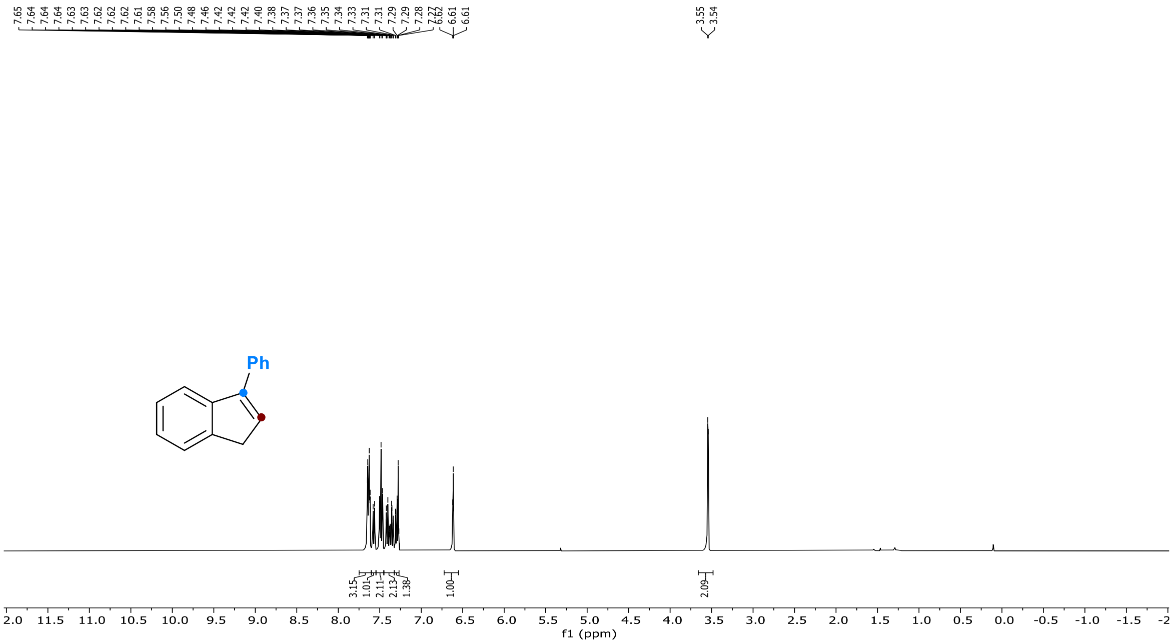

$^{13}C\{^1H\}$ NMR (101 MHz, $CDCl_3$)

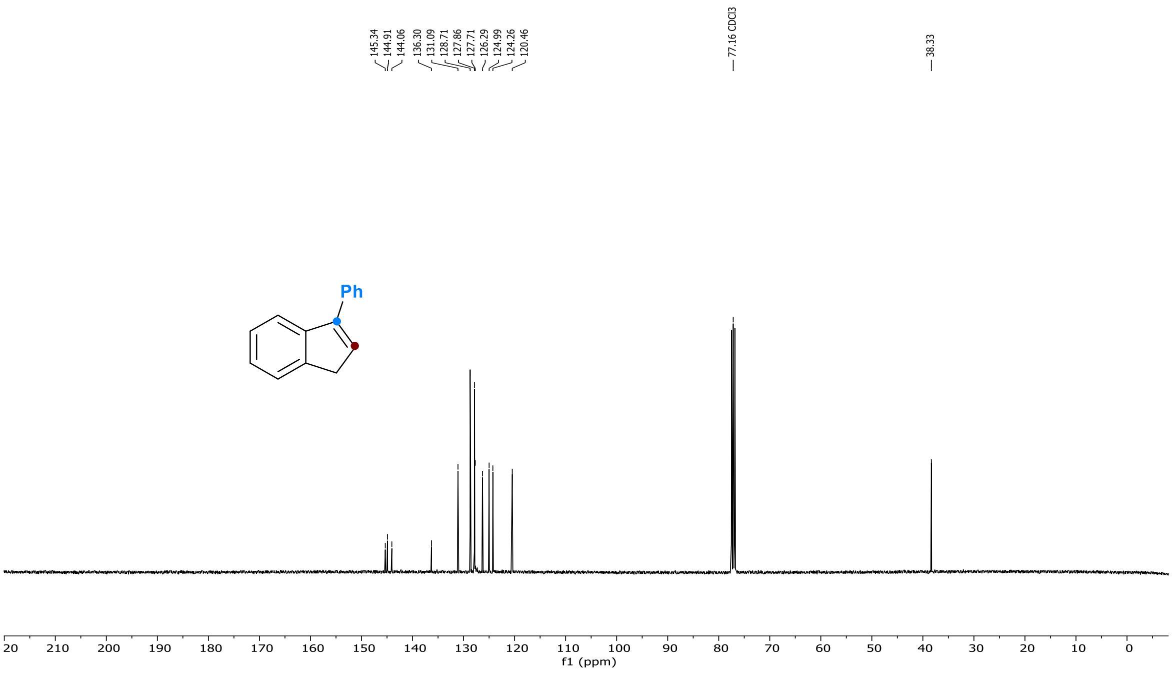

Compound **4l**:

^1H NMR (400 MHz, $CDCl_3$)

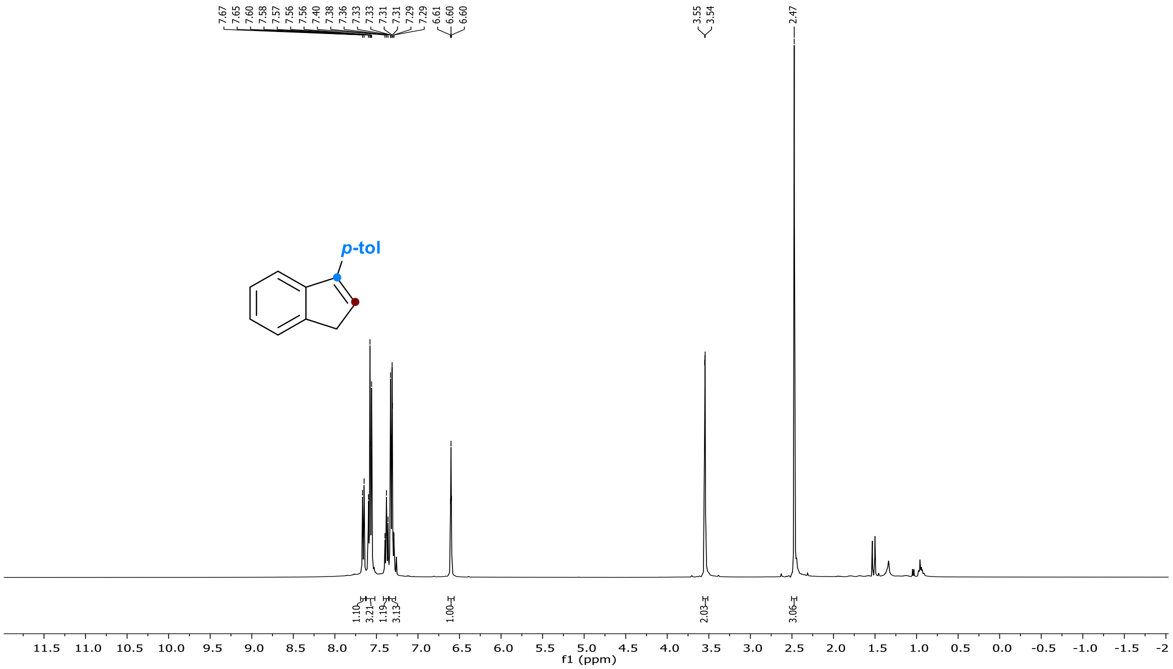

$^{13}C\{1H\}$ NMR (101 MHz, $CDCl_3$)

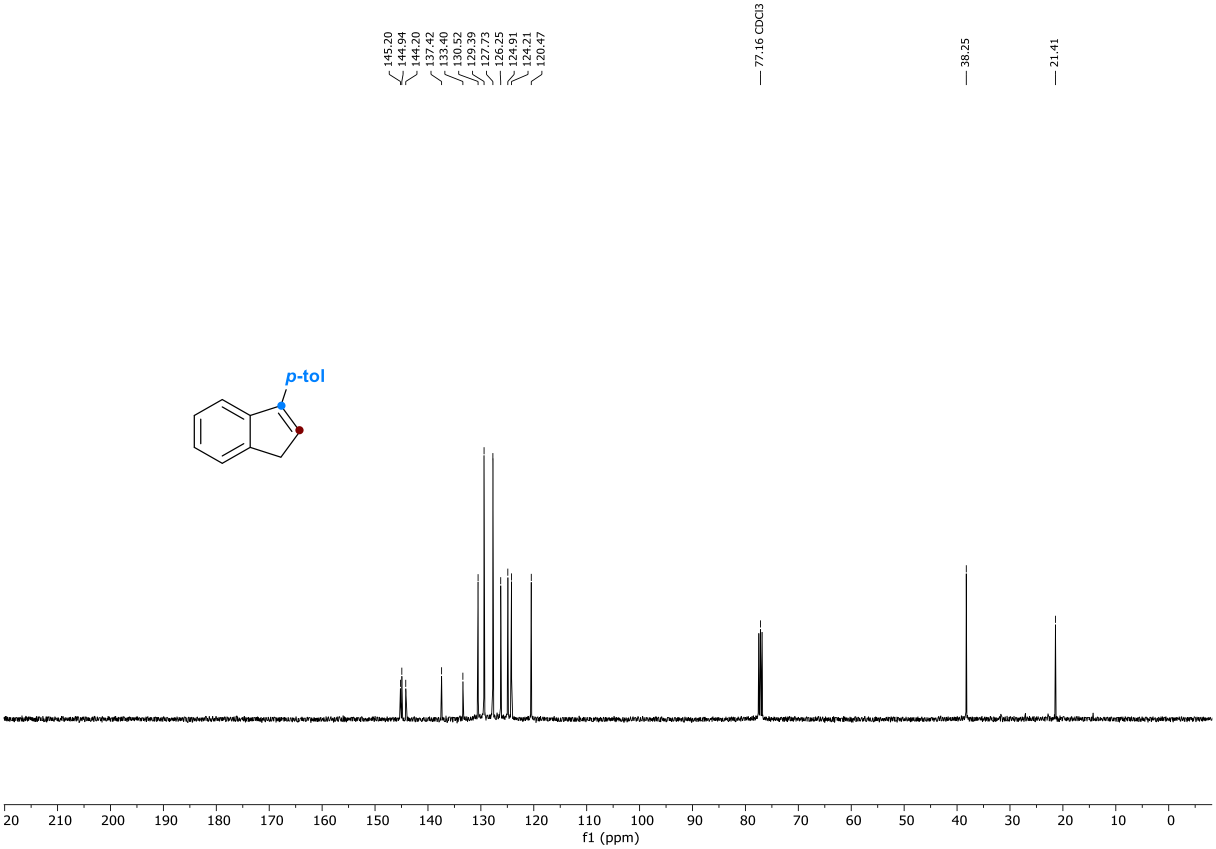

Compound **4m**:

^{1}H NMR (400 MHz, $CDCl_3$)

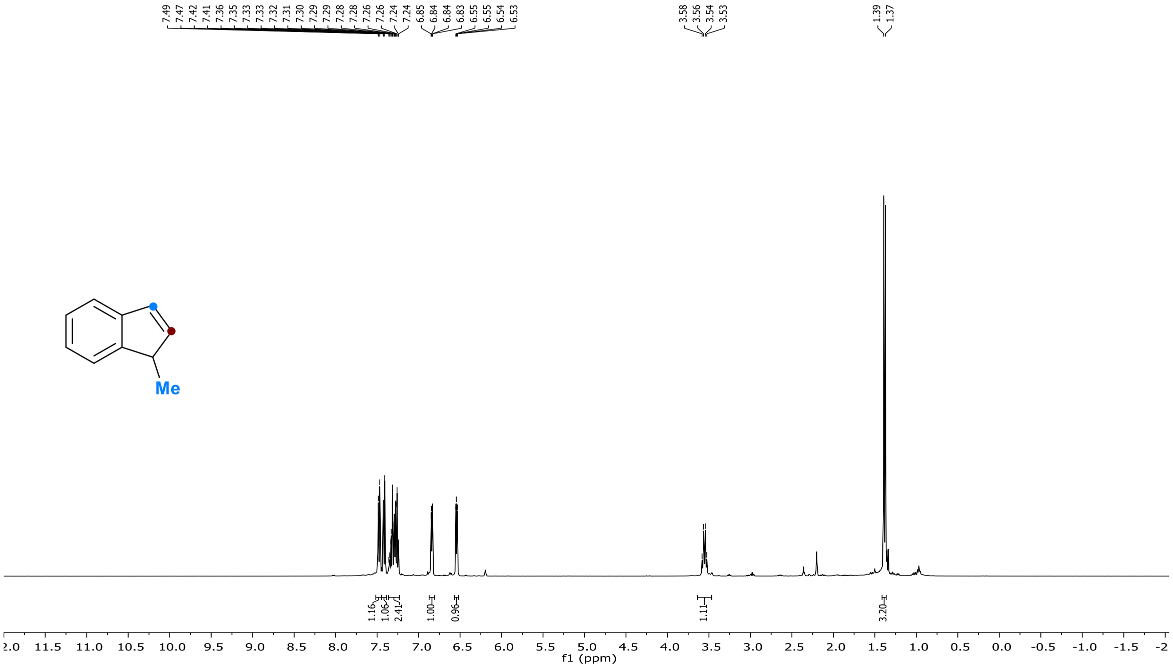

$^{13}C\{1H\}$ NMR (101 MHz, $CDCl_3$)

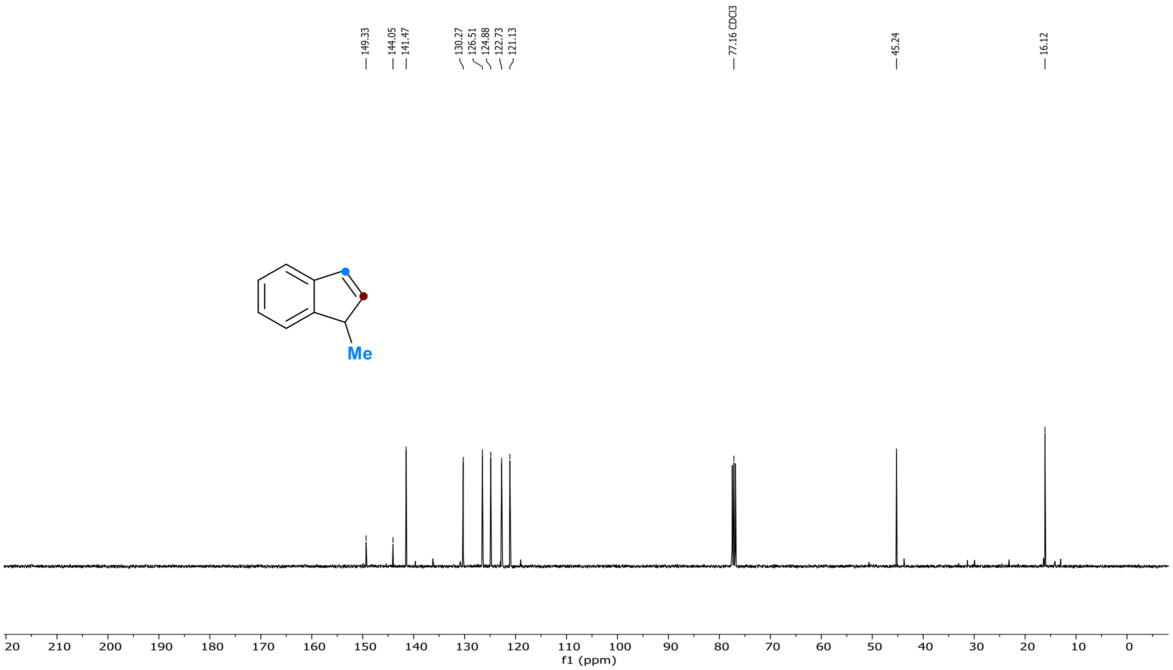

Compound **4n**:

^1H NMR (400 MHz, $CDCl_3$)

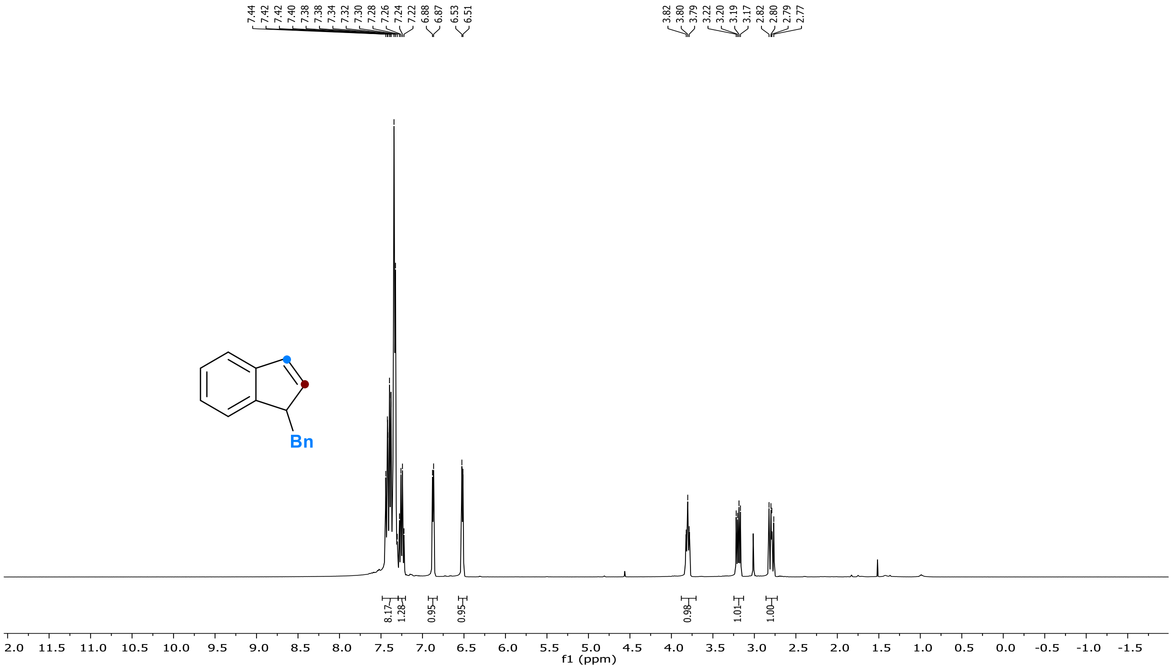

$^{13}C\{1H\}$ NMR (101 MHz, $CDCl_3$)

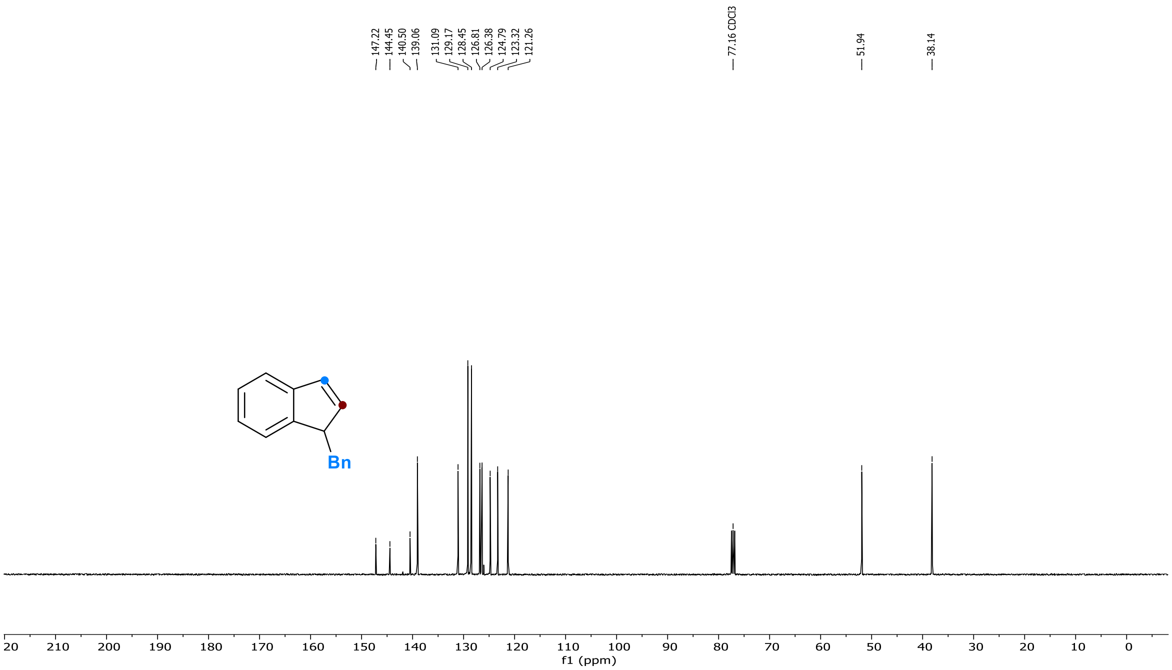

Compound **4o**:

^{1}H NMR (400 MHz, $CDCl_3$)

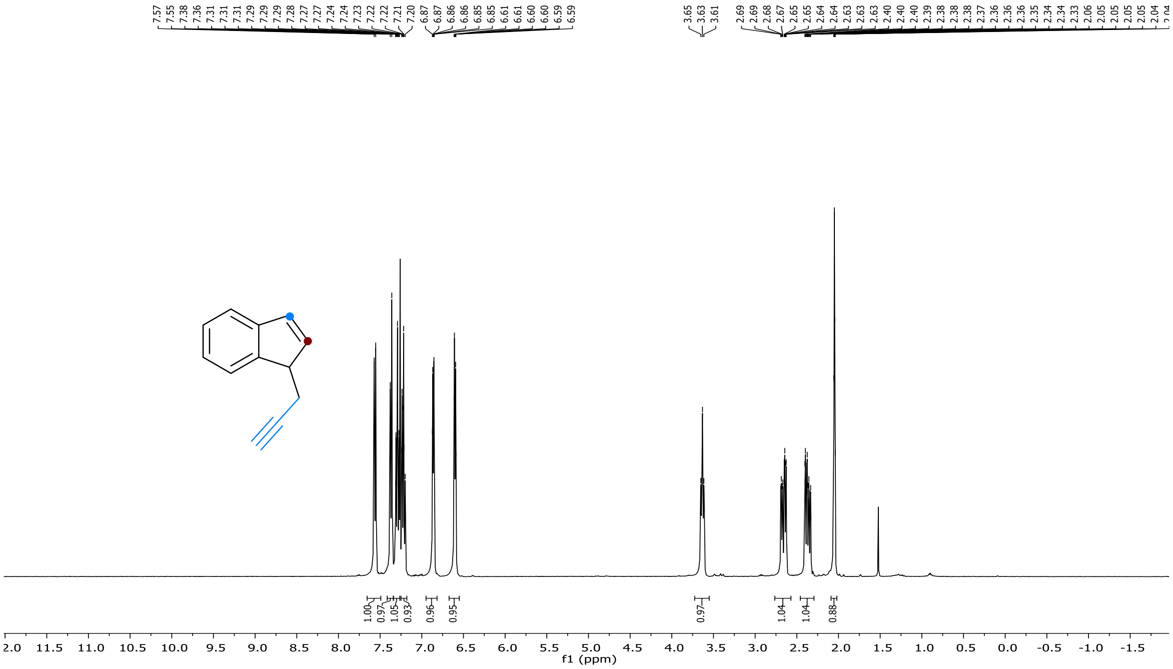

$^{13}C\{1H\}$ NMR (101 MHz, $CDCl_3$)

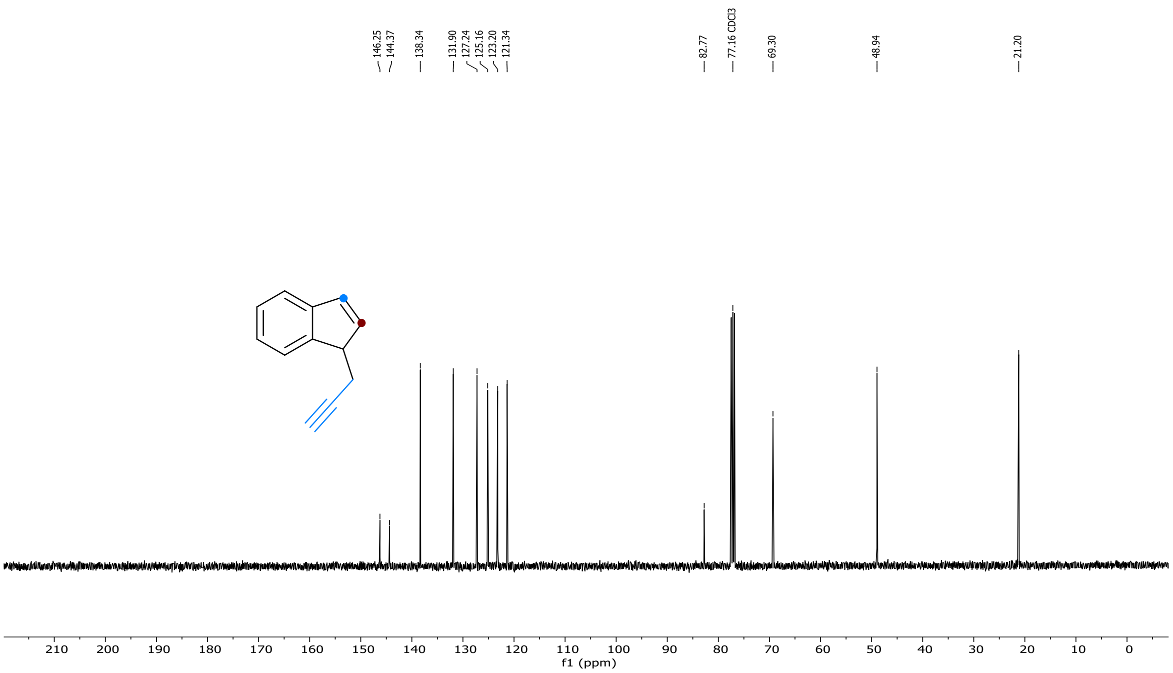

Compound **4p**:

^{1}H NMR (400 MHz, $CDCl_3$)

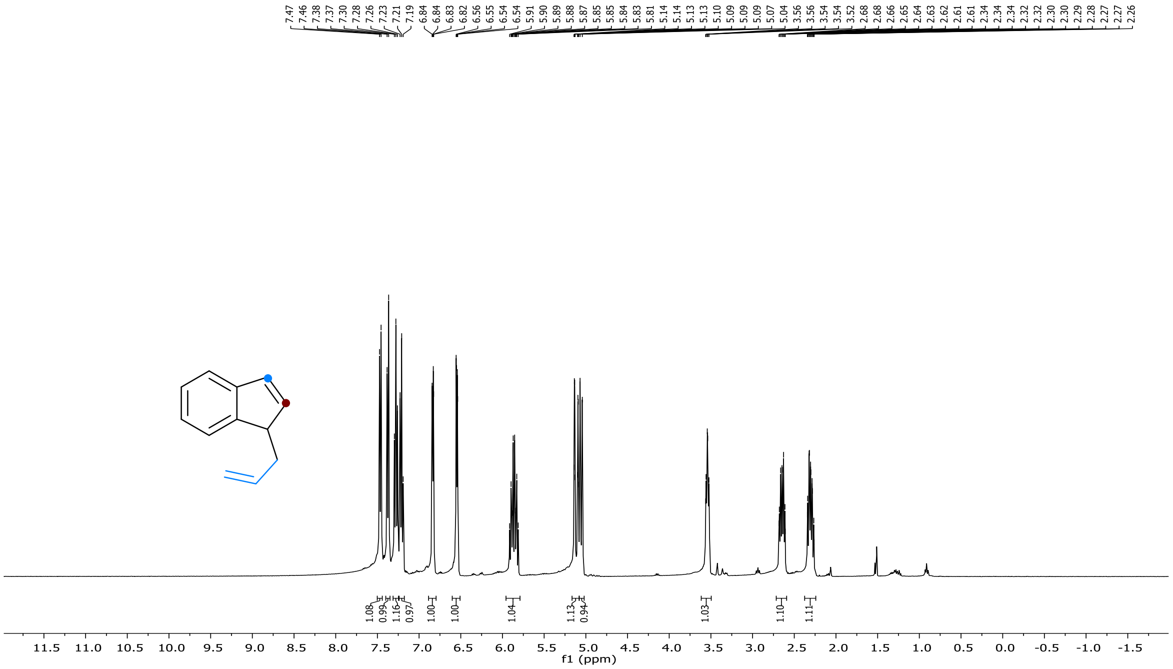

$^{13}C\{1H\}$ NMR (101 MHz, $CDCl_3$)

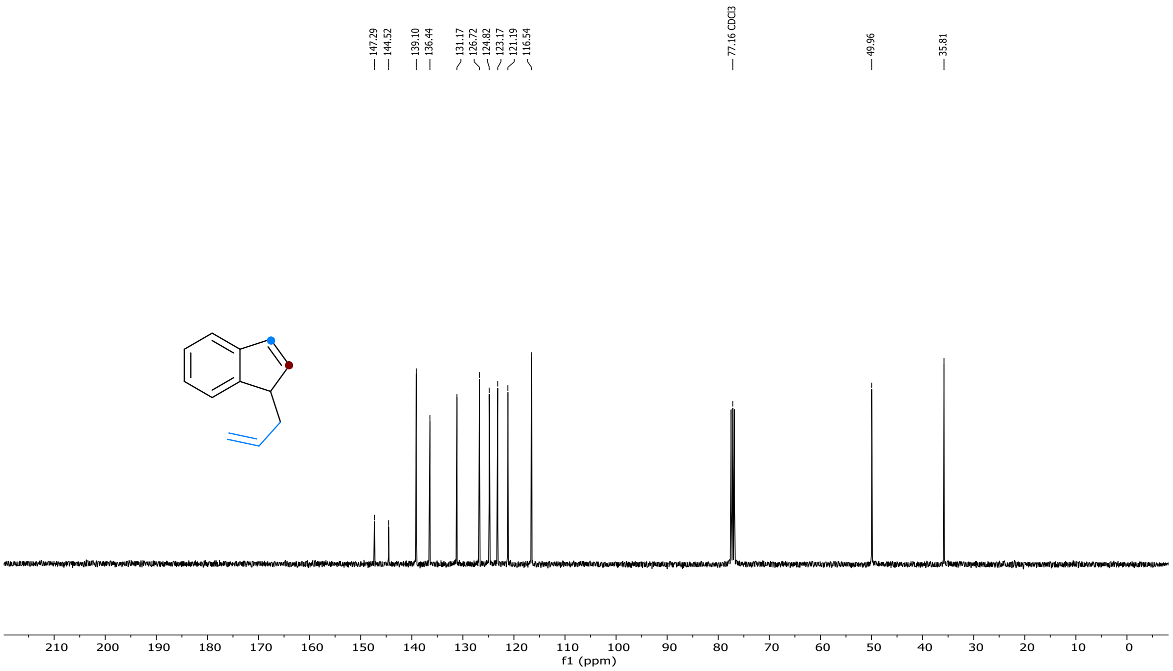

Compound **4q**:

^{1}H NMR (400 MHz, $CDCl_3$)

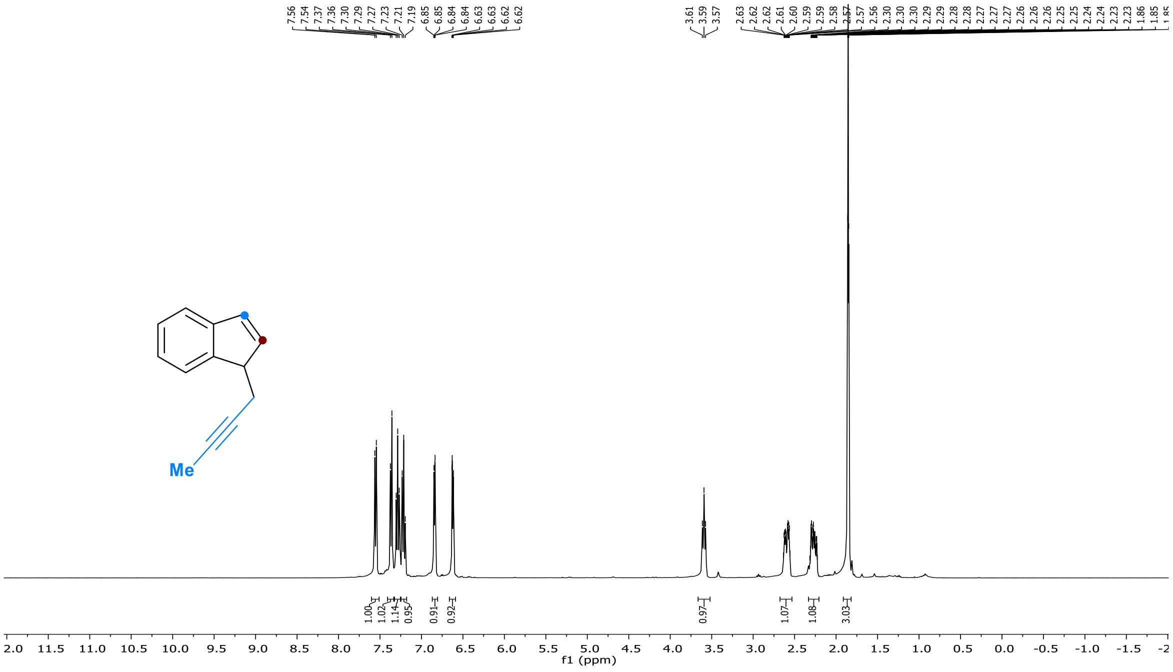

$^{13}C\{1H\}$ NMR (101 MHz, $CDCl_3$)

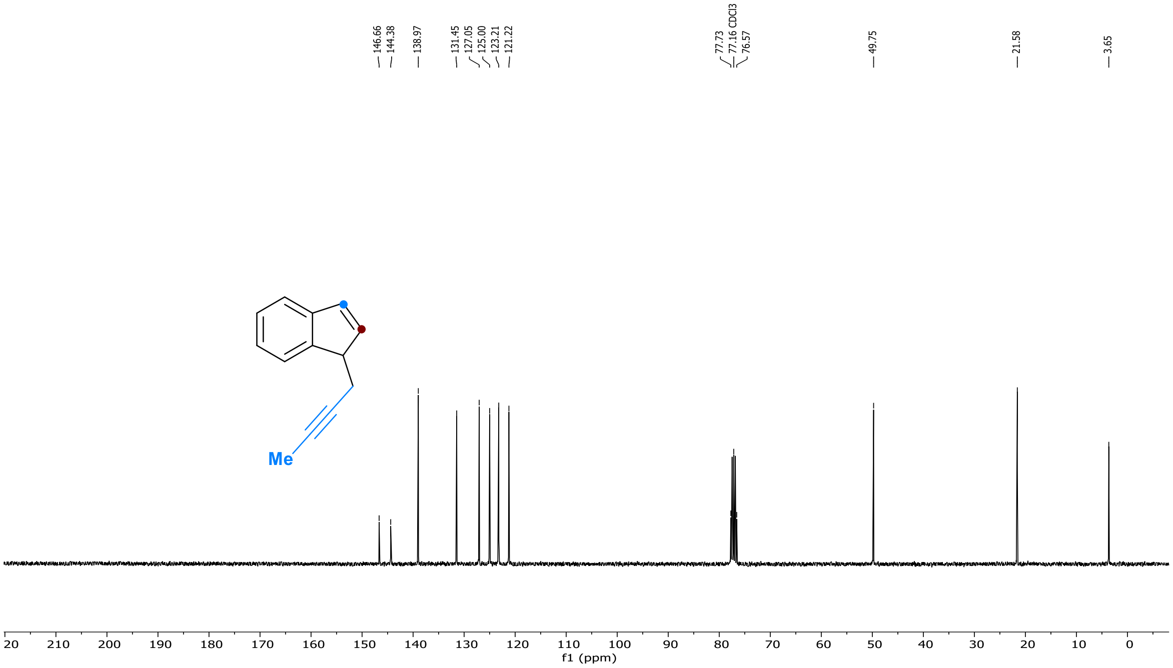

Compound **4u**:

^{1}H NMR (300 MHz, $CDCl_3$)

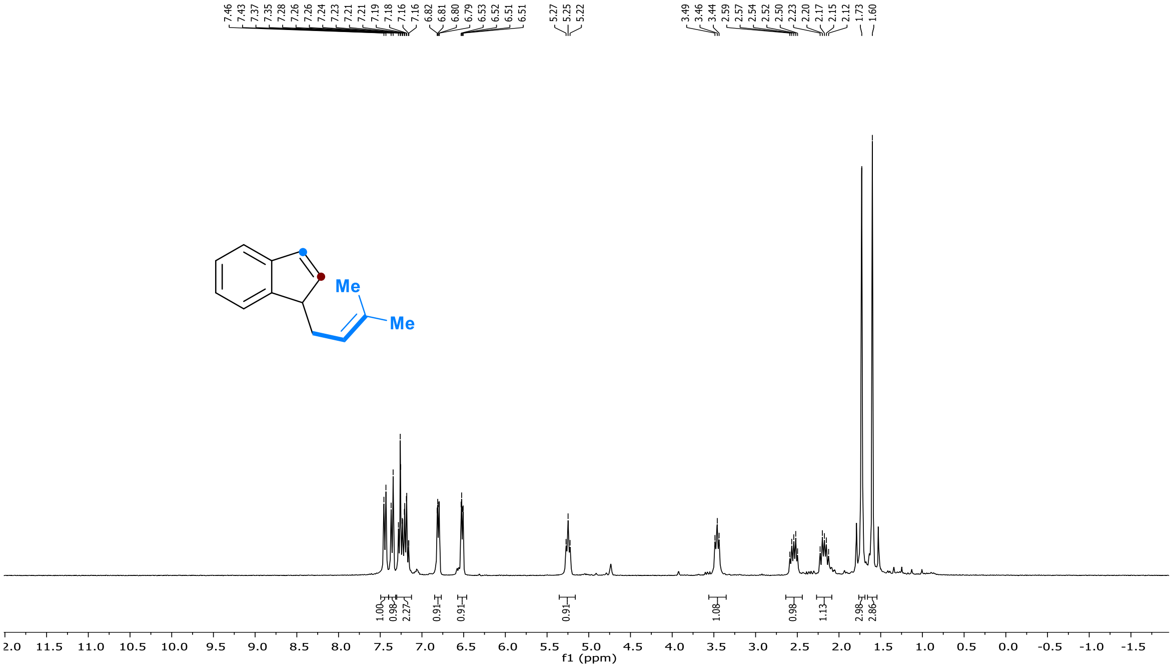

$^{13}C\{1H\}$ NMR (75 MHz, $CDCl_3$)

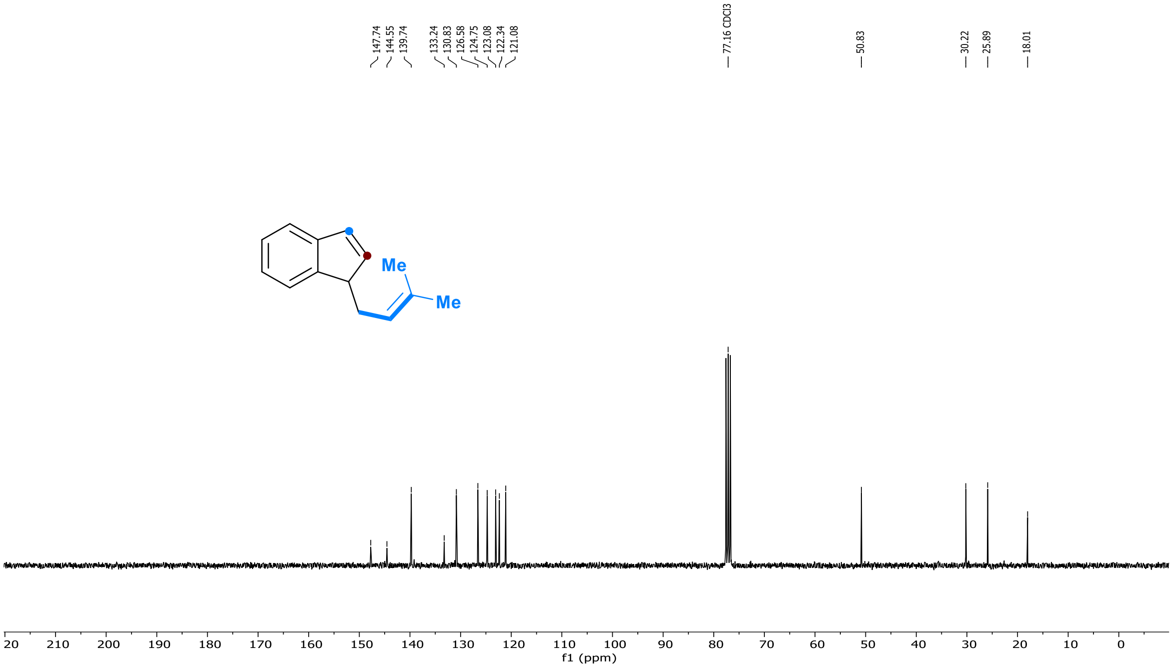

Compound **S9a**

^{1}H NMR (400 MHz, $CDCl_3$)

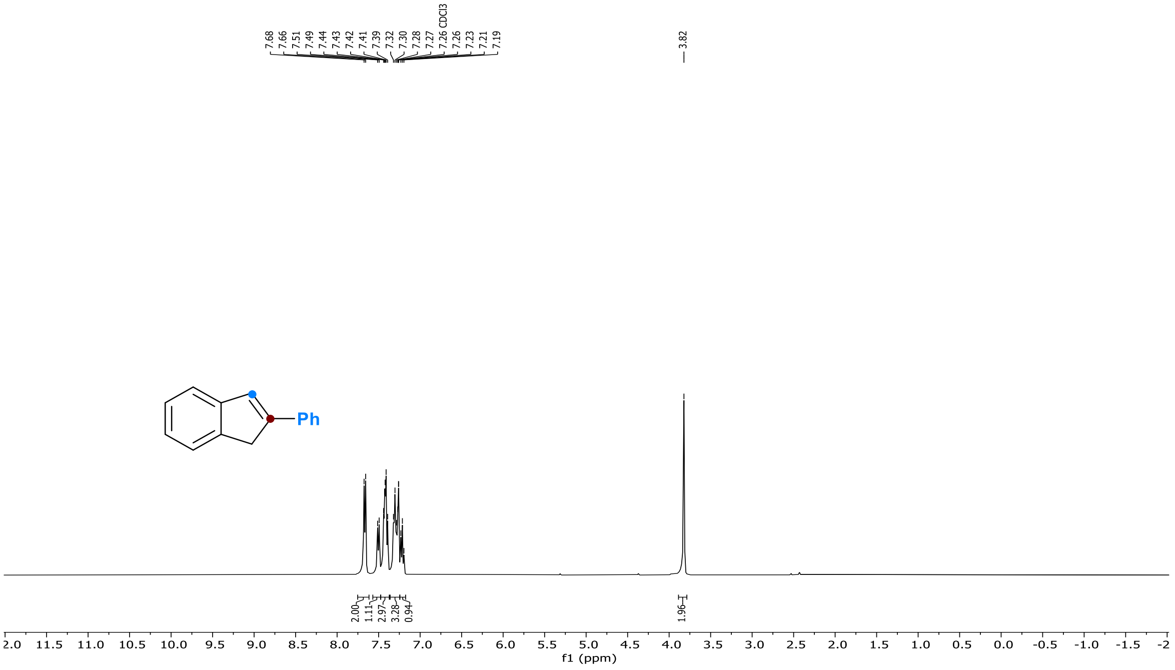

$^{13}C\{1H\}$ NMR (101 MHz, $CDCl_3$)

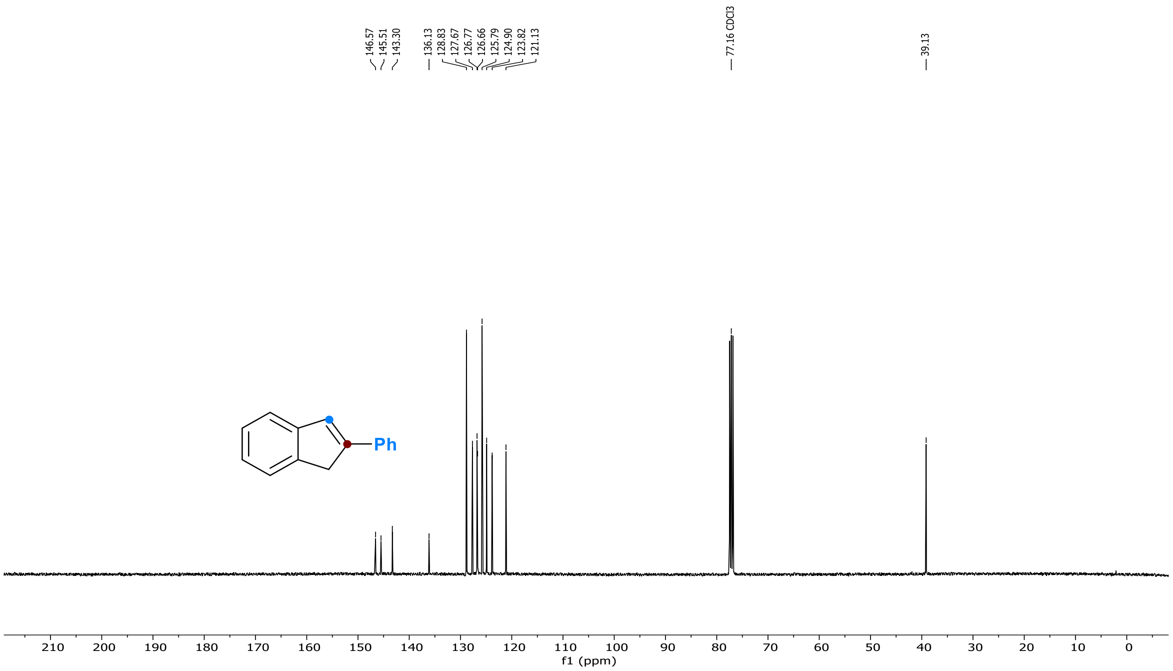

Compound **5a**:

^{1}H NMR (400 MHz, $CDCl_3$)

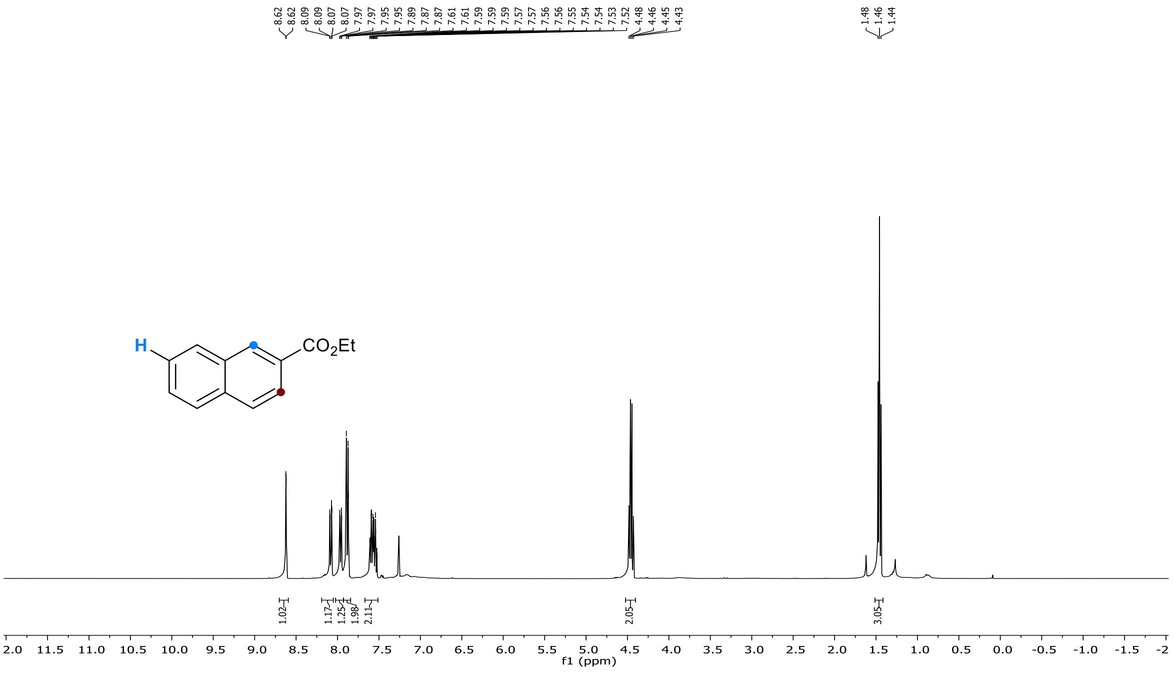

$^{13}C\{1H\}$ NMR (101 MHz, $CDCl_3$)

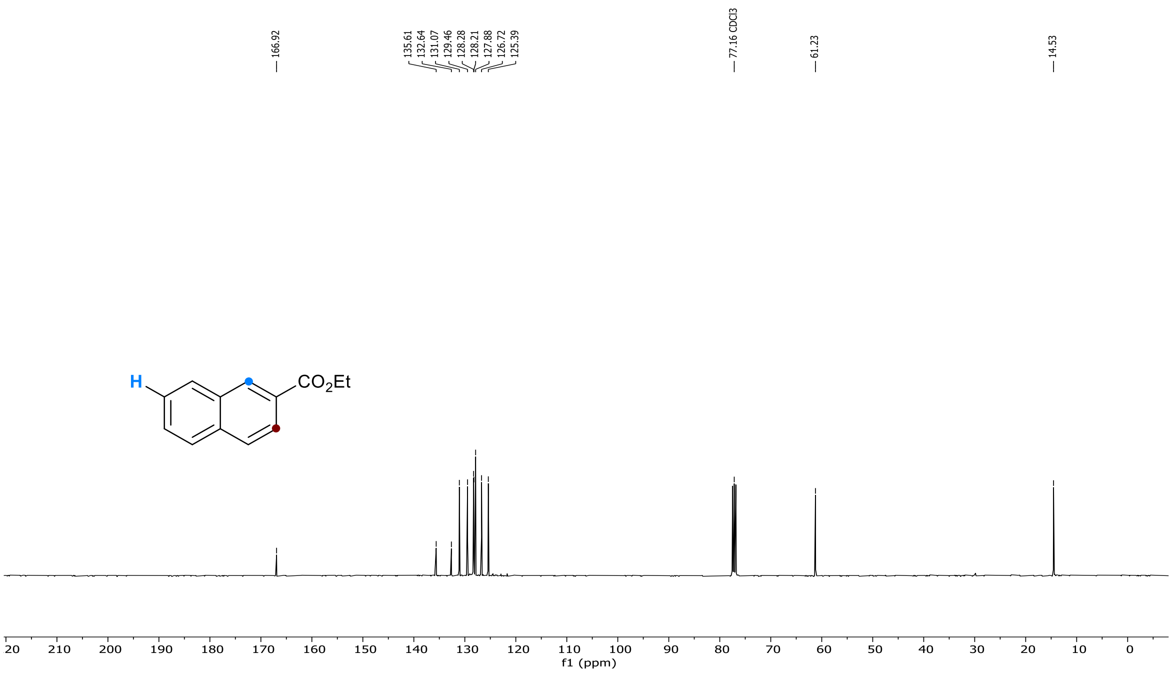

Compound **5b**:

^{1}H NMR (300 MHz, $CDCl_3$)

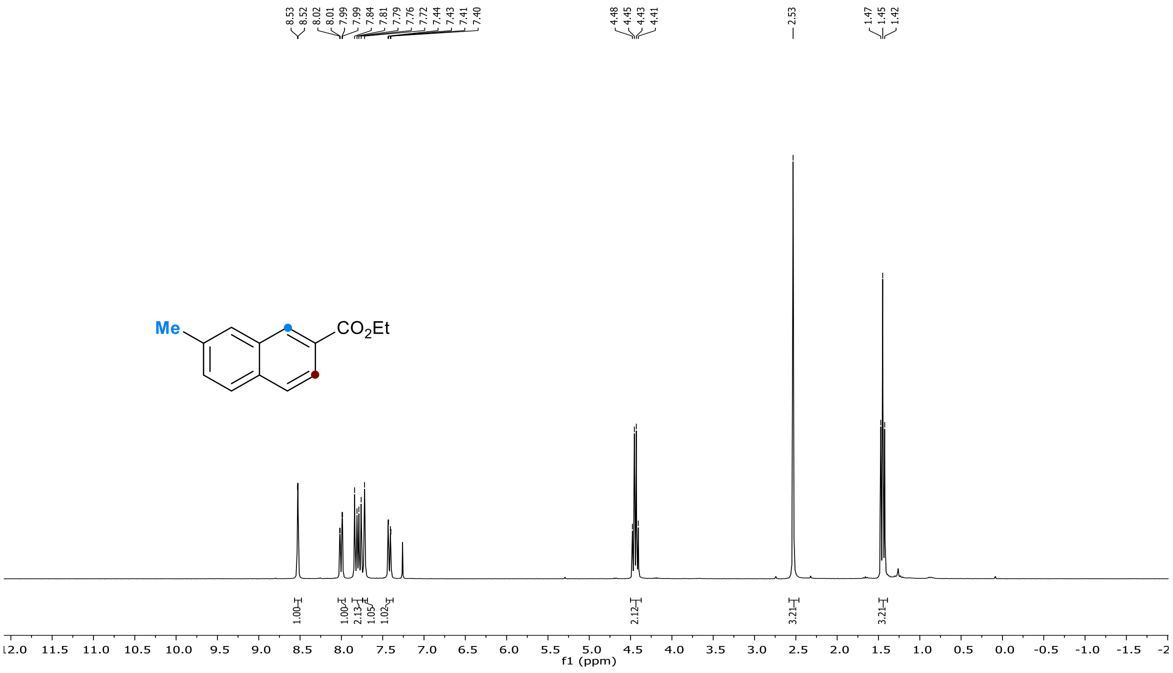

$^{13}C\{1H\}$ NMR (101 MHz, $CDCl_3$)

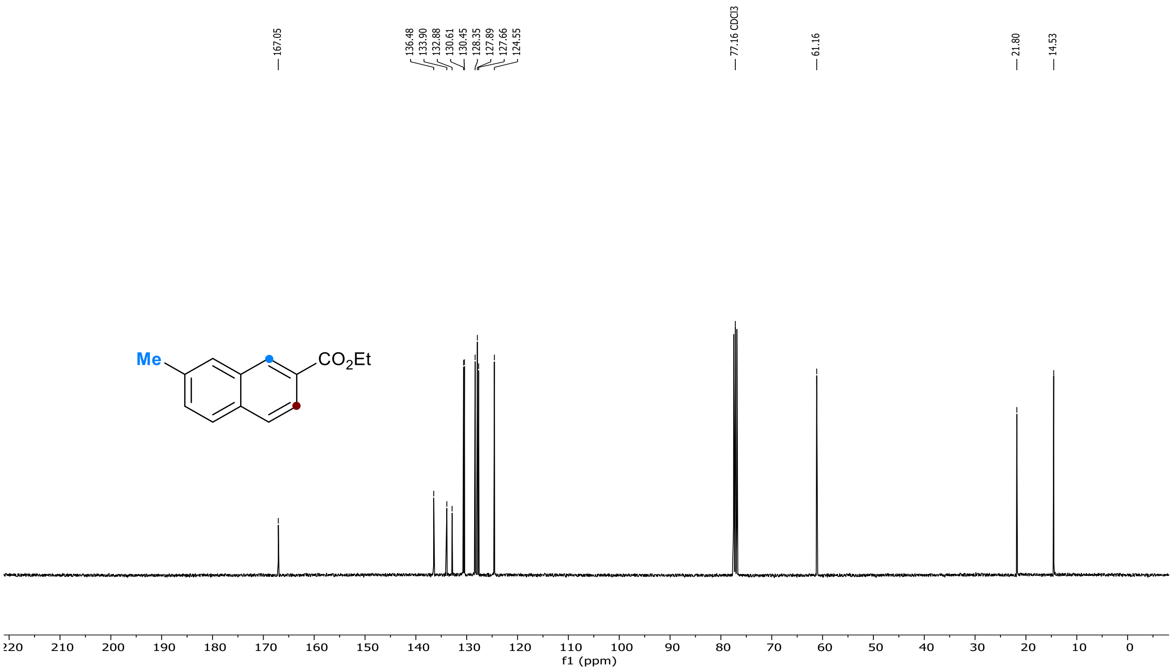

Compound **5c**:

^{1}H NMR (300 MHz, $CDCl_3$)

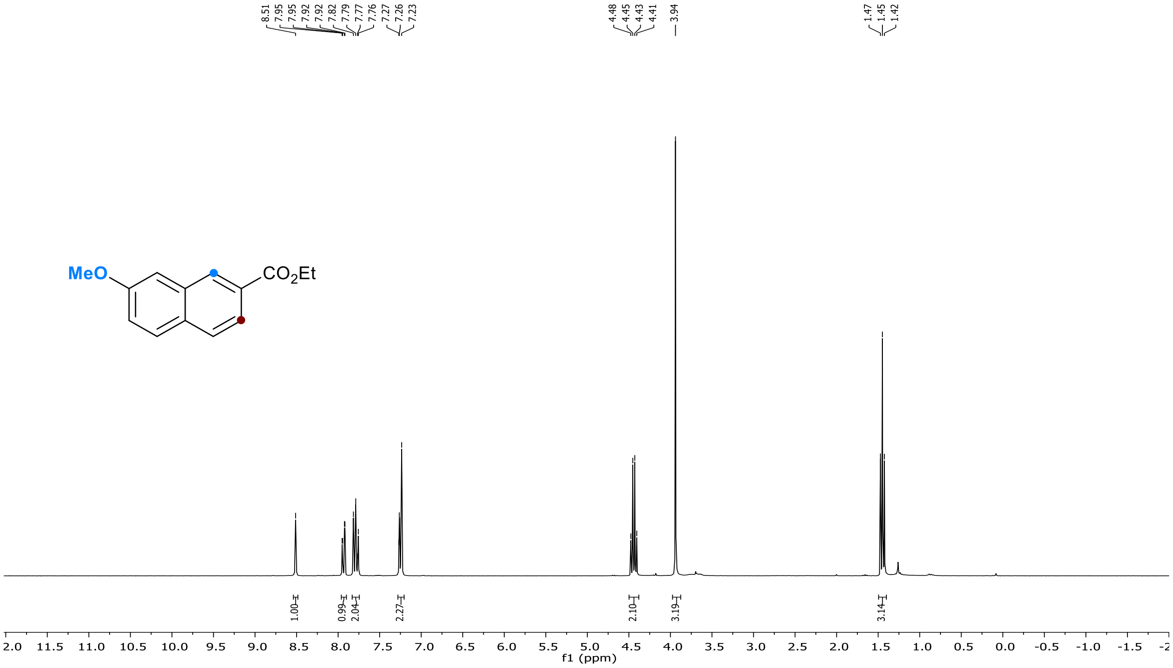

$^{13}C\{1H\}$ NMR (101 MHz, $CDCl_3$)

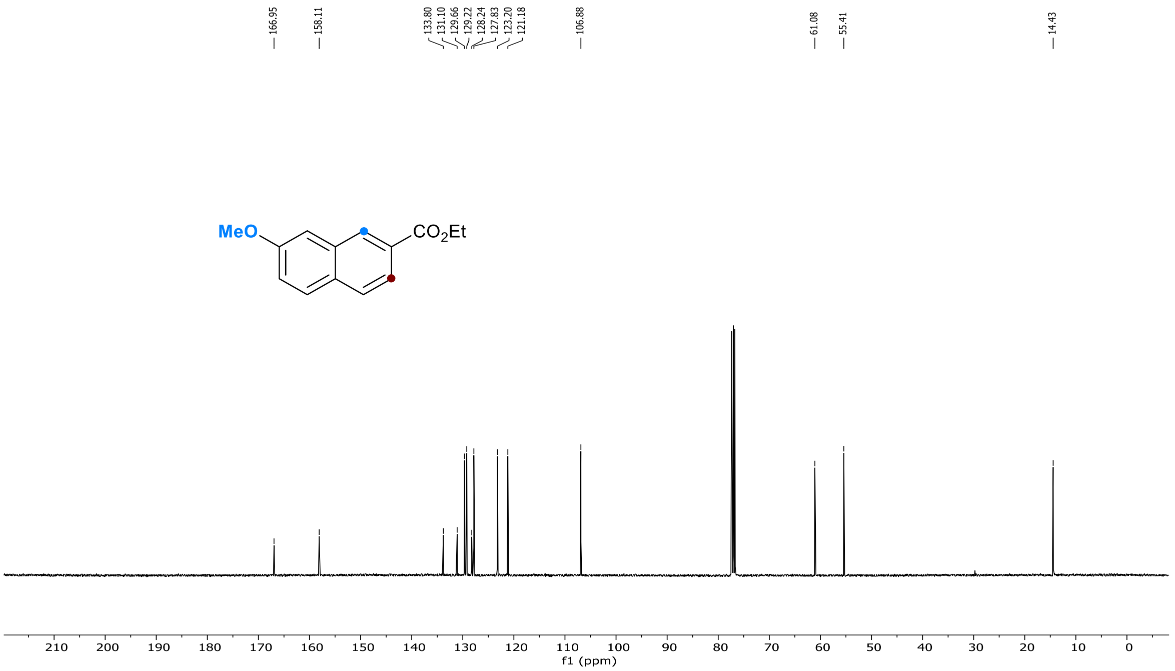

Compound **5d**:

^{1}H NMR (300 MHz, $CDCl_3$)

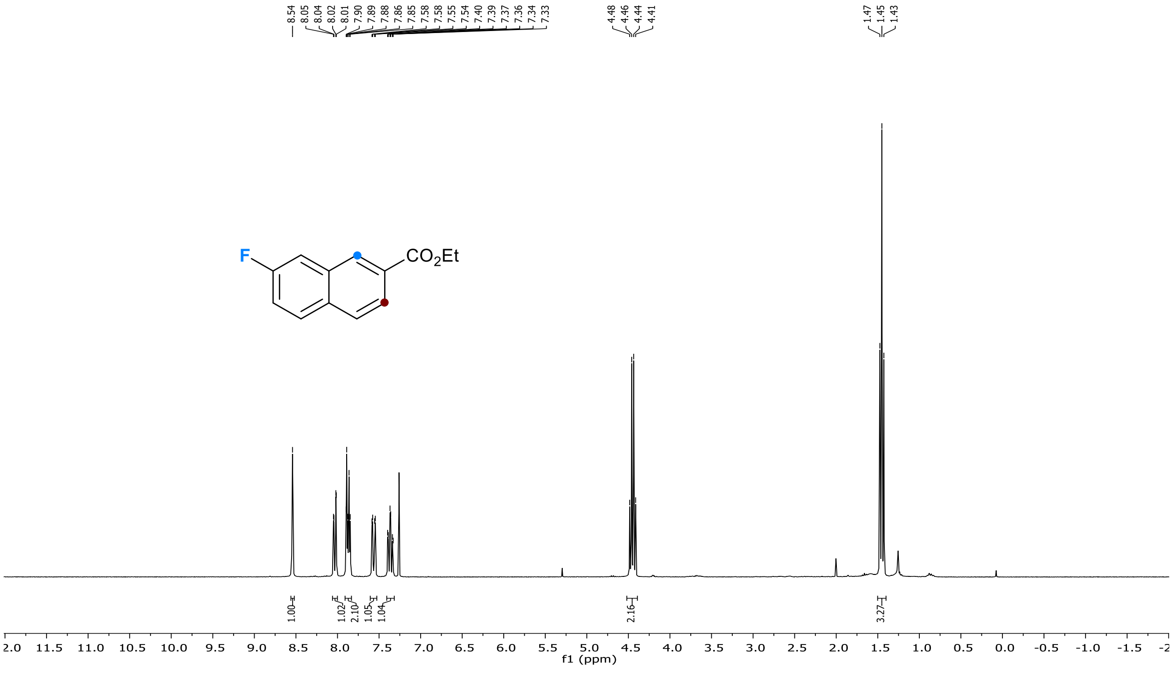

$^{13}C\{1H\}$ NMR (101 MHz, $CDCl_3$)

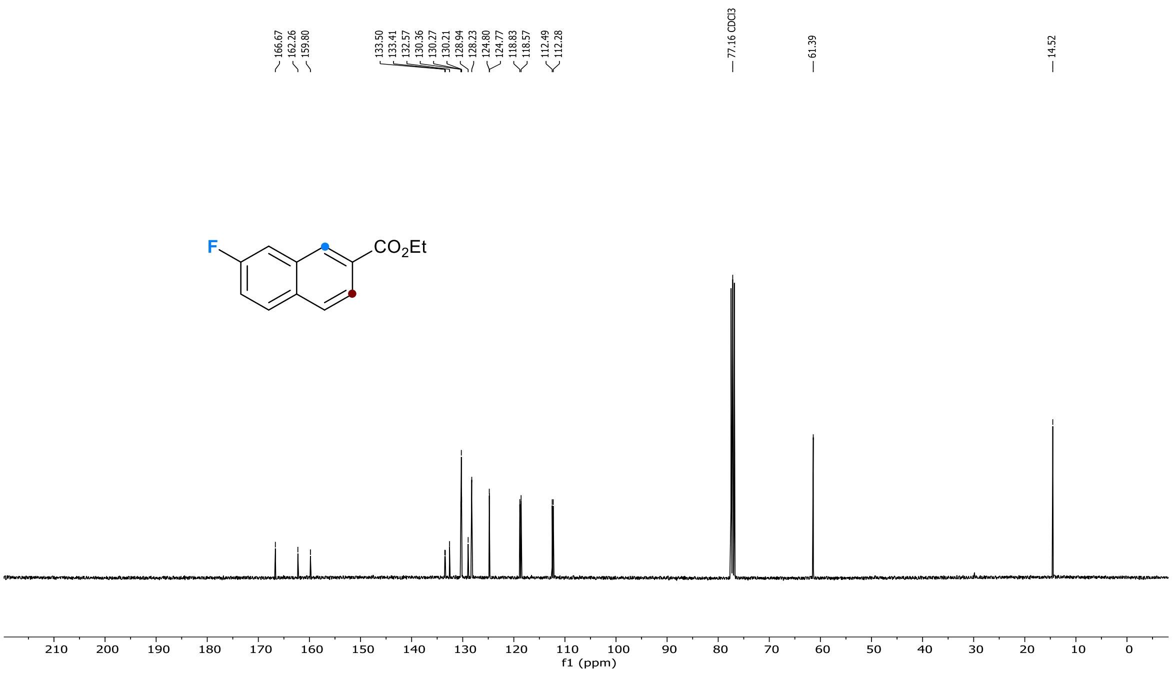

^{19}F NMR (377 MHz, $CDCl_3$)

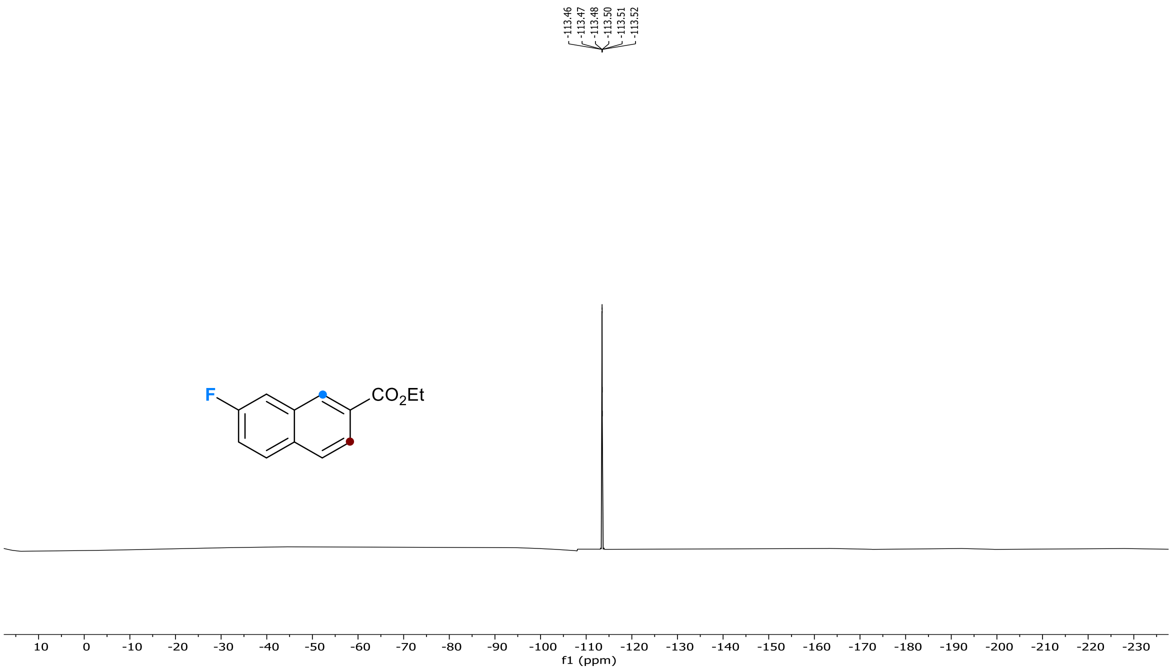

Compound **5e**:

^{1}H NMR (300 MHz, $CDCl_3$)

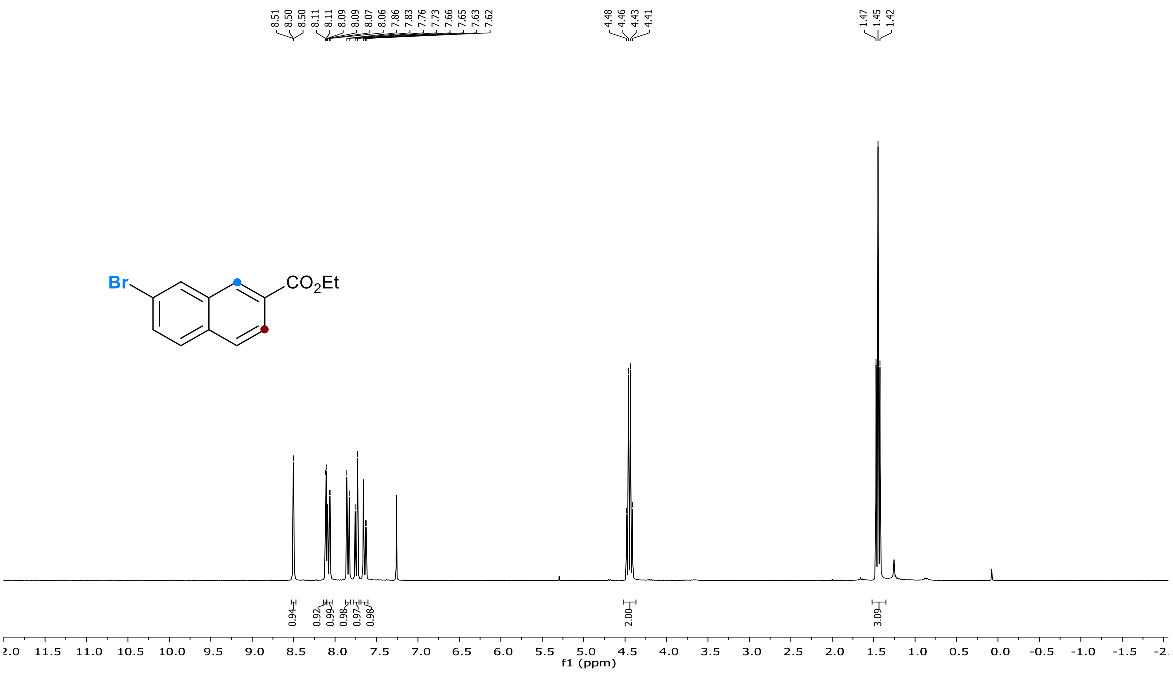

^{13}C{1H} NMR (101 MHz, $CDCl_3$)

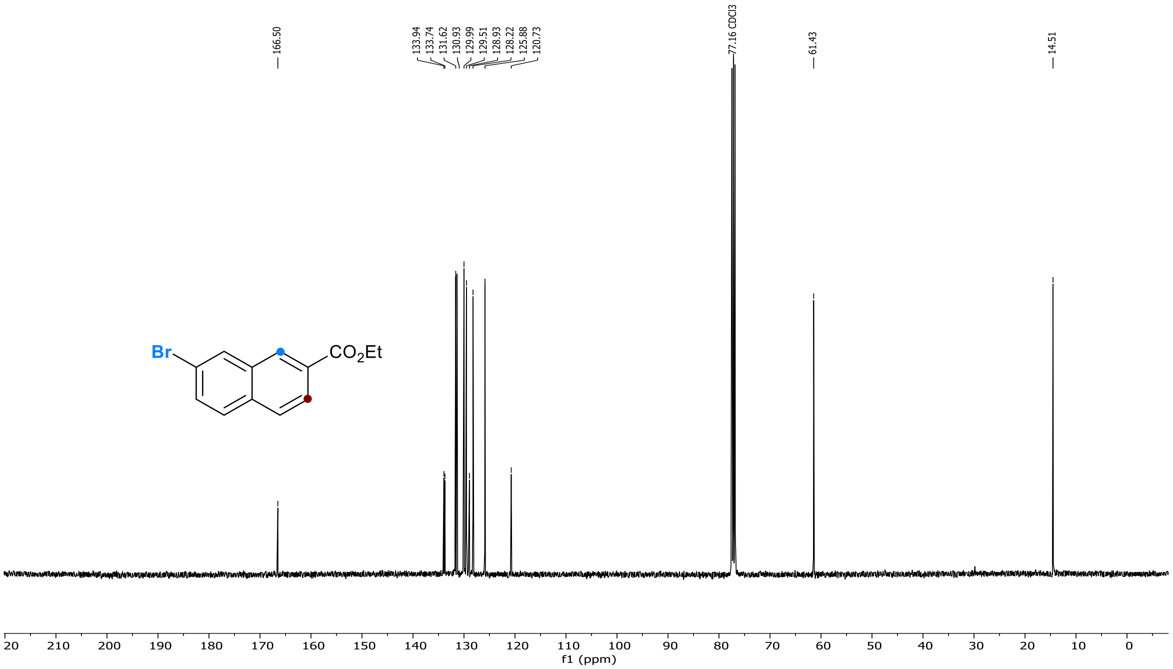

Compound **5f**:

^{1}H NMR (300 MHz, $CDCl_3$)

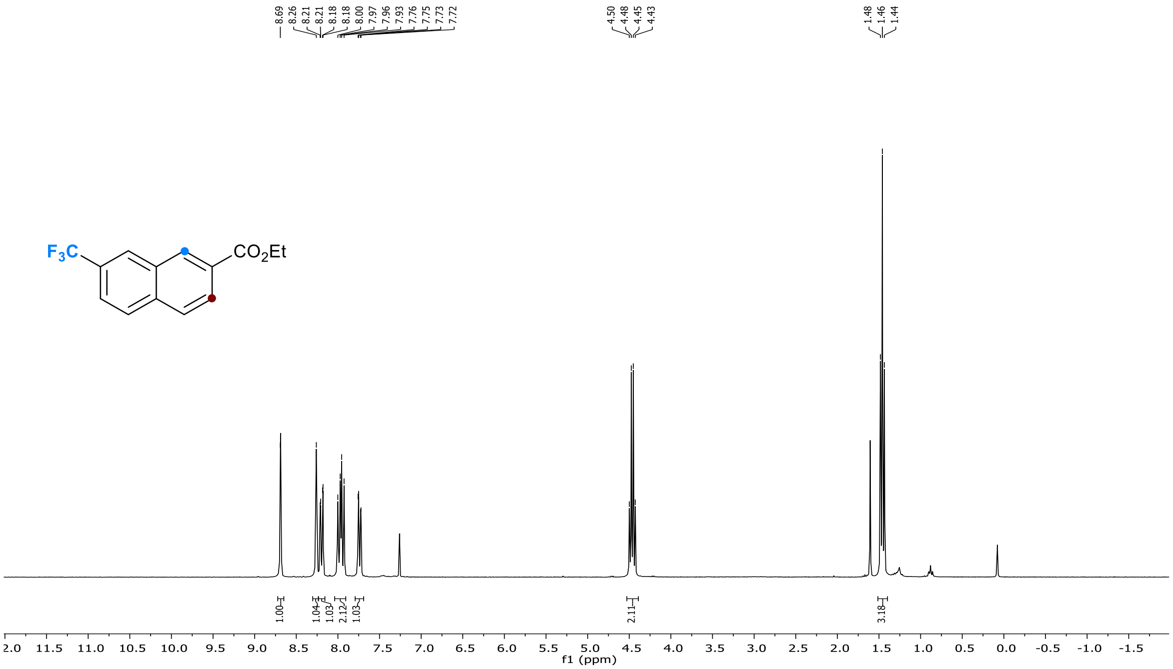

^{13}C{1H} NMR (75 MHz, $CDCl_3$)

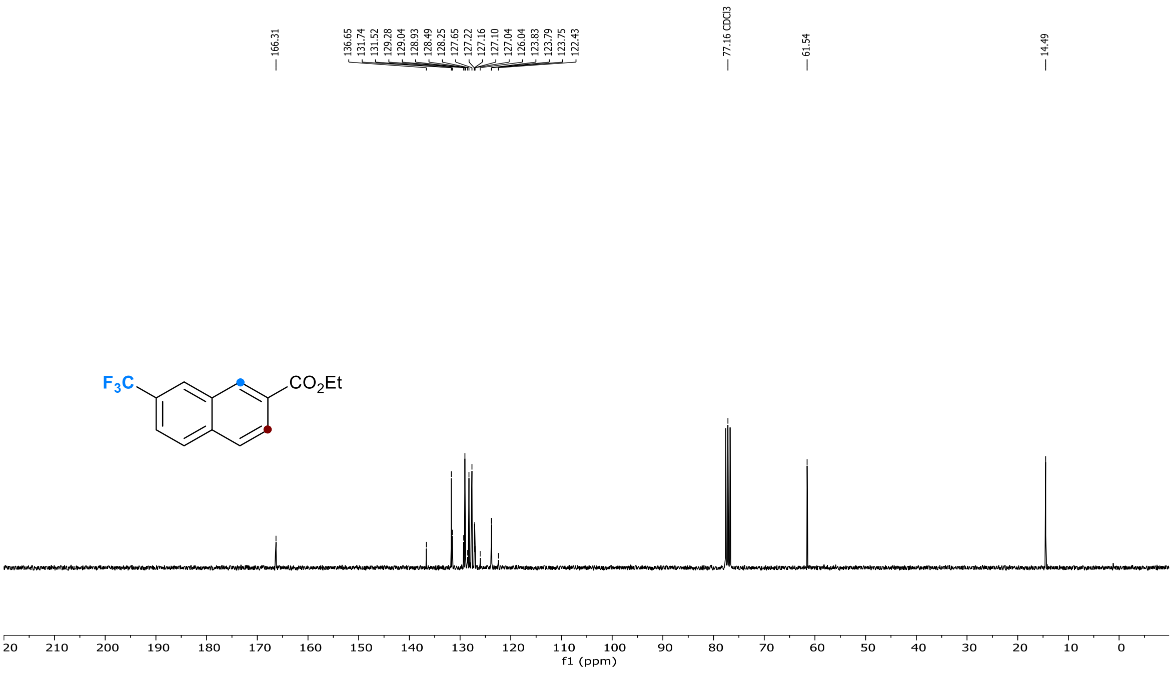

^{19}F NMR (282 MHz, $CDCl_3$)

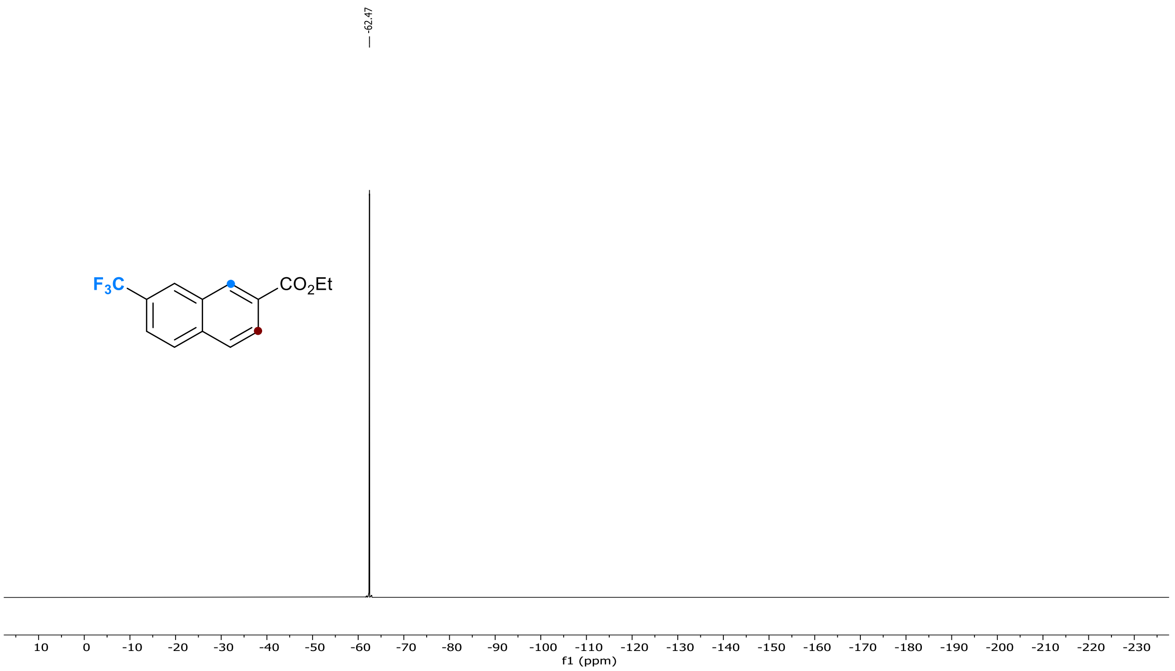

Compound **5g**:

^{1}H NMR (300 MHz, $CDCl_3$)

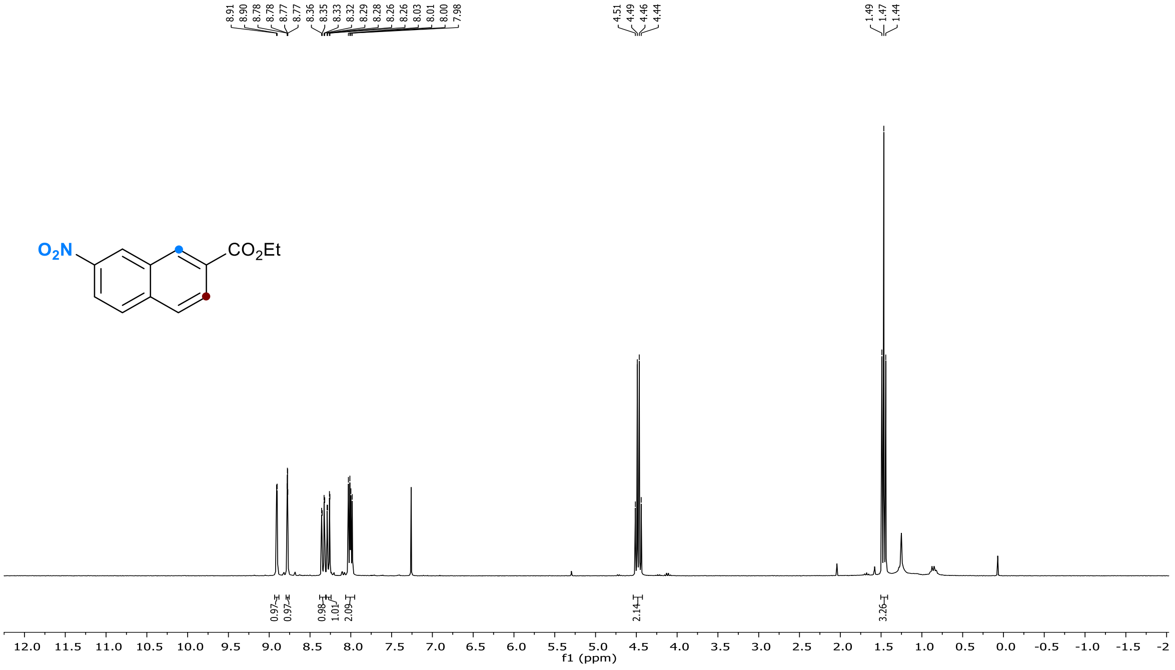

$^{13}C\{1H\}$ NMR (101 MHz, $CDCl_3$)

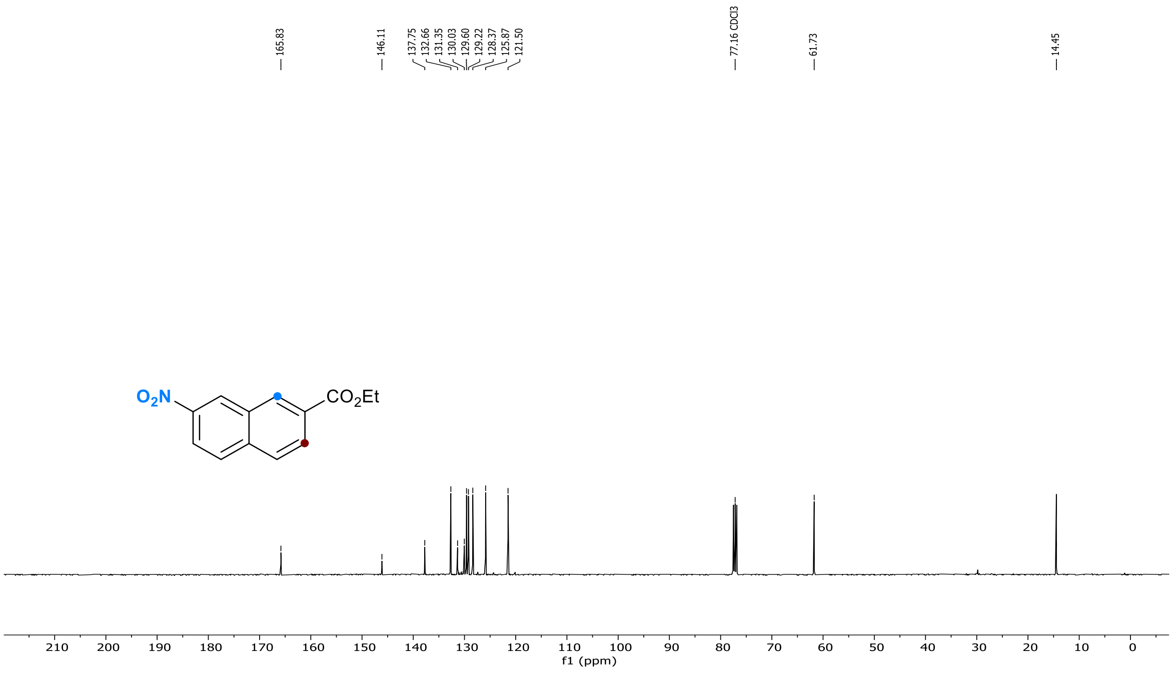

Compound **5h**:

^{1}H NMR (300 MHz, $CDCl_3$)

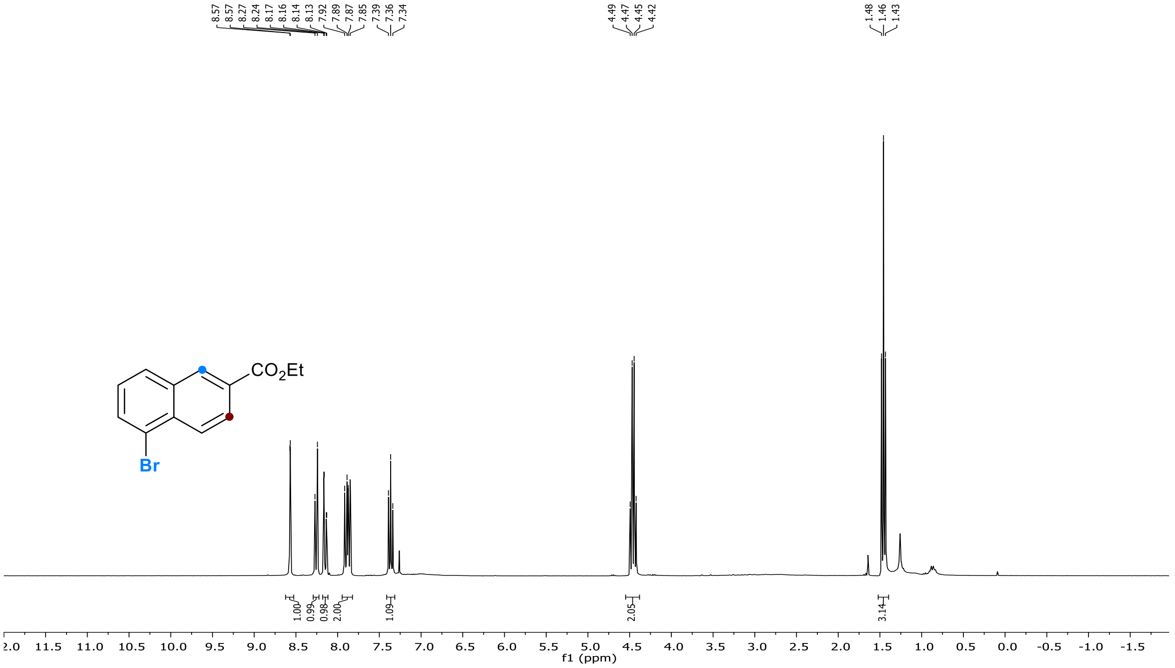

$^{13}C\{1H\}$ NMR (101 MHz, $CDCl_3$)

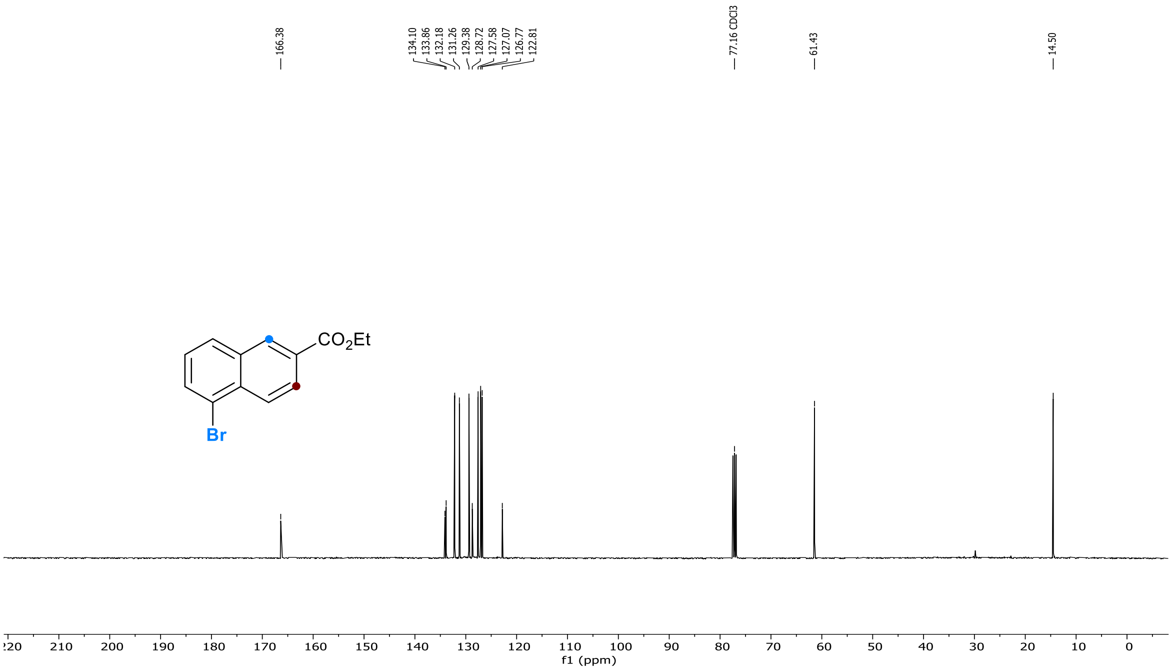

Compound **5i**:

^{1}H NMR (400 MHz, $CDCl_3$)

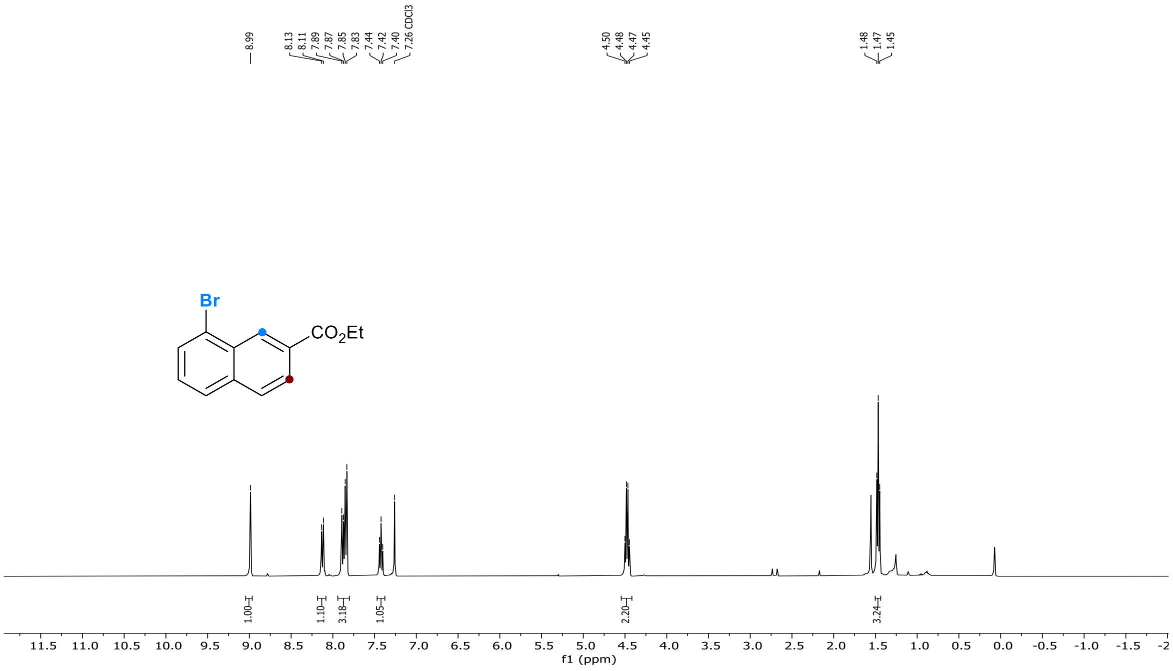

$^{13}C\{1H\}$ NMR (101 MHz, $CDCl_3$)

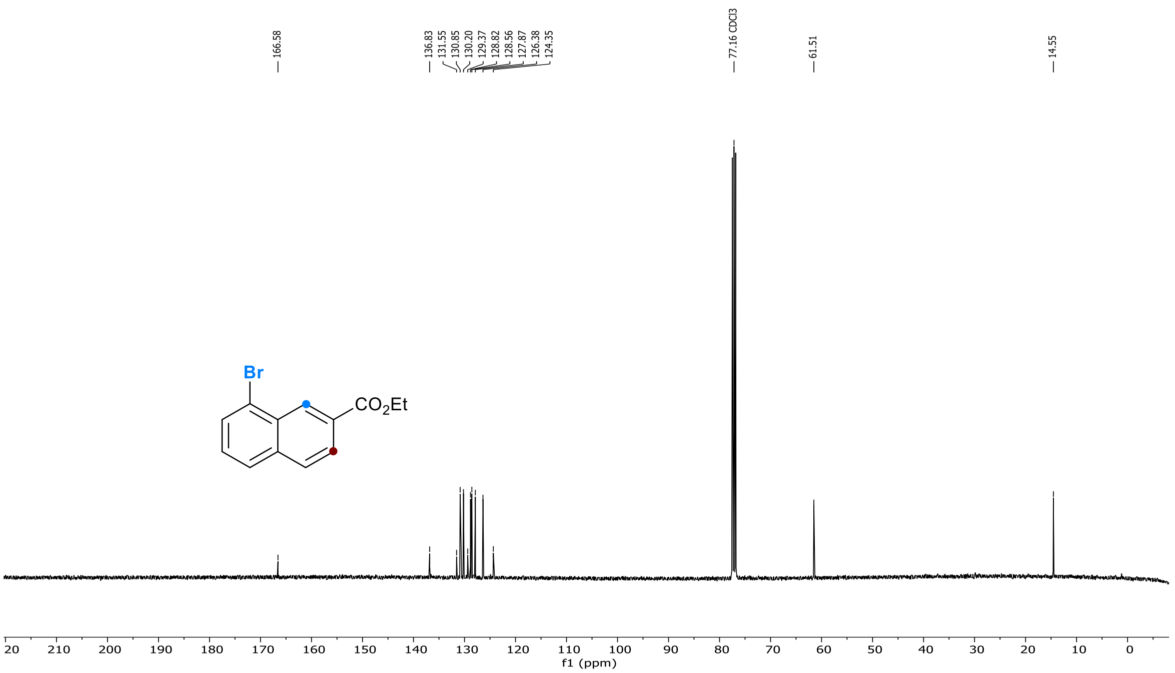

Compound **5j**:

^{1}H NMR (300 MHz, $CDCl_3$)

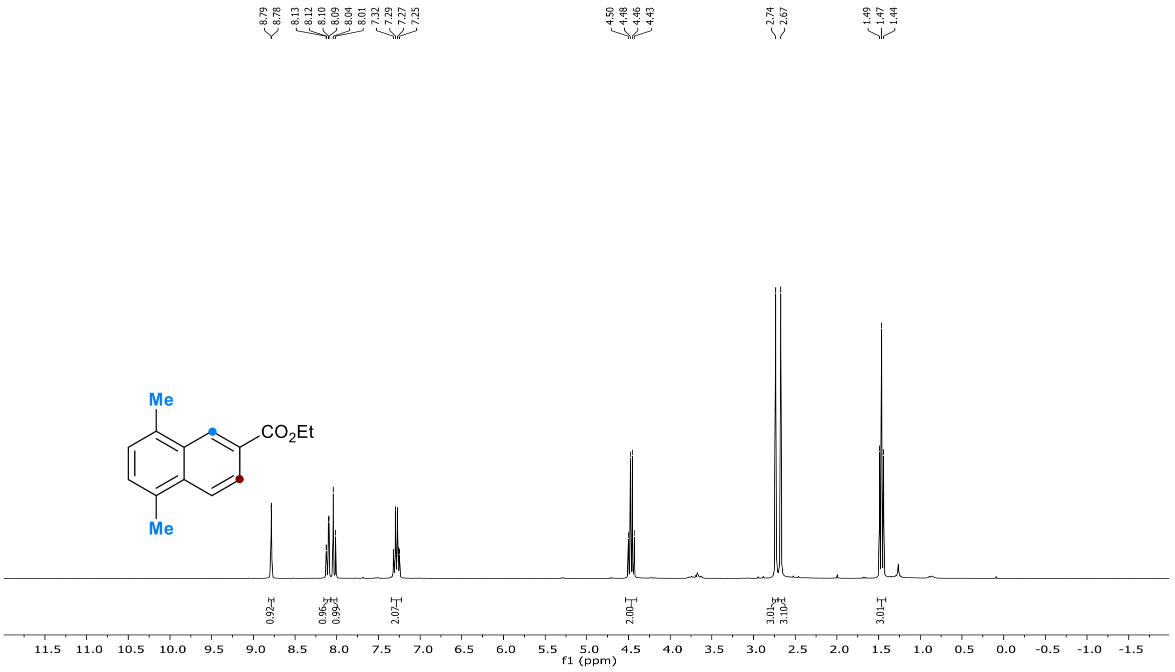

$^{13}C\{1H\}$ NMR (101 MHz, $CDCl_3$)

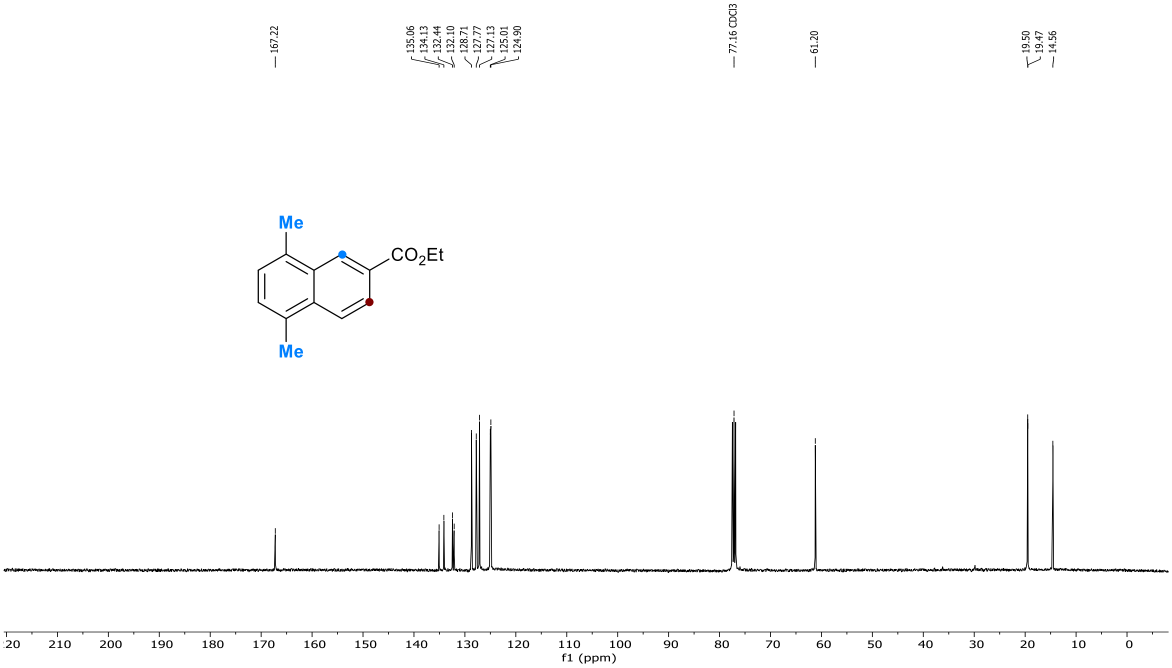

Compound **5k**:

^1H NMR (300 MHz, $CDCl_3$)

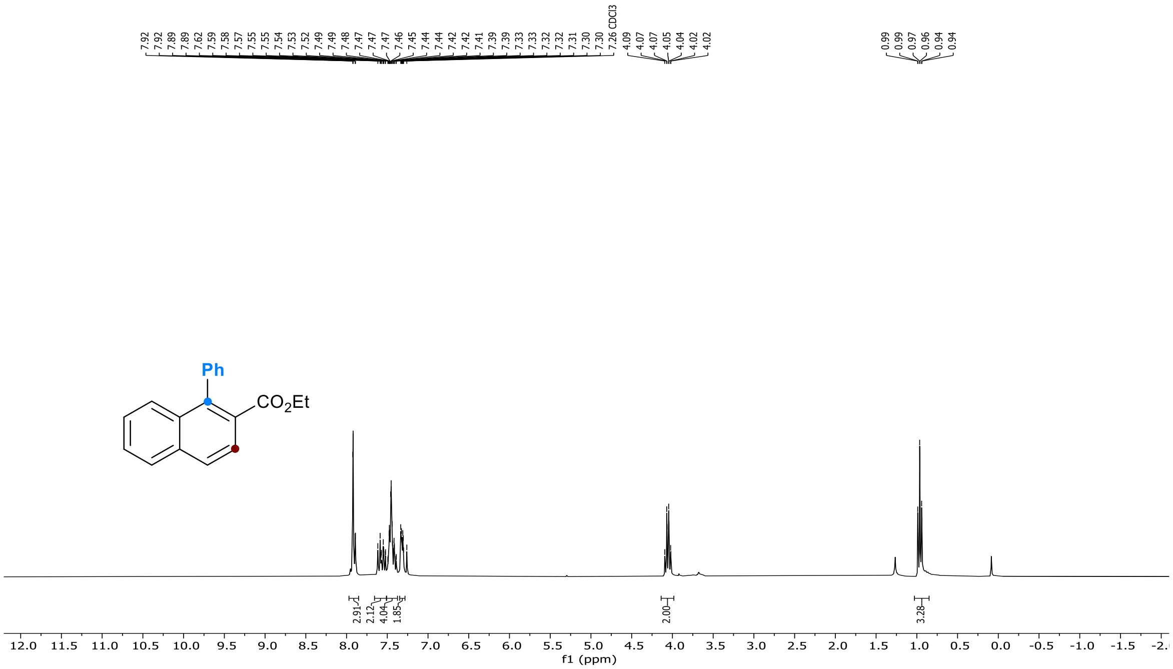

$^{13}C\{1H\}$ NMR (101 MHz, $CDCl_3$)

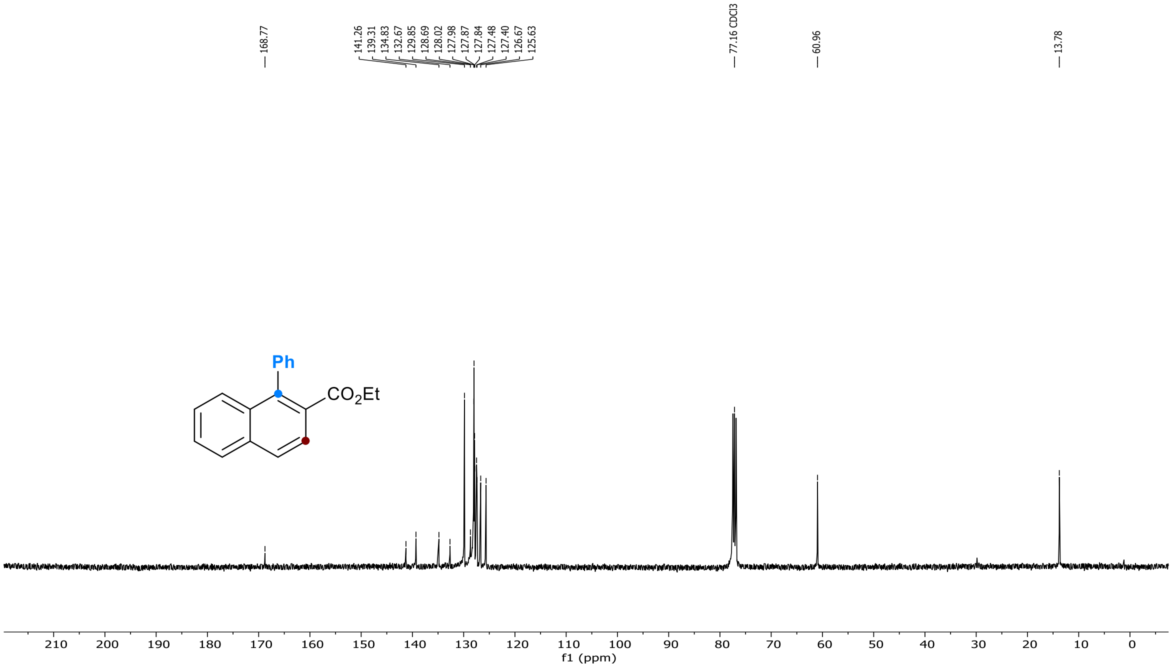

Compound **5l**:

^1H NMR (400 MHz, $CDCl_3$)

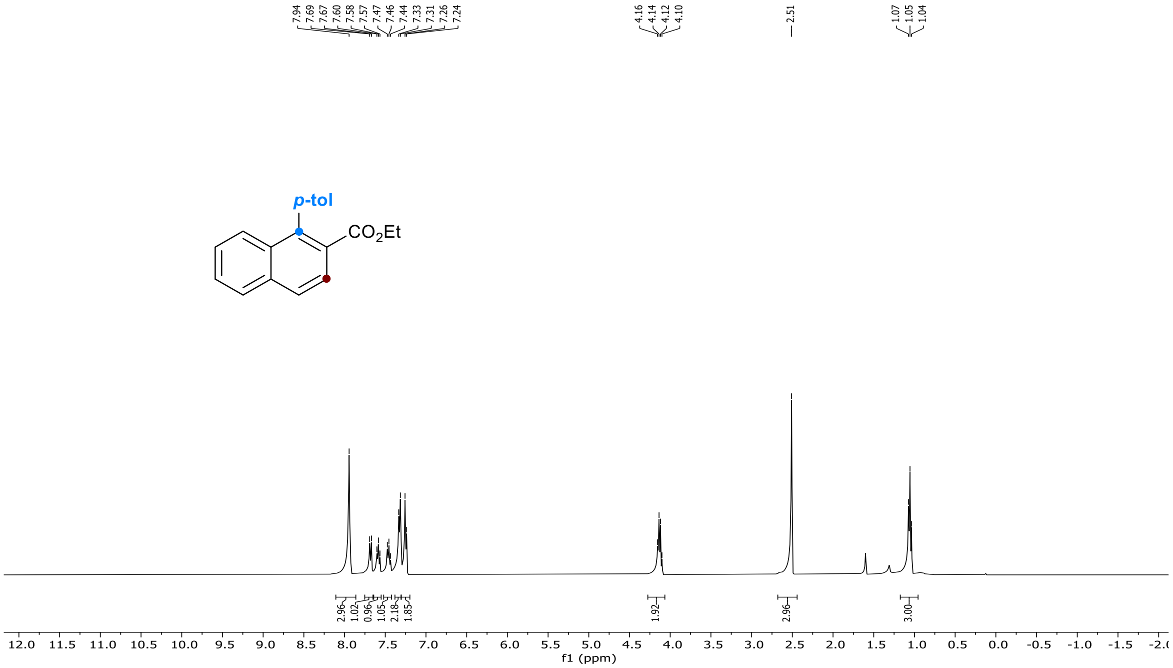

$^{13}C\{1H\}$ NMR (101 MHz, $CDCl_3$)

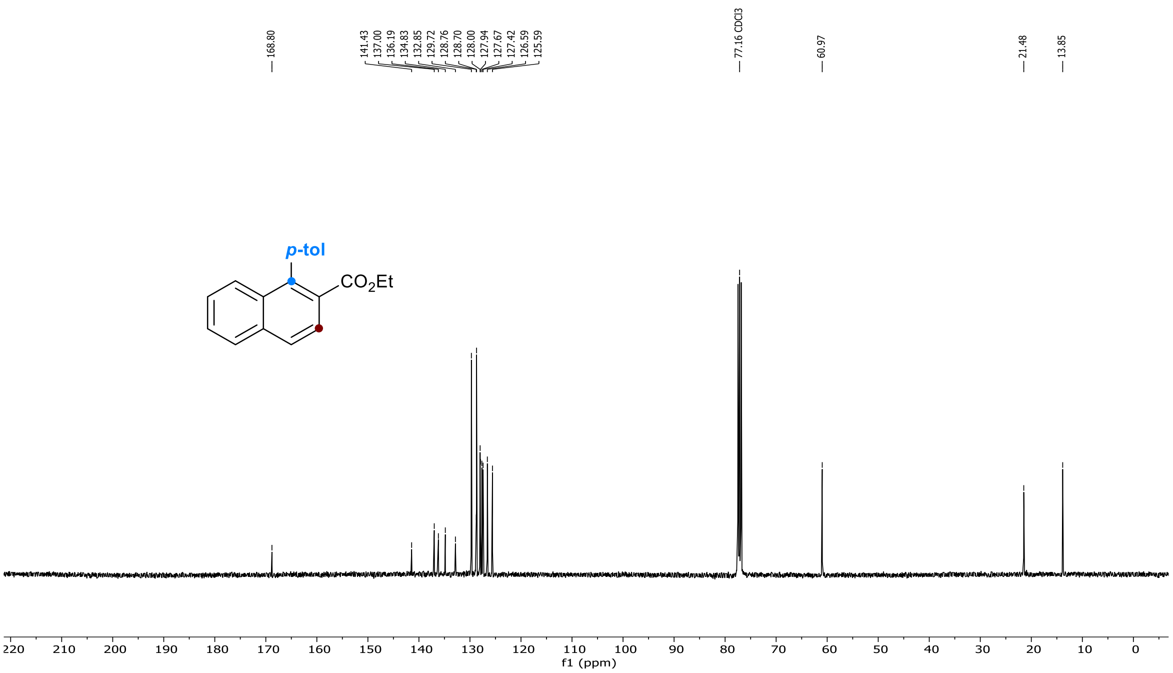

Compound **5m**:

^{1}H NMR (400 MHz, $CDCl_3$)

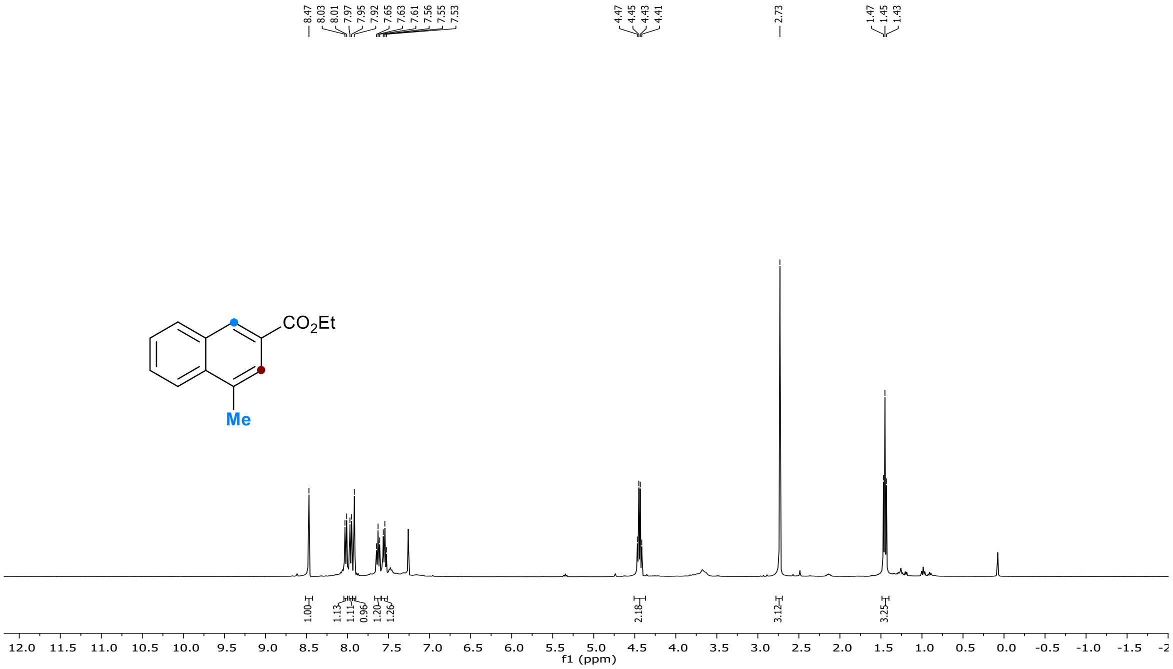

$^{13}C\{1H\}$ NMR (101 MHz, $CDCl_3$)

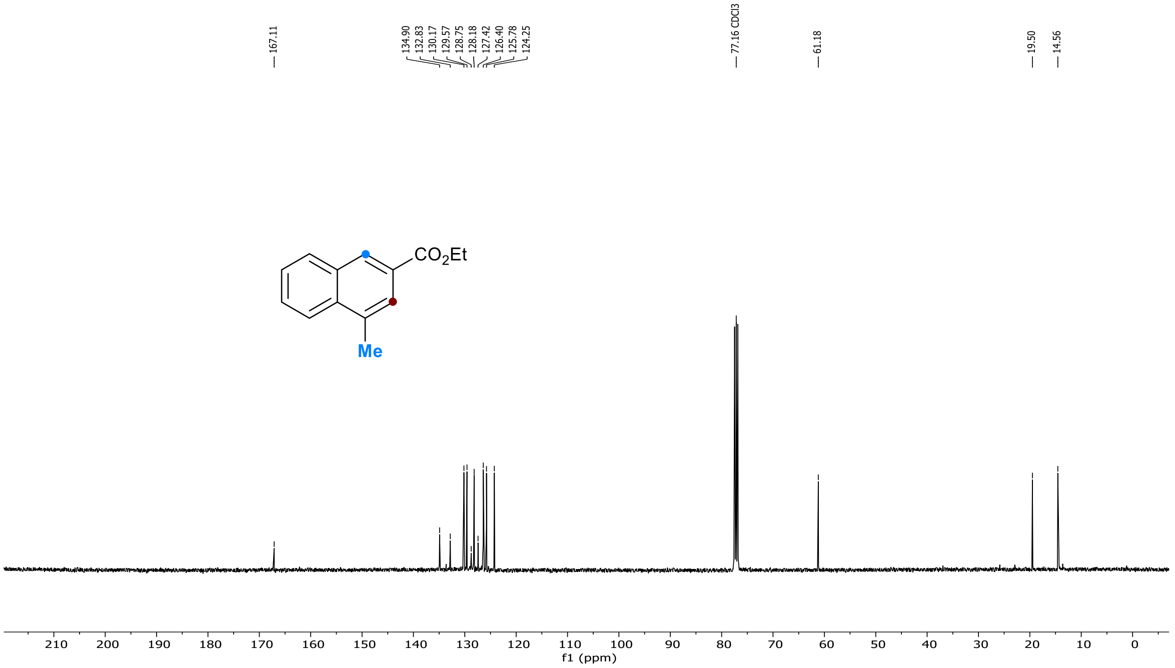

Compound **5n**:

^{1}H NMR (300 MHz, $CDCl_3$)

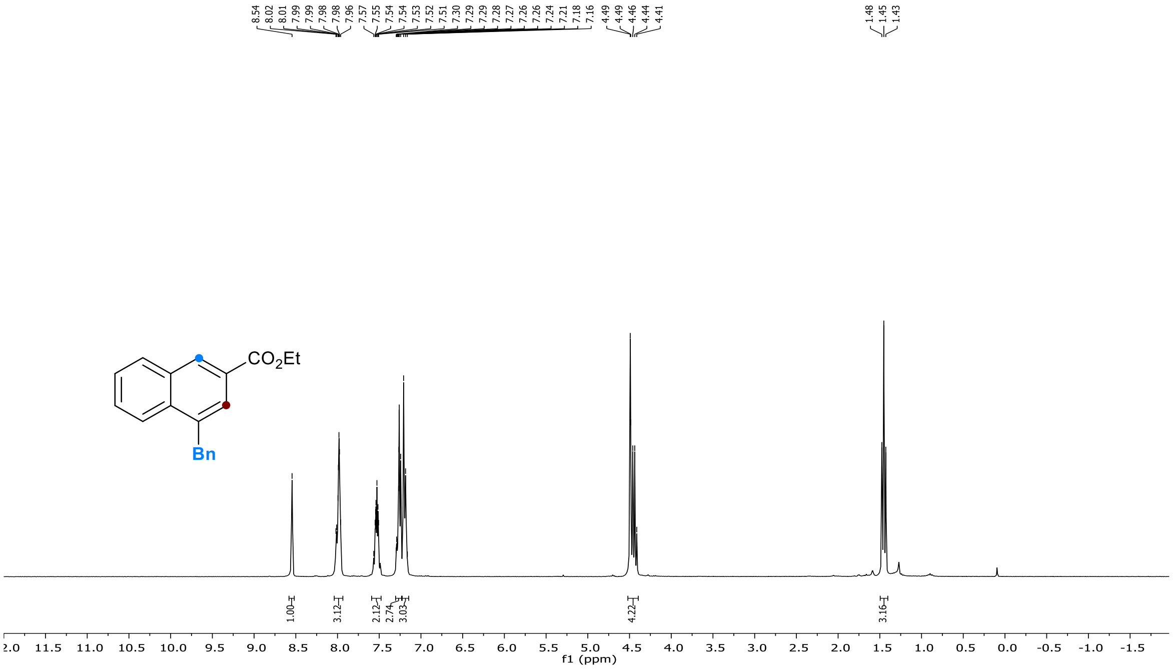

$^{13}C\{1H\}$ NMR (101 MHz, $CDCl_3$)

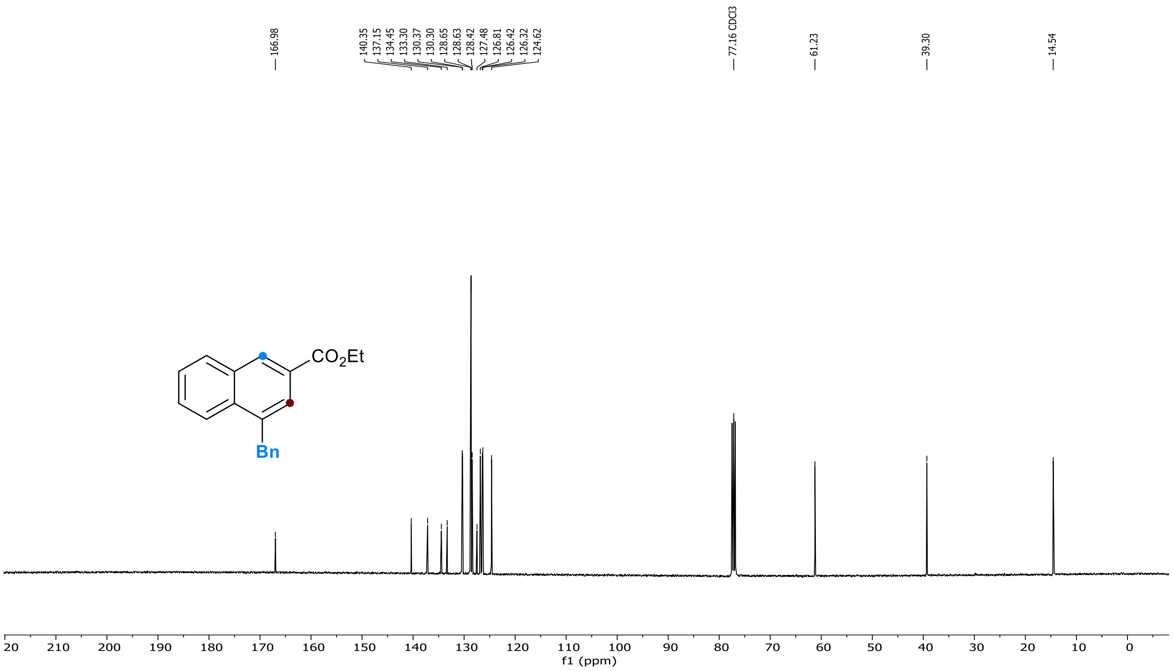

Compound **5o**:

^{1}H NMR (300 MHz, $CDCl_3$)

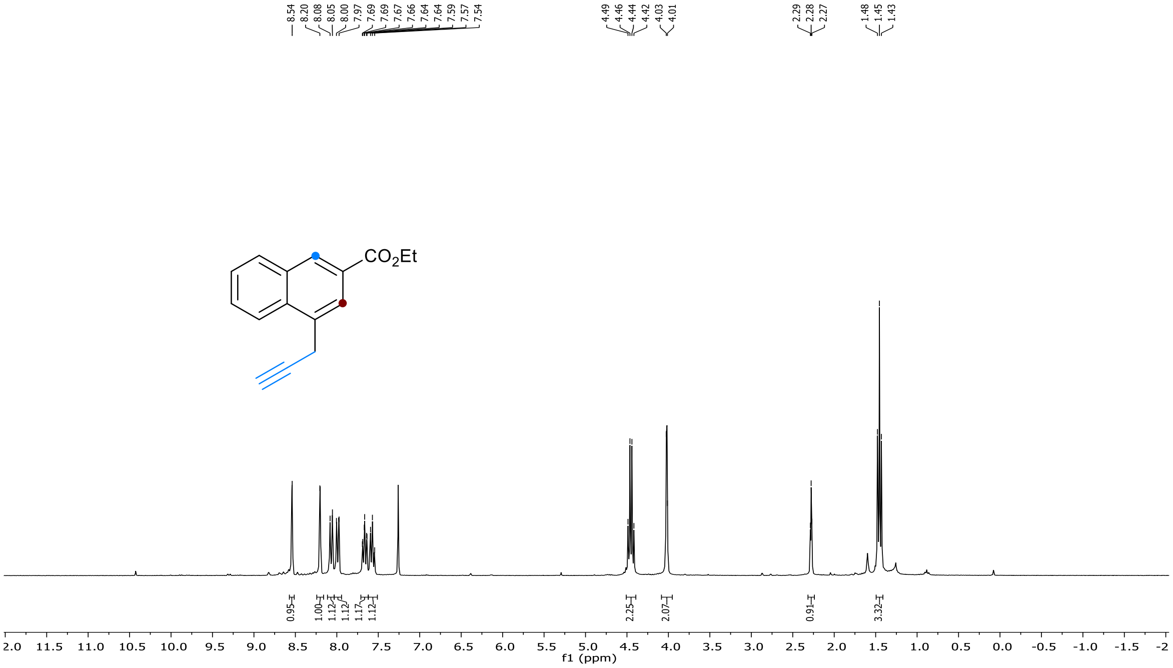

$^{13}C\{1H\}$ NMR (101 MHz, $CDCl_3$)

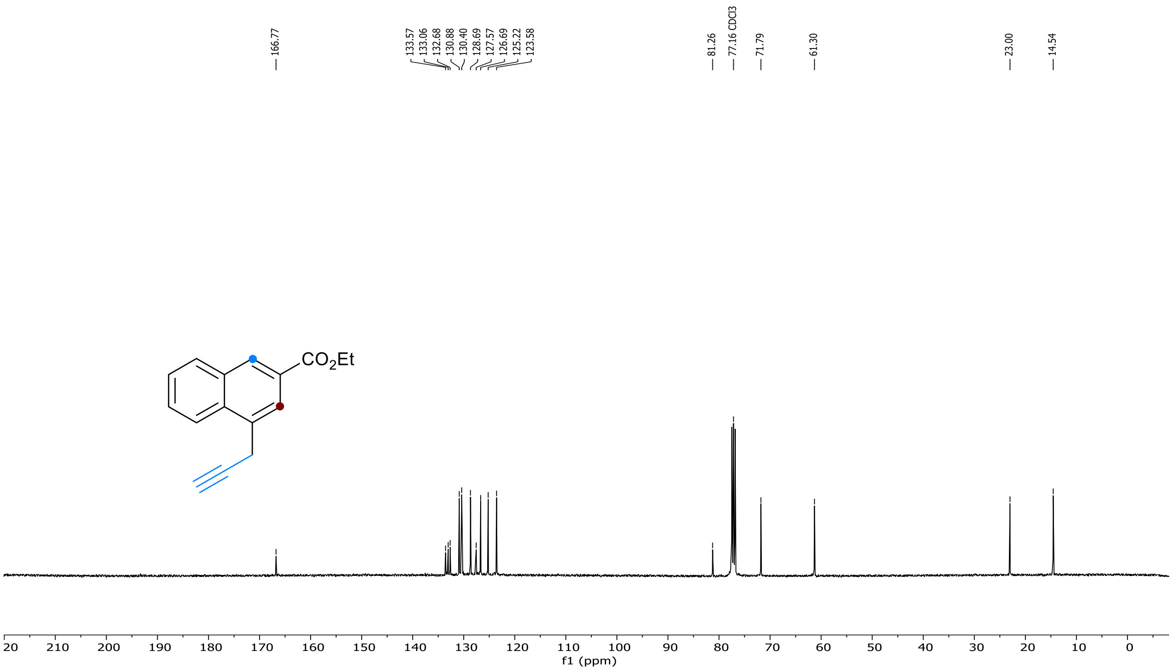

Compound **5p**:

^1H NMR (300 MHz, $CDCl_3$)

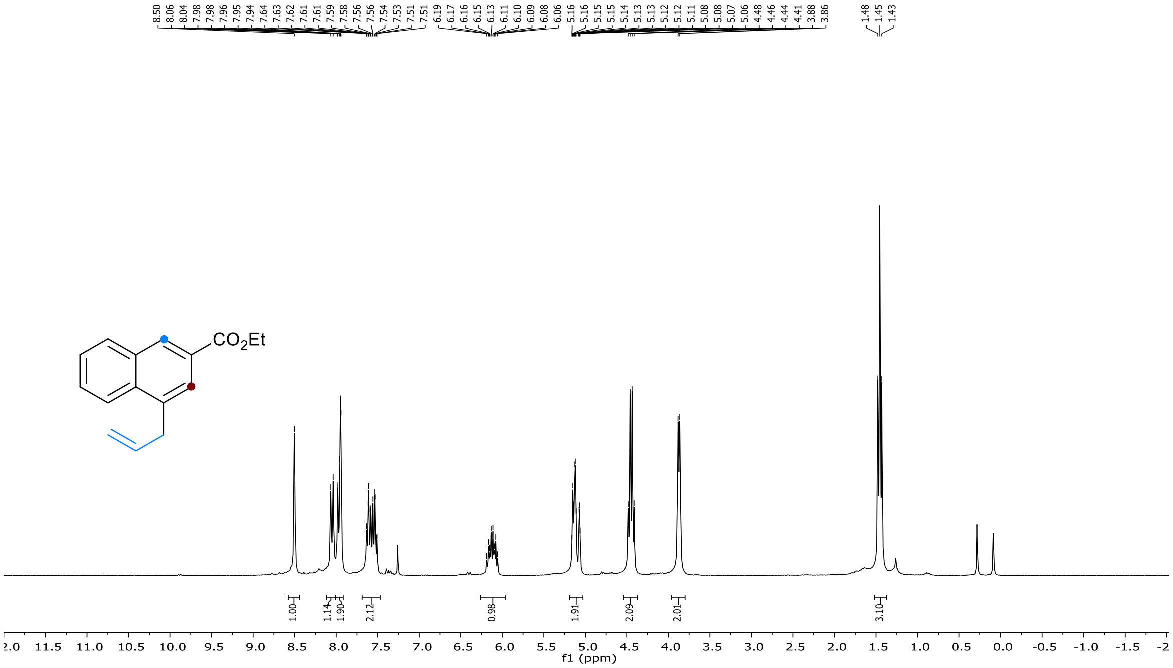

$^{13}C\{1H\}$ NMR (101 MHz, $CDCl_3$)

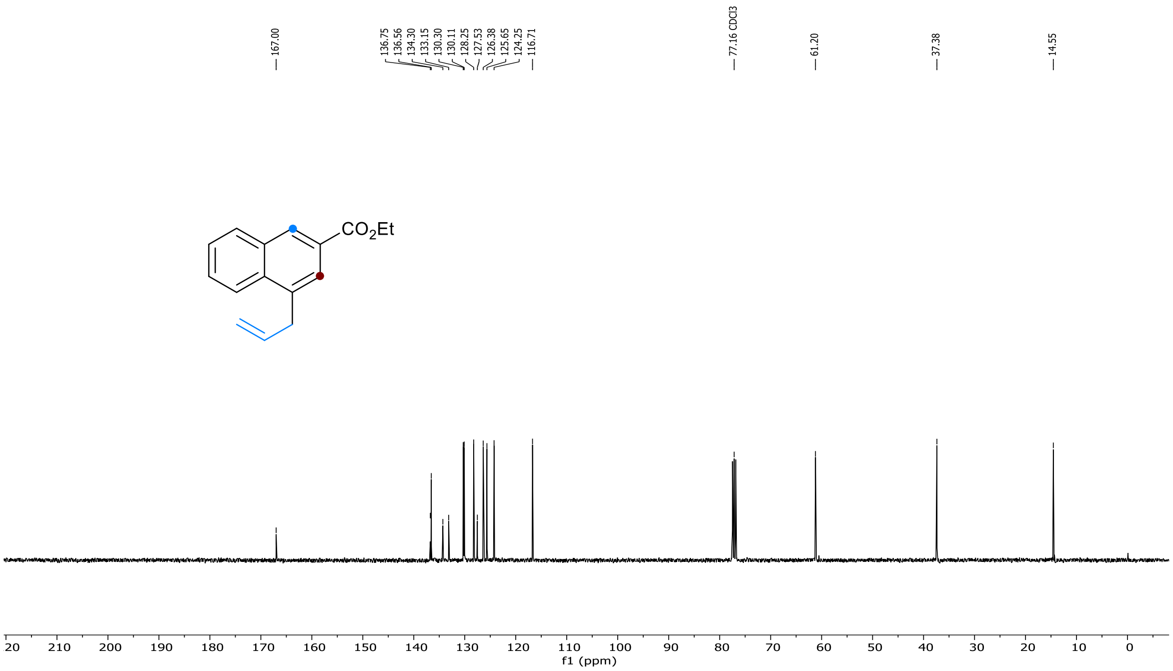

Compound **5q**:

^1H NMR (400 MHz, $CDCl_3$)

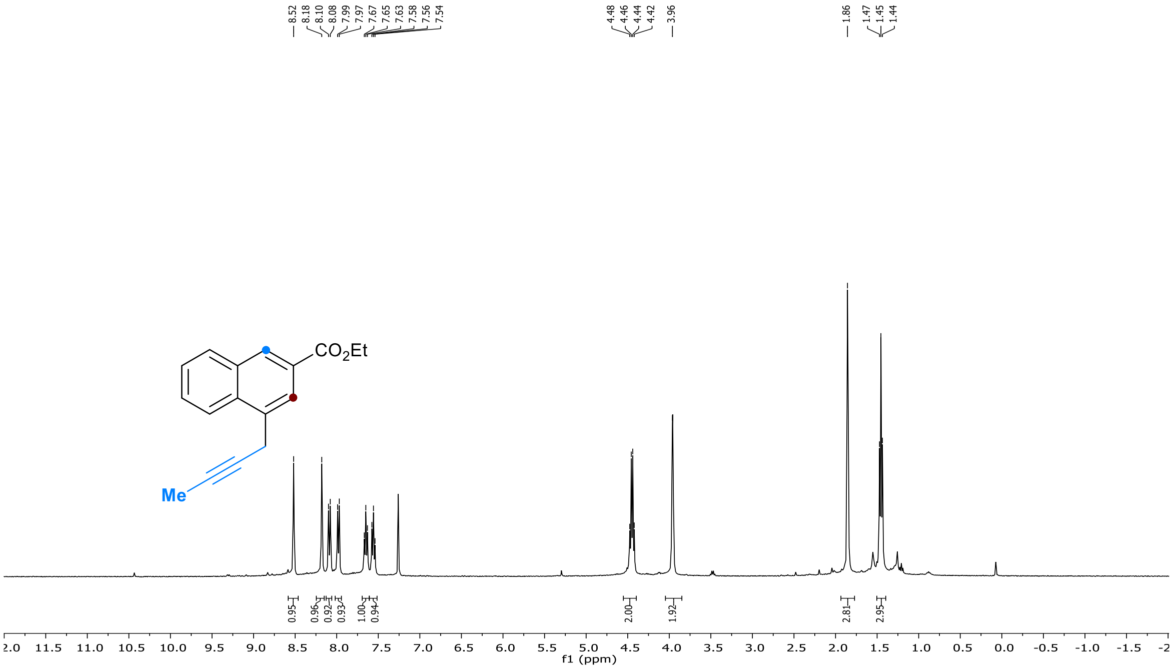

$^{13}C\{1H\}$ NMR (101 MHz, $CDCl_3$)

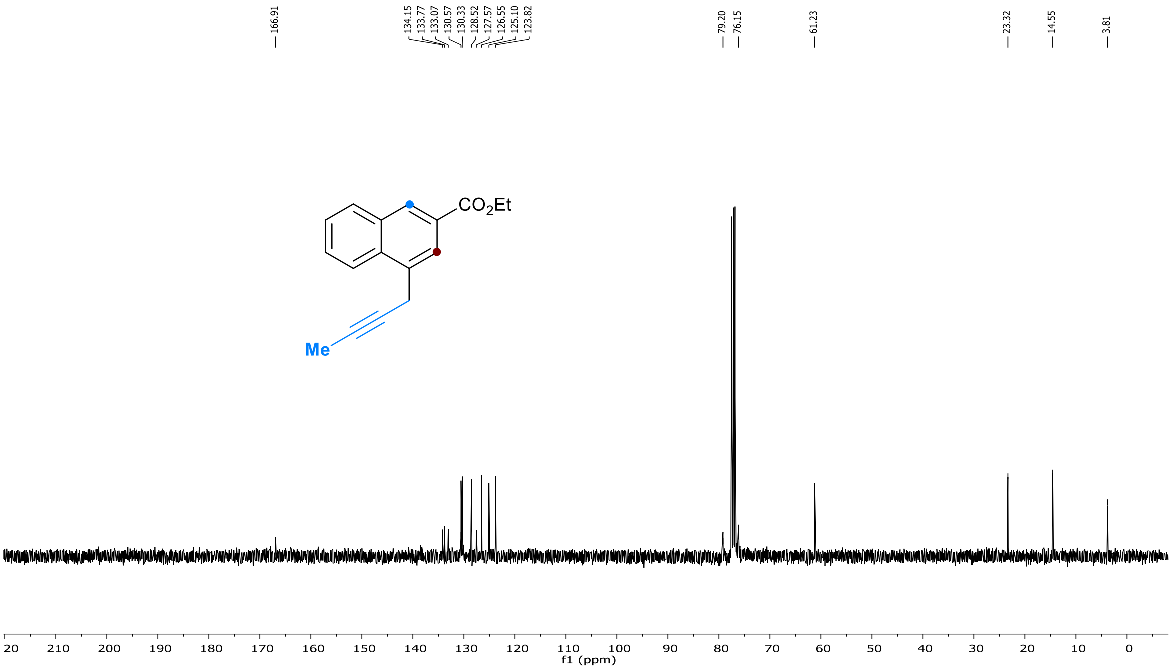

Compound **5r**:

^{1}H NMR (300 MHz, $CDCl_3$)

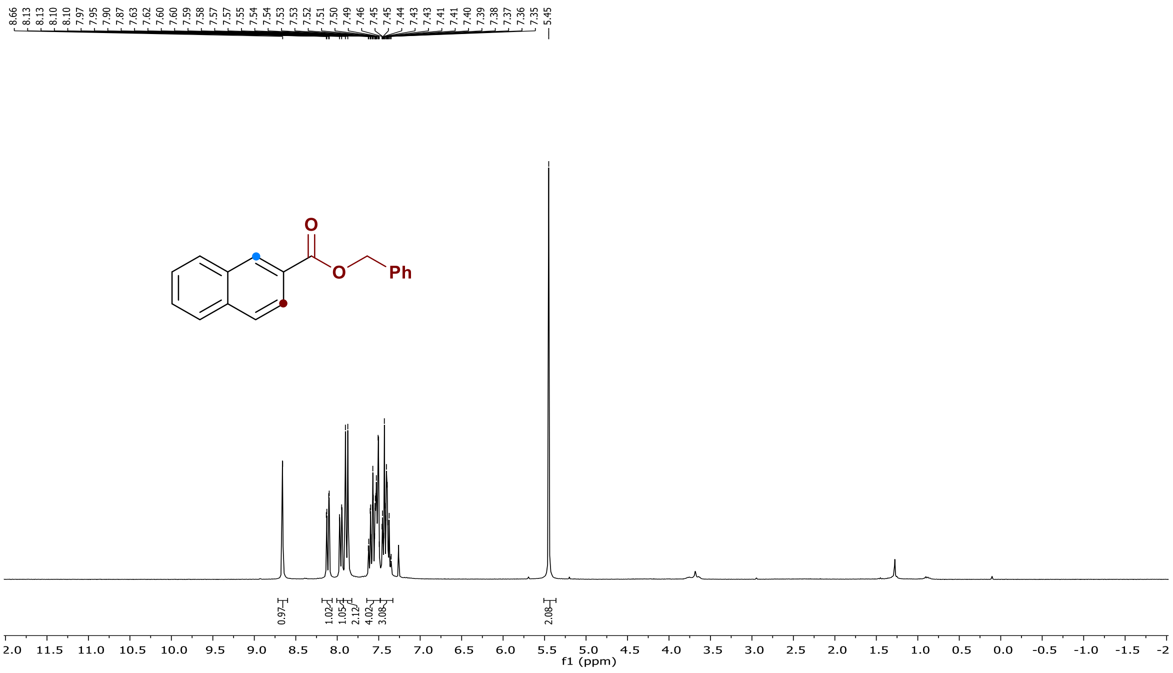

$^{13}C\{1H\}$ NMR (101 MHz, $CDCl_3$)

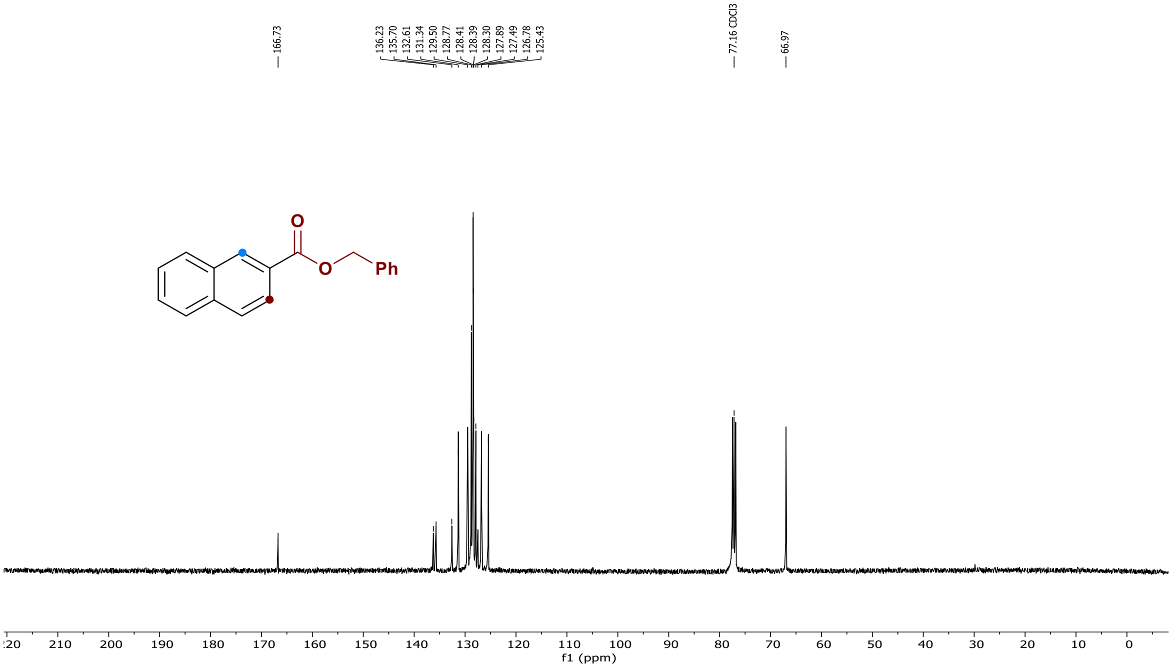

Compound **5s**:

^{1}H NMR (300 MHz, $CDCl_3$)

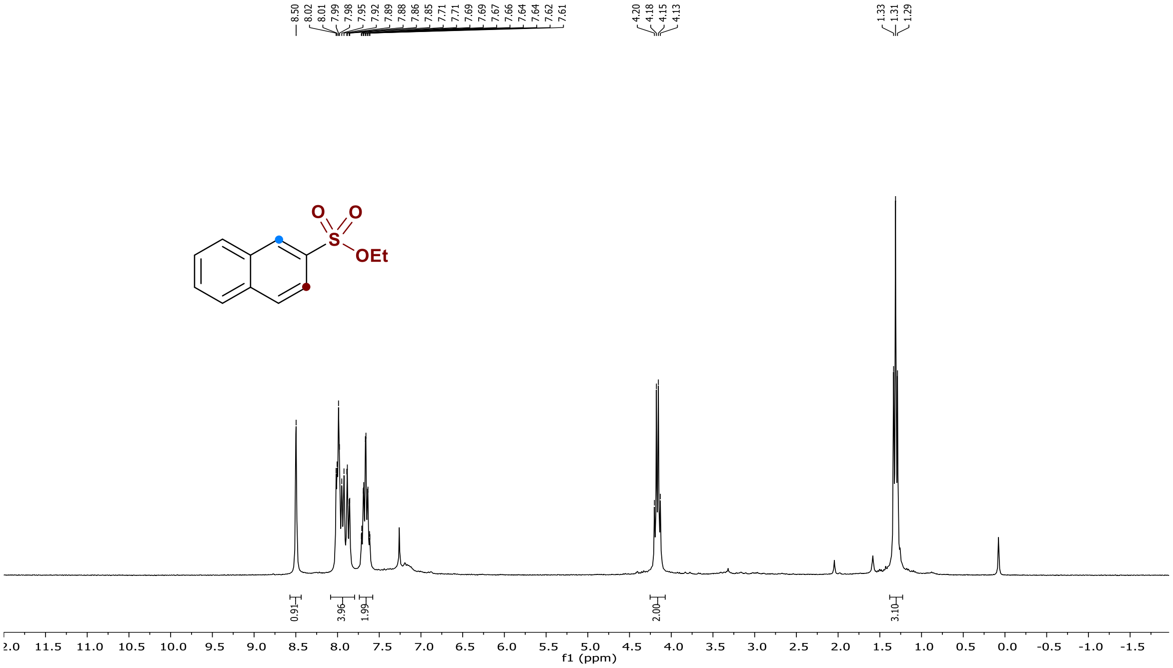

$^{13}C\{1H\}$ NMR (101 MHz, $CDCl_3$)

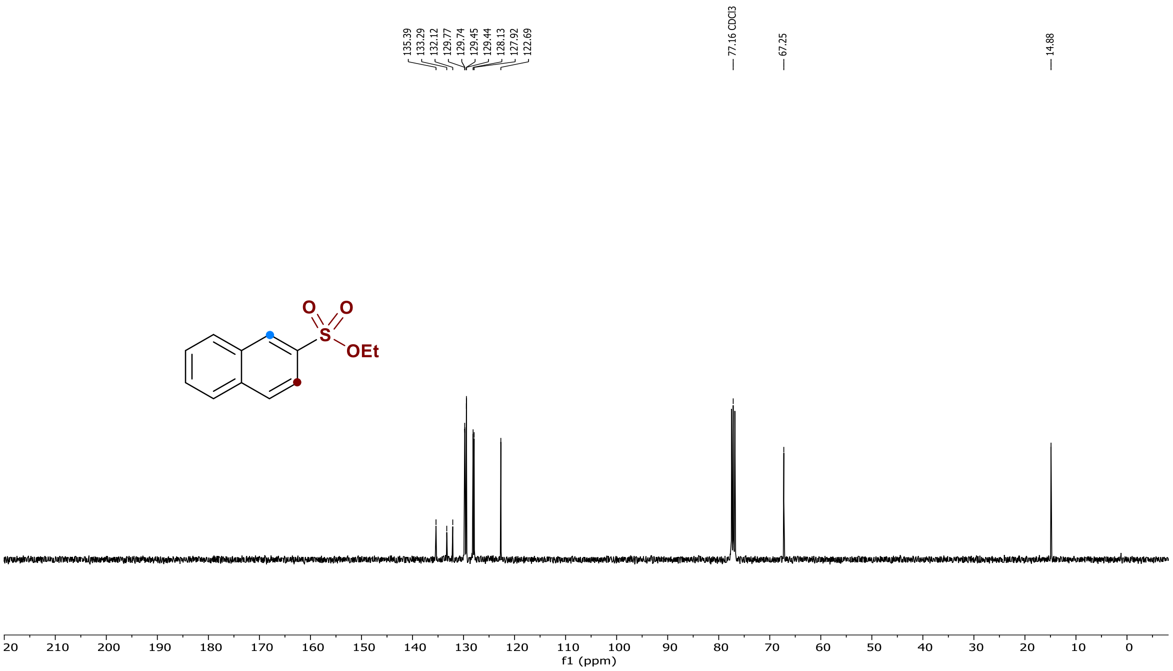

Compound **6b**:

^{1}H NMR (400 MHz, CD_3CN)

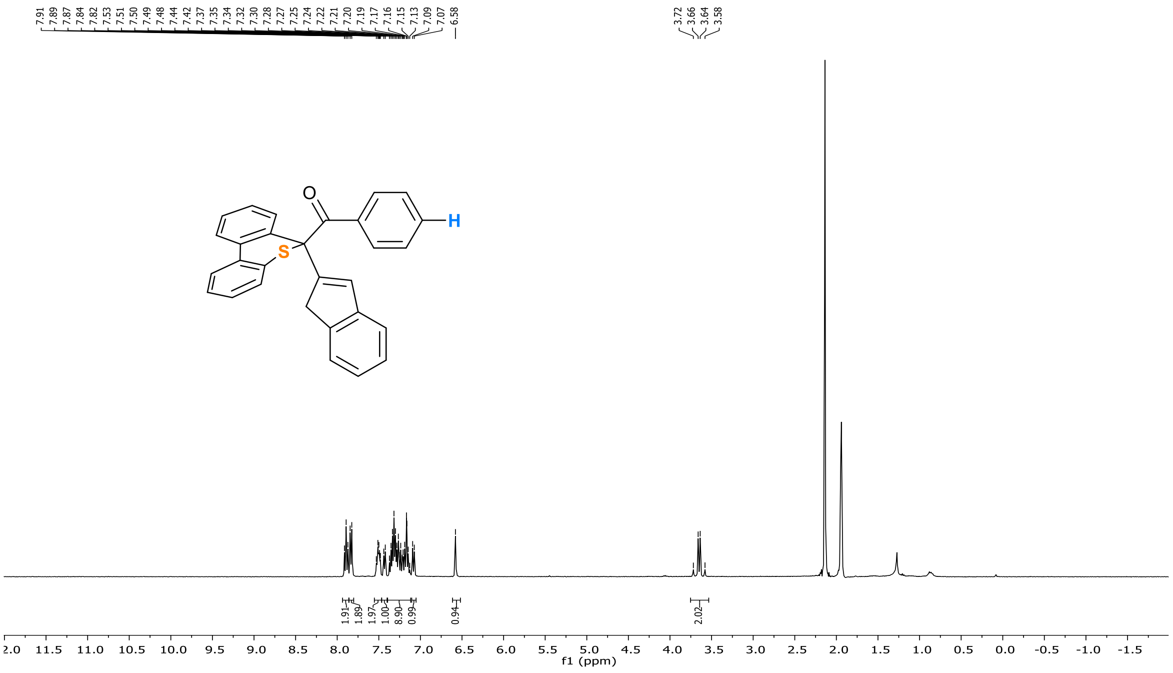

$^{13}C\{1H\}$ NMR (101 MHz, $CDCl_3$)

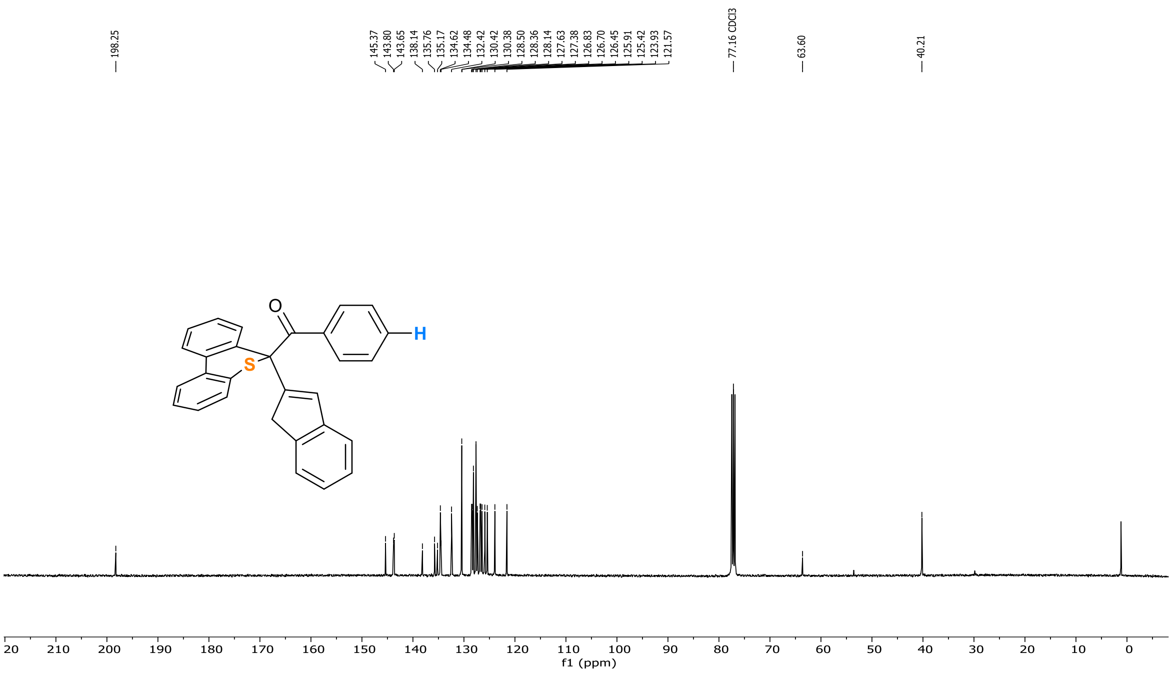

Compound **6c**:

^1H NMR (400 MHz, CD_3CN)

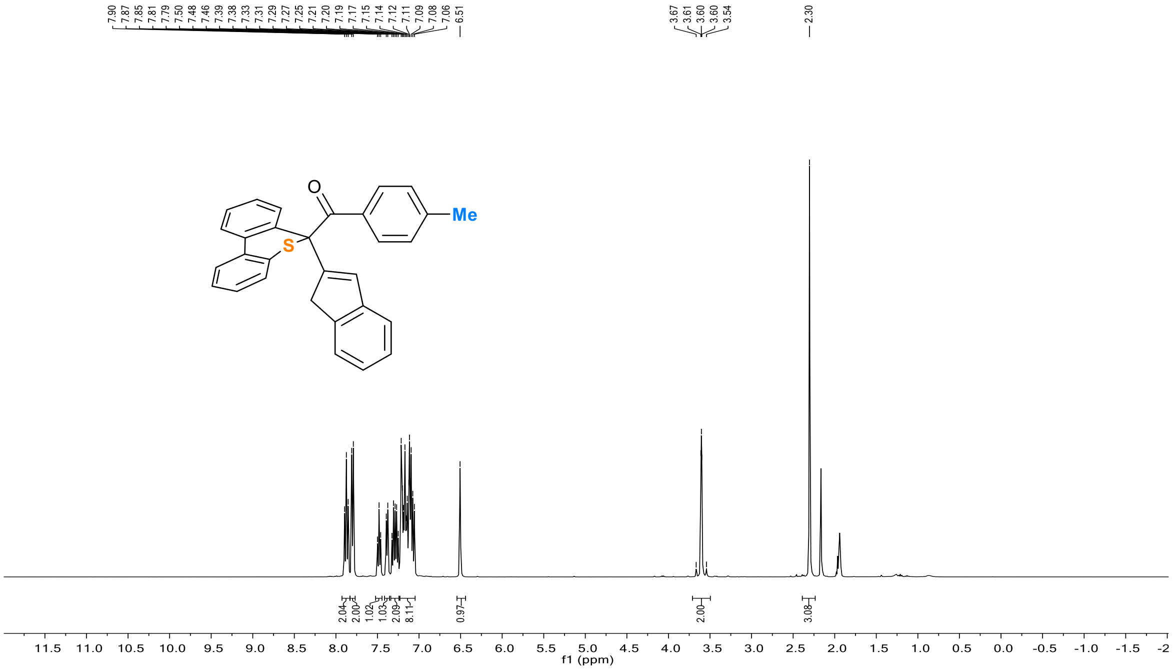

$^{13}C\{1H\}$ NMR (101 MHz, CD_3CN)

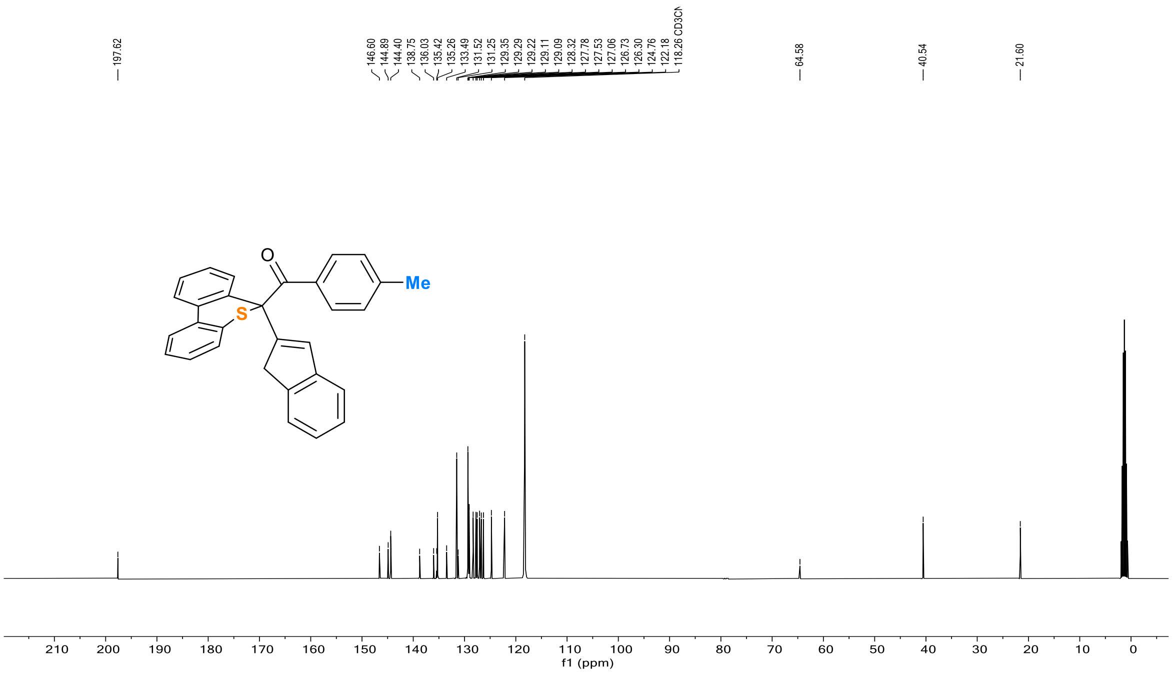

Compound **6d**:

^1H NMR (400 MHz, CD_3CN)

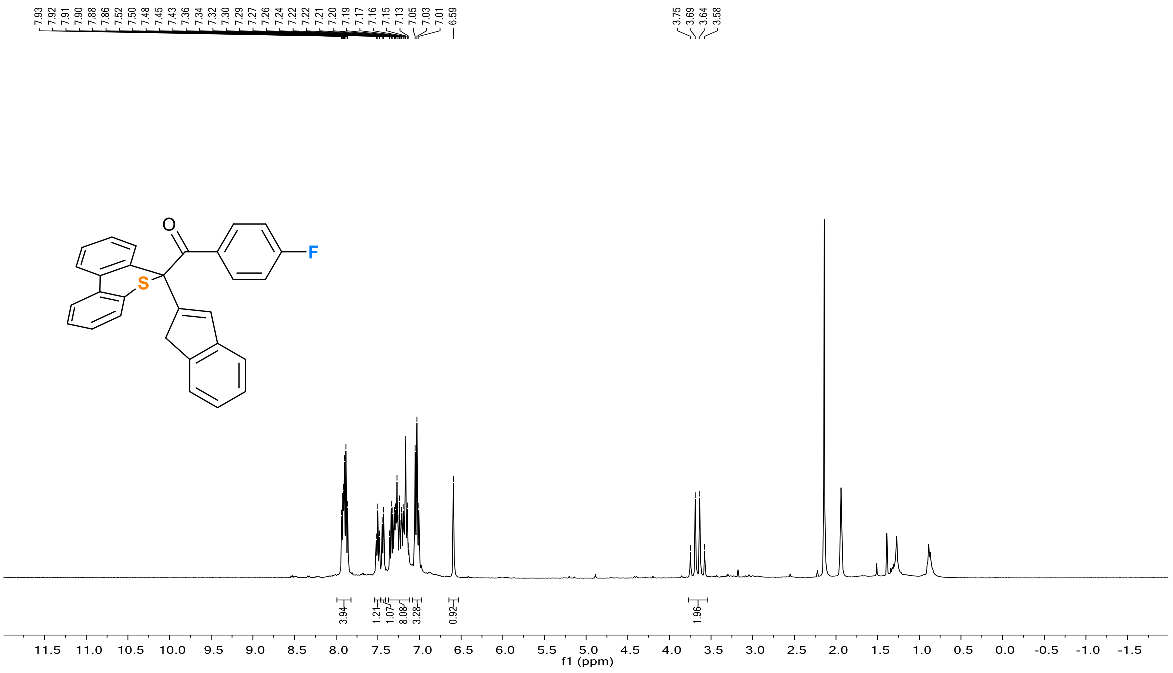

$^{13}C\{1H\}$ NMR (101 MHz, CD_3CN)

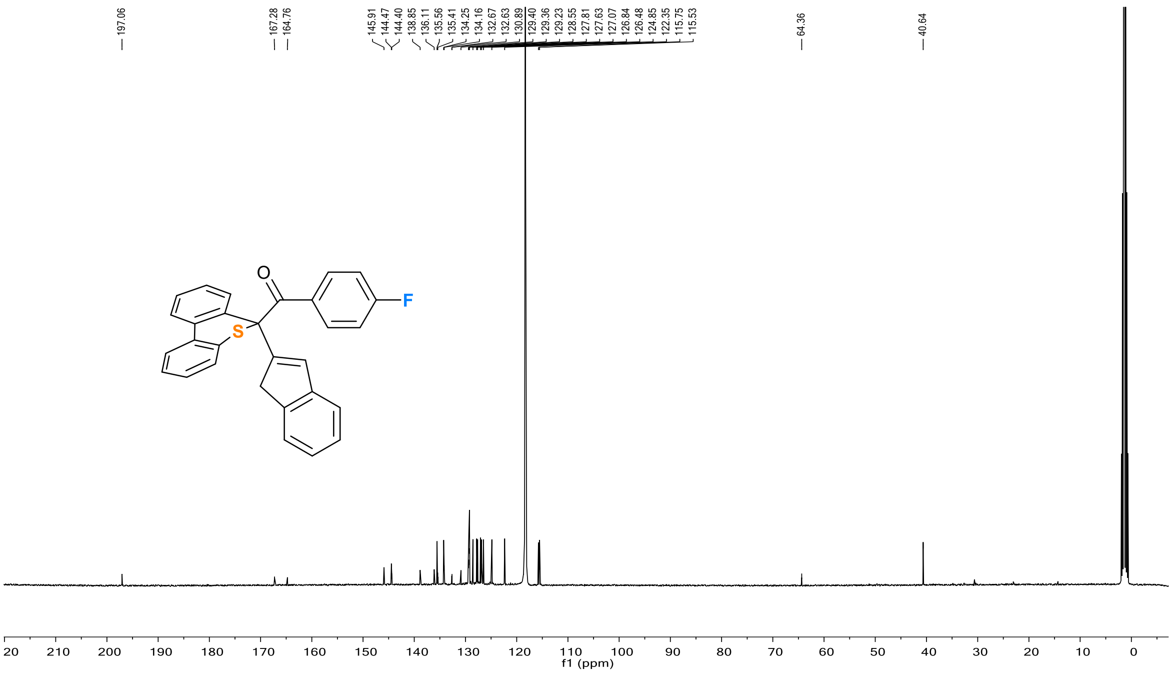

^{19}F NMR (377 MHz, CD_3CN)

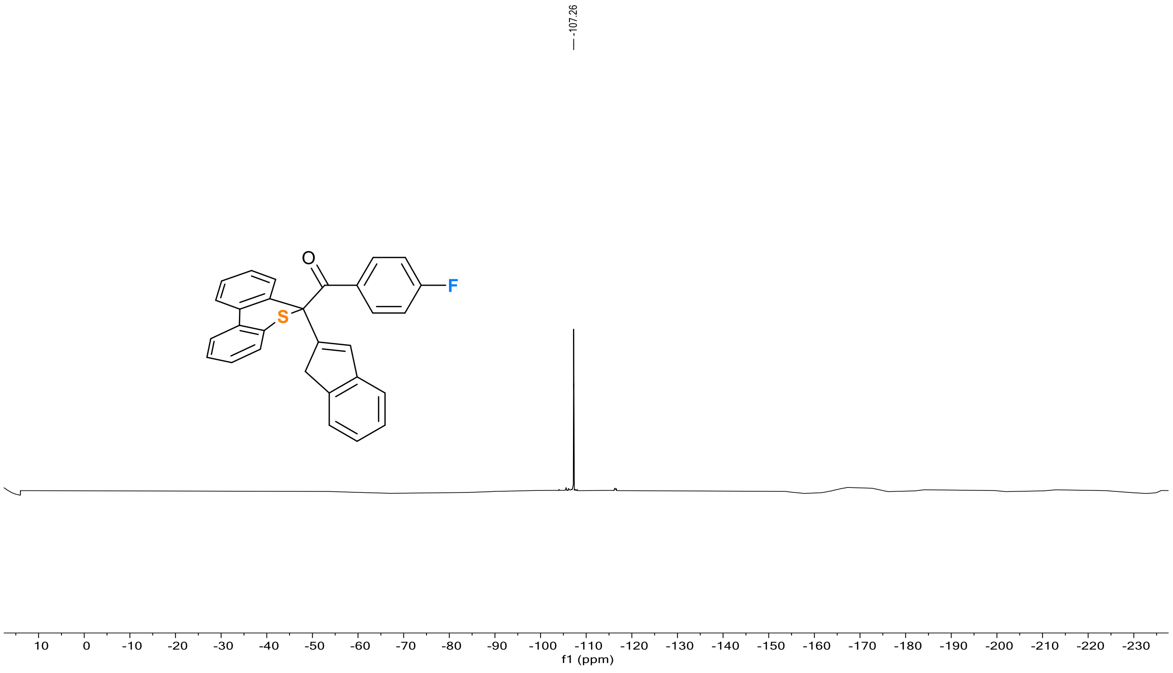

Compound **6e**:

^{1}H NMR (400 MHz, CD_3CN)

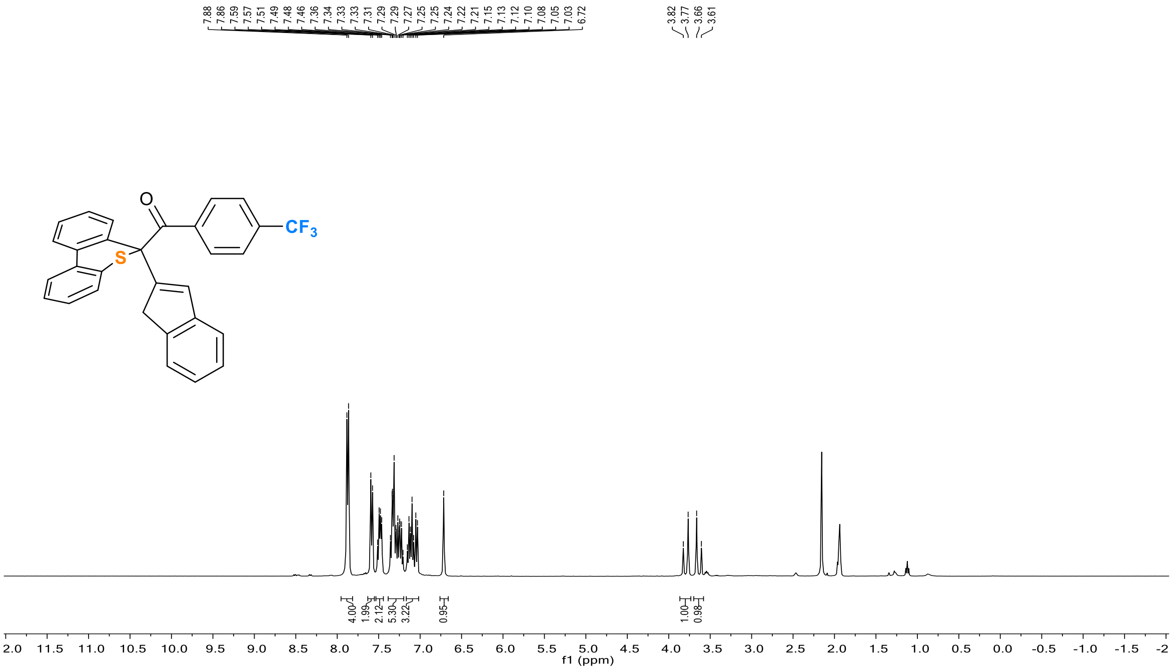

$^{13}C\{1H\}$ NMR (101 MHz, CD_3CN)

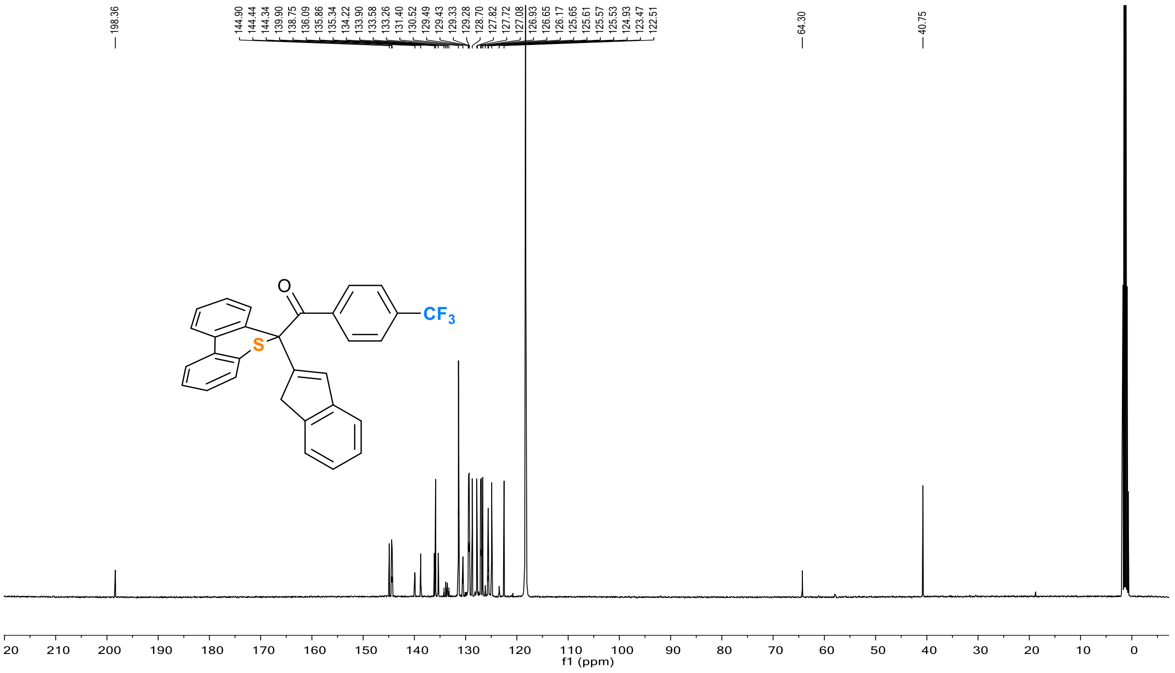

^{19}F NMR (377 MHz, CD_3CN)

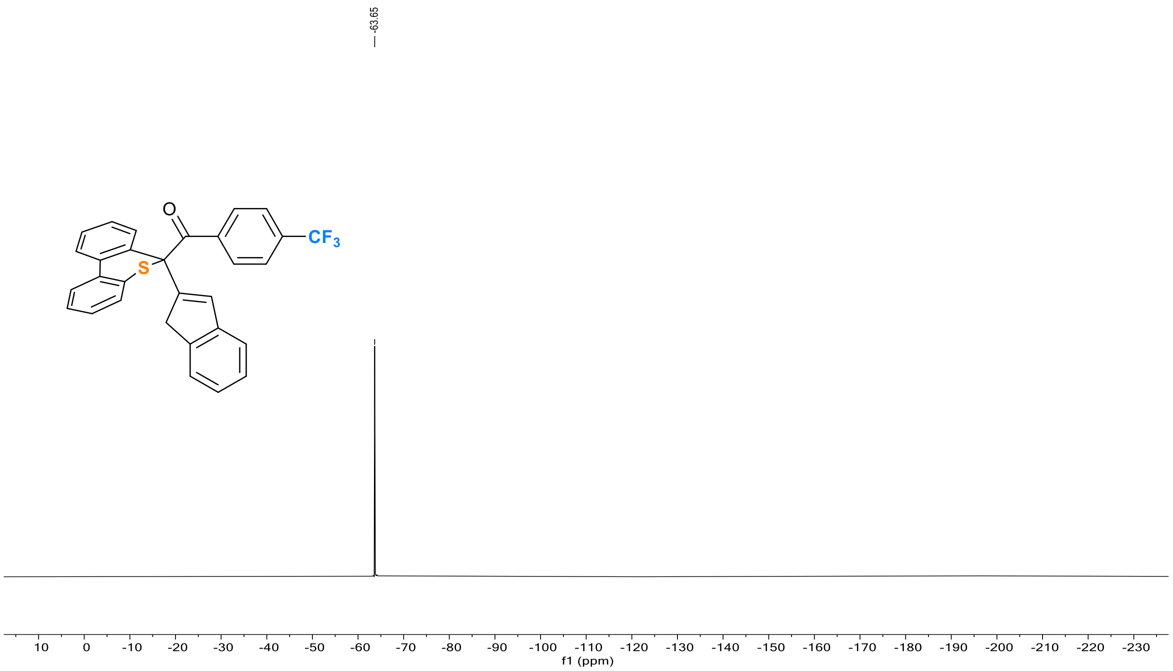

Compound **7c**

^1H NMR (400 MHz, $CDCl_3$)

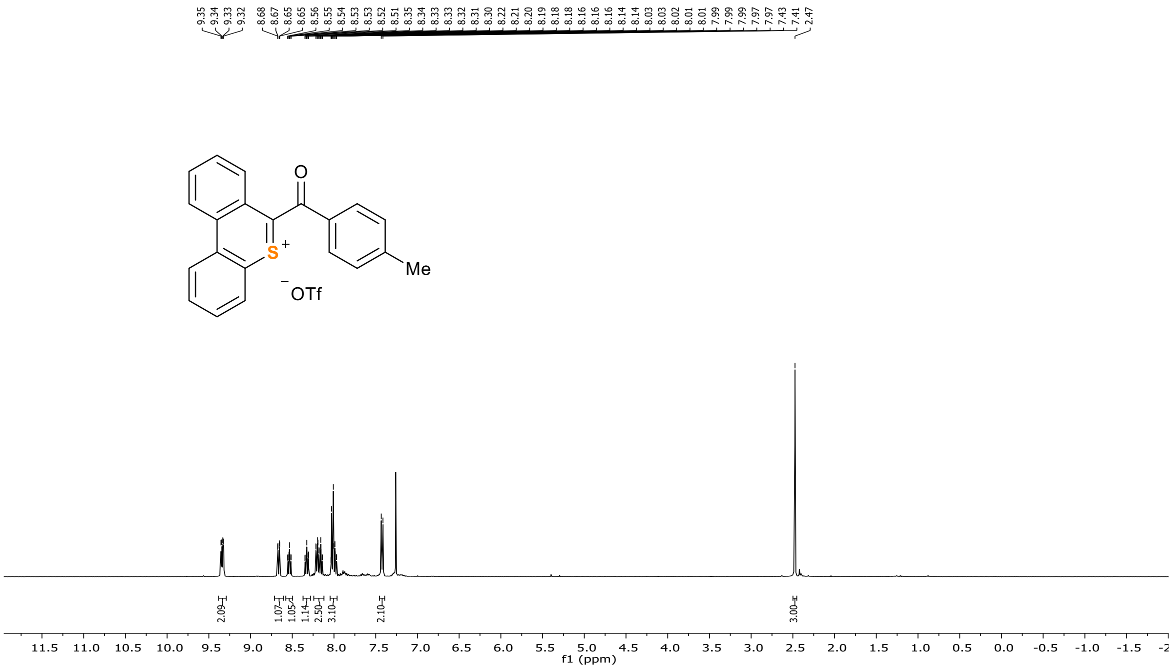

$^{13}C\{1H\}$ NMR (101 MHz, $CDCl_3$)

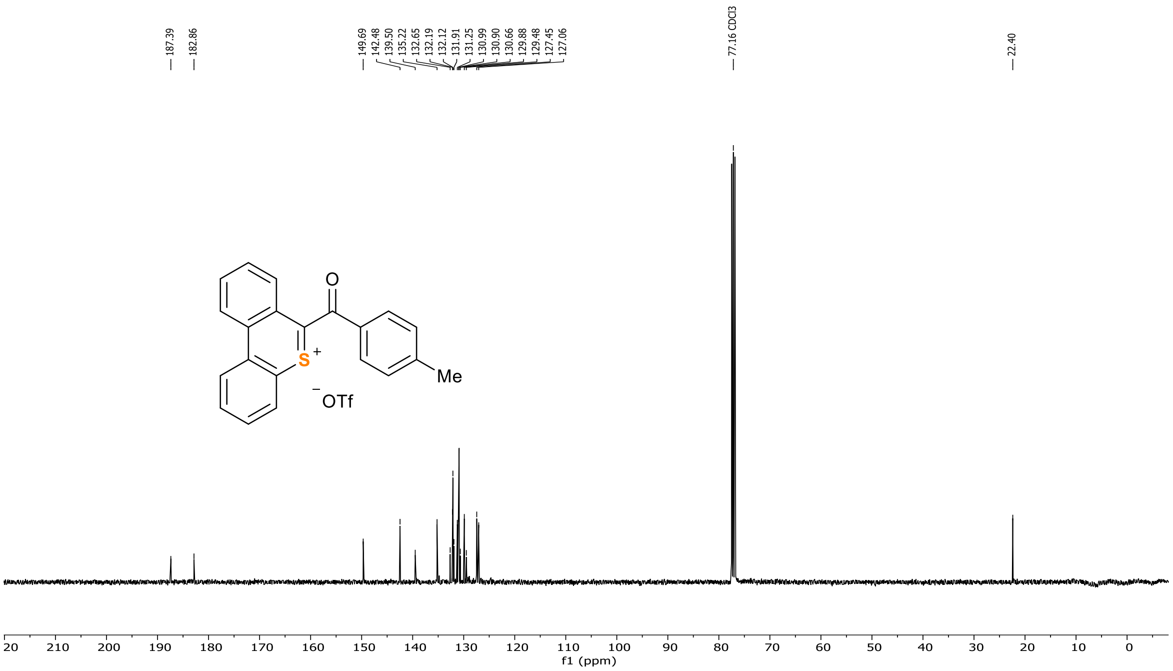

^{19}F NMR (377 MHz, $CDCl_3$)

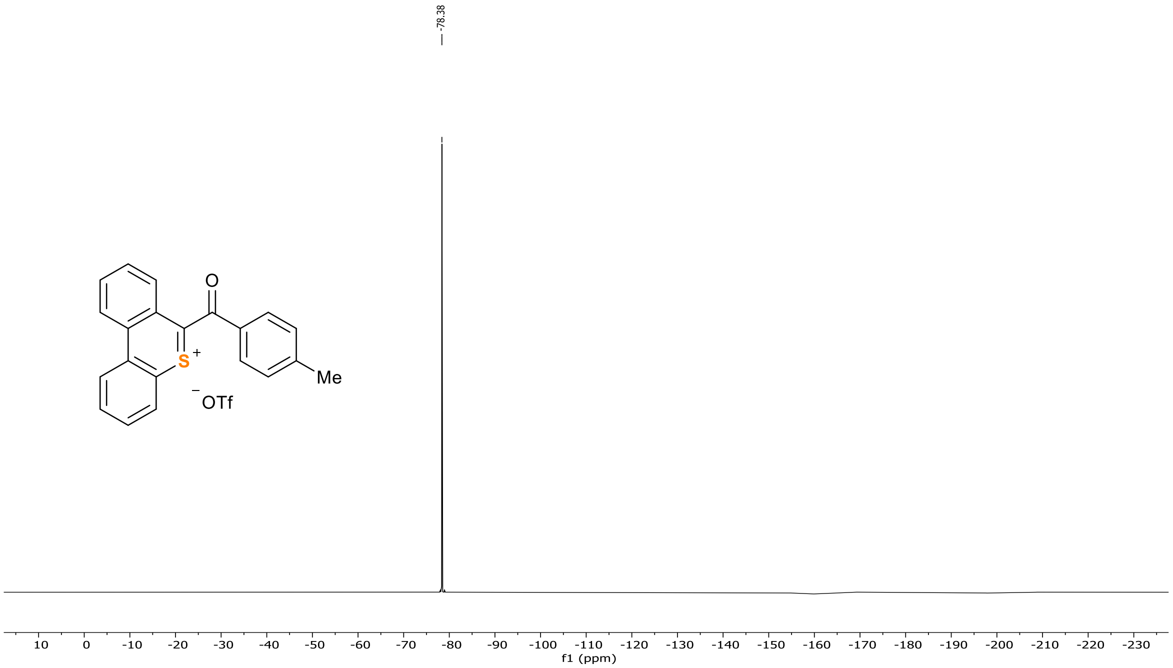

Compound **8c**:

^{1}H NMR (400 MHz, CD_3CN)

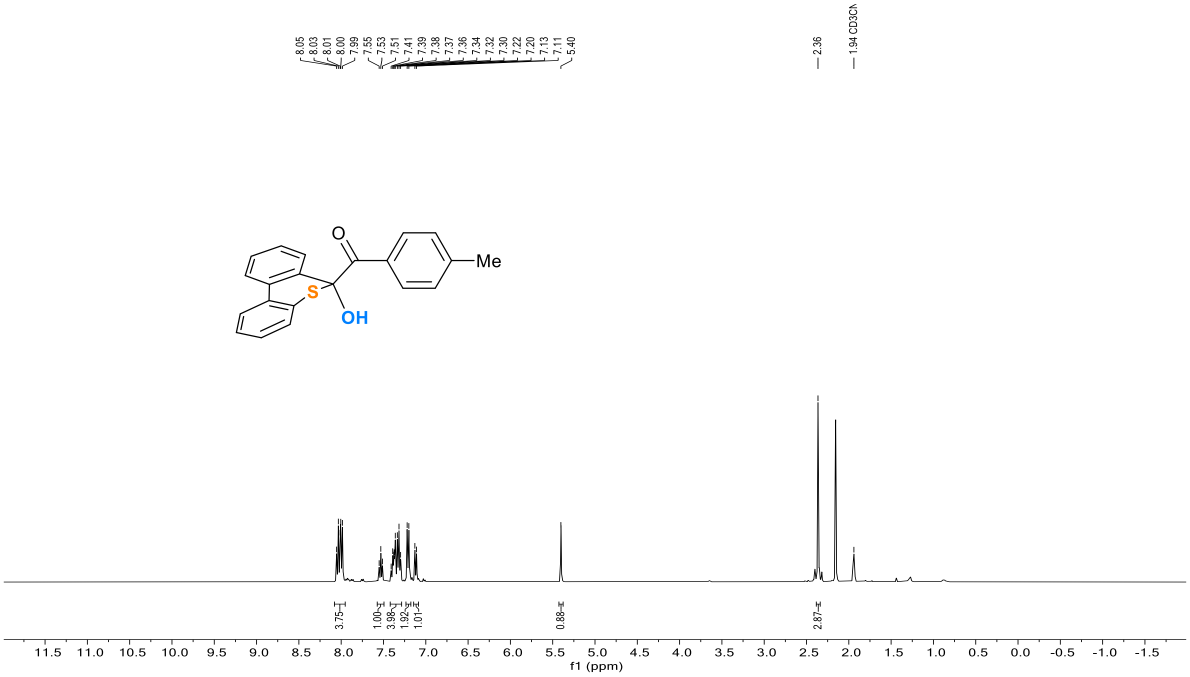

$^{13}C\{1H\}$ NMR (101 MHz, CD_3CN)

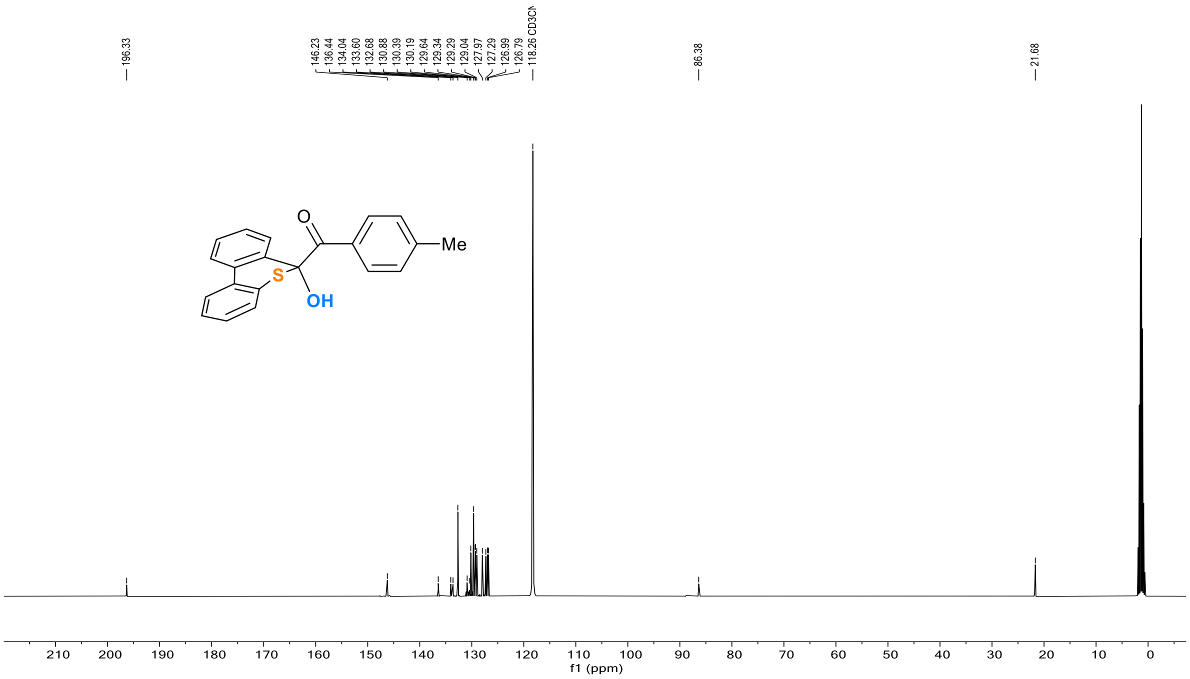

Compound **9a**:

^{1}H NMR (300 MHz, $CDCl_3$)

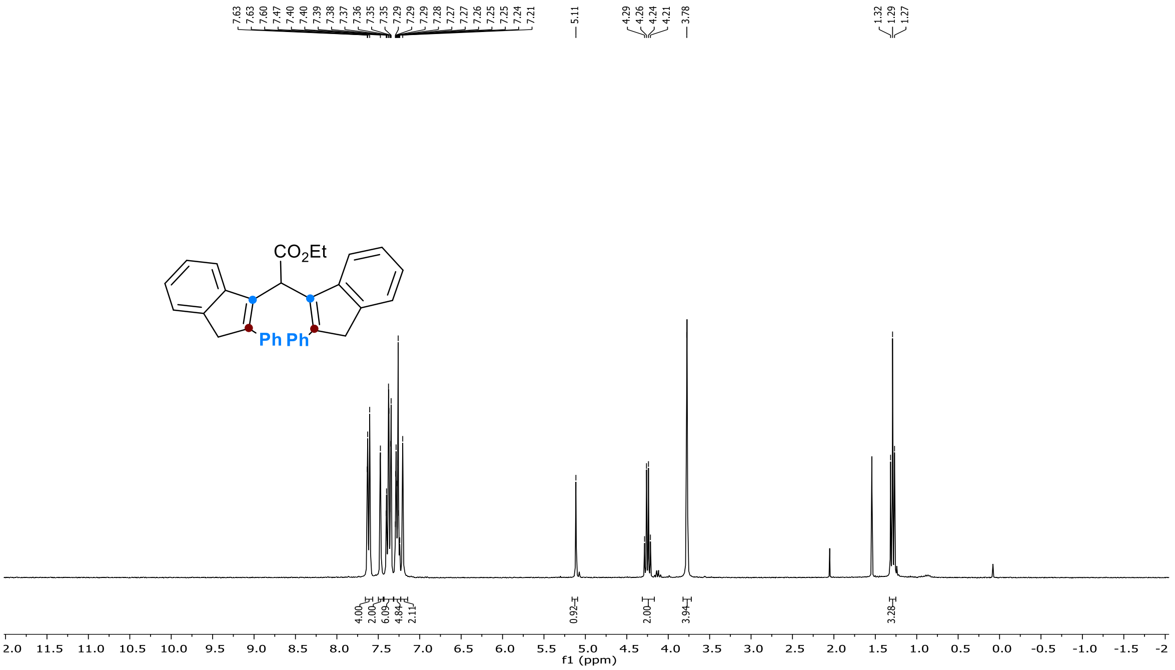

APT $^{13}C\{1H\}$ NMR (101 MHz, $CDCl_3$)

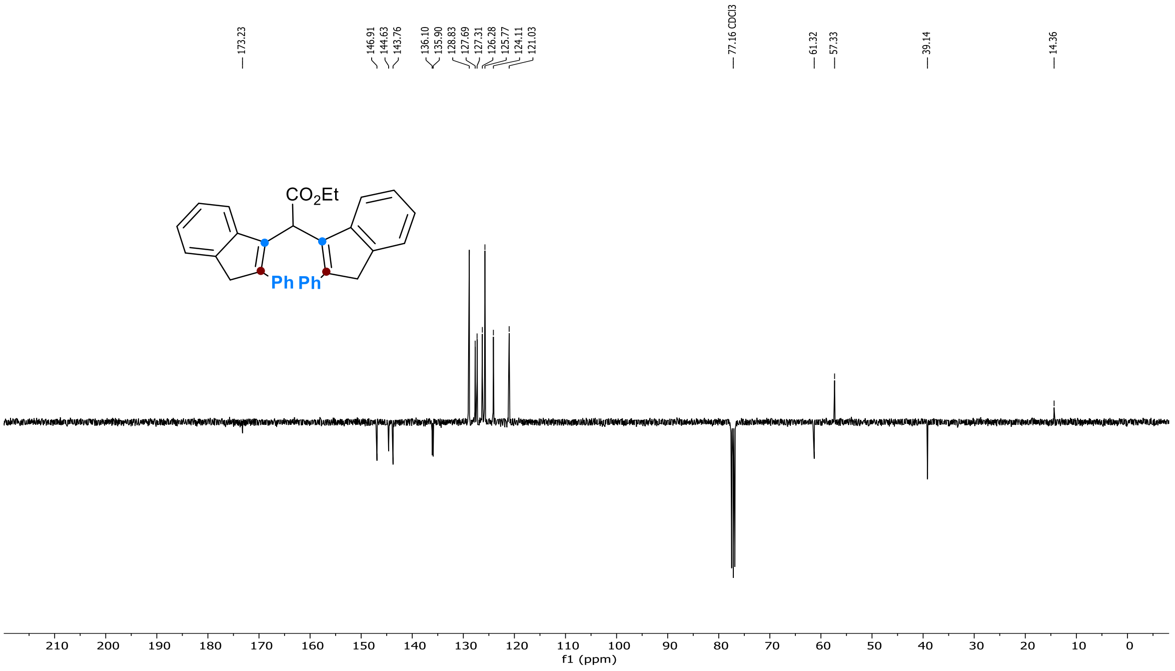

Compound **9b**:

^{1}H NMR (300 MHz, $CDCl_3$)

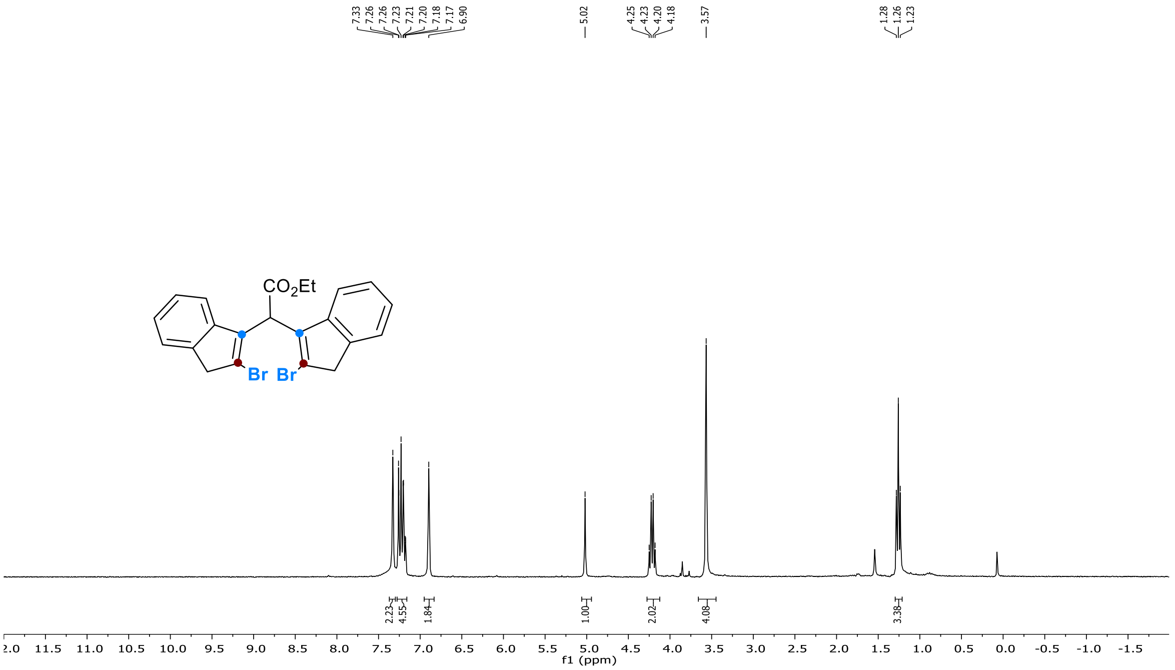

^{13}C{1H} NMR (75 MHz, $CDCl_3$)

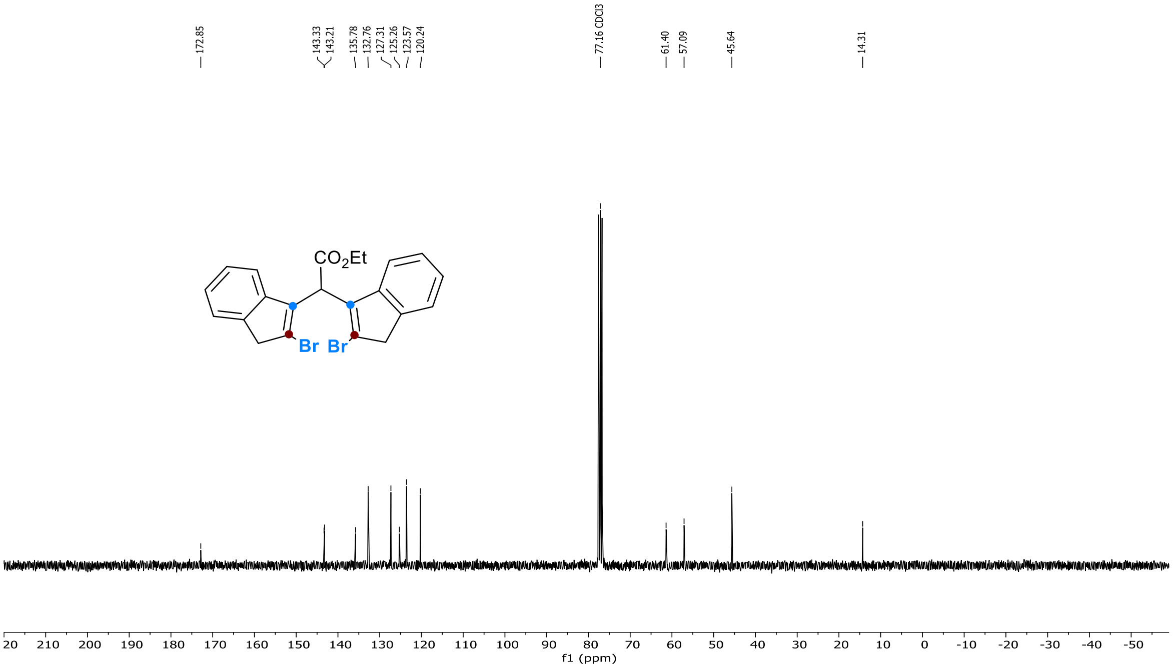

Compound **10f**:

^{1}H NMR (400 MHz, CD_3CN)

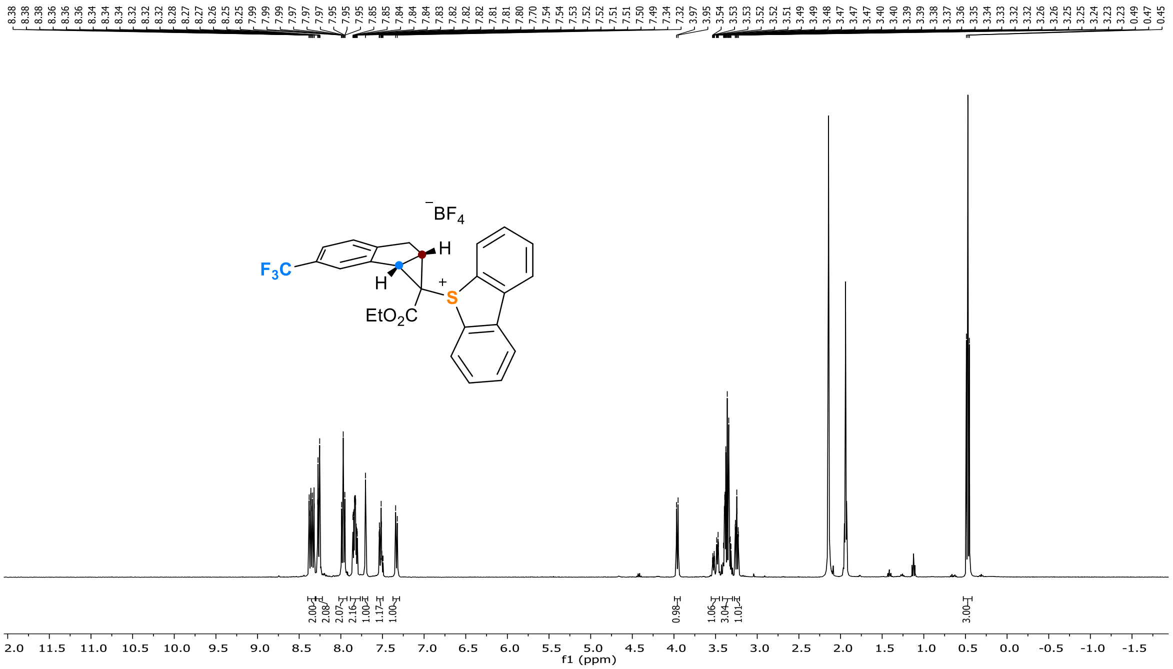

$^{13}C\{1H\}$ NMR (101 MHz, CD_3CN)

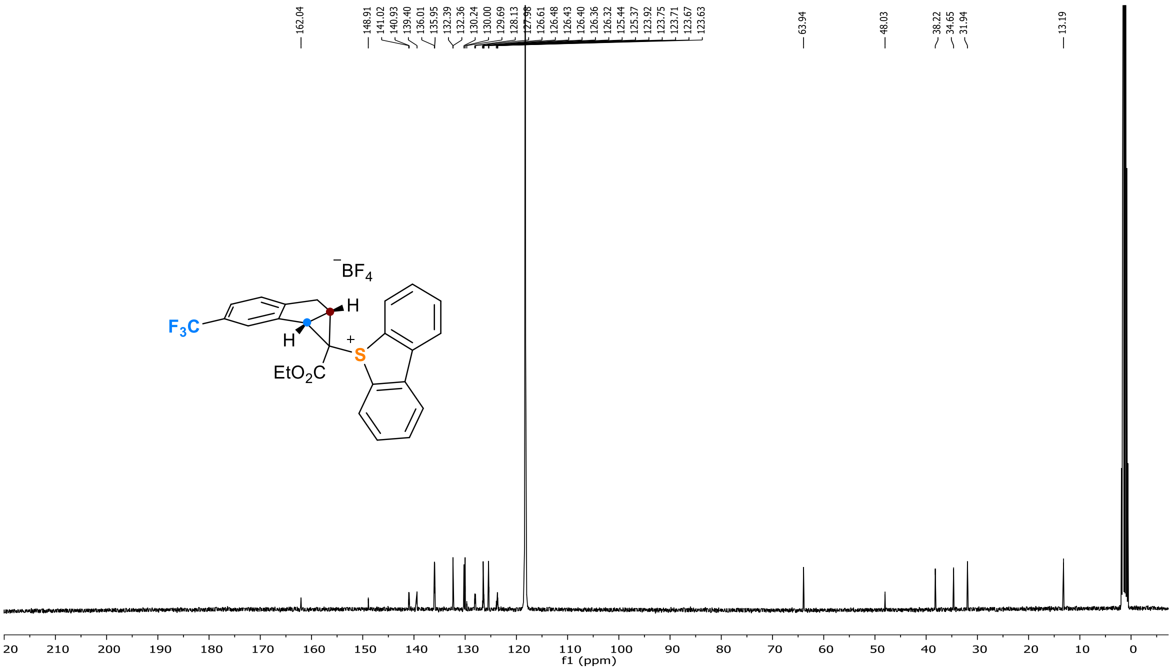

^{19}F NMR (377 MHz, CD_3CN)

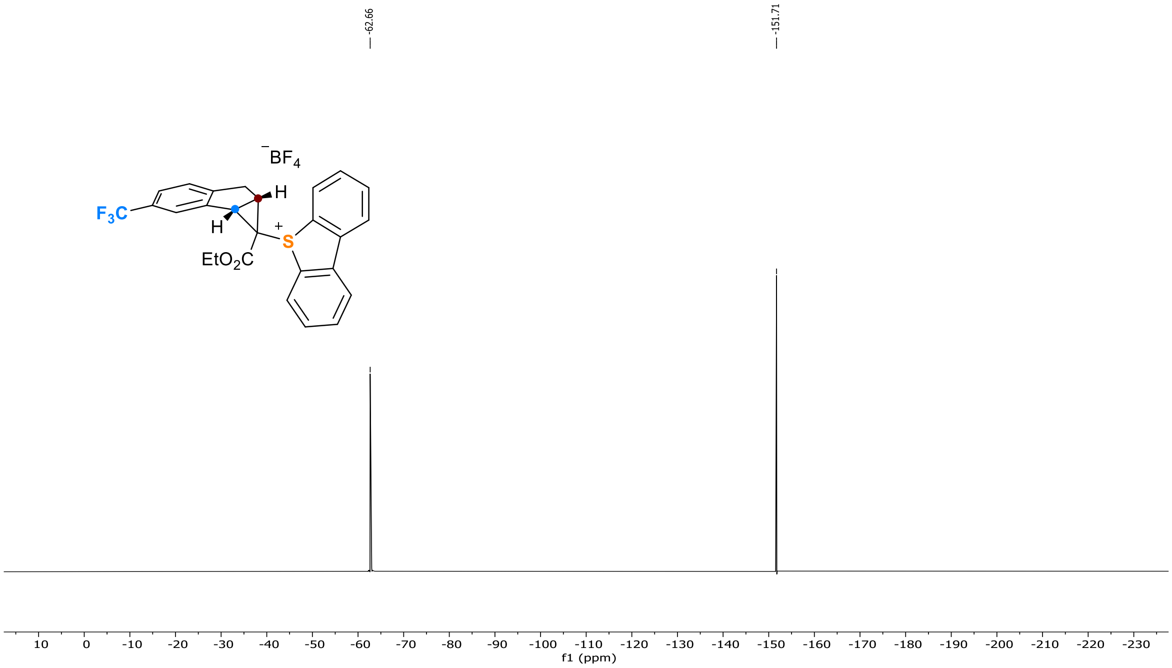

Compound **10i**:

^{1}H NMR (400 MHz, CD_3CN)

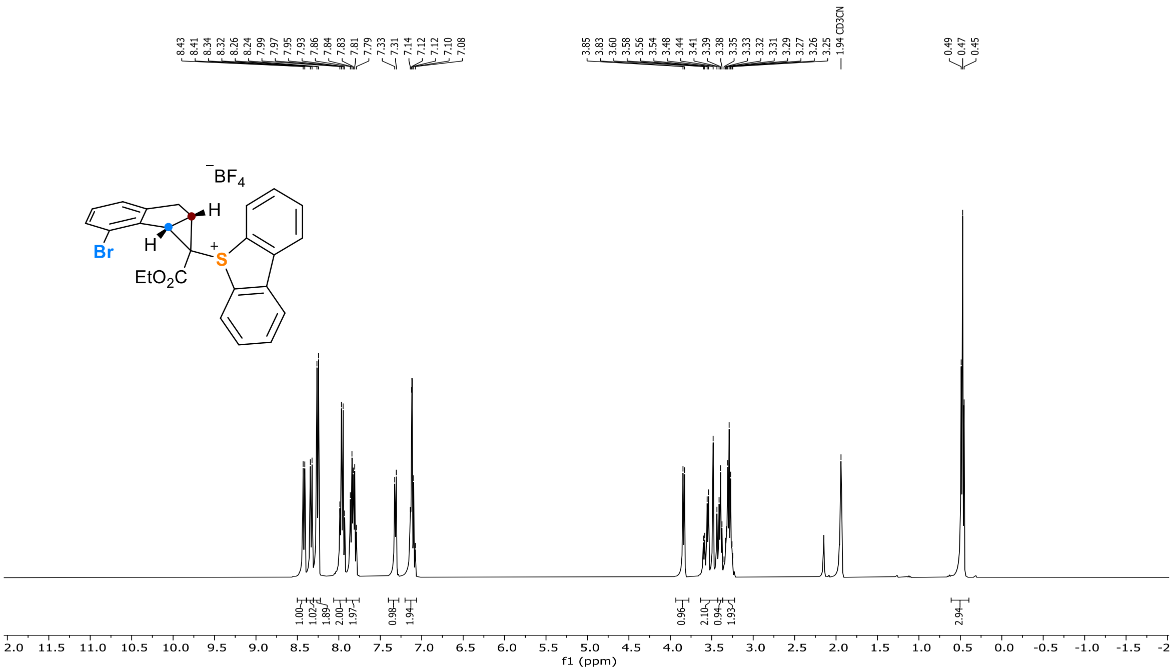

$^{13}C\{1H\}$ NMR (101 MHz, CD_3CN)

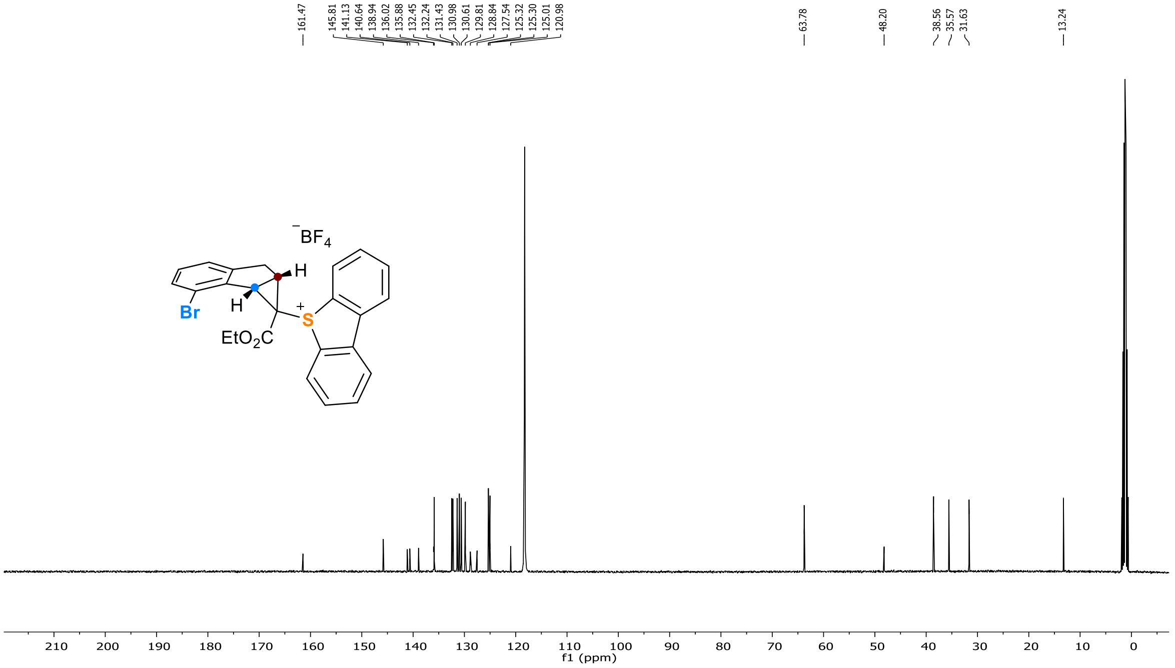

^{19}F NMR (377 MHz, CD_3CN)

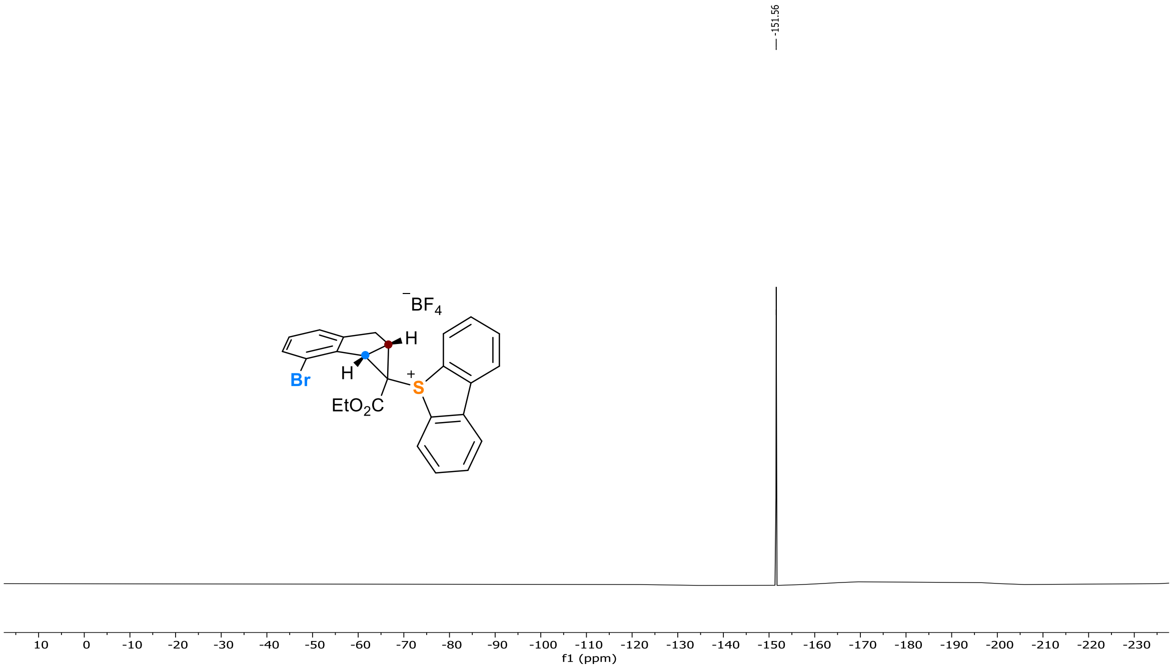

Compound **10t**:

^{1}H NMR (400 MHz, CD_3CN)

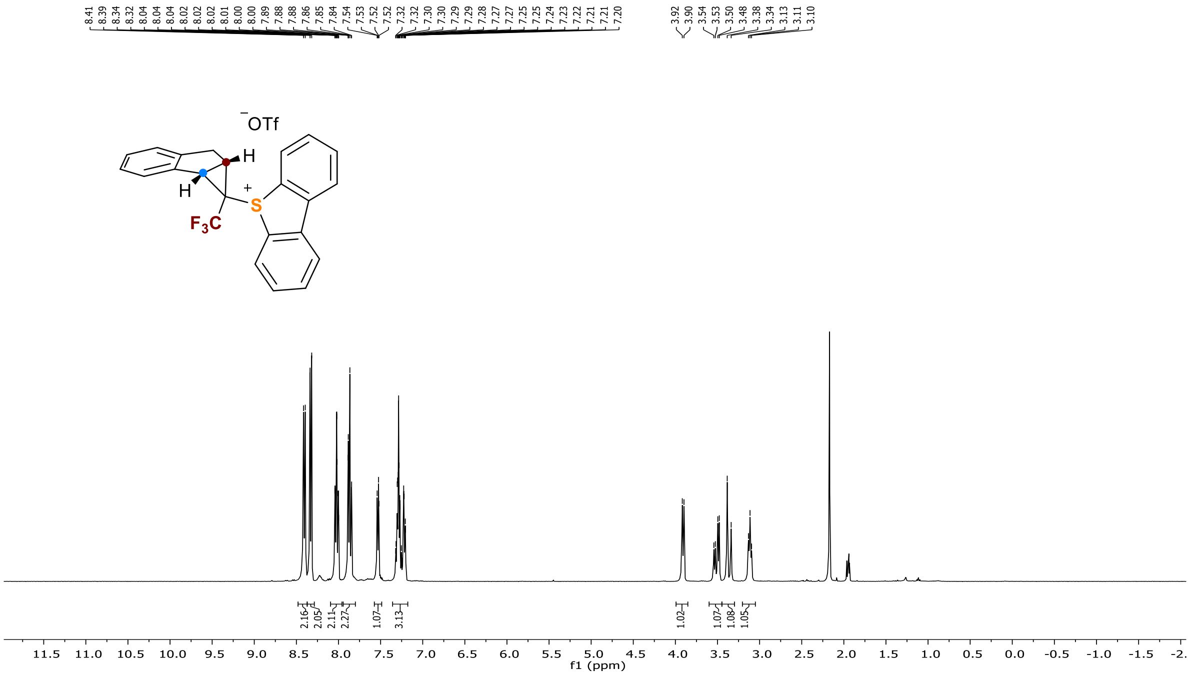

^{13}C{1H} NMR (101 MHz, CD_3CN)

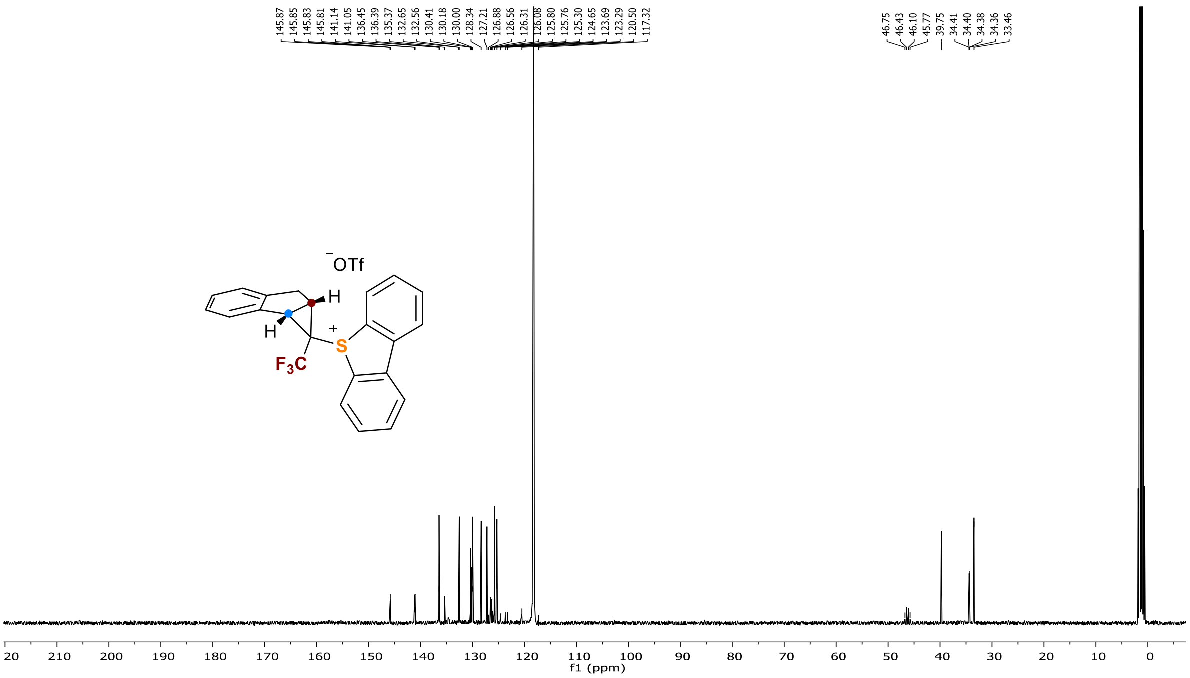

^{19}F NMR (377 MHz, CD_3CN)

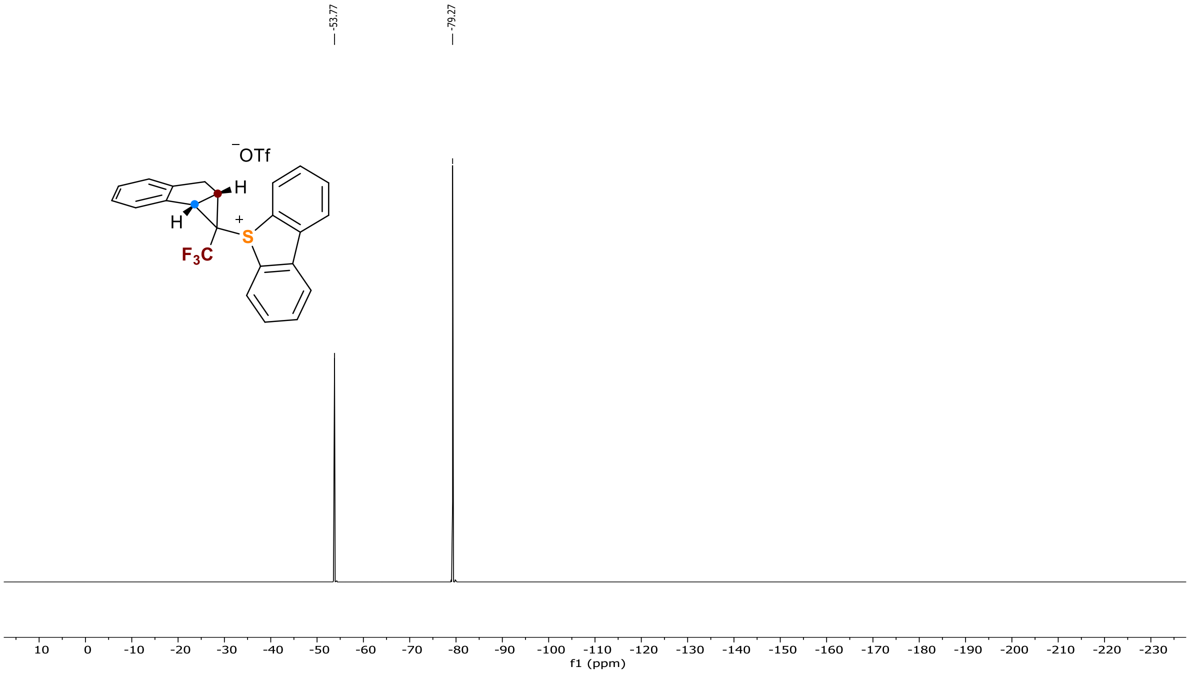

Compound **11**:

^{1}H NMR (300 MHz, $CDCl_3$)

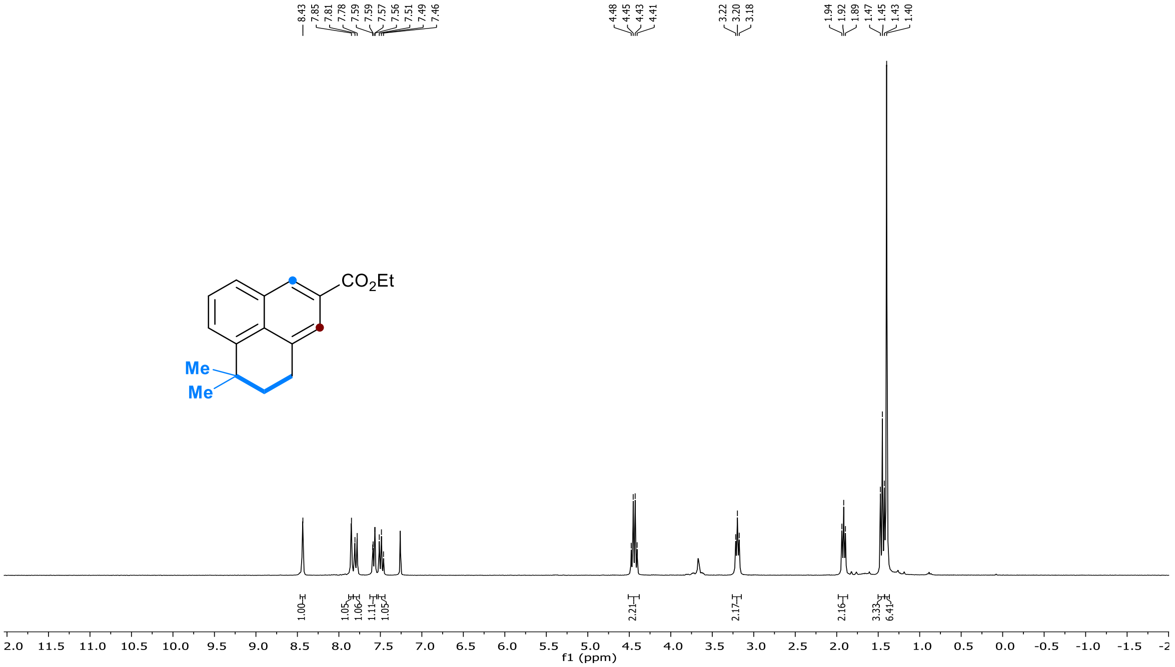

$^{13}C\{1H\}$ NMR (75 MHz, $CDCl_3$)

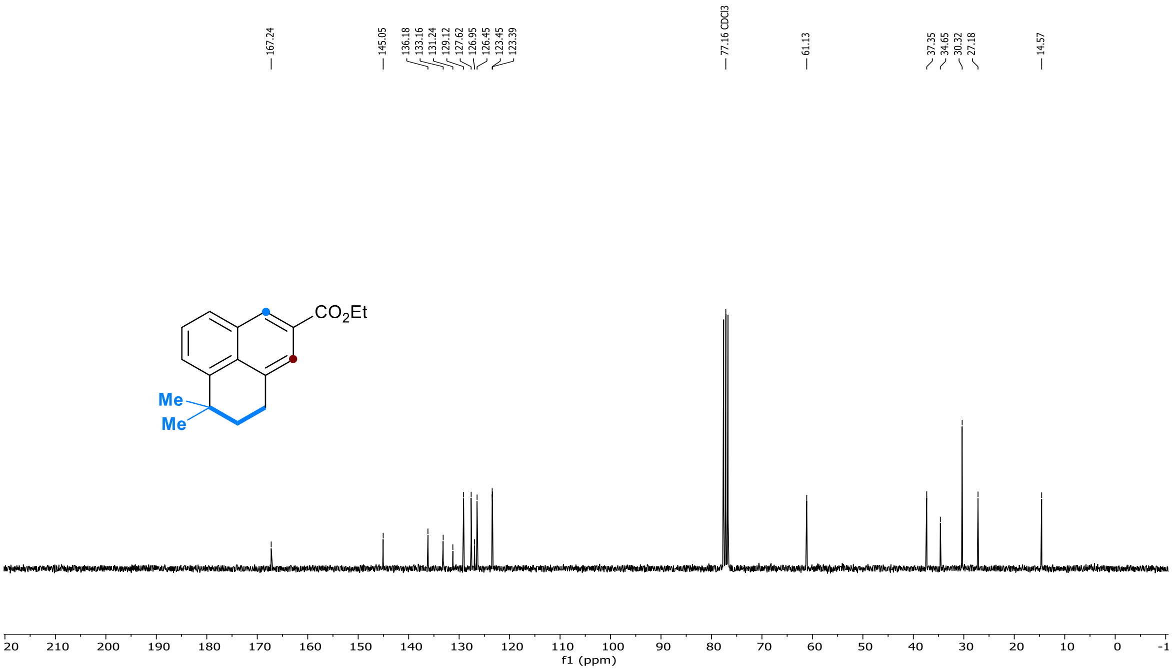

COMPUTATIONAL STUDIES

Calculations were carried out with Gaussian 16, Rev. A.03, and output files were analyzed with GaussView 6.0.19.[33] Molecular overlay was constructed using the structure overlay plugin in OLEX^2 and the coordinates from the geometry optimizations.

Absolute electronic energies for calculation of ΔH, ΔG and ΔS are listed in the table below:

	Electronic Energy (EE) [Hartree]	EE + Thermal Free Energy Correction [Hartree]
Int(endo)	-1514.265696	-1513.925993
Int(exo)	-1514.259025	-1513.900864
TS1(endo)	-1514.237827	-1513.920676
TS1(exo)	-1514.19972	-1513.865049

Optimized geometry of minima and transition states (cartesian coordinates) as well as used Gaussian command lines (following #) are supplied. Intrinsic reaction coordinate optimizations were carried out to verify the found transition states are connected to the corresponding intermediate. The Gaussian input line was:

irc=(calcall,tight,maxpoints=200,lqa,maxcycle=512,stepsize=40,noeigen) b3lyp def2tzvp empiricaldispersion=gd3bj int=grid=superfinegrid

Int(endo)

opt=tight freq b3lyp def2tzvp empiricaldispersion=gd3bj int=(grid=superfinegrid)

S 0.05437700 -0.65827100 0.59410800

O -0.18091900 3.04073200 -0.83295200

O -1.64571400 1.33254700 -0.91998300

C -0.62163700 -1.49822900 -0.81538800

C -1.98815800 -1.74866100 -0.63639900

C	-2.51241400	-1.19765200	0.61069900
C	-1.53865100	-0.53379400	1.36682000
C	-1.79477500	0.07595300	2.57892500
H	-1.01646900	0.57227800	3.14199900
C	-3.10322600	0.03602500	3.05032200
H	-3.34668200	0.50933600	3.99126200
C	-4.09432000	-0.61832800	2.32205900
H	-5.10456900	-0.64653800	2.70699000
C	-3.80833900	-1.23856700	1.11100000
H	-4.59077800	-1.74275900	0.56062600
C	-2.65416400	-2.45633400	-1.62975200
H	-3.70892600	-2.67376700	-1.53206800
C	-1.95459200	-2.87916400	-2.75461800
H	-2.47608600	-3.42506100	-3.52901200
C	-0.59358600	-2.61926200	-2.89906400
H	-0.06781900	-2.96455900	-3.77832600
C	0.10015600	-1.92687600	-1.91150500
H	1.15997300	-1.73560900	-1.99839700
C	0.50047500	1.00810400	0.08498700
C	1.95663500	1.21989800	-0.38255200
C	2.91076000	0.10954600	-0.12521100
C	3.57046900	-0.69701300	-1.04143900

C	4.39745600	-1.70858400	-0.56179900
H	4.92826500	-2.34432100	-1.25746400
C	4.55011100	-1.90548700	0.80838700
H	5.19768400	-2.69495900	1.16537000
C	3.89145700	-1.08857200	1.72457600
H	4.02814300	-1.24140000	2.78749400
C	3.07974600	-0.07110400	1.25044400
C	2.29089500	0.95726200	2.02800900
C	1.52567300	1.73142900	0.95618900
C	-0.57922300	1.79256800	-0.60520100
C	-1.12241700	3.91016600	-1.53708300
H	-1.35337700	3.44479400	-2.49472200
H	-2.03813400	3.95466500	-0.94866700
C	-0.46628900	5.25807800	-1.69084400
H	-1.14670000	5.93078600	-2.21442800
H	-0.23477600	5.69688700	-0.72008200
H	0.45339400	5.18517400	-2.27171000
H	2.05362900	1.85674700	-1.24943900
H	2.95301200	1.64742800	2.55536400
H	1.64214600	0.51178400	2.78676100
H	1.30244400	2.77518900	1.11336300
H	3.45640800	-0.53839900	-2.10653300

Int(exo)

opt=tight freq b3lyp def2tzvp empiricaldispersion=gd3bj int=(grid=superfinegrid)

S	1.00366000	0.54559700	-1.61113100
O	-0.55086200	-1.63750500	0.65592900
O	-0.35109400	0.47789800	1.40928400
C	2.08476900	-0.73088300	-1.00527700
C	2.91098500	-0.24661800	0.01714700
C	2.69279800	1.17036500	0.30469400
C	1.68710400	1.73859000	-0.48689700
C	1.30353000	3.06246500	-0.40144100
H	0.52711700	3.47172700	-1.03206500
C	1.95065500	3.85428700	0.54054400
H	1.67412500	4.89412700	0.64415800
C	2.95418300	3.31720700	1.34400700
H	3.45023700	3.94988700	2.06730400
C	3.33373200	1.98465500	1.23030700
H	4.11955700	1.58756800	1.85810900
C	3.79787800	-1.13697700	0.61315900
H	4.45767800	-0.80473700	1.40287600
C	3.82937700	-2.45949300	0.18497400
H	4.51945900	-3.15052900	0.64979600
C	2.99433400	-2.91001300	-0.83591000

H	3.03989100	-3.94137500	-1.15660800
C	2.10496000	-2.03742200	-1.45345800
H	1.45167400	-2.37613500	-2.24512500
C	-0.60409000	0.09537900	-0.93164500
C	-1.76426400	0.92991000	-1.44974800
C	-2.95623300	0.85796400	-0.55956100
C	-3.52100600	1.86070800	0.21280400
C	-4.61083600	1.54500800	1.01684700
H	-5.07084900	2.31295300	1.62381400
C	-5.10842400	0.24454100	1.05416500
H	-5.95517100	0.01466700	1.68701800
C	-4.53352200	-0.76077700	0.28145900
H	-4.93301300	-1.76662700	0.30948300
C	-3.45879700	-0.44671300	-0.53579500
C	-2.69989300	-1.34850800	-1.48257600
C	-1.55129400	-0.47250300	-1.96890500
C	-0.50865500	-0.31637900	0.52211600
C	-0.41372400	-2.15624900	2.01207800
H	0.49847300	-1.73999000	2.43884100
H	-1.26005300	-1.79298100	2.59458300
C	-0.37439000	-3.66017800	1.91873600
H	-0.27153500	-4.08286500	2.91880900

H	-1.29104700	-4.05184300	1.47743200
H	0.47335500	-3.99159200	1.31825500
H	-1.55944400	1.81227900	-2.04080400
H	-3.31867900	-1.63006000	-2.33807300
H	-2.35410600	-2.26731400	-1.01082500
H	-1.15478700	-0.63606300	-2.96122500
H	-3.12086800	2.86600300	0.19969400

TS1(endo)

opt=(calcall,tight,ts,maxstep=6,maxcycle=900,noeigen) freq b3lyp def2tzvp empiricaldispersion=gd3bj int=(grid=superfinegrid)

S	-0.14457200	0.18905000	1.16539900
O	1.63203200	-1.93805700	-1.07064200
O	0.97024200	-0.06278100	-2.14372600
C	-0.09160200	1.74659700	0.34787100
C	1.22905300	2.19017400	0.16354000
C	2.20942200	1.26688800	0.71899500
C	1.61186800	0.14219800	1.31319800
C	2.34441400	-0.86216600	1.92529400
H	1.85982300	-1.71445900	2.38108500
C	3.72799700	-0.74126600	1.93469400
H	4.32777100	-1.50548600	2.40943100
C	4.34610200	0.36197200	1.34541700
H	5.42445000	0.44218000	1.36699900

C	3.59807600	1.36516800	0.74250400
H	4.09130100	2.21949600	0.29909200
C	1.43367700	3.40198100	-0.48722800
H	2.43665900	3.77490300	-0.64415800
C	0.34001000	4.13073800	-0.93602800
H	0.50002400	5.07213200	-1.44363200
C	-0.96118300	3.67176900	-0.73524300
H	-1.79928600	4.26072500	-1.08162800
C	-1.19320900	2.46670600	-0.08360400
H	-2.19855200	2.11062300	0.08739100
C	-0.52212700	-1.13531000	-0.63872100
C	-1.75102400	-0.85344000	-1.30458300
C	-2.99774200	-0.65979900	-0.58373000
C	-3.94240000	0.31066600	-0.92358800
C	-5.03192000	0.50877600	-0.08871000
H	-5.76686100	1.26300300	-0.33496600
C	-5.18544800	-0.25845800	1.06531200
H	-6.04220300	-0.09798000	1.70556400
C	-4.26733700	-1.25430600	1.38086000
H	-4.41767400	-1.87703300	2.25345000
C	-3.18705700	-1.47010600	0.53957400
C	-2.20278800	-2.59897400	0.62135600

C -1.09919200 -2.39704700 -0.37050000

C 0.77441300 -0.97880200 -1.38987400

C 2.96870300 -1.83607900 -1.65248800

H 2.86133400 -1.79350600 -2.73555400

H 3.40034500 -0.89545800 -1.31139200

C 3.75852000 -3.03394500 -1.19352500

H 4.76541700 -2.98122300 -1.60935900

H 3.83842100 -3.05656100 -0.10703500

H 3.29971400 -3.96270600 -1.53299800

H -1.68062500 -0.59933500 -2.35642500

H -2.69754700 -3.55317500 0.43109300

H -1.75381000 -2.67985900 1.62038900

H -0.60069300 -3.26522800 -0.78389000

H -3.80840800 0.91723800 -1.80965500

TS1(exo)

opt=(calcall,tight,ts,maxstep=6,maxcycle=900,noeigen) freq b3lyp def2tzvp empiricaldispersion=gd3bj int=(grid=superfinegrid)

S -1.04890800 0.44465600 1.68719300

O -0.44165400 -2.25656300 -0.45466800

O -0.33184700 -0.27970700 -1.56143800

C -2.49157200 -0.18746100 0.92882800

C -2.97306300 0.67209300 -0.07803500

C -2.18241000 1.89037600 -0.18387500

C	-1.13307200	1.92375500	0.75371000
C	-0.28452800	3.01671500	0.88253700
H	0.47362800	3.05268300	1.65368100
C	-0.46915300	4.09061000	0.01975100
H	0.17139700	4.95790600	0.10167300
C	-1.48767000	4.06601400	-0.93169700
H	-1.62085800	4.91273400	-1.59100900
C	-2.34880900	2.97900700	-1.03155600
H	-3.14500000	2.98421000	-1.76362600
C	-4.10571600	0.28878600	-0.78912200
H	-4.50170600	0.92426800	-1.56952200
C	-4.73147800	-0.91383800	-0.48433200
H	-5.61642700	-1.20659300	-1.03289800
C	-4.24448500	-1.74497700	0.52698300
H	-4.75546700	-2.66993900	0.75598300
C	-3.11109600	-1.39099100	1.24341900
H	-2.72648400	-2.02956300	2.02650900
C	1.08063700	-0.67259400	0.26940300
C	1.99169900	0.38617200	0.43942900
C	3.41517600	0.28068900	0.02545400
C	4.06068900	1.21867300	-0.76807300
C	5.36875600	0.95634500	-1.16177600

H	5.89326400	1.67150000	-1.78069800
C	6.00429100	-0.21722300	-0.76724700
H	7.02299300	-0.40510800	-1.07807100
C	5.35012700	-1.14349000	0.04028500
H	5.85679000	-2.04531200	0.35868600
C	4.05263000	-0.87858800	0.45036500
C	3.20066400	-1.67996100	1.38180100
C	1.80834600	-1.05136000	1.41890400
C	0.00459900	-1.02503300	-0.67464100
C	-1.39322100	-2.77606600	-1.43660500
H	-2.24313300	-2.09731900	-1.47218900
H	-0.90083700	-2.76005800	-2.40896700
C	-1.77807400	-4.16685300	-1.00643600
H	-2.48386800	-4.58248200	-1.72663000
H	-0.90846300	-4.82259600	-0.96479800
H	-2.25650500	-4.15592500	-0.02737500
H	1.57395100	1.38790100	0.51787400
H	3.60336500	-1.67155700	2.39593000
H	3.10313800	-2.72781900	1.08267900
H	1.25431500	-1.18681600	2.34028900
H	3.55684400	2.12071900	-1.08861500

REFERENCES

1 (a) A. Kaupang and T. Bonge-Hansen, *Beilstein J. Org. Chem.* 2013, **9**, 1407−1413; (b) T. Toma, J. Shimokawa and T. Fukuyama, *Org. Lett.* 2007, **9**, 3195−3197.

2 B. Waldecker, K. Kafuta and M. Alcarazo *Org. Synth.* 2019, **96**, 258−276.

3 (a) W.-F. Yang, T. Shu, H.-R. Chen, H.-L. Qin and H. Tang, *Org. Biomol. Chem.* 2022, **20**, 3506−3510; (b) Z. Luo, Y. Meng, X. Gong, J. Wu, Y. Zhang, L.-W. Ye and C. Zhu, *Chin. J. Chem.* 2020, **38**, 173−177.

4 G. Pisella, A. Gagnebin and J. Waser, *Org. Lett.* 2020, **22**, 3884−3889.

5 R. P. Pandit, S. H. Kim and Y. R. Lee, *Adv. Synth. Catal.* 2016, **358**, 3586−3599.

6 W.-M. Shu, J.-R. Ma, K.-L. Zheng, H.-Y. Sun, M. Wang, Y. Yang and A.-X. Wu, *Tetrahedron* 2014, **70**, 9321−9329.

7 Y. Wang, M. J. Bartlett, C. A. Denard, J. F. Hartwig and H. Zhao, *ACS Catal.* 2017, **7**, 2548−2552.

8 (a) F. J. Sarabia and E. M. Ferreira, *Org. Lett.* 2017, **19**, 2865−2868; (b) B. Musio, F. Mariani, E. Śliwiński, M. Kabeshov, H. Odajima and S. Ley, *Synthesis* 2016, **48**, 3515−3526.

9 (a) X. Zhao, J.-Y. Shou, J. J. Newton and F.-L. Qing, *Org. Lett.* 2022, **24**, 8412−8416; (b) D. M. Hodgson and D. Angrish, *Chem. Eur. J.* 2007, **13**, 3470−3479.

10 S. Hyde, J. Veliks, B. Liégault, D. Grassi, M. Taillefer and V. Gouverneur, *Angew. Chem. Int. Ed.* 2016, **55**, 3785−3789.

11 (a) Y. Yang, R. Tang, S. Abid, L. Khrouz, J. C. Vantourout and A. Tlili, *Eur. J. Org. Chem.* 2023, **26**, e202300619; (b) A. E. Bosnidou and K. Muñiz, *Angew. Chem. Int. Ed.* 2019, **58**, 7485−7489.

12 G. M. Sheldrick, *Acta. Cryst.* 2008, **A64**, 112−122.

13 O. V. Dolomanov, L. J. Bourhis, R. J. Gildea, J. A. K. Howard and H. Puschmann, *J. Appl. Cryst.* 2009, **42**, 339−341.

14 T. Kottke and D. Stalke, *J. Appl. Cryst.* 1993, **26**, 615−619.

15 X. Li, C. Golz and M. Alcarazo, *Angew. Chem. Int. Ed.* 2021, **60**, 6943−6948.

16 S. Pearson, S. Fillery, K. Goldberg, J. Demeritt, J. Eden, J. Finlayson and A. Patel, *Synthesis* 2018, **50**, 4963−4981.

17 J. Bucher, T. Stößer, M. Rudolph, F. Rominger and A. S. K. Hashmi, *Angew. Chem. Int. Ed.* 2015, **54**, 1666−1670.

18 R. Iwai, S. Suzuki, S. Sasaki, A. S. Sairi, K. Igawa, T. Suenobu, K. Morokuma and G.-I. Konishi, *Angew. Chem. Int. Ed.* 2020, **59**, 10566−10573.

19 R. Deng, L. Sun and Z. Li, *Org. Lett.* 2007, **9**, 5207−5210.

20 T. Fujino, T. Hyodo, Y. Otani, K. Yamaguchi and T. Ohwada, *J. Org. Chem.* 2022, **87**, 12653−12672.

21 B. Michelet, C. Bour and V. Gandon, *Chem. Eur. J.* 2014, **20**, 14488−14492.

22 N. G. Léonard, W. N. Palmer, M. R. Friedfeld, M. J. Bezdek and P. J. Chirik, *ACS Catal.* 2019, **9**, 9034−9044.

23 C. Vila, V. Hornillos, M. Giannerini, M. Fañanás-Mastral and B. L. Feringa, *Chem. Eur. J.* 2014, **20**, 13078−13083.

24 E. Villaseñor, R. Gutierrez-Gonzalez, F. Carrillo-Hermosilla, R. Fernández-Galán, I. López-Solera, A. R. Fernández-Pacheco and A. Antiñolo, *Eur. J. Inorg. Chem.* 2013, **2013**, 1184−1196.

25 A. Monrose, H. Salembier, T. Bousquet, S. Pellegrini and L. Pélinski, *Adv. Synth. Catal.* 2017, **359**, 2699−2704.

26 D. Kang, J. Kim, S. Oh and P. H. Lee, *Org. Lett.* 2012, **14**, 5636−5639.

27 Y. Iyori, K. Takahashi, K. Yamazaki, Y. Ano and N. Chatani, *Chem. Commun.* 2019, **55**, 13610−13613.

28 A. Cappelli, G. La Pericot Mohr Gl, A. Gallelli, M. Rizzo, M. Anzini, S. Vomero, L. Mennuni, F. Ferrari, F. Makovec, M. C. Menziani, P. G. De Benedetti and G. Giorgi, *J. Med. Chem.* 2004, **47**, 2574−2586.

29 J. W. Lim, K. H. Kim, S. H. Kim and J. N. Kim, *Tetrahedron Lett.* 2012, **53**, 5449−5454.

30 B. Lu, F. Zhu, H.-M. Sun and Q. Shen, *Org. Lett.* 2017, **19**, 1132−1135.

31 F.-P. Wu, C. C. Chintawar, R. Lalisse, P. Mukherjee, S. Dutta, J. Tyler, C. G. Daniliuc, O. Gutierrez and F. Glorius, *Nat. Catal.* 2024. (DOI: 10.1038/s41929-023-01089-x)

32 B. Spingler, S. Schnidrig, T. Todorova and F. Wild, *CrystEngComm* 2012, **14**, 751−757.

33 Gaussian 16, Revision A.03, M. J. Frisch, G. W. Trucks, H. B. Schlegel, G. E. Scuseria, M. A. Robb, J. R. Cheeseman, G. Scalmani, V. Barone, G. A. Petersson, H. Nakatsuji, X. Li, M. Caricato, A. V. Marenich, J. Bloino, B. G. Janesko, R. Gomperts, B. Mennucci, H. P. Hratchian, J. V. Ortiz, A. F. Izmaylov, J. L. Sonnenberg, D. Williams-Young, F. Ding, F. Lipparini, F. Egidi, J. Goings, B. Peng, A. Petrone, T. Henderson, D. Ranasinghe, V. G. Zakrzewski, J. Gao, N. Rega, G. Zheng, W. Liang, M. Hada, M. Ehara, K. Toyota, R. Fukuda, J. Hasegawa, M. Ishida, T. Nakajima, Y. Honda, O. Kitao, H. Nakai, T. Vreven, K. Throssell, J. A. Montgomery, Jr., J. E. Peralta, F. Ogliaro, M. J. Bearpark, J. J. Heyd, E. N. Brothers, K. N. Kudin, V. N. Staroverov, T. A. Keith, R. Kobayashi, J. Normand, K. Raghavachari, A. P. Rendell, J. C. Burant, S. S. Iyengar, J. Tomasi, M. Cossi, J. M. Millam, M. Klene, C. Adamo, R. Cammi, J. W. Ochterski, R. L. Martin, K. Morokuma, O. Farkas, J. B. Foresman, and D. J. Fox, Gaussian, Inc., Wallingford CT, 2016.
